# Multiple massive domestication and recent amplification of *Kolobok* superfamily transposons in the clawed frog *Xenopus*

**DOI:** 10.1186/s40851-018-0100-4

**Published:** 2018-06-16

**Authors:** Akira Hikosaka, Seigo Konishi

**Affiliations:** Graduate School of Integrated Arts and Sciences, Hiroshima University, Kagamiyama 1-7-1, Higashi-Hiroshima, Hiroshima Japan

**Keywords:** *Xenopus laevis*, *Xenopus tropicalis*, DNA transposon, *Kolobok*, T2-MITE, Transposase, Domestication, Molecular evolution

## Abstract

**Background:**

DNA transposons are generally destroyed by mutations and have short lifespans in hosts, as they are neutral or harmful to the host and therefore not conserved by natural selection. The clawed frog *Xenopus* harbors many DNA transposons and certain families, such as T2-MITE, have extremely long lives. These have ancient origins, but have shown recent transposition activity. In addition, certain transposase genes may have been “domesticated” by *Xenopus* and conserved over long time periods by natural selection. The aim of this study was to elucidate the evolutionary interactions between the host and the long-lived DNA transposon family it contains. Here, we investigated the molecular evolution of the *Kolobok* DNA transposon superfamily. *Kolobok* is thought to contribute to T2-MITE transposition.

**Results:**

In the diploid western clawed frog *Xenopus tropicalis* and the allotetraploid African clawed frog *Xenopus laevis*, we searched for transposase genes homologous to those in the *Kolobok* superfamily. To determine the amplification and domestication of these genes, we used molecular phylogenetics and analyses of copy numbers, conserved motifs, orthologous gene synteny, and coding sequence divergence between the orthologs of *X. laevis* and *X. tropicalis*, or between those of two distant *X. tropicalis* lineages. Among 38 *X. tropicalis* and 24 *X. laevis* prospective transposase genes, 10 or more in *X. tropicalis* and 14 or more in *X. laevis* were apparently domesticated. These genes may have undergone multiple independent domestications from before the divergence of *X. laevis* and *X. tropicalis.* In contrast, certain other transposases may have retained catalytic activity required for transposition and could therefore have been recently amplified.

**Conclusion:**

Multiple domestication of certain transposases and prolonged conservation of the catalytic activity in others suggest that *Kolobok* superfamily transposons were involved in complex, mutually beneficial relationships with their *Xenopus* hosts. Some transposases may serve to activate long-lived T2-MITE subfamilies.

**Electronic supplementary material:**

The online version of this article (10.1186/s40851-018-0100-4) contains supplementary material, which is available to authorized users.

## Background

Transposable elements (TEs) are endosymbiotic or parasitic genetic elements in the host genome. These elements usually replicate at their loci in the same manner as host genes. Nevertheless, they occasionally transpose to other loci and, in some cases, amplify themselves in the host genome. This non-Mendelian inheritance creates a conflict between TEs and their hosts. Transposable elements promote their amplification rates to increase their “fitness” in the host. In effect, their transposition and amplification are generally neutral, but occasionally detrimental, to the host as they increase mutations, such as gene disruption and ectopic recombination. In general, then, TE copies are not conserved. They are removed by purifying selection, and they lose their structure and function as they accumulate mutations.

TEs are classified into two major classes according to their transposition mechanisms: class I (RNA transposons or retrotransposons) and class II (DNA transposons) [1, 2]. An autonomous DNA transposon encodes its own transposase to catalyze its transposition between terminal inverted repeats (TIRs) including the transposase recognition site. In contrast, a nonautonomous DNA transposon has lost its own transposase gene owing to mutation(s). Its transposition depends on transposases provided by autonomous transposons coexisting in the genome. Autonomous transposons statistically lose their activity over time without purifying selection and become nonautonomous or nontransposable. Over time, the host genome will accumulate many nonautonomous copies and nontransposable “fossils” and retain relatively few autonomous copies. The TE family must keep producing autonomous copies to persist in the host. However, the transposase protein catalyzes the transposition of many nonautonomous copies and few autonomous copies, without distinguishing between them. Therefore, the probability that autonomous copies are transposed and amplified gradually decreases as the ratio of nonautonomous copies increases in the genome. Most DNA transposon families eventually become extinct in a host genome. This process is known as “vertical inactivation” [3, 4].

An alternative strategy for TE survival in a host is “molecular domestication.” If a TE-derived gene, such as a transposase, has mutated and become beneficial to the host, it can be conserved by natural selection [5, 6]. Domesticated genes typically lose their original nature (that is, the ability to catalyze transposition/amplification) and behave like host genes. Over time, domesticated genes diverge from their copies produced before domestication. As a result, they become and remain nonrepetitive (single-copy) genes. Domesticating a parasitic element is an important evolutionary innovation for hosts. Many TE-derived proteins have been domesticated [7, 8]. In the clawed frog *Xenopus*, we found that two subfamilies of the *TxpB* family belonging to the *piggyBac* superfamily have employed different survival strategies. The transposase-derived gene of the *Kobuta* subfamily was domesticated before the separation of *X. tropicalis* and *X. laevis*. In contrast, the transposase of the *Uribo2* subfamily retained its catalytic activity and can still excise transposons from DNA [9].

Nonautonomous TEs can also be domesticated. Miniature inverted-repeat transposable elements (MITEs) are a subclass of short nonautonomous DNA transposons. These are characterized by a TIR structure, high copy numbers, and highly similar sequences among copies [10]. MITEs generate functional transcriptional regulatory elements [11] and matrix attachment regions [12]. In *Xenopus*, MITEs also form simple sequence repeats (SSRs). Xmix, a predominant MITE in *X. laevis* and *X. tropicalis*, has an amplified internal segment representing a large SSR family, Xstir [13, 14]. Simple sequence repeats are essential for higher-order chromosomal structure [15].

Xmix is a member of the T2 MITE family (T2-MITE), which is characterized by a TTAA target site and a terminal AGGRR (R: A or G) motif in its TIR [16, 17]. These features are common to members of the DNA transposon superfamily *Kolobok*, which targets the TTAA site and has an RR terminal sequence [18]. Therefore, T2-MITE is presumably a nonautonomous member of the *Kolobok* DNA transposon superfamily. We classified 16 major T2-MITE subfamilies based on an in silico screening of the *X. tropicalis* genome sequence [19]. Subfamilies A1 (T2-A1, Xmix) and C (T2-C) were the most prevalent and were present in both *X. tropicalis* and *X. laevis.* They probably originated before these two lineages diverged ~ 48 Mya [20]. Despite their age, both subfamilies include “young” (highly homogeneous) members. Therefore, they probably underwent relatively recent amplification. We also found evidence for intraspecific T2-A1 and T2-C insertion polymorphisms [21]. These results suggest that these subfamilies have been actively transposing for more than 48 million years. Their extraordinary longevity suggests that their continued transpositional activity has been conserved by natural selection. The transposition and/or amplification of these subfamilies presumably have been advantageous to the host. We found that the sequences derived from T2-C in *X. tropicalis* were significantly over-represented in the 5′ upstream regions of genes. Therefore, they may regulate the expression of neighboring genes [22]*.*

If the transpositional activities of T2-MITEs are conserved by natural selection, then transposases such as *Kolobok*, which are presumably responsible for their transposition, should be domesticated by the host. In the present study, we surveyed *Kolobok* transposase genes in the diploid *X. tropicalis* genome and the recently decoded allotetraploid *X. laevis* genome [20]. To elucidate their evolution within the hosts, we analyzed their molecular phylogenies, copy numbers, syntenies, and sequence conservation.

## Methods

### Search for prospective *Kolobok* transposase-coding sequences

*X. tropicnalis* (Nigerian 9.1) and *X. laevis* (J-Strain 9.2) genome assemblies were downloaded from the Xenbase FTP site [23, 24] and used in the analyses described below. An automated pipeline for these analyses was developed using the Ruby language.

The search for prospective *Kolobok* transposase-coding sequences (CDSs) was carried out as follows. Query *Kolobok* superfamily transposase protein sequences were collected from vertebrate, zebrafish, and invertebrate data sets using Repbase Update [25] v. 22.09 [26] (Additional file 5: Table S1) and applied to a tblastn search (e-value <1e-5) [27]. The hit regions were extracted with flanking sequences (1800 bp each upstream and downstream). The longest open reading frames (ORFs) in each extracted sequence were treated as candidate *Kolobok* transposase CDSs. ORFs that were too short (< 1800 bp) were excluded from the following analyses. To confirm the homology of proteins encoded in the candidate CDSs to the *Kolobok* transposases, the CDSs were translated to amino acid sequences and used as queries in backward homology searches (blastp, e-value <1e-5) to the transposase sequences used as the queries in the forward tblastn search. Candidate CDSs with prospective amino acid sequences that were homologous to at least five *Kolobok* transposases were selected. The protein sequences predicted from the candidate CDSs were aligned by MEGA7 [28] using MUSCLE [29] as an alignment engine. The positions of their start methionines were verified. Excess 5′ regions were manually trimmed from the candidate CDSs to align the translation initiation site. Trimmed CDSs and protein sequences were used in the analyses described below.

### Copy number analysis

Prospective *Kolobok* transposase CDSs from *X. laevis* and *X. tropicalis* were used as queries for blastn searches (e-value <1e-100) to the corresponding genome. Adjacent high-scoring segment pairs were considered single hit regions if the distance between them was less than the query length. Hit regions in each prospective CDS were counted as closely related truncated copies of the CDS if they did not overlap with any prospective CDSs.

### Molecular phylogenetics and synteny analyses

Prospective *Kolobok* transposase CDSs from both species were translated to protein sequences and used in molecular phylogenetic analyses. The amino acid sequences were realigned with MEGA7 and the alignment engine MUSCLE and used to construct phylogenetic trees. The neighbor-joining method [30] was used to generate the phylogenetic trees with MEGA7. Positions with less than 60% site coverage were eliminated. The evolutionary distances were computed using the JTT matrix-based method [31]. Rate variations among sites were modeled with a gamma distribution (shape parameter = 1). Dot plot analyses were performed with the polydot program in EMBOSS [32]. The word sizes for the nucleotide and protein sequences were 12 and 5, respectively.

Putative orthologous transposase sets were collected with reference to the phylogenetic tree. NCBI gene models (XT9_1_GCA.gff3 and XL9_2_GCA.gff3) were downloaded from the Xenbase FTP site [23, 24]. Gene models around the putative orthologous CDSs were compared. The CDS orthologs were defined as those located on the homologous *X. tropicalis* chromosome and *X. laevis* L/S chromosomes and flanked by multiple orthologous neighbor genes.

The ratios of synonymous and nonsynonymous substitutions between two coding DNA sequences (dN/dS ratio) were calculated by the method of Yang and Nielsen [33] using the yn00 program of PAML v. 4.8 [34].

### Search for full-length *Kolobok* transposons

The upstream and downstream sequences flanking the CDSs of each repetitive *XKol* subfamily were compared by dot plot analysis and multiple alignment to find the left and right TIRs, respectively. The left and right terminal sequences were compared to confirm their similarity. The sequences between similar left and right TIRs were considered as full-length TEs. Four-base pair sequences flanking the full-length TEs were examined to check the conservation of their duplicated target sites (TTAA).

### Cloning of *XKol*-Tpases from two *X. tropicalis* lineages

*X. tropicalis* was provided by the National Bioresource Project, Japan [35]. Genomic DNAs were extracted from the Nigerian and Asashima lines using a previously described method [13]. Polymerase chain reaction (PCR) was performed using KOD-Plus DNA polymerase (Toyobo Co. Ltd., Osaka, Japan) and 100 ng genomic DNA. The default PCR conditions were as follows: initial denaturation (94 °C, 120 s), followed by 35 cycles of denaturation (98 °C, 10 s), annealing (60 °C, 30 s), and extension (68 °C, 90 s). The annealing temperature, extension time, and/or PCR cycles were changed as needed to optimize amplification. The primers used in the PCR are shown in (Additional file 6: Table S2). PCR products were inserted into the pCRBluntII-TOPO or pCR4Blunt-TOPO vector (Invitrogen, Carlsbad, CA, USA) and cloned into OneShotTOP-10 competent cells (Invitrogen). The cloned sequences were analyzed using a BigDye3.1 Terminator Cycle Sequencing Kit (Applied Biosystems, Foster City, CA, USA) and an ABI PRISM 310 or 3100 Genetic Analyzer (Applied Biosystems).

## Results

### Prospective *Kolobok* transposase CDSs and proteins

**Fig. 1:**
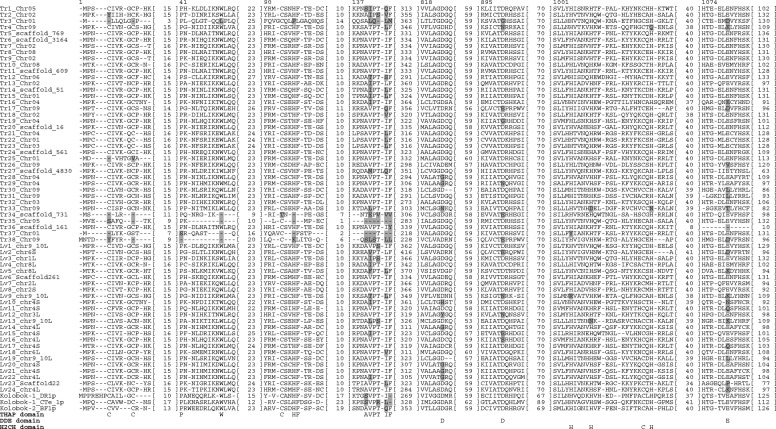
Conserved regions of prospective *XKol* transposase CDSs. Conserved regions in a multiple alignment of prospective transposases predicted from the putative CDSs of *XKol*-Tpase genes with three outgroup transposases (*Danio rerio* Kolobok-1_DR, *Capitella teleta* Kolobok-1_CTe, and *Branchiostoma floridae* Kolobok-2_BF). Numbers above the alignment indicate the position of the amino acids in it (Additional file 1: Figure S1). Numbers in square brackets are the abbreviated amino acids. Consensus residues of conserved domains (DDE, THAP, and H2CH) are shown below the alignment. Nonconserved amino acid residues in conserved motifs are marked by gray shading

To identify candidates of active (able to catalyze TE transposition) or domesticated *Kolobok* transposase genes, we performed tblastn (protein query versus nucleotide database) homology searches of *X. tropicalis* and *X. laevis* genomes. We searched the hit loci and their surrounding regions for the longest ORFs that encoded proteins homologous to *Kolobok* transposases. Multiple alignments of putative proteins encoded in the longest ORFs revealed that the *N*-terminal sequences encoded by certain ORFs were longer than those of other ORFs. We trimmed the excess 5′ regions from these ORFs to align their start codons with those of the majority genes (Additional file 1: Figure S1). Figure 1 shows the *N*-terminal region and some conserved regions of the aligned proteins. It is unclear whether these trimmed ORFs were the actual CDSs of *Kolobok* transposase genes. Nevertheless, we expected that most of these ORFs coincided or overlapped with the CDSs. Therefore, we ascribed them to be CDSs of prospective *Kolobok* transposase genes. In *X. tropicalis* and *X. laevis*, 38 and 24 prospective CDSs were found, respectively. We referred to the prospective *Xenopus Kolobok* transposons as the *XKol* family and its transposase as *XKol*-Tpase. We designated the *XKol*-Tpase CDSs and proteins serially as Tr1–Tr38 for *X. tropicalis* and Lv1–Lv24 for *X. laevis* (Table 1). The five shortest *XKol*-Tpase proteins (Tr34–Tr38) lacked a certain number of conserved amino acids (Fig. 1 and Table 2). Therefore, they may not be functional. The transposases encoded in the Kolobok-1_XT and Kolobok-2_XT transposons previously reported in the RepBase [18] most resembled, but were not identical to, Tr8 (723/783 identical) and Tr7 (782/783 identical), respectively.

**Table 1:** Prospective *Xenopus XKol* transposase CDSs

Name	Locus	Longest ORF	Trimmed CDS	No. of truncated copies
A. Prospective *X. tropicalis XKol* transposase CDSs				
Tr1	Chr05:4592573..4595167(−)	2595 bp	2202 bp	1
Tr2	Chr02:100830975..100833458(+)	2484 bp	2421 bp	3
Tr3	Chr01:90056839..90059247(+)	2409 bp	2409 bp	0
Tr4	Chr09:49289527..49291908(+)	2382 bp	2319 bp	0
Tr5	scaffold_769:44126..46486(−)	2361 bp	2361 bp	10
Tr6	scaffold_3164:2182..4536(−)	2355 bp	2355 bp	5
Tr7	Chr02:77614330..77616681(−)	2352 bp	2352 bp	2
Tr8	Chr08:54548260..54550611(+)	2352 bp	2352 bp	9
Tr9	Chr02:55486066..55488417(+)	2352 bp	2352 bp	2
Tr10	Chr08:50277255..50279597(+)	2343 bp	2292 bp	0
Tr11	scaffold_609:18293..20635(−)	2343 bp	2343 bp	9
Tr12	Chr06:112308032..112310368(−)	2337 bp	2250 bp	9
Tr13	Chr02:162067553..162069883(−)	2331 bp	2331 bp	0
Tr14	scaffold_51:231432..233759(−)	2328 bp	2328 bp	5
Tr15	Chr01:83498100..83500424(+)	2325 bp	2325 bp	5
Tr16	Chr04:108160710..108163031(−)	2322 bp	2322 bp	0
Tr17	Chr09:29639969..29642278(−)	2310 bp	2310 bp	0
Tr18	Chr02:66952233..66954539(−)	2307 bp	2307 bp	0
Tr19	Chr04:41679747..41682050(+)	2304 bp	2229 bp	0
Tr20	scaffold_16:1256554..1258851(+)	2298 bp	2298 bp	4
Tr21	Chr04:107657217..107659511(+)	2295 bp	2295 bp	9
Tr22	Chr01:168455606..168457900(−)	2295 bp	2295 bp	9
Tr23	Chr03:106168762..106171056(−)	2295 bp	2295 bp	9
Tr24	scaffold_561:30795..33086(−)	2292 bp	2256 bp	0
Tr25	Chr01:107512548..107514821(+)	2274 bp	2274 bp	4
Tr26	Chr09:13318232..13320439(−)	2208 bp	2208 bp	0
Tr27	scaffold_4830:88..2292(−)	2205 bp	2205 bp	5
Tr28	Chr04:128881775..128883943(−)	2169 bp	2169 bp	0
Tr29	Chr04:128872195..128874363(−)	2169 bp	2169 bp	0
Tr30	Chr09:10540280..10542436(+)	2157 bp	2157 bp	0
Tr31	Chr09:10547870..10549993(+)	2124 bp	2091 bp	0
Tr32	Chr03:80573408..80575504(+)	2097 bp	2097 bp	0
Tr33	Chr09:10557320..10559413(+)	2094 bp	2094 bp	1*
Tr34	scaffold_731:1892..3937(−)	2046 bp	2046 bp	0
Tr35	Chr05:37113502..37115535(−)	2034 bp	2034 bp	3
Tr36	scaffold_161:120530..122407(+)	1878 bp	1878 bp	8
Tr37	Chr01:130854739..130856610(−)	1872 bp	1872 bp	0
Tr38	Chr09:29654976..29656787(−)	1812 bp	1812 bp	0
B. Prospective *X. laevis XKol* transposase CDSs				
Lv1	chr9_10L:117582522..117585011(−)	2490 bp	2433 bp	4
Lv2	chr6S:1512718..1515198(−)	2481 bp	2481 bp	1*
Lv3	chr1L:4995471..4997924(−)	2454 bp	2454 bp	4
Lv4	chr8L:50116481..50118844(+)	2364 bp	2292 bp	0
Lv5	Scaffold261:26626..28977(+)	2352 bp	2352 bp	0
Lv6	chr8L:99349197..99351548(−)	2352 bp	2352 bp	2
Lv7	chr2L:167641114..167643453(−)	2340 bp	2340 bp	0
Lv8	chr2S:147754724..147757057(−)	2334 bp	2334 bp	0
Lv9	chr9_10L:77656066..77658378(+)	2313 bp	2313 bp	0
Lv10	chr4S:91247377..91249686(−)	2310 bp	2310 bp	1*
Lv11	chr4S:1463674..1465929(−)	2256 bp	2217 bp	1*
Lv12	chr3L:58143401..58145647(+)	2247 bp	2211 bp	0
Lv13	chr9_10L:110876140..110878383(−)	2244 bp	2244 bp	0
Lv14	chr4L:133759875..133762109(+)	2235 bp	2187 bp	0
Lv15	chr4S:20852192..20854426(−)	2235 bp	2235 bp	0
Lv16	chr4L:26594086..26596320(+)	2235 bp	2235 bp	1*
Lv17	chr4S:1470489..1472717(−)	2229 bp	2229 bp	2*
Lv18	chr6L:145404209..145406428(−)	2220 bp	2220 bp	0
Lv19	chr9_10L:110897783..110899978(−)	2196 bp	2196 bp	0
Lv20	chr4S:112529046..112531238(−)	2193 bp	2193 bp	0
Lv21	chr4S:112539344..112541533(−)	2190 bp	2190 bp	0
Lv22	chr4L:133769461..133771647(+)	2187 bp	2187 bp	0
Lv23	Scaffold22:1796503..1798647(−)	2145 bp	2145 bp	3
Lv24	chr4L:26633802..26635940(+)	2139 bp	2103 bp	0

Prospective *XKol*-Tpases found in (A) *X. tropicalis* and (B) *X. laevis*. Asterisks in the “No. of truncated copies” column indicate semi-nonrepetitive CDSs

**Table 2:** Amino acid residues in conserved motifs of *Kolobok* transposase

Name	DDE	H2CH	C2CH	PWF	AVPTIF	Conservation
Tr1	DDE	HHCH	CCCH	PWF	SIPTQF	
Tr2	DDE	HHCH	YCCH	PWF	AIPSVF	
Tr3	DGD	HHCH	--CP	PLL	ALQ-LM	
Tr4	DNK	HHCQ	CCCH	PWF	ATPTLF	
Tr5	DDE	HHCH	CCCH	PWF	AVPTIF	++
Tr6	DDE	HHCH	CCCH	PWF	AVPTIF	++
Tr7	DDE	HHCH	CCCH	PWF	AVPTIF	++
Tr8	DDE	HHCH	CCCH	PWF	AVPTIF	++
Tr9	DDE	HHCH	CCCH	PWF	AVPTIF	++
Tr10	DDE	HHCH	CCCH	PWF	AVPTIF	++
Tr11	DDE	HHCH	CCCH	PWF	AVPTIF	++
Tr12	DDE	HHCH	CCCH	PWF	ATPTSF	
Tr13	DDE	HHCH	CCCH	PWF	AIPTLF	+
Tr14	DDE	HHCH	CCCH	PWF	AIPTLF	+
Tr15	DDE	HHCH	CCCH	PWF	AIPTLF	+
Tr16	DDK	HHCH	CCCH	PWF	AIPTIF	
Tr17	DED	HHCH	CCCH	PWF	AVPTQF	
Tr18	DDE	HHCH	CCCH	PWF	AVPTVF	+
Tr19	DGE	HHCH	CCCH	PWF	AVPTIF	
Tr20	DDE	HHCH	CCCH	PWF	AVPTIF	++
Tr21	DDE	HHCH	CCCH	PWF	AVPTLF	+
Tr22	DDE	HHCH	CCCH	PWF	AVPTLF	+
Tr23	DDE	HHCH	CCCH	PWF	AVPTLF	+
Tr24	DDE	HHCH	CCCH	PWF	AVPTIF	++
Tr25	DDE	HHCH	-VCH	PWF	AVPTIF	
Tr26	DDV	HHCH	CCCH	PWF	AVPTIF	
Tr27	DDE	HHCH	CCCH	PWF	AMPTQF	
Tr28	GDE	HHCH	CCCH	PWF	AVPTIF	
Tr29	GNE	HHCH	CCCH	PWF	AVPTIF	
Tr30	DDL	HHCH	CCCH	PWF	AVPTIF	
Tr31	DDL	HHCH	CCCH	PWF	AVPTIF	
Tr32	DDE	HHCH	CCCH	PWF	AVPTIF	++
Tr33	NDT	HDCN	CCCH	PWF	ALPTIF	
Tr34	DEE	HHCH	--T-	P--	TEPVVV	
Tr35	DDE	HHCH	S-C-	P--	–	
Tr36	DD-	HHCH	CCCH	PWF	AVPTIY	
Tr37	DDE	YHCH	-SC-	S-F	---T--	
Tr38	DED	HHCH	Y-CK	--L	SLSTLL	
Lv1	DDE	HHCH	CCCH	PWF	AVPSIF	+
Lv2	DED	HHCH	CCCH	PWF	AVPTLF	
Lv3	DDE	HHCH	CCCH	PWF	AIPSIF	+
Lv4	DDE	HHCH	CCCH	PWF	AIPTIF	+
Lv5	DDK	HHCH	CCCH	PWF	AFPTLF	
Lv6	DDE	HHCH	CCCH	PWF	AVPTIF	++
Lv7	DDE	HHCH	CCCH	PWF	AVPTIF	++
Lv8	DDE	HHCH	CCCH	PWF	AVPTIF	++
Lv9	DNK	RHCH	CCCH	PWF	ATPTLF	
Lv10	HDN	HHCH	CCCH	PWF	AIPTIF	
Lv11	DDQ	HHCH	CCCH	PWF	AVPTIF	
Lv12	YNE	HHCH	CCCH	PWF	AVPTIF	
Lv13	DDK	HNCH	CCCH	PWF	ALPSIF	
Lv14	GDE	HHCH	CCCH	PWF	AVPTIF	
Lv15	DEE	HHCH	CCCH	PWF	AIPTIF	
Lv16	DSE	HHCH	CCCH	PWF	AVPTIF	
Lv17	DDQ	HHCH	CCCH	PWF	AVPTIF	
Lv18	DDE	HHCH	CCCH	PWF	AVPTVF	+
Lv19	DDL	HHCH	CCCH	PWF	AVPTIF	
Lv20	GDE	HHCH	CCCH	PWF	AVPTIF	
Lv21	GDE	HHCH	CCCH	PWF	AVPTIF	
Lv22	GDE	HHCH	CCCH	PWF	AIPTIF	
Lv23	DDP	HHCH	CCCH	PWF	AVPTLF	
Lv24	DDK	HHCH	CCCH	PWF	AVPTIF	

The “++” in the “Conservation” column indicates that all motifs are conserved. The “+” in the same column indicates that all motifs are conserved except for substitutions of similar amino acids in the AVPTIF motif

*Kolobok* superfamily transposases usually possess three motifs: a catalytic “DDE” domain, a THAP DNA-binding domain, and an H2CH putative zinc-finger domain  [18, 36]. The THAP DNA-binding domain includes a C2CH consensus, three key residues (P, W, and F), and a C-terminal AVPTIF box [37]. These features were identified in the prospective *XKol*-Tpases (Fig. 1). The H2CH domain is highly conserved in the prospective *XKol*-Tpases. Thirty-five of the 38 *XKol*-Tpases in *X. tropicalis* and 22 of the 24 *XKol*-Tpases in *X. laevis* retained this motif (Table 2). Three THAP domain motifs were also conserved. The C2CH motif was conserved in 31 of 38 and 24 of 24 *XKol*-Tpases in *X. tropicalis* and *X. laevis*, respectively. The PWF motif was conserved in 33 of 38 and 24 of 24 *XKol*-Tpases in *X. tropicalis* and *X. laevis*, respectively. The AVPTIF motif was conserved only in 17 of 38 and 12 of 24 *XKol*-Tpases in *X. tropicalis* and *X. laevis*, respectively. However, most of the remaining *XKol*-Tpases had chemically similar residues at this position. When considering such residues as conserved, this motif was conserved in 27 of 38 and 23 of 24 *XKol*-Tpases in *X. tropicalis* and *X. laevis*, respectively. In contrast, the catalytic DDE motif was conserved in only 24 of 38 *XKol*-Tpases in *X. tropicalis* and seven of 24 *XKol*-Tpases in *X. laevis*. Some *XKol*-Tpases lost one or two of these motifs by deletion (Tr3, Tr25, Tr34, Tr35, Tr36, Tr37, and Tr38). These proteins may lack DNA-binding and/or catalytic activity. In summary, 17 *X. tropicalis* and seven *X. laevis XKol*-Tpases retained all the motifs characterizing *Kolobok* transposase.

### Truncated copies of the *XKol*-Tpase genes

Transposase genes may be retained in a host either by constant amplification of autonomous elements or by domestication in the host. Amplification of autonomous elements results in the accumulation of multiple intact and/or truncated copies in the genome. Therefore, they would have repetitive (multicopy) transposase genes. In contrast, if a copy of a transposase gene was domesticated by its host, it would be conserved by purifying selection. Its undomesticated relatives would not have been conserved by purifying selection and would have accumulated mutations and diverged from the domesticated copy. Over time, then, there would be no apparent homology between a domesticated transposase gene and its relatives, and the domesticated transposase genes would become nonrepetitive (single copies). Conversely, the nonrepetitiveness of a transposase-related gene may indicate that its host domesticated it long ago. *X. laevis* is an allotetraploid species retaining two of its progenitor’s subgenomes as L and S chromosomes, respectively [20]. Therefore, this species could have conserved both homeologous transposases if the genes had been domesticated before the segregation of the two progenitor species. In contrast, if either of the domesticated L or S genes had degraded because of functional redundancy, the remaining prospective gene would be accompanied by a closely related truncated sequence on its homeologous chromosome. If one of the tandemly duplicated paralogous transposase genes was degraded, the remaining gene would be accompanied by a closely related truncated sequence on its neighboring locus. In the analyses discussed below, we describe such genes as “semi-nonrepetitive.”

To find nonrepetitive or semi-nonrepetitive genes derived from transposases, we surveyed truncated (nonprospective) copies closely related to each *XKol*-Tpase CDS using a blastn (nucleotide query versus nucleotide database) search. If a query did not hit genomic sequences other than itself or other *XKol*-Tpase CDSs, it was considered nonrepetitive. The number of truncated copies and their loci are shown in Table 1 and (Additional file 7: Table S3), respectively. Eighteen of 38 *X. tropicalis XKol*-Tpase genes (47%) and 15 of 24 *X. laevis XKol*-Tpase genes (63%) were found to be nonrepetitive. Two *X. laevis XKol*-Tpase genes (Lv2 and Lv10) could be considered semi-nonrepetitive because their single truncated copies were located on the homeologous chromosome (Additional file 7: Table S3). One *X. laevis XKol*-Tpase gene (Lv16) and one *X. tropicalis XKol*-Tpase gene (Tr33) may also be semi-nonrepetitive, as their hit sequences were located in the neighborhood of the *XKol*-Tpase CDSs and may therefore have been amplified by tandem duplication rather than transposition. Lv11 and Lv17 had one and two truncated hits, respectively. However, one of their hit regions covered the entire length of a very short Scaffold105792 (216 bp). Therefore, this hit could be an artifact caused by an imperfect genome sequence assembly. Another hit sequence to Lv17 was located on the neighbor of its homeolog Lv16 and might be a truncated paralog of Lv16 (Additional file 7: Table S3 and Fig. 4). Lv11 and Lv17, then, may also be semi-nonrepetitive genes. It is possible that the host domesticated these nonrepetitive or semi-nonrepetitive genes long ago.

### Phylogenetic analyses of *XKol*-Tpases

If a transposase gene was domesticated before the divergence of *X. laevis* and *X. tropicalis* and is conserved in both species, then the *X. laevis* and *X. tropicalis* genes are orthologous and the two *X. laevis* genes are homeologous. They should be closely related in the molecular phylogenetic tree and located on homologous chromosome loci. The L and S genes derived from ancestral L and S species are located on the L and S chromosomes, respectively. Therefore, if both were conserved in *X. laevis*, then the branching pattern should be a triplet consisting of the *X. tropicalis* (semi-)nonrepetitive transposase and the two (semi-)nonrepetitive transposases on the *X. laevis* L and S chromosomes (triplet-branching). Alternatively, if either the L or S gene was lost or degraded in *X. laevis*, the branching pattern should consist of a pair of (semi-)nonrepetitive transposases from *X. tropicalis* and *X. laevis* (doublet-branching).

**Fig. 2:**
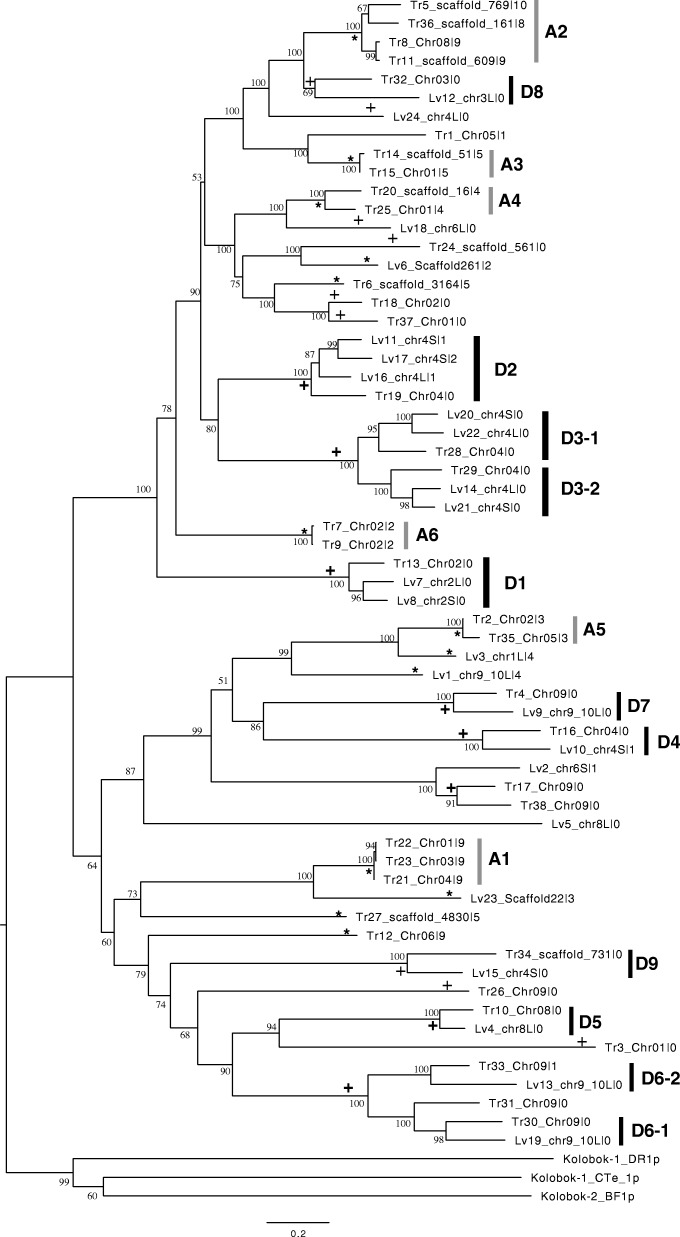
Molecular phylogeny of prospective *XKol* transposases. Molecular phylogenetic trees of *XKol*-Tpase amino acid sequences inferred using the neighbor-joining method. Each operational taxonomic unit is represented by the names of the CDS and a chromosome or scaffold on which the CDS is located. The number of blastn hits to truncated transposase sequences are shown as numerals following the vertical bar. The percentage of replicate trees in which the associated taxa clustered together in the bootstrap test (500 replicates) are shown next to the branches. The symbol “+” on the branches indicates domestication events. Asterisks indicate transpositional amplification events. Subfamilies of putative domesticated transposase orthologs are grouped by black bars and named D1–D9. CDS subfamilies that may have been duplicated by recent transposition and amplification are grouped by gray bars and designated A1–A6

**Fig. 3:**
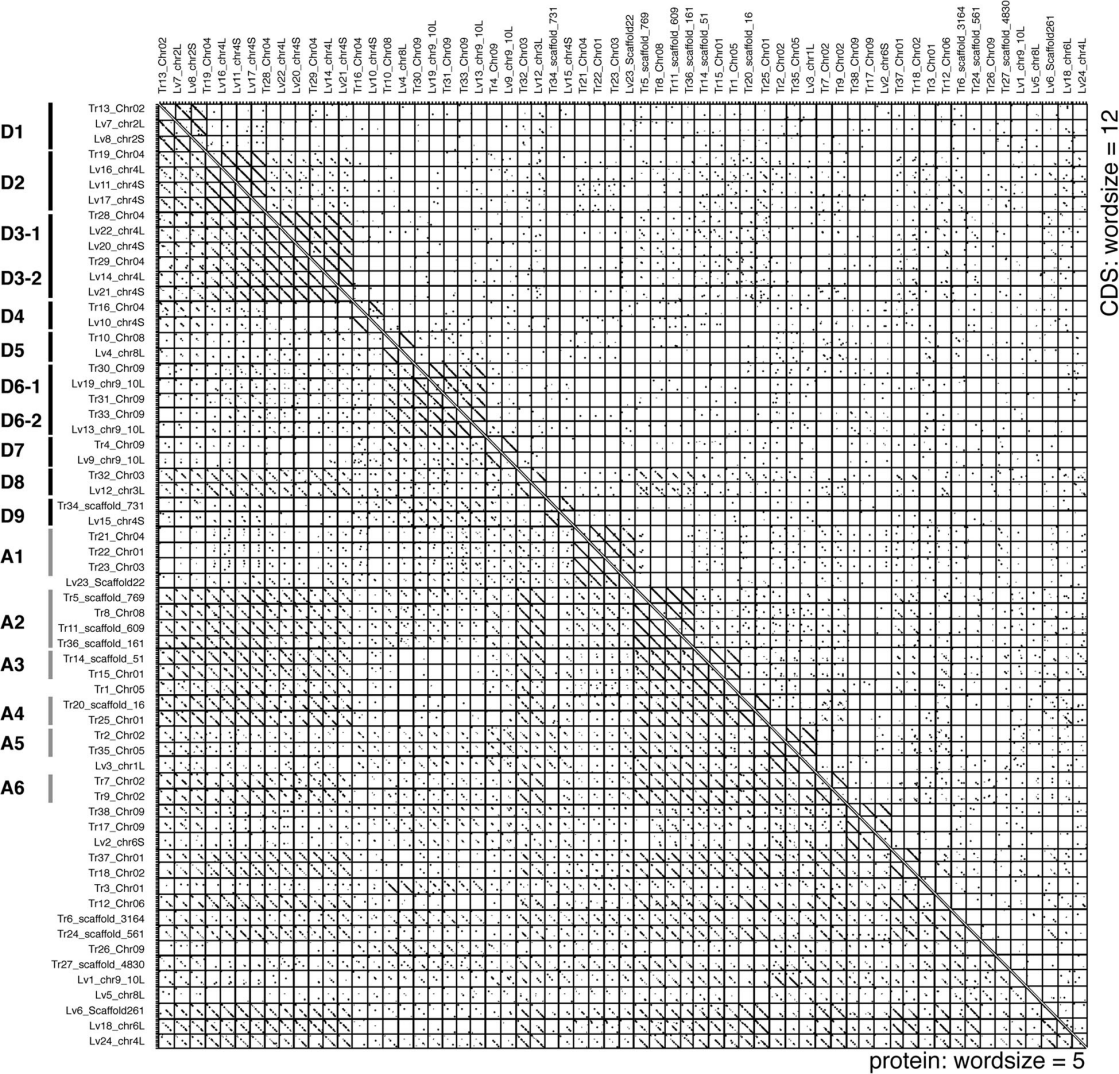
Dot plot analysis of *XKol*-Tpase gene CDSs. All-to-all comparisons of *XKol*-Tpase CDSs (upper right) and proteins (lower left) performed by dot plot analyses. D1–D9 and A1–A6 are grouped in the same way as in Fig. 2

**Fig. 4:**
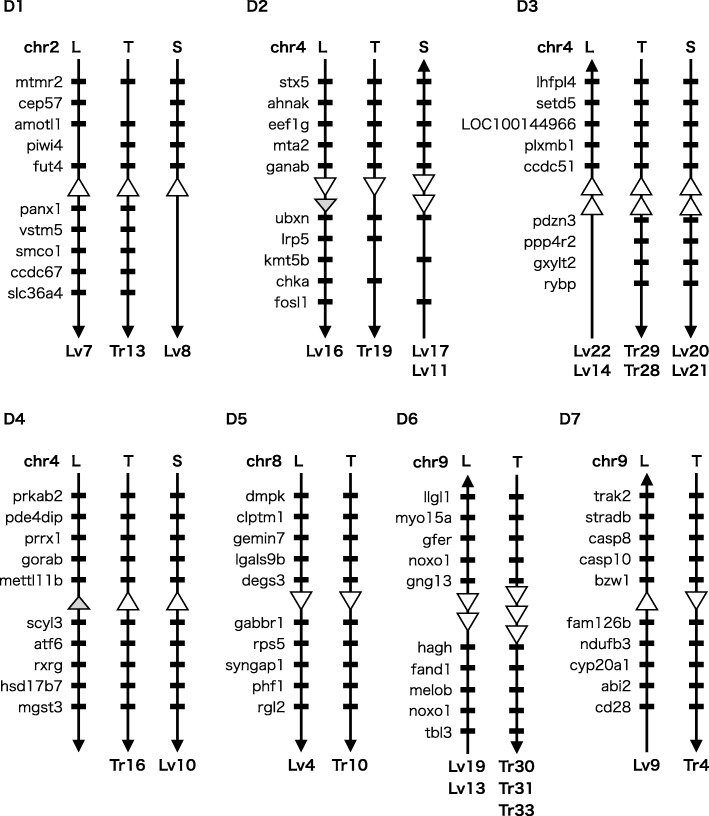
Synteny of *XKol*-Tpase genes. Conserved synteny around putative domesticated orthologs of subfamily D1–D7. *Xenopus laevis* homeologous chromosomes L and S and *Xenopus tropicalis* homologous chromosome (T) are indicated by arrows. The orientation of each arrow indicates the 5′ to 3′ direction. *XKol*-Tpase CDSs are represented by white triangles on the chromosomes, and their names are shown below the chromosomes. Gray triangles indicate truncated *XKol*-Tpase sequences. Gene models around the CDSs are represented by black bars

Figure 2 shows a molecular phylogenetic tree for the *XKol*-Tpases. A typical triplet-branching pattern can be seen for nonrepetitive Tr13, Lv7, and Lv8 (grouped as subfamily “D1” in Figs. 2 and 3), whose genes are located on the homologous chromosomes *X. tropicalis* Chr02, *X. laevis* chr2L, and chr2S, respectively. *X. tropicalis* Tr13 branched first, followed by the branching of *X. laevis* Lv7 and Lv8. This pattern corresponded to the order of divergence in *X. tropicalis* and two ancestral species of *X. laevis*. A dot plot analysis showed that their genes were significantly similar in amino acid and nucleotide sequences (Fig. 3). This relationship could also be seen for the other three (semi-)nonrepetitive *XKol*-Tpase triplets: Tr19/Lv16/Lv11-Lv17 (D2), Tr28/Lv22/Lv20 (D3–1), and Tr29/Lv14/Lv21 (D3–2). Lv11 and Lv17 genes were tandemly located within ~ 4.6 kb of each other on chr4S. Lv16 was found to be a semi-nonrepetitive gene accompanied by a closely related truncated sequence located in its neighborhood (Fig. 4). Therefore, these genes were thought to be tandemly duplicated before the segregation of the two ancestor species of *X. laevis*. The last two triplets Tr28/Lv22/Lv20 and Tr29/Lv14/Lv21 were closely related (Fig. 2). The tandemly duplicated gene pairs Tr28-Tr29, Lv14-Lv22, and Lv2-Lv20 were located within ~ 8 kb of each other on the homologous chromosomes *X. tropicalis* Chr04, *X. laevis* chr4L, and chr4S, respectively (Fig. 4). These paired genes were thought to be paralogs tandemly duplicated before the divergence of *X. laevis* and *X. tropicalis*. Six doublet pairs of closely related *X. tropicalis* and *X. laevis* (semi-)nonrepetitive genes were found to be located on the homologous chromosomes Tr16/Lv10 (chr4, D4), Tr10/Lv4 (chr8, D5), Tr30/Lv19 (chr9, D6–1), Tr33/Lv13 (chr9, D6–2), Tr4/Lv9 (chr9, D7), and Tr32/Lv12 (chr3, D8). One doublet pair, Tr34/Lv15 (D9), was also closely related; however, the chromosomal location of Tr34 was uncertain. These pairs may also be orthologs domesticated by the common ancestor of *X. laevis* and *X. tropicalis*. One of the *X. laevis* homeologs may have been lost or broken.

Some *X. tropicalis XKol*-Tpase genes encode highly similar proteins and are located on different chromosomes. These genes were probably recently amplified. For example, Tr21, Tr22, and Tr23 (grouped as subfamily “A1” in Figs. 2 and 3) were found to be located on Chr04, Chr01, and Chr03, respectively (Tables 1 and 3, Figs. 2 and 3). They also contained multiple truncated copies of *XKol*-Tpase genes on Chr01, Chr02, Chr04, Chr05, Chr06, and Chr09 (Additional file 7: Table S3). Another five *X. tropicalis XKol*-Tpase gene groups, i.e., Tr5-Tr8-Tr11-Tr36 (A2), Tr14-Tr15 (A3), Tr20-Tr25 (A4), Tr2-Tr35 (A5), and Tr7-Tr9 (A6), were also closely related to each other and had multiple truncated copies on different chromosomes (Fig. 2, Additional file 7: Table S3). Although *X. tropicalis* Tr6, Tr12, and Tr27 and *X. laevis* Lv1, Lv3, Lv6, and Lv23 did not have similar *XKol*-Tpase genes, they had truncated copies on various chromosomes (Additional file 7: Table S3), representing traces of their amplification and transposition. Similarities in nucleotide sequences between these *XKol*-Tpase genes and their truncated hits are shown by dot plots (Additional file 2: Figure S2).

**Table 3:** Relationship of transposases between *X. tropicalis* and *X. laevis*

Name	Closely related CDS	Subfamily	Synteny
A. Relationship between *X. tropicalis* and *X. laevis* transposases			
Tr1			
Tr2	Lv3(L)	A5	
Tr3			
Tr4	Lv9(L)	D7	+
Tr5		A2	
Tr6			
Tr7		A6	
Tr8		A2	
Tr9		A6	
Tr10	Lv4(L)	D5	+
Tr11		A2	
Tr12			
Tr13	Lv7(L), Lv8(S)	D1	+
Tr14		A3	
Tr15		A3	
Tr16	Lv10(S)	D4	+
Tr17	Lv2(S)		
Tr18			
Tr19	Lv16(L), Lv11(S), Lv17(S)	D2	+
Tr20		A4	
Tr21	Lv23	A1	
Tr22	Lv23	A1	
Tr23	Lv23	A1	
Tr24			
Tr25		A4	
Tr26			
Tr27			
Tr28	Lv22(L), Lv20(S)	D3–1	+
Tr29	Lv14(L), Lv21(S)	D3–2	+
Tr30	Lv19(L)	D6–1	+
Tr31		D6–1	+
Tr32	Lv12(L)	D8	
Tr33	Lv13(L)	D6–2	+
Tr34	Lv15(L)	D9	
Tr35	Lv3(L)	A5	
Tr36		A2	
Tr37			
Tr38	Lv2(S)		
B. Relationship between *X. laevis* and *X. tropicalis* transposases			
Lv1			
Lv2	Tr17*, Tr38		
Lv3	Tr2*, Tr35		
Lv4	Tr10	D5	+
Lv5			
Lv6			
Lv7	Tr13	D1	+
Lv8	Tr13	D1	+
Lv9	Tr4	D7	+
Lv10	Tr16	D4	+
Lv11	Tr19	D2	+
Lv12	Tr32	D8	
Lv13	Tr33	D6–2	+
Lv14	Tr29	D3–2	+
Lv15	Tr34	D9	
Lv16	Tr19	D2	+
Lv17	Tr19	D2	+
Lv18			
Lv19	Tr30	D6–1	+
Lv20	Tr28	D3–1	+
Lv21	Tr29	D3–2	+
Lv22	Tr28	D3–1	+
Lv23	Tr22*, Tr21, Tr23		
Lv24			

(A) Relationship between *X. tropicalis* and *X. laevis* transposases and (B) relationship between *X. laevis* and *X. tropicalis* transposases. Asterisks in the “closely related CDS” column indicate CDSs used in the analysis of differences in nucleotides and amino acids between *X. laevis* and *X. tropicalis* (Table 4B)

### Evidence of *XKol*-Tpase gene domestication

The triplet or doublet *XKol*-Tpase genes described above were thought to have been domesticated by the common ancestor of *Xenopus*. We analyzed the neighbors of these putative domesticated *XKol*-Tpases to assess whether the genes in each triplet or doublet were located on homologous loci. All four triplets (D1, D2, D3–1, and D3–2) and five doublets (D4, D5, D6–1, D6–2, and D7) in the *X. tropicalis* and *X. laevis* genes conserved synteny with the neighbors (Fig. 4). In contrast, we did not find synteny for the doublet pairs D8 and D9 (data not shown).

We calculated the dN/dS ratio between putative orthologous or similar *X. tropicalis* and *X. laevis XKol*-Tpase genes to determine whether they were affected by natural selection (Table 4B). All dN/dS ratios were less than 1.0; therefore, these genes were probably conserved by purifying selection.

**Table 4:** Differences in transposase CDSs and amino acids, and presumptive status

Name	Nucleotide substitution	Nucleotide gap	Amino acid substitution	dN/dS	Presumptive status
A. Differences between Nigerian and Asashima lines, and presumptive status					
Tr1	20	6	10	0.396	active?
Tr2	11	24	frame-shift	–	active?
Tr3	5	0	3	1.006	domestication?
Tr4	11	12	7	0.501	ancient domestication
Tr5					currently active
Tr6	1	0	1	dS = 0	currently active
Tr7	9	0	7	0.696	currently active
Tr8	26	0	21	1.057	currently active
Tr9					currently active
Tr10	5	0	1	0.084	ancient domestication
Tr11					currently active
Tr12	205	991	frame-shift	–	active?
Tr13	26	0	8	0.173	ancient domestication
Tr14					currently active
Tr15	0	3	0	dS = 0	currently active
Tr16	3	0	2	0.527	ancient domestication
Tr17	24	0	13	0.434	domestication?
Tr18	15	0	9	0.504	domestication?
Tr19	4	30	4	dS = 0	ancient domestication
Tr20	53	0	29	0.403	currently active
Tr21					currently active
Tr22	0	0	0	dS = 0	currently active
Tr23					currently active
Tr24	57	6	23	0.28	domestication?
Tr25					active?
Tr26	15	0	3	0.058	domestication?
Tr27	55	0	19	0.118	active?
Tr28	9	0	6	0.629	ancient domestication
Tr29	27	0	12	0.312	ancient domestication
Tr30	5	0	2	0.328	ancient domestication
Tr31	21	3	11	0.359	ancient domestication
Tr32	7	0	6	1.444	domestication?
Tr33	30	0	8	0.14	ancient domestication
Tr34	23	0	9	0.192	domestication?
Tr35					active?
Tr36					active?
Tr37					domestication?
Tr38	12	0	5	0.196	domestication?
B. Differences between *X. laevis* and *X. tropicalis*, and presumptive status					
Lv1					currently active
Lv2	466	237	202	0.285	domestication?
Lv3	472	63	210	0.194	currently active
Lv4	263	6	110	0.257	ancient domestication
Lv5					domestication?
Lv6					currently active
Lv7	289	51	140	0.391	ancient domestication
Lv8	293	57	141	0.301	ancient domestication
Lv9	303	42	154	0.341	ancient domestication
Lv10	302	36	161	0.463	ancient domestication
Lv11	335	42	172	0.358	ancient domestication
Lv12	546	252	213	0.100	domestication?
Lv13	359	198	164	0.365	ancient domestication
Lv14	305	36	155	0.413	ancient domestication
Lv15	411	393	157	0.195	domestication?
Lv16	305	36	162	0.349	ancient domestication
Lv17	331	54	162	0.336	ancient domestication
Lv18					domestication?
Lv19	335	69	156	0.328	ancient domestication
Lv20	323	66	165	0.497	ancient domestication
Lv21	312	33	166	0.472	ancient domestication
Lv22	356	72	193	0.521	ancient domestication
Lv23	591	294	240	0.153	active?
Lv24					domestication?

(A) Differences between Nigerian and Asashima lines and (B) differences between *X. laevis* and *X. tropicalis*. *X. tropicalis* CDSs used in the analysis of differences with those of *X. laevis* are indicated by asterisks in Table 3B. The last column indicates the presumptive status of each *XKol* transposase

We investigated the evolutionary conservation of the CDSs compared to their flanking 2000 bp sequences for subfamilies D1–D9 (Additional file 3: Figure S3). The dot plots showed that the CDS regions were more conserved than their flanking regions. This result also supported the hypothesis that the *XKol*-Tpase proteins have a function conserved by natural selection.

We amplified and cloned the CDSs of the putative domesticated *XKol*-Tpase genes from the distinct Nigerian and Asashima lineages of *X. tropicalis.* There were nucleotide substitutions and indels between the clones of the two lines. Nevertheless, the ORFs of the CDSs were not destroyed by nonsense or frameshift mutations (Table 4A and Additional file 4: Figure S4). For the *X. tropicalis* triplet and doublet genes, the dN/dS ratios between the Nigerian and Asashima lines were less than 1.0 in all cases except for Tr19, wherein all four substitutions were nonsynonymous. We also cloned and compared CDSs from nine other nonrepetitive genes (Tr3, Tr17, Tr18, Tr24, Tr26, Tr31, Tr32, Tr34, and Tr38) in the Nigerian and Asashima lines (Table 4A and Additional file 4: Figure S4). Once again, neither substitutions nor indel mutations destroyed the ORFs. The dN/dS ratios were < 1.0 for seven genes, approximately 1.0 for Tr3, and > 1.0 for Tr32. These results indicated that most nonrepetitive or semi-nonrepetitive *XKol*-Tpase genes were domesticated by the host and conserved by purifying selection.

### Conservation of repetitive and recently active *XKol*-Tpase genes

In general, active transposons were not conserved by purifying selection because they were untamed and potentially harmful to the host. *X. tropicalis* Tr21-Tr22-Tr23 (A1), Tr5-Tr8-Tr11-Tr36 (A2), Tr14-Tr15 (A3), Tr20-Tr25 (A4), Tr2-Tr35 (A5), Tr7-Tr9 (A6), Tr6, Tr12, and Tr27 had multiple intact and/or truncated copies. Dot plot analyses revealed that all intact genes and some truncated copies were highly similar (Additional file 2: Figure S2), suggesting that they were recently amplified. Therefore, they were either currently active or were active until recently.

We cloned and compared the CDSs of the repetitive *XKol*-Tpases in the Nigerian and Asashima lines (Table 4A). The ORFs of Tr22, Tr8, Tr15, Tr20, Tr6, and Tr27 were conserved and not destroyed by nonsense or frameshift mutations. The clones of Tr22 and Tr15 from the Asashima line were identical to those from the Nigerian line. For Tr2 and Tr12, frameshift mutations destroyed the ORFs of the clones from the Asashima line. Tr12 from the Asashima line was degraded by many mutations.

We searched the upstream and downstream flanking sequences of the CDSs of the repetitive *XKol*-Tpases for left and right TIRs and found both left and right ones for ten CDSs (Table 5). All of them had AG terminal sequences, and all except for the right TIR of Tr11 were flanked by an intact TTAA target sequence. These are probably full-length autonomous *XKol* copies that still have transposition activity.

**Table 5:** Full-length *X. tropicalis XKol* sequences

Subfamily	CDS	CDS locus	Full-length *Xkol* sequence	Length	TIR left	TIR right	TSD left	TSD right
A1	Tr21	Chr04:107657217..107659511(+)	Chr04:107650836..107663091(+)	12,216	AGCGATTCTGACATGG	AGTGATACTGACAGTA	TTAA	TTAA
A1	Tr22	Chr01:168455606..168457900(−)	Chr01:168451382..168460584(−)	9163	AGCGATTCTGACATGG	AGTGATACTGACAGTA	TTAA	TTAA
A1	Tr23	Chr03:106168762..106171056(−)	Chr03:106165634..106173458(−)	7785	AGCGATTCTGACATGG	AGTGATACTGACAGTA	TTAA	TTAA
A2	Tr8	Chr08:54548260..54550611(+)	Chr08:54546557..54553359(+)	6763	AGGAGAAGGAAAGGCT	AGGAAATGGCAAGCCA	TTAA	TTAA
A2	Tr11	scaffold_609:18293..20635(−)	scaffold_609..15284:22344(−)	7021	AGGAGAAGGAAAGGCT	AGGAAATGGCAAGCCA	TTAA	ATAA
A3	Tr14	scaffold_51:231432..233759(−)	scaffold_51..227047:240993(−)	13,907	AGGACATGTCAACCCC	AGGACGTGTCAACCCT	TTAA	TTAA
A3	Tr15	Chr01:83498100..83500424(+)	Chr01:83495893..83504753(+)	8821	AGGACATGTCAACCCC	AGGACGTGTCAACCAT	TTAA	TTAA
A4	Tr25	Chr01:107512548..107514821(+)	Chr01:107510216..107521330(+)	11,075	AGGACAAGGAAAGCTT	AGGAAAATGAAAGTCA	TTAA	TTAA
A5	Tr2	Chr02:100830975..100833458(+)	Chr02:100828018..100838339(+)	10,282	AGGGGAACTATCATGA	AGGGGATCTATCATGA	TTAA	TTAA
A6	Tr9	Chr02:55486066..55488417(+)	Chr02:55483392..55492644(+)	9213	AGAGCAAGTAAAGTCG	AGAGCAAGGCAAGCTT	TTAA	TTAA

The position of full-length transposases, their left and right terminal sequences, and conservation of target sequences duplicated by the insertion of a transposon

## Discussion

### Multiple massive domestication of *Kolobok* transposases

In the present study, we surveyed the *X. tropicalis* and *X. laevis* genomes and found 38 and 24 prospective *Kolobok* transposase genes, respectively, whose CDSs encoded proteins of more than 600 amino acids in length. Subfamily A2 (Tr8/Tr11) and A6 (Tr7/Tr9) resembled previously reported *X. tropicalis* transposases of Kolobok-1_XT and Kolobok-2_XT, respectively [18], but others were novel genes. It is possible that some of these were not functional, because they were encoded by shorter CDSs that lacked certain lengths of conserved regions. For example, Tr36 lacked the C-terminal region, and Tr34, Tr35, Tr37, and Tr38 lacked the N-terminal regions conserved in the other *XKol*-Tpases. In contrast, most *XKol*-Tpases retained the DNA-binding motifs of *Kolobok* transposases, such as the C2CH domain [36] and the THAP domain [37]. Therefore, at the very least, they partially retained their molecular functions.

Among the 62 *XKol*-Tpases, 24 (39%) were grouped into seven triplet or doublet clusters of *X. tropicalis* and *X. laevis XKol*-Tpases on the molecular phylogenetic tree (subfamilies D1–D7). Their genes were located on homologous chromosome loci. All the dN/dS ratios between the *X. tropicalis* and *X. laevis* CDSs within each subfamily were less than 1.0. These results indicated that they were derived from an ancestral transposase gene domesticated before the divergence of the *Xenopus* genus. Among them, only five (21%) of the *XKol*-Tpases (Tr10, Tr13, Lv4, Lv7, and Lv8 belonging to D1 or D5) retained all conserved *Kolobok* transposase motifs. The catalytic DDE motif was conserved only in these five proteins, whereas the other 19 lost this motif. In contrast, the C2CH and PWP DNA-binding motifs were conserved in all 24 proteins. The H2CH and AVPTIF domains, the latter of which was defined as conserved if each of its amino acids was substituted by similar ones, were highly conserved. Only four proteins in subfamily D6–2 or D7 (Tr4, Tr33, Lv9, and Lv13) lost the former domain, and two proteins in subfamily D7 (Tr4 and Lv9) lost the latter domain. These results suggest that most of the domesticated *XKol*-Tpases retained their DNA-binding activity, but lost the catalytic activity necessary for transposition. These “transposases” may suppress transposons by competitively binding the target sequences of active transposases.

Fifteen other nonrepetitive *XKol*-Tpase genes were also found. Among them, only five proteins conserved the DDE motifs, whereas the H2CH-, C2CH-, PWF-, and AVPTIF motifs were conserved in 14, 11, 12, and 11 of the 15 proteins, respectively. Therefore, most of these *XKol*-Tpases may also have retained their DNA-binding activity but lost the ability to catalyze transposition. They may have been domesticated after the divergence of the two *Xenopus* species. Alternatively, they may constitute the remainder of ancestral domesticated transposase genes, and their orthologs may have been lost in other species. According to this study, 39 prospective *XKol*-Tpase genes (63%) were apparently domesticated.

The best tblastn hits to *XKol*-Tpase in the GenBank database (excluding hits to the *Xenopus* sequence) were invertebrate sequences (data not shown). These included the cnidarian *Acropora digitifera* uncharacterized LOC107332277 mRNA (XM_015897006) and the *Exaiptasia pallida* uncharacterized LOC110237754 mRNA (XM_021043370). The lack of domesticated genes encoding *Kolobok* transposases orthologous to *XKol*-Tpases in other vertebrates suggests that domestication occurred after *Xenopus* diverged from other model animals, including mammals and birds. Molecular phylogeny suggested that independent domestication events occurred several times. If the *XKol*-Tpases located on nonhomologous loci had been domesticated independently, domestication may have occurred at least 16 times in *Xenopus*. This situation was the opposite of that reported for the *Xenopus piggyBac* superfamily *TxpB* [9]. The *TxpB* family includes only one domesticated subfamily, *Kobuta*, and it was domesticated only once in the *Xenopus* ancestor.

### Currently active *Kolobok* transposases and the T2-MITE family

Among the 62 *XKol*-Tpase genes, 23 (37%) were repetitive, having interspersed multiple intact and/or truncated copies in the genome. Of these, 15 (65%) retained all conserved motifs of the *Kolobok* superfamily transposase. We regard them as currently active transposases. The eight other repetitive *XKol*-Tpases had lost at least one conserved motif. We could not determine whether they were currently active.

Some “currently active” *X. tropicalis XKol*-Tpases were conserved between the Nigerian and Asashima lines. The CDSs of Tr6, Tr15, and Tr22 cloned from the Asashima line were nearly or exactly identical to those from the Nigerian line*.* Considering the distance between these two lines [35], this similarity may be explained by recent introgression or horizontal transfer of these genes from the original population of the Asashima line to that of the Nigerian line or vice versa. The CDSs of Tr7, Tr8, and Tr20 presented with 9, 26, and 53 nucleotide substitutions between the two lineages, respectively. However, their ORFs were conserved despite the large number of mutations. The “currently active” *X. laevis XKol*-Tpase Lv3 and the closely related *X. tropicalis* Tr2 had 472 nucleotide substitutions and 63 nucleotide gaps, but their ORFs were also conserved (Table 4). This phenomenon may be explained by purifying selection because the dN/dS ratio between them was 0.194. These results suggested that some *XKol* family transposons were currently active in the host and had also been domesticated by it. Domesticated *XKol*-Tpases belonging to the D1 and D5 subfamilies also conserved all *Kolobok* transposase motifs, including the catalytic DDE domain. Therefore, domestication and catalytic transposition activity may not be mutually exclusive in the *XKol* family. This apparent contradiction may be resolved by considering that the transposition catalysis of *XKol*-Tpases has been beneficial to the host.

*Kolobok* transposases probably transpose the T2-MITE family [38], which is predominant in *Xenopus* [13, 14, 16]. Certain subfamilies (T2-A1 and T2-C) may be “long-lived”; that is, they have retained transposition activity in the *X. tropicalis* lineage since before the divergence of *X. laevis* and *X. tropicalis* [19, 21]. Our recent analysis of *X. laevis* revealed that some T2-MITE subfamilies have multiple identical copies, suggesting that they have recently been active (unpublished data). The long conservation activity of T2-MITE subfamilies in both species suggested that they may have contributed to host fitness through transposition. Recently, we reported that the T2-C subfamily tended to be located near the upstream regions of genes in *X. tropicalis*. The expression patterns of genes with upstream insertions were strongly correlated [22]. This distribution may indicate its function to be recruited by the host. For example, T2-C may include a *cis*-regulatory element. The insertion of a certain element into an upstream region may have been beneficial to the host; hence, it was conserved by natural selection. Recent studies have revealed that TE-derived sequences were recruited by hosts as *cis*-regulatory elements. TE transposition and amplification are important drivers of the evolution of the gene regulatory network [39]. The conservation of the *Kolobok* superfamily may be partially explained by pressure from the host to continue to activate the transposition of some T2-MITE subfamilies and to rewire their gene regulatory network.

## Conclusions

Our results indicated that multiple massive *XKol*-Tpase domestication events occurred during *Xenopus* evolution and that *XKol* family transposons have complex, mutually beneficial relationships with their hosts. Long conservation of transposition activity and/or conserved catalytic and DNA-binding domains in certain *XKol*-Tpases suggest that they may benefit the host by catalyzing the transposition of long-lived T2-MITE subfamilies.

## Additional files

Additional file 1: **Figure S1.** Full-length multiple alignment of transposases predicted from prospective CDSs of *XKol*-Tpase genes with three outgroup transposases. Certain proteins predicted from the longest ORFs had excess N-terminal amino acids compared with other proteins. We trimmed the corresponding 5′ regions from these prospective CDSs to align their start methionine codons with those in the others. This alignment was used in molecular phylogeny (Fig. 1). (PDF 50 kb)

Additional file 2: **Figure S2.** Repetitive *XKol*-Tpase CDSs (highlighted in red) were compared with their truncated copies by dot plot analyses (word size = 12). Flanking upstream and downstream 1000 bp CDSs and truncated copy sequences are also included for comparison. (PDF 717 kb)

Additional file 3: **Figure S3.** *XKol*-Tpase CDSs and flanking upstream and downstream 2000 bp sequences of subfamily D1–D9 were compared by dot plot analyses (word size = 10). (PDF 319 kb)

Additional file 4: **Figure S4.** Multiple alignment of prospective transposase CDSs from the *X. tropicalis* and *X. laevis* genome databases and from CDSs cloned from *X. tropicalis* Nigerian and Asashima lineages. (PDF 167 kb)

Additional file 5: **Table S1.** Name for the *Kolobok* superfamily transposase query used in the tblastn search. Name for the source transposon in Repbase and protein length are shown. (XLSX 31 kb)

Additional file 6: **Table S2.** Primer sequences used in PCR to clone *XKol*-Tpase CDSs from the *X. tropicalis* Nigerian and Asashima lineages. (XLSX 33 kb)

Additional file 7: **Table S3.** Results of blastn searches to identify truncated copies of *XKol*-Tpase genes. Prospective *XKol*-Tpase CDS used as a query, locus of the CDS, hit locus of the blastn search (e-value <1e-100), and its length (bp) are shown. (XLSX 30 kb)
