# Supplementary material for: Multiple massive domestication and recent amplification of Kolobok superfamily transposons in the clawed frog Xenopus
Source: Zoological Lett. 2018 Jun 16;4:17. doi: 10.1186/s40851-018-0100-4 (PMC6004289; doi:10.1186/s40851-018-0100-4)
Supplement: Supplementary file 1 — Figure S1. Full-length multiple alignment of transposases predicted from prospective CDSs of XKol-Tpase genes with three outgroup transposases. Certain proteins predicted from the longest ORFs had excess N-terminal amino acids compared with other proteins. We trimmed the corresponding 5′ regions from these prospective CDSs to align their start methionine codons with those in the others. This alignment was used in molecular phylogeny (Fig. 1). (PDF 50 kb) [file 40851_2018_100_MOESM1_ESM.pdf]

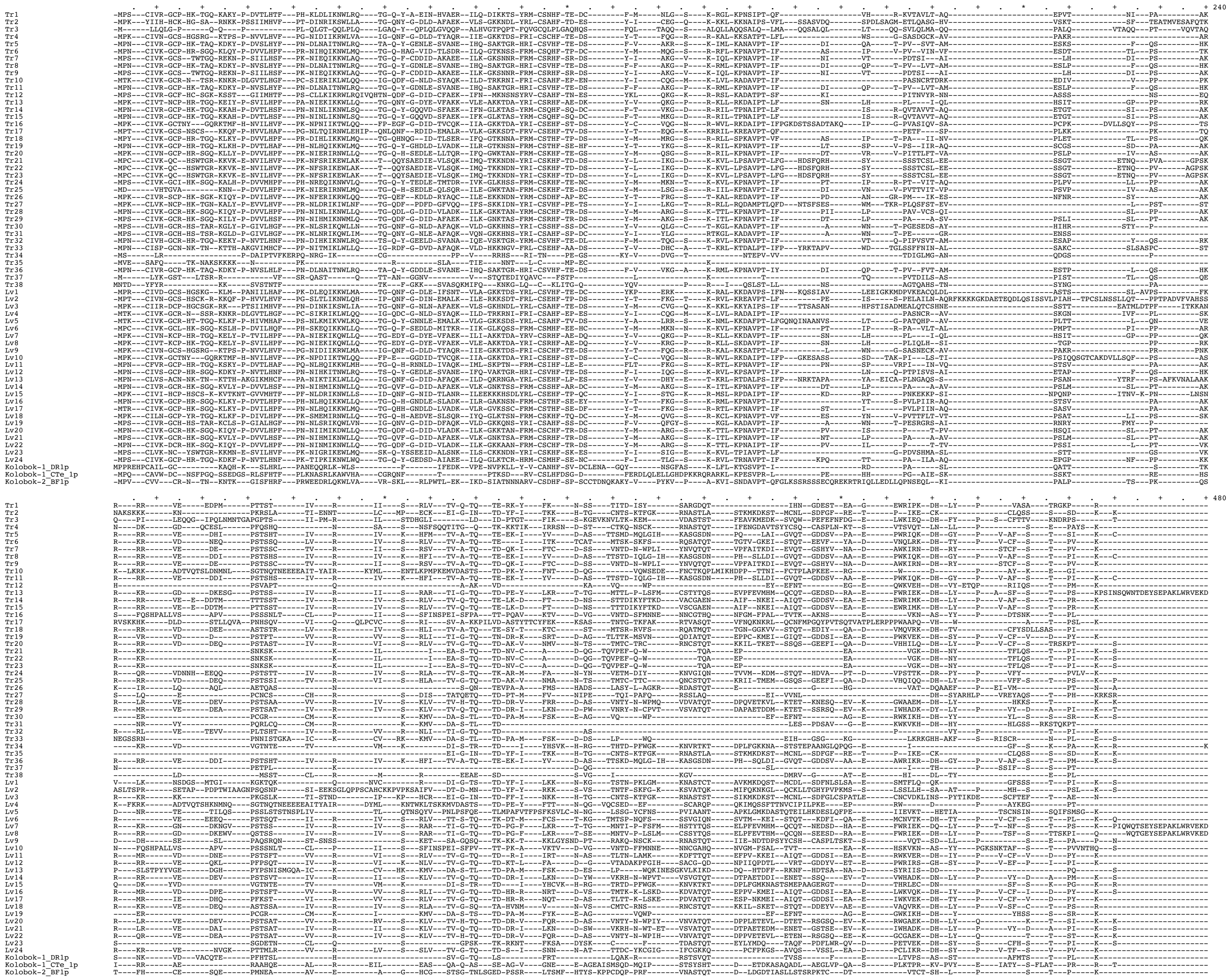

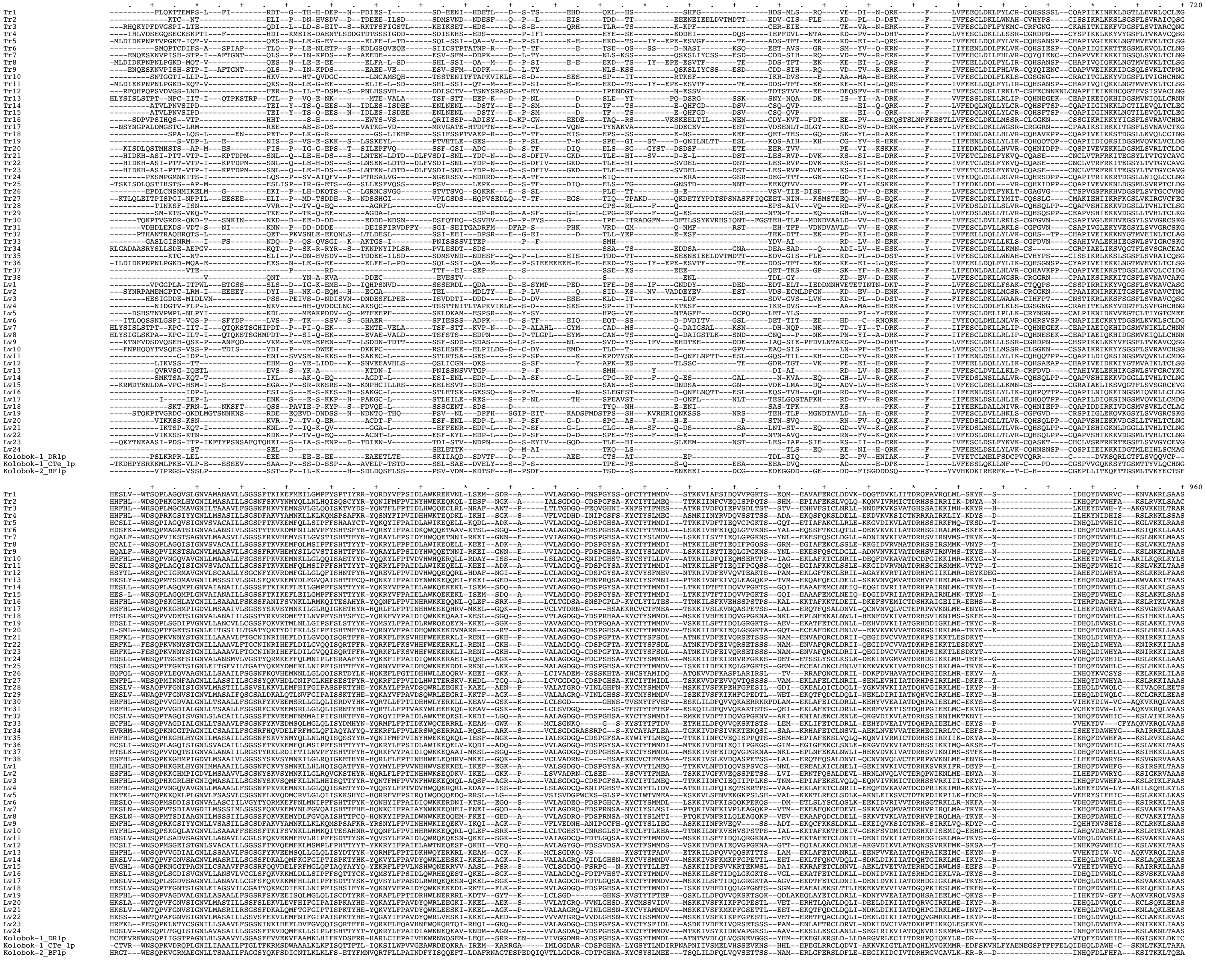
720
960
Tr1
Tr2
Tr3
Tr4
Tr5
Tr6
Tr7
Tr8
Tr9
Tr10
Tr11
Tr12
Tr13
Tr14
Tr15
Tr16
Tr17
Tr18
Tr19
Tr20
Tr21
Tr22
Tr23
Tr24
Tr25
Tr26
Tr27
Tr28
Tr29
Tr30
Tr31
Tr32
Tr33
Tr34
Tr35
Tr36
Tr37
Tr38
Lv1
Lv2
Lv3
Lv4
Lv5
Lv6
Lv7
Lv8
Lv9
Lv10
Lv11
Lv12
Lv13
Lv14
Lv15
Lv16
Lv17
Lv18
Lv19
Lv20
Lv21
Lv22
Lv23
Lv24
Kolobok-1_DR1p
Kolobok-1_CTe_1p
Kolobok-2_BF1p

```
                        .    +    .    +    .    +    .    *    .    +    .    +    .    +    .    +    .    +    .    +    .    +    .    +    .    +    .    +    .    *    .    +    .    +    .    +    .    +    .    +    .    +    .    +    .    + 1200
Tr1                 KTRNGKAIAPWVEAITKHL-W-WCSQTCYRNVDVLIDKWKSVLYHISNRHTF-PAL-KHYNKCHH-KTWTAA-QSECKR-WIPPGHPAHTILTKIITDPKLIKDISQIGKFC-HTE-ELENFHSK-AFKFR----PKGMCLGVDSMH-ARTM----LAVLSHNRNTG-R-WKATNHDPRKSRLEVRQ-EG-IRTTYPKRK-EDS-VA----KPIYEQVTDPHIFDIMSDSAR-IA-----
Tr2                 KKKKFSEIKEWISPTINHL-W-WCARTCNGNPDLLREKWLSLLYHVTNVHQW-QTG-HLYHECAH-SALAPH-LDHKRK-WMQDESTVFEQFKDIITSRKILDDLAHLSHFC-HTG-ELEVYHSN-LLKYR----SKRHHYFIDGMI-ARSQ----LGALDHNFNVQ-R-EQARVCTTGPECDSVGS-LR-HKLEFTKSK-KDW-VV----KPIYTRPSNSFLFELLKDIIR-LA-----
Tr3                 KGKTLVKLINWLPAISTHL-W-WSCITSQRNTVMFQERWQSLLHHVTNQHQW-KNA-ETFHGCSH-GKLTSM-QHRQCS-WLKKGTPAFHTLRDVVMNPQITKDFHCMSRFC-CTE-EMDVYKSF-VTKYQ----TNRFHLNINAME-ARTK----LAALAYNANVH-RYP---IHTKYMGRGSIGR-AR-QKHMFPKCR-KRR-VA----RSVYTDTFTEHTLSMMADVLK-IY-----
Tr4                 KQKNCSQISQWIKPAVSHL-L-WASQTCDGSADLLKKKWQSLLNHVTKVHKW-ETS-QQFHGCTQ-ENLSA--RCK-RK-WMKCGSTAFNRFKEIVMSAQLIRDLNHLSEFC-HTD-ELKLYHSN-LLKYR----PRTLH-SMDDVV-VRTQ----LAALDHNYNVH-R-GKAQWNDTDVQDPSAG-----LQ---PKVR-----------KCVFDAASKAFLLVILKDIVR-YV-----
Tr5                 KRRKCKDIAHWIGPITKHL-W-WCSQTCDRNVENLLDKWRSLLYHIANKHTF-PNL-KTYKKCQH-RKMTAG-EIREKK-WITPSHPAYCTLVAILTDKLLIRDICQTEKFC-HTG-DLENFRSK-VLKYW----PERIDFTLDSMY-ARTM----LAALSHNKNVN-R-PQASARCSKISELPVGE-KT-FQIVFPKHN-KEW-VA----KPIIEEVGDWRLFDIISDAGK-IL-----
Tr6                 KKRKCRDIAHWINAITNHL-W-WSSQTCKQNVNVLLAKWKSVLFHVANKHTF-KSL-KYYKKCQH-RRLTAA-QKKDAR-WIACNHPAHSTLKEIINDRFLLRDIAQTEKFC-HTG-DLENFHSK-VLKYK----PKRISFKMDAMI-ARTS----LGALSHNRNVH-R-HQAVVKRATKTSLAFGE-KR-FKIVFPKMK-KDW-IA----KPVYEDVIDEHLLDITSDSIK-IL-----
Tr7                 KKKNCGDISKWISSITNHL-W-WCSQTCDQNVEVLLSKWKSLMFHISNVHTF-PKL-EHYKKCIH-PKISRK-EVRKAP-WIKPGDPAHATLASIVNSKYLLKDMHHIEKFC-HTG-DLENFHSK-VLKYR----LKRIAFKIDAMH-ARTV----LAILSHNRNVN-R-PQATVKCAKKSTLPVGS-KR-YKFVFPKHK-KNW-VT----RAIYEEVVDDHLLDIAGDSLK-IL-----
Tr8                 KKRKCKDIAHWIGPITNHL-W-WCSQTCDHNVDNLLDKWRSVLYHIANKHTF-RNL-KTYKKCEH-KKIRGA-EMKDKK-WITPSHPAYSTLVAILNSKQLMKDITQIEKFC-HTG-DLENYHSK-VLKYR----PKRIAFKLDSMY-ARTM----LAALSHNKNVD-R-PQATVHCSKKSEFPVGE-KR-FKIVFPKHK-KEW-VA----KPIFEEISDVHLFDIISDAGK-IV-----
Tr9                 KKKNCGDISKWISSITNHL-W-WCSQTCDQNVEVLLSKWKSLMFHISNVHTF-PKL-EHYKKCIH-PKISRK-EVRKAP-WIKPGDPAHATLASIVNSKYLLKDMHHIEKFC-HTG-DLENFHSK-VLKYR----LKRIAFKIDAMH-ARTV----LAILSHNRNVN-R-PQATVKCAKKSTLPVGS-KR-YKFVFPKHK-KNW-VT----RAIYEEVVDDHLLDIAGDSLK-IL-----
Tr10                KRKACPELEKWIPTITSHL-W-WAANTSHGNTDLLLERWQSLLPHLVNQHKW-KGM-KFSSGCTH-RPLAAR-EQKSCP-WLKKGTLAFYSLRQVIIHPHVSNDLLHLSQIS-HAE-EVEMYHRF-VLKYR----PKCMHLKMDAIE-AMTK----LAVLAYNANIH-R-QKLRCFSVKKGKGVFGTVTH-VR----KCR-----LA----KPLYHEASSEHVIPMMTDVIK-IS-----
Tr11                KKRKYKDIAHWIGPITNHL-W-WCSQTCDHNVDNLLDKWRSVLYHIANKHTF-RNL-KTYKKCEH-KKIRAA-EMKDKK-WITPSHPAYSTLVAILNSKQLMKDITQIEKFC-HTG-DLENYHSK-VLKYR----PKRIAFKLDSMY-ARTM----LAALSHNKNVD-R-PQATVHCSKKSEFPVGE-KR-FKIVFPKHK-KEW-VA----KPIFEEISDVHLFDIISDAGK-IV-----
Tr12                KKKNCGDIAEWIPAITNHL-W-WSSSKCEGDLRMLRERWQSVLMHISDQHEWDHGI--MYHACTH-KKYTDE-ERSQRP-WIEKDSQAYLSLAEVVLSKNMTKDLEHMAHFR-HTG-ALEVYHSF-SLKYR----PKRIHFKMDGME-ARTK----LAALAHNANVH-R-ERSRVRCAGEGRDPVGS-LR-HKLVFPKFK-KKW-VT----KVLYEPTTNEHIMPMLVDVLK-LL-----
Tr13                KKPKCQGLTWWITPIQNHL-W-WCVQTSRQNPDVLIDKWLSVLFHIADVHNF-PRL-KHYKRCIH-TTLTPQ-KRKEIK-WIKPSHPAHRALEKIVSDPALLKDLQHITQIS-HTP-QFEVYRSK-SLKYR----SKNYSYKIESAY-ARTI----LAILSNNHSVG-K-ERTALRSPSKSDLPVVE-KC-CKTVFSKQE-KDW-VA----TPIYKQGVDSHLFKLMNDCLS-IM-----
Tr14                KKKNGKDIGPWIGAITNHL-W-WCSQTCDHDVNLLIDKWKSLLFHIANKHTF-RSL-KNYTKCDH-KRLPAS-QSEDKN-WITTSHPAHTVLTQIITDPKLIKDISKVEKFC-HTG-ELENFHSK-VLKFR----PKRIYFGMDSMH-ARTM----LAILSHNKNIG-R-PQATIQDATKSSLELGQ-KR-FKIVYPKQK-KDW-VA----KPIYEKVTDSHLFDIMLDSAR-IV-----
Tr15                KKKNGKDIGPWIGAITNHL-W-WCSQTCDHDVNLLIDKWKSLLFHIANKHTF-RSL-KNYTKCDH-KRLPAS-ESEDKN-WITTSHPAHTVLTQIITDPKLIKDISKVEKFC-HTG-ELENFHSK-VLKFR----PKRIYFGMDSMH-ARTM----LAILSHNKNIG-R-PQATIQDATKSSLELGQ-KR-FKIVYPKQK-KDW-VA----KPIYEKVTDSHLFDIMLDSAR-IV-----
Tr16                RRRNCAELCSWIDPTVNHF-W-WSLKTSNGNVELLLEKWASLVNHVINVHEW-PGTTILYHNCAHSQREMPC-E---RK-WLHRESTAFNTFRSIVLSKRVICDLSNLSHFY-QAR-QNKCYHND-MLKCH----PRLYHDSSEAML-ARTE----LSAMHHNFITE-R-AQTQL----KTEL------R-HRPGFFREN-KER-LI----KSVAKTTSNDFFLDILKDVAR-YV-----
Tr17                KKRNCSELSQWGAPVRNHL-W-WAVRSCEGNTELMMEKWNSLIYHIIGVHKW-RTG-ALFHTCEH---APQE-DEVTRS-FLVKGSRAHRKLREIVLSRDINKNLPKLRNIC-HMG-ELDVFRSN-ALKYR----PKQSHFSAHSLI-ARTQ----LAALDHNRNVH-H-QLAI-LANGRDEEVLRE-DY-PRVEFRELK-KDW-RV----KPVYDLARCDFIFTIMKDVVR-YV-----
Tr18                KKRNCSDIASWITPITNHL-W-WSSQTCKQNVNVLIDKWKSLLFHIANKHTF-KSL-QYYQKCQH-QRLTAA-QKKHTR-WIGSNHPAYNSLKLIVNDT--LRDIRKIEKFC-HTS-DLENFHSK-VLKYK----PKRLSFKMDGMI-ARTA----LGALSHNRNVH-R-QQAVVTSAKKSTLPVGK-KR-FKIVFPKQK-EQW-VA----KPMYEEVLDEHLFEIMSDSIK-IL-----
Tr19                KKRHCRDIYKWISAITNHL-W-WCAQTCNQDVDVLLDKWKSVMFHLSNKHSF-HSL-ENYKKCQH-KRIPAV-KARRFA-WITSEHPAYAPLSKIINDKALLRDISKIEKFC-HTR-DLESFRSN-VLKYK----SKRLSLNMDSMY-ADTI----LAALSQNRNVS-R-EEAKLNCPQNSPLPFGE-KH-YKVSFCKQN-----------NRVEDDVVDDHLLDIMSNSLK-IL-----
Tr20                KKRKCKDISRWISAITNHL-W-WSAQTCDQNVDLLLDKWKSVLYHVANEHSF-GAL-KHYQKCQH-RRLTPE-EQKQTC-WIGANHPAHSVLAEIINDTKFLNDLSHIGKFC-HTG-DLENFHSK-VSRYK----PKRLSFPMDSVI-ARTT----LAALSHNRNVK-R-KQGSVQCPAKSTLSFGQ-NR-DQIVFPKLK-KDW-AA----KPVFEGGVDDHLFEILQDCLK-IL-----
Tr21                KRKQCKDLGLWVESIIRHF-W-WCVRNCQGDEELLREMWVSVLHHTVNQHRW-RGS-RKYFKCSH-GRLSKE-QSESKE-WLLKDSAAYLQLTDIVTNPILIGDLQRLVNNC-HTG-MLECYHSK-MLKFR----TKRIHFTMDSMV-ARTS----LAVLSHNFNTN-R-QQAVVKRQTTGGAQVGE-KR-TSLVFPRSR-KRW-VV----RTIYEKTSNEHLFHLMEDLLN-IV-----
Tr22                KRKQCKDLGLWVESIIRHF-W-WCVRNCQGDEELLREMWVSVLHHTVNQHRW-RGS-RKYFKCSH-GRLSKE-QSESKE-WLLKDSAAYLQLTDIVTNPILIGDLQRLVNNC-HTG-MLECYHSK-MLKFR----TKRIHFTMDSMV-ARTS----LAVLSHNFNTN-R-QQAVVKRQTTGGAQVGE-KR-TSLVFPRSR-KRW-VV----RTIYEKTSNEHLFHLMEDLLN-IV-----
Tr23                KRKQCKDLGLWVESIIRHF-W-WCVRNCQGDEELLREMWVSVLHHTVNQHRW-RGS-RKYFKCSH-GRLSKE-QSESKE-WLLKDSAAYLQLTDIVTNPILIGDLQRLVNNC-HTG-MLECYHSK-MLKFR----TKRIHFTMDSMV-ARTS----LAVLSHNFNTN-R-QQAVVKRQTTGGAQVGE-KR-TSLVFPRSR-KRW-VV----RTIYEKTSNEHLFHLMEDLLN-IV-----
Tr24                RKKNCGEVAFWIRPITNHM-W-WAAQTCRQNADLLVEKWKSVLFHISNEHAF-PSF-VHYKKCQH-RRIMAA-QRRSYR-WIGPAHPAHSALRKIIMDPLLLRDLRKVGKFC-HTG-ELENFRGK-LIKYR----PKCLSFNRDSVI-ARTC----LGILAHNRNVH-R-QEVV--------------KK------PLS------LD----KPIYAAVVDDHLFDILSDSID-IL-----
Tr25                KTRKCKDIARWISAITNHL-W-WSAQTCNQNVDLLLDKWKSVLYHVANKHSF-RAL-KNYNKCQH-RRLTPE-QKKEAC-WIDANHPAHSVLAEIINDNRFLKDLSHVEKFC-HTG-DLENFHSK-VLRYK----PKRLSFRKDSMI-ARTT----LAALSHNRNVN-R-KQASVKCPAKSTFPFGQ-KR-FKIVFPKQK-KDW-VA----KPIFEGVVDDHLFEILQDCLK-IL-----
Tr26                KKKMSEKIELWINPIINHF-W-WSIKTCGGDGNMLRERWQSLLYHLTNQHNW-VDL-DLYSSCSH-KPLTWK-ERKATP-WLKKKSLAYKHLVEVVKDPQMEKDFPHLSCYC-HTG-YVVSFHSY-IRKYR-----ASLALDMDSME-ARTK----LAVLAHNHNIG-K--QGKIRVPVKHKKWFDM-QKEMKIA-SRAN-SRW-LV----KTVHGEMSTAHLKGLLTDLLR-MC-----
Tr27                KKRACADLKLWHNPIINHF-W-WCCRRCKGNEQELREKWQSLLYHVQNKHKW-KKG-KKYQKCAH-LPLSND-EIQTTQ-WLDKYSPAFANLQEIVKNDQIEKDLPHLTYFC-HTG-IIETYNSL-SLKFR----LKRIHYGIDSME-ARSK----LAALTHNFNVG-R-QQAVVKVQKHTGEAVGNIQN------PK-----W-------Q--FSE--------------K--------
Tr28                KKRKCGSIARWIPAITNHL-W-WSAQTCKQNVNLLIDKWKSVLFHVANVHHF-PSL-KLYQKCQH-ERISKM-EEKNTE-WIHSDHPAHAALAEIINDPLLLDDISKTETFC-HTR-DLEAFYRT-IEKCQ----SEHLPLSIDELY-ARTI----LAALSYNRNVP-R-EQTSVIWPELSNLNTGD-------TFNKVEESEW-VP----KS--ED-RESYLLDIMSDSVK-IL-----
Tr29                KKKNCSIIADWIPAITNHL-W-WSSQTCNQNVDVFMERWKSALFHIANVHSF-PSL-KFYKKCQH-ARISET-EQQETN-WIPFNHPAHEALVEIISDPILLDDIAKAEKFC-HTR-YLEAFYSK-IEKYQ----SGHLSSVTDDLH-VRTA----LAALSYNRDLD-R-GQPSV-GPLKSPLD-AE-NP-HKIVFPEER-RDL-VL----DTIQEDAAESHFLDMSSQIVK-IL-----
Tr30                KKRKCSQIRKWIPSIMLHF-E-WSSRTSHGDVALFREKWQSLLMHITNHHKW-DGS-KVCHVCCH-KALVR--HSRPLP-WIKMWCPAFHALREVILSSRLAKDLALLSQFS-HGE-AVLLYHRL-LQKYM----PSSVHLRMDAVD-ARTK----LAALTYNANVHMRRTKAEVSVEVSG----GA-DY------------SWPTL----KSLSKPDSNAHLLRMMVDAIK-LC-----
Tr31                KRRKCSQISKWIPAIIQHL-E-WVSRTSHGDVALFREKWQSLLMHITNRHKS-DSS-IVCHACRH-KALNRC--LRSLP-WIKKWTPAFHALREVVLSSNLTKDMARFSQFS--LGKDILLYRRL-LKKYR----PSDVRFRMDSAD-ARTK----LAALTYNAYMH-T-GQ---RRPKVSPRATGG----SDLQIDKVD--NWSAL----SSTSKPNSNGHLLRMMVDAIK-LC-----
Tr32                KKRKCKDIAQWIGPITNHL-W-WCAQTCDQNVENLLDKWRSLLYHIANKHTF-KNL-KTYKSCQH-KQLPAE-ESRDRK-WITPSHPAYSTLVGILNSPLLIKDISKIEKFC-HTR-DLENFHSK-ILKYR----PKKISFNIDSMY-ARTM----LAVLSHNKTVN-R-PQVTI-CSKKHKLFVGE-KK-NYSGISQGK-E----G----KPT-------HLLNIISDAEK-IV-----
Tr33                KRRKCSQISKWIPAIIQHL-E-WASRTSHGDAELLREKWQSLLLHVTNHDEL-DGF-RVCHVCCN-KALNP--HSRSLP-WIRKWSPAFHALRGVILSSKLTKDLACLSQFS--SGKEVTLYHCF-LQKYR----PNKISFRMDAVD-ARTK----LAALAYNANVH-M-----------------E----HKLSFVSSKVKDS-VDPSLLKPPHRLNSNTHLLQMMLDAIK-LC-----
Tr34                RKRSCGDIAAWTPAVINHL-W-YAAKCSAGDGDLLREKWRSILRHVRNEHQWTNGL-SA-HSCGH-RRLNLL-ESKQRR-WLKVDSRAYQQLKKIVTDPLVLGDLTHLSSLS-HTD-QIEVFHSF-MLKYQ----PRRAHVSLEAME-ASTK----LAALAHNANVH-R-HQSVARIPGR---NIG-----FH---SKTR-KPW-TT----KRIYDPESSAHLFPVLVSALK-LA-----
Tr35                KKKKYSEIKEWISPTINHL-W-WCARTCNGNPDLLREKWLSLLYHVTNVHQW-QTG-HLYHECAH-SALAPH-LDHKRK-WMQDESTVFEQFKDIITSHKILDDLAHLSHFC-HTG-ELEVYHSN-LLKYR----SKRHHYFIDGMI-ARSQ----LGALDHNFNVQ-R-EQARVCTTGPECDSVGS-LR-HKLEFTKSK-KDW-VV----KPIYTRPSNSFLFELLKDIIR-LA-----
Tr36                KKSKLKDIAPWIGPITNHL-W-WCSQTCDQNVENLLDKWRSVLYHIANKHTF-RNL-KTYKKCQH-KKITAE-EMKEKK--------------------------------------------------------------MD------------------------------------------------------------------------------------NSIS--------
Tr37                KKRNCRDIASWISPITNHL-W-WSSQTCKQNVNVLIDKWKSLLFYIANKHTF-KSL-RYYKKCQH-RRLTAA-QKKYIR-WIGSNHPAHNSLKLNINDNSLLLDIGKIEKFC-HTS-DLENFHSK-VLKYK----PKRLCFKMDGMI-ARTA----LAALSHNRNVN-R-QQSVVKSAKKSTLPVGK-KR-FKIVFPKQK-KQW-VA----KPIYEALLDEHLFEIMSDSIK-IL-----
Tr38                KKRNCSELAQWSAPVRNHL-W-WAVRSCEGNTELMMEKWNSLIYHVIGVHKW-RTG-ALYHGCEH---APQE-DEVTRS-FLGKGSRAHRKLREIVLSRDINKDLPKLRNIC-HMG-ELDVFRSN-ALKYR----PKQSHFSTHSLI-ARTQ----LAALDHNRNVH-H-QLAI-LANGRDEEVLRE-DY-PRVEFRELK-KDW-RV----KPVYDLARCHFIFTIMKDVVR-YV-----
Lv1                 KKKNCEEIKEWISATVNHL-W-WCSRTCDGNVEILKEKWTSLTKHVTNVHQW-ESG-KYYHQCDH-EELSEL-TNRKRK-WMTEGSTAYITFTDIILSFTLLKDLDQLSHFC-HTG-ELEVYHSK-QLKYR----SKRYHYFMDGMF-ARTQ----IGALDHNYNVQ-R-EQAKVSVSGMGRDPCGS-MR-HKQEFTKAR-KDW-VV----KPIFEPTSNAFLFSMMRDVM--LCA----
Lv2                 KKRNCSELSEWSAPVRNHL-W-WAVRSCKGNTELMMEKWNSLIYHVIGVHNW-KTG-ALYHGCEH----APE-EDDAARLFLRKDSRAYRKLREIVLSQNINKDLPKLVNIC-HTG-ELDVFRTN-TLKYC----PKQNHFSRGSLI-ARTQ----LASLDHNRNVH-Y-QLAI-LANEKDEEALRE-DY-PRVEFSQLK-KDW-RV----KPVYDIERCDFIFTILKDVVK-YV-----
Lv3                 KKKKYTDLTEWITPTINHL-W-WCARTCNGNPDLLKEKWLSLLYHVTNVHQW-QTG-HLYHECAH-SALPPD-LDHKRK-WLIDGSSMFEHFKEIITSRRFLKDLSYLSHFC-HTG-ELEVYHSN-MLKYR----SKRHHYYMDGMI-ARTQ----IGALDHNFNVQ-R-QQATVHITGPGRDSVGS-LR-HKLEFTKTK-KDW-VV----KPIYTRHSSSFLFEILKDIYF-FA-----
Lv4                 KRRACPELEKWIPAITSHL-W-WAAKTSHGNTDLLRERWQSLLLHIVNQHTW-KGS-IFCSACTH-RPLAAR-EQTSCP-WLKKGTSALYSLRQVIMNPRVTNDLVHLSQ-CSHAE-EIEMYHRF-VFKYR----PKYMHLKMDAIE-AMTK----LAALAYNANIH-N-QKLRCFSAIKGKGVFGTVTH-VR----KCR-----LA----KPVYHDASSEHVIPMMTDVIK-IS-----
Lv5                 KKKNCADIGPWIEPIINHL-C-WSAKTCNGDKSVFKEKWKSLLYHVCNIHSW-QSG-NTYKKCSH-NDVTLE-NCNLRM-WLKRNSTAFRTLKFIVLDAKLQRHLQKSSEFG-DVA-ELKDYHHK-ALKYR----PEGVHFHLDGII-ARTQ----LAALDHNFSTC-L-TKAIESVVEEGGVPIGT-RR-YRMHRTDGR-NKW-LR----KNVCEKNATGFVFQILDNVINSIY-----
Lv6                 KKKNCKDITYWITPIINHL-W-WSAQTCQQNKDILMDKWKSVLFHIANKHEF-PTL-VHYKKCQH-KRFTLA-QKKDCK-WIGSTHPAHAALRKIIMDKYLLKDLGQVEKFC-HTG-ELENFHSK-VLKYR----PKRLSFRMDSMI-ARTS----LAVLSHNRNVH-R-EQAVVKNPHASSAGLGE-KR-FKIVYPKQK-KEW-VA----KPIFESVVDDHLFQIMSDSLN-IL-----
Lv7                 RHPKCQDLTWWITPIQNHL-W-WCVQTSRQNPDVLVDKWQSVLFHIADVHNF-PRL-KHYKRCMH-RTLTPQ-KRKEVK-WIKLSHPAHRALEKIVSDPALLRDLRHITKIS-HTP-QFEVYRSM-SLKYR----SKSYSYKMDSAY-ARTI----LAILSNNHSVG-R-EQPM--TPSKSDLPASG-KS-YQTVLPMQEMGR--VV----TPIYRQGVENHLFKLLNDCLS-IM-----
Lv8                 RHPKCKDLTWWITPIQNHL-W-WCVQTSRQNPDVLIDKWQSVLFHIADVHSF-PRL-NHYKRCMH-RTFTPQ-KRKEVK-WIKPNHPAHRALEKIVSDPALLRDLRHITKIG-HTP-QFEVYRSK-SLKYR----SKSYSYKMDSAY-ARTI----LAILSNNHSVG-R-ERPM---PLKSDLPVRE-KS-YQTVLPMQE-KNW--R----TPIYRQGVENHLFKLQNDCLS-IM-----
Lv9                 KRKNCSQISQWITPAVNHL-L-WASKTCDGSADLLKKKWQSLMNRVATVHKW-ETA-QLFHGCAH-ENLSV--RCK-RK-WMKCGSTAFNRFRDIVMSARLIRDLNPLSKIC-HTD-ELKLYHNN-LLQYW----PRTLH-CVEDMV-VRTQ----LAALDYNYSVH-R-GKSPENFTHVQD-PSGA----LQ---PKVR-----------KCVFDAASKAFLLVILKDIVT-FA-----
Lv10                RKRSCTELCNWIDPTVNHF-W-WSLKTCGGNVELLLEKWASLVNHVANVHEW-PGTTKVYHSCTH--NLTEM--AHERK-WLQRESTAFNTFRDIVLSKRIMKDLSRFCQFF-QTR-Q-NSFNCREIFRHH----PRLYHDSSDAMV-AKTA----LAAMRHNFIIE-K-EQAQLKTE----------LR-HISGFSVEN-KEC-LI----NSVSKTTSNEFFLDLLKDVDK-FI-----
Lv11                RKRHCHDICKWISAIANHL-W-WCAQTCNQDVDVLLDKWKSVMFHVANKHRF-HSL-ENYTHCQH-KRLTAV-KERKCA-WITSQHPAHTPLSRIINDRALIRDISKIEKFC-HTR-DLQSFHSK-ALKYK----SKRSSFNMDSMY-TDTI----LAALSHNRNVS-R-EEAKINSAQNS--PFGE-EH-YKVAFSKQKHRD---D----K--YEDVVDDYLFDIMSNSLK-IL-----
Lv12                KKRKCKDIAQWIGPITNHL-I-WCVQTCDQNVDLLLDKWRSLMYHIANRHTF-KNL-KHYTKCQH-KQLTAQ-EQKVRK-WITPAHPAYSTLVDILNNRLLIRDISRSEKFC-HPG-DLENFHSK-VLKYR----PKKTAFQMDSVY-ARTM----LAVLSHNKNVN-H-PQAMVHCSKTTPLAAGN-KS-FQLVFPEQK-KEW-IP----NPIYEQVTDVHLFDIISDAAE-IV-----
Lv13                KRRNCSHISKWIPAIIQHLEWSW--RTSHGDAELLCEKWQSLLLHITNHNK--RDASRVCHACCH-KALNCF--SRSLP-WIKKWSLAFHALRGVIFSSKLAKDLACLSPFS-NGR-EIKLYHRF-LQKYR----PSEVYFRMDAVD-ARTK----LAALTYNENVH-M-GHTKPKVSSKVKESVCL-QR-HKVVYSGTN-SPSELK----SPP-KLNSNAHLRKMMLDAIK-LC-----
Lv14                KKKKCSTIASWLCAITKHL-W-WSAQTCKQNVDLFIEKWKSVLFHVANVHSF-QSF-KFYKKCQH-KSISDT-EKQNTN-WIPLNHPAHAALAEIINDPFLLDDIAKAEKFC-HTR-DLEAFYCK-IEKFQ----SEHFSLVTDELY-VRTA----LAALSYNRNGT-REPQSMW--PLKAPLNVGE-NF-LKFFSPTER-RDL-VL----DAIYEDSVESDFSDIMSQIMK-ML-----
Lv15                KKRSCRDIATWTPAIIKHL-W-HVCKCSAGDCDLLQEKWRSILRHIRNEHQWTNGL--VLHNCGH-RRLSSA-EAKQRR-WLKVDSRAYQQLKKIITDPLVLGDLTHLSSVS-HTD-QVEVFHSF-MLKYQ----PKRAHVSQDAME-ASTQ----LAALAHNANVH-R-HQSM------SR-NLGA-NRELSSTLPKAR-KPW-TA----RRIYDPKSSAHLFPMLVDILK-LA-----
Lv16                KKRDCRDICKWISAITNHL-W-WCAQTCNQDVDVLLDKWKSVMFHIANKHRF-HSL-ENYKHCQH-KRLNAV-KERKCA-WITSEHPAHTPLSRIINDQALLRDISKIEKFC-HTR-DLESFHSK-VLKYK----SKRSAFSMDSVY-TDTI----LAALSHNRNVS-R-EDVNINCAQKSPLPFGE-KR-CKVAFSKSENRD---D----KFVGEDVVENYLLDIMSNSLK-IL-----
Lv17                RKRHCHDICKWISAIANHL-W-WCAQTCNQDVDVLLDKWKSVMFHVANKHRF-HSL-ENYTHCQH-KRLTAV-KERKCA-WITSQHPAHTPLSRIINDRALIRDISKIEKFC-HTR-DLQSFHSK-ALKYQ----SKRSSFNMDSMY-TDTI----LAALSHNRNVT-R-EEAKKNSAQNS--PFGE-EH-YKVAFSKQK-HR--AD----KYVCADVVDDYLLDIMSNSLK-IL-----
Lv18                KKRKCKDILNWISAITNHL-W-WSSQTCNQNVKILLDKWKSVLFHIANRHSF-GSL-KYYKNCQH-RRLSPQ-GKKDAR-WIVANHPAHSVLAEIIHDKRFLVDLQHAEKFC-QTV-DLENFRSK-VLRFK----PKRLSFRMDSMI-AQTI----LAVLSHNRTVQ-R-KQALV---------FGE-KH-RKIVFPNRK-KQW-VA---------EVVDDHLFEILHDSVK-MLTGQLH
Lv19                KKRKCSQISKWIPAIILHF-E-WSSKTSQGNVSLFREKWQSLLMHITNHHEW-GGS-VVCHACDH-KALLR--NSRPLP-WIKLWCPAFHALREVILSFKLTKDLTRLSQFS-HGR-EILLYHRL-LQKYR----PGVVRLRMDSVD-ARTK----LAALAYNANVH-M-GHTKSQVSLETSGGAAQ-ER-LKMDYS------WRTL----KSLSKPDSNAHLLRMMVDAIK-LC-----
Lv20                MKRKCSNIASWIGAITKHL-W-WSAQTCQQNVDLLIEKWKSVLFHVANVHSF-ESL-KYYKQCQH-GSISDA-EQMNTN-WIHWNHPAHAALAEIINDPVLLEDIKKTEKFC-HTR-DLEAFHSN-IEKFQ----SEHLSLSTDELY-VRTA----LAALSYNWNMT-G-EQTSIIRPGTSVSNIDE----------NLK-SEW-V------PKYED-CESSLLNIMLDSVK-IL-----
Lv21                KKRKCSSIASWLCAITEHL-W-WSAQTCKQNVDLLIEKWKSVLFHVANVHSF-QSL-KFYKKCQH-KSISDM-EKQNTN-WIPLNHPAHAALAKIINNPVLLDDIAKADKFC-HTR-DLEAFYCK-IEKFQ----SEHFSFVADELY-VRTA----LAALSYNRNGT-REPHSMW--PLNSPLNVGE-NH-HKLVFPMER-RDL-IL----DPMCENGVESDFSDIMSQIMKRLL-----
Lv22                TKRNCDSIASWIDSITKHL-W-WSAQTCQQNTDLLIDKWKSVLFHVANVHSF-DSL-KLYKQCQH-GSISYE-DQLNTD-WIHCNHPAHSALVEIVNDPVMLDDIKKIEKFC-HTR-DLEAFHST-IEKLQ----SDHVSLSTDELY-VRTV----LAALTYNWNVT-G-SPTSIIQPDTSFLNIGE-N--FKS--------QW-VS----K--YE-YCESSLSNIMLDTVK-IL-----
Lv23                RRKQCKVLGLWVETIIRHF-W-WCIRNCQGDHDMLREMWLSLLHHIVNKHTW-RGC-KKYPKCTH-AKLTME-QCQSKE-WLVKDSAAYLMLSEIVTNPILIKDLK-------ASGEQLP-HRTLGVLPFKNIEIPNETHPFYHGLYDSKNKSCSPFSQLQY-------QQGTGCCKTKNK---G-----------RSR--SW-------GKTYQPS-----IPTIPEQVG--------
Lv24                KKRKCKDIGQWISPITNHL-W-WCSQTCDQNVDLLLEKWQSLLYHIANKHSF-HNL-KNYRKCQH-KPLSPQ-EQETRK-WITAGHPAYSSLVCILTDPAFIKDISNTDKFC-HTR-CLKNFHSK-ILKYR----PRRILFRRDSVH-ARTM----LAVLSHNKNVN-R-PQATILVPKITPLAYSE-KP-SKIVFPTQK-SEW-LT----KPVYESGTDDHLFDIMSDATK-IV-----
Kolobok-1_DR1p      QIKGCEKLRKWLRSIKNHI-Y-WTAASSTTGPERV-AKWTSILNHVQDKHVH-ED--PNFPACLHPQRISRD-KNK----WLSAATMPFYKLEKVLANKRILKDVAKLSPHH-QTS-TVEAFHSV-ILRFA----PKNVVFPFLGML-CRLY----LAALHYNENAG-R-PQAT---SATGK-PI------YKLAFPKAKKGEY-RV----REVKTQ----QTFGYVEELLD-LI-----
Kolobok-1_CTe_1p    NTKGCEALMQWIRAISNHL-W-YSASTCKKNAQLLKEKWLSVLHHVAGVHSW-SGG-QKFNKCDH-GPMSDAPPGMEVA-YLKRTSPAFKALKDVVSATALLKLLPKLTKFC-HTG-TLEVFHSF-LLRFC----SKRQYFPYPGMK-ARLM----LAALSWNA-LQ-R-SNAT------EKVDVVK--------WSKRK-GAW-VLEERRKKQASDSVPPQLKSLMSRVVE-VA-----
Kolobok-2_BF1p      SRRAKKDLGPWIKFIVNHL-W-HISDTCQGDDVLLQEKWLSMLKHIGNIHVF-PEN-SIFTRCAH-PHLDRA-DTDDIM-WLRPGSPPHQALYNIATNKTLLKDLSHLTGFK-HTG-TVEVFHSM-LLKYA----PKRQHYFFHGMK-GRLQ----LAVLDHNENVL-R-EQAK---------TLEDEAR-RSAAFSKRT-KRW-VY----RRLFEGKTYEFREYLMADVVRRQASGQYK

                        .    +    .    +    .    +    .    +    .    +    .    +
Tr1                 ---KGQLLHRWESRC*------------------------------------------
Tr2                 ---CGDIDIQWQPVRAD-L-----PSNIASKPRPDKYEAVEKHLSR-----FKTQS*-
Tr3                 ---TGRLNHSWIPRCVNE------PTNIPSEPVPVQLWTIAPSVSH-----FPVLL*-
Tr4                 ---EGEVNIERQTLSDR-L-----PDNPSLEHRPMKNES------------W*-----
Tr5                 ---KGEIVPRWESHSKG-L-----AGNIASKNSPEKPVVVAQYSLNNVTVVWGNT*--
Tr6                 ---TGEIGHDWVSISKQ-M-----PQNIATTERPEKSEVIEKHMSR-----FSRC*--
Tr7                 ---SGELIHNWDSKSSS-V-----PANIATLERPDKNELISKHVSR-----FAR*---
Tr8                 ---KGEVVHRWESHTKG-L-----PKNIASKERPEKSVVVAQHYSR-----FGTC*--
Tr9                 ---SGELIHNWDSKSSS-V-----PANIATLERPDKNELISKHVSR-----FAR*---
Tr10                ---SGKLCHSWFPRVTTAL-------------RP*-----------------------
Tr11                ---KGELVHRWESHAKG-L-----PKNIASKERPEKSVVVAQHYSR-----F*-----
Tr12                ---QGKLSHSWDSRNST-M-----PDNIATVQRPDKSLAVQQHMSR-----FQDRDM*
Tr13                ---SGDVLCE*-----------------------------------------------
Tr14                ---NGELVHRWESRSSK-Y-----PANIALTERPEKTEVIAKHLSR-----FERE*--
Tr15                ---NGELVHRWESRSSK-Y-----PANIALTERPEKTEVIAKHLSR-----FERE*--
Tr16                ---NGELDIKVQS*--------------------------------------------
Tr17                ---YGE-------RC---------PG*-------------------------------
Tr18                ---TGEISHEWLSMSQR-M-----PKNIATYERPEKTEVIDQHSSC-----FARP*--
Tr19                ---NGELVNQWSSRSRI-L-------------R*------------------------
Tr20                ---TGELQHDWSSTARE-M-----PNNYSYC*--------------------------
Tr21                ---EGNLLTTYESVTDT-L-----PRNLATVEKPDKASAVAEHQSR-----F*-----
Tr22                ---EGNLLTTYESVTDT-L-----PRNLATVEKPDKASAVAEHQSR-----F*-----
Tr23                ---EGNLLTTYESVTDT-L-----PRNLATVEKPDKASAVAEHQSR-----F*-----
Tr24                ---TGKLTHRWPSETKF-V-----PKNIATSDRPIKSEVIANHVYQ-----FSHS*--
Tr25                ---TGELQHDWSSTARE-M-----PKNIATVE*-------------------------
Tr26                ---NGKLVSPWKARSVH-L-----PHSARAARRPVA----------------GARRK*
Tr27                ---------Q*-----------------------------------------------
Tr28                ---FGGLQYNWISVPTF-L-----PQTVVRCKAEV*----------------------
Tr29                ---LGELEHK-GS*--------------------------------------------
Tr30                -----SINPN---PC*------------------------------------------
Tr31                ---STNSKQRV*----------------------------------------------
Tr32                ---KGELVHRWESRTGGYL-----P--ILRQNKDLKN*--------------------
Tr33                -----SLNQP*-----------------------------------------------
Tr34                ---NGEPSHSWGARETR-------------KNR*------------------------
Tr35                ---CGDIDIQWQPVRAD-L-----PSNIASKPRPDKYEAVEKHLSR-----FKTQS*-
Tr36                ---SG-L*--------------------------------------------------
Tr37                ---TGEITHEWLSMSKR-M-----AKNIATCERPAKSEVIERYLSR-----FEKS*--
Tr38                ---YGE-------KC---------PG*-------------------------------
Lv1                 ---SGELNVTWISPCAQ-L-----PANIALQERPPKKEAVEKHLSR-----FR*----
Lv2                 ---YGE-------KC------------------------------------FG*----
Lv3                 ---CGDIDIEWRPVRAT-L-----PSNIALKPRPDKDEAVGKHLSR-----FKPRN*-
Lv4                 ---TGKLQHSWFSRVTTAL-----------SP*-------------------------
Lv5                 ---RG---H*------------------------------------------------
Lv6                 ---TGKLKHQWLSQSKQ-M-----PQNIAAAERPGKSELIAKHVSR-----FSRS*--
Lv7                 ---SGDILL*------------------------------------------------
Lv8                 ---SGDILS*------------------------------------------------
Lv9                 ---EGEVNIERQTLSDR-F-----SDNPSLEQRPIKDEQ------------W*-----
Lv10                ---SGELDIQVKS*--------------------------------------------
Lv11                ---NGELVHLWSSRSRI-L-------------R*------------------------
Lv12                ---RGEIVHRW*----------------------------------------------
Lv13                -----SLNQ--IQNCNS-------RFTLLQGAK*------------------------
Lv14                ---LSELEHKGK*---------------------------------------------
Lv15                ---NGELGHSWRLHETQ-------QNS*------------------------------
Lv16                ---NGELVNQWSSRSRI-L-------------R*------------------------
Lv17                ---SGELVNQWSSRSRI-L-------------R*------------------------
Lv18                HDYTGQLHHDWSSTSNE-M-----PNN-SYCERA*-----------------------
Lv19                -----SVNPSLAV*--------------------------------------------
Lv20                ---YGNLENKWNSASNF-L-----PKTVSCKAEV*-----------------------
Lv21                ----SELEHKGR*---------------------------------------------
Lv22                ---FGHRENKWSSASNF-L-----PKTV-SCKAED*----------------------
Lv23                ---CS---YRF*----------------------------------------------
Lv24                ---KGYLIHR*-----------------------------------------------
Kolobok-1_DR1p      ---FNQVFVDPSPYVDEVLGIHIPPALSSAYDRPEMEEAISSRVTR-----FNQ*---
Kolobok-1_CTe_1p    ---SQHLQLP--PIAEPKL-----QRRGCHVAKPSKEEL--RHL--------------
Kolobok-2_BF1p      ---CGKVVLP-DPGPDI-------SGNIAPEERGDKQAIIDGYMTR-----FGLR---
```
