# Supplementary material for: Multiple massive domestication and recent amplification of Kolobok superfamily transposons in the clawed frog Xenopus
Source: Zoological Lett. 2018 Jun 16;4:17. doi: 10.1186/s40851-018-0100-4 (PMC6004289; doi:10.1186/s40851-018-0100-4)
Supplement: Supplementary file 2 — Figure S2. Repetitive XKol-Tpase CDSs (highlighted in red) were compared with their truncated copies by dot plot analyses (word size = 12). Flanking upstream and downstream 1000 bp CDSs and truncated copy sequences are also included for comparison. (PDF 717 kb) [file 40851_2018_100_MOESM2_ESM.pdf]

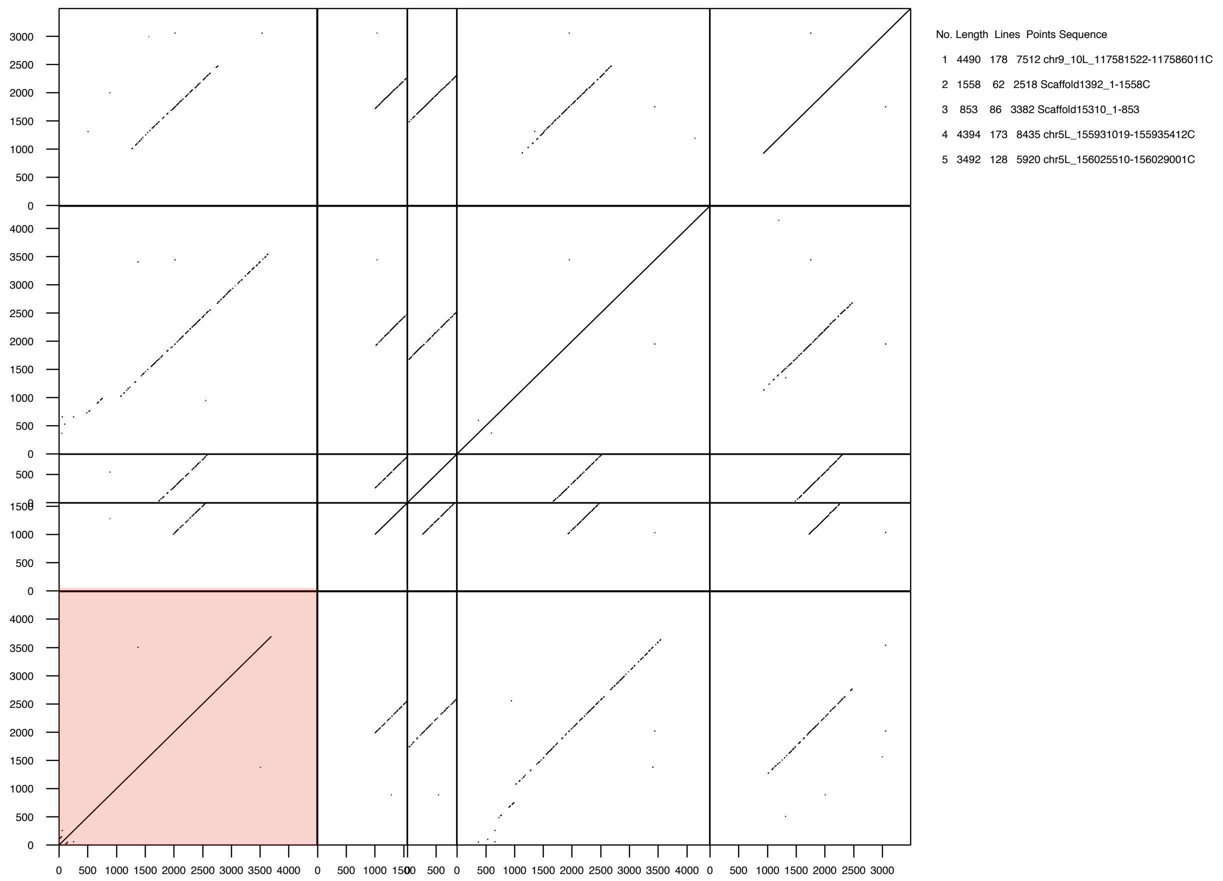
No. Length Lines Points Sequence
1 4490 178 7512 chr9_10L_117581522-117586011C
2 1558 62 2518 Scaffold1392_1-1558C
3 853 86 3382 Scaffold15310_1-853
4 4394 173 8435 chr5L_155931019-155935412C
5 3492 128 5920 chr5L_156025510-156029001C

Lv1

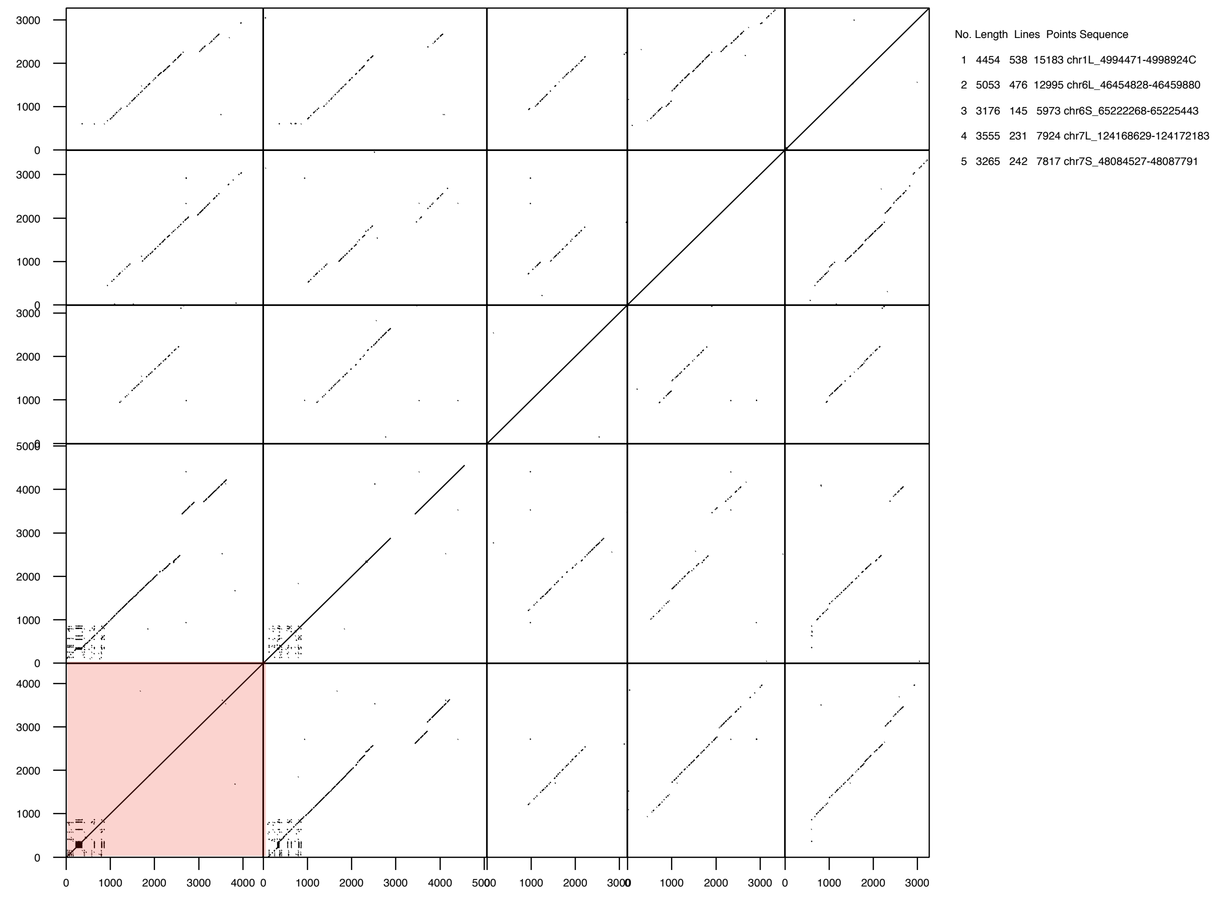
No. Length Lines Points Sequence
1 4454 538 15183 chr1L_4994471-4998924C
2 5053 476 12995 chr6L_46454828-46459880
3 3176 145 5973 chr6S_65222268-65225443
4 3555 231 7924 chr7L_124168629-124172183
5 3265 242 7817 chr7S_48084527-48087791

Lv3

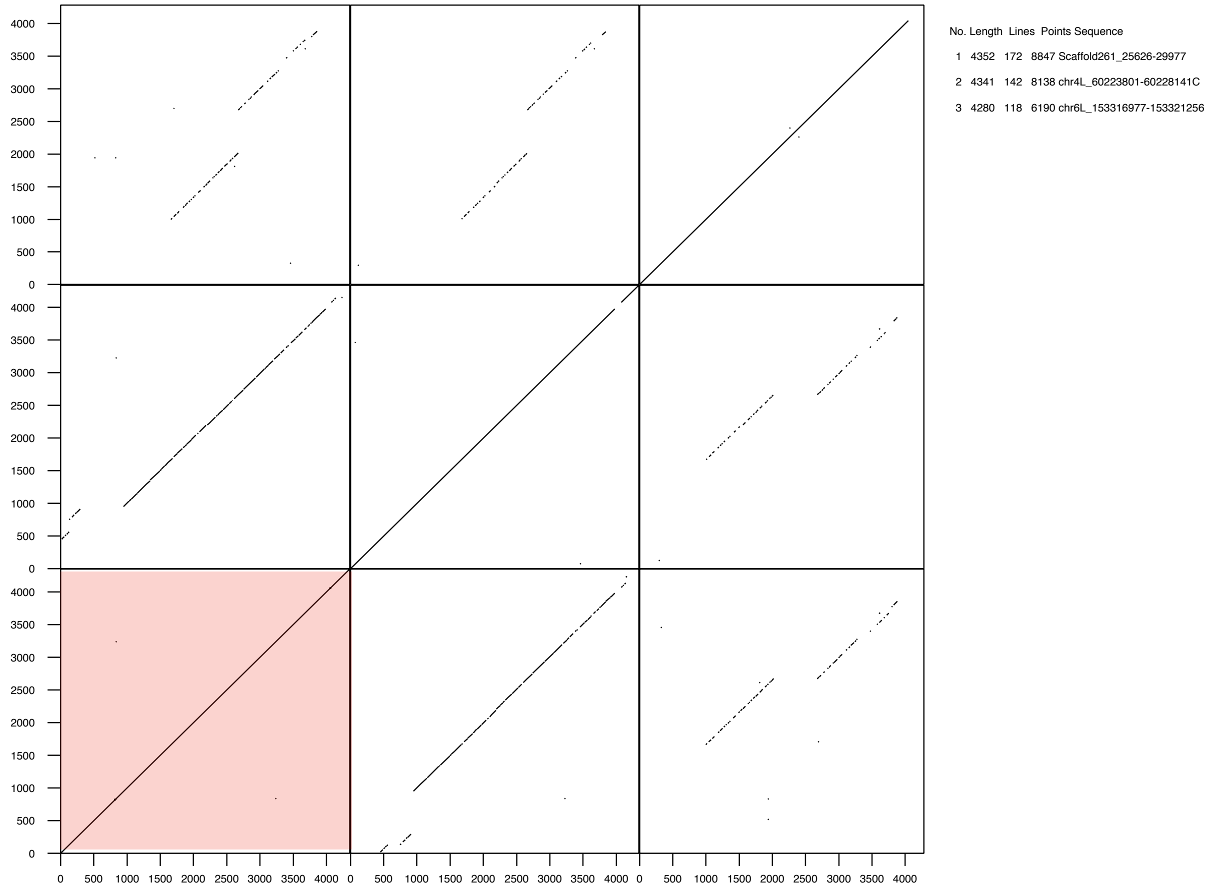
No. Length Lines Points Sequence
1 4352 172 8847 Scaffold261_25626-29977
2 4341 142 8138 chr4L_60223801-60228141C
3 4280 118 6190 chr6L_153316977-153321256

Lv6

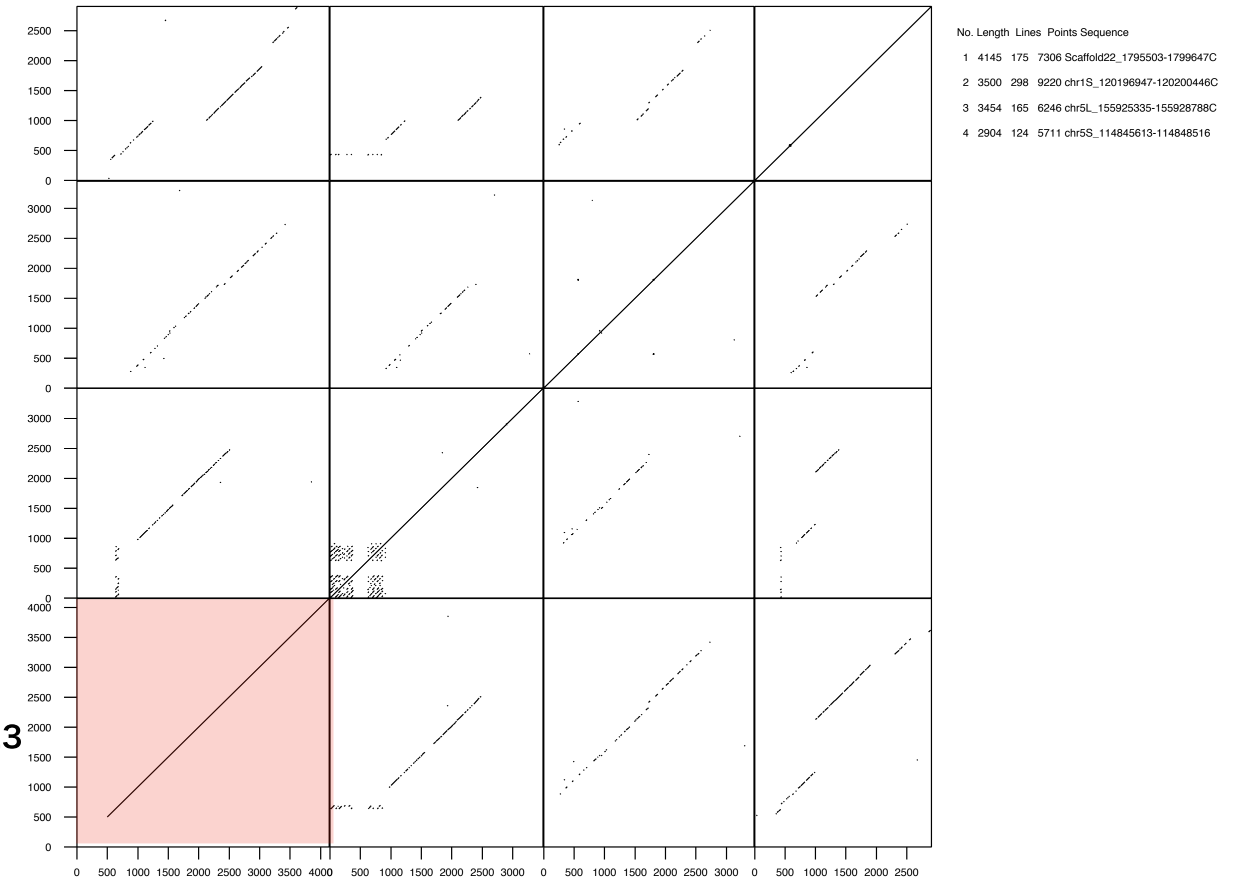
No. Length Lines Points Sequence
1 4145 175 7306 Scaffold22_1795503-1799647C
2 3500 298 9220 chr1S_120196947-120200446C
3 3454 165 6246 chr5L_155925335-155928788C
4 2904 124 5711 chr5S_114845613-114848516

Lv23

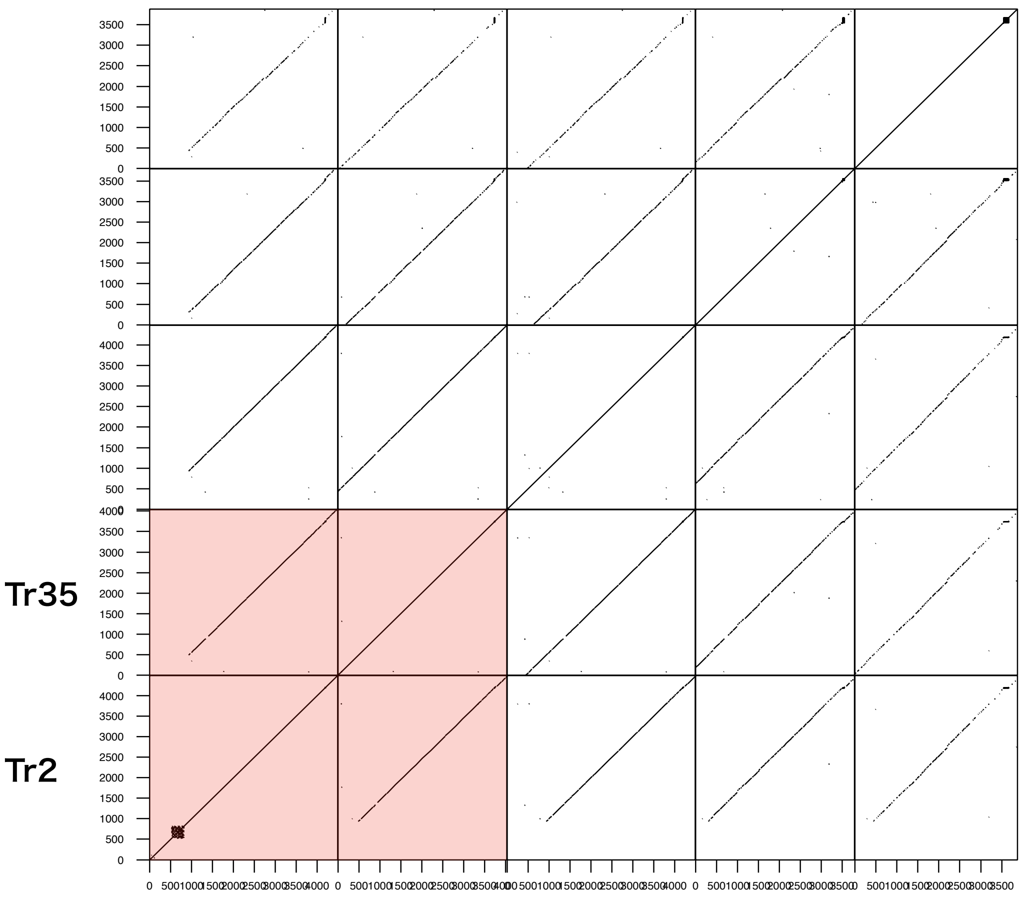

No.	Length	Lines	Points	Sequence
1	4484	522	19957	Chr02_100829975-100834458
2	4034	409	17866	Chr05_37112502-37116535C
3	4483	449	18390	Chr01_63996857-64001339C
4	3798	623	20245	Chr01_75842405-75846202C
5	3877	790	26042	Chr02_37360377-37364253

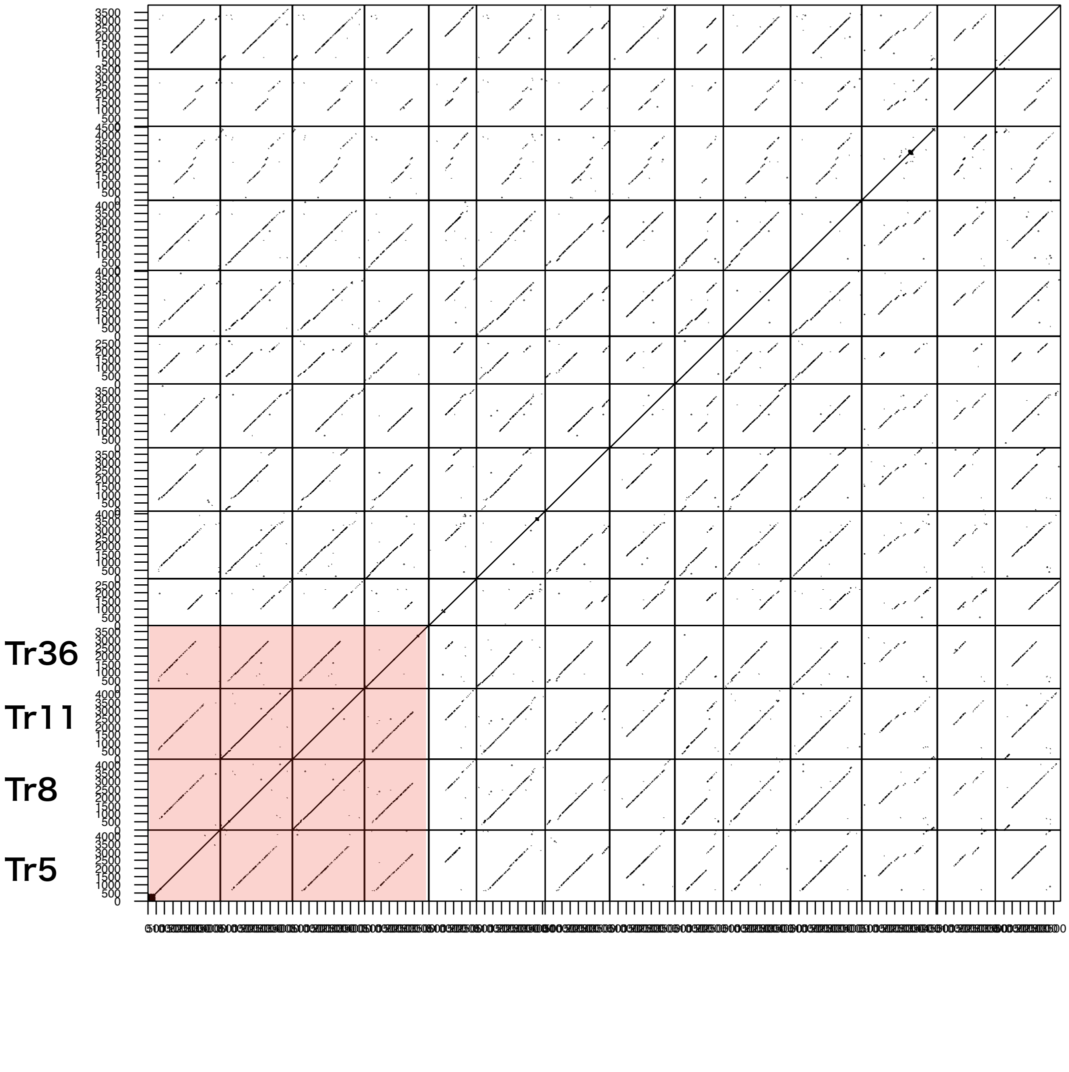

No.	Length	Lines	Points	Sequence
1	4361	951	24924	scaffold_769_43126-47486C
2	4352	903	27234	Chr08_54547260-54551611
3	4343	926	27246	scaffold_609_17293-21635C
4	3878	715	20911	scaffold_161_119530-123407
5	2873	475	12684	Chr01_71636575-71639447C
6	4143	938	25380	Chr02_79580932-79585074C
7	3888	855	23673	Chr03_136256643-136260530C
8	3928	779	22097	Chr04_49448295-49452222
9	2927	610	16197	Chr05_64713464-64716390C
10	4042	947	25126	Chr07_61524215-61528256C
11	4302	1031	27983	Chr09_44143841-44148142C
12	4551	745	19077	scaffold_381_57060-61610
13	3508	448	11166	scaffold_470_32001-35508C
14	3929	865	22925	scaffold_61_656144-660072

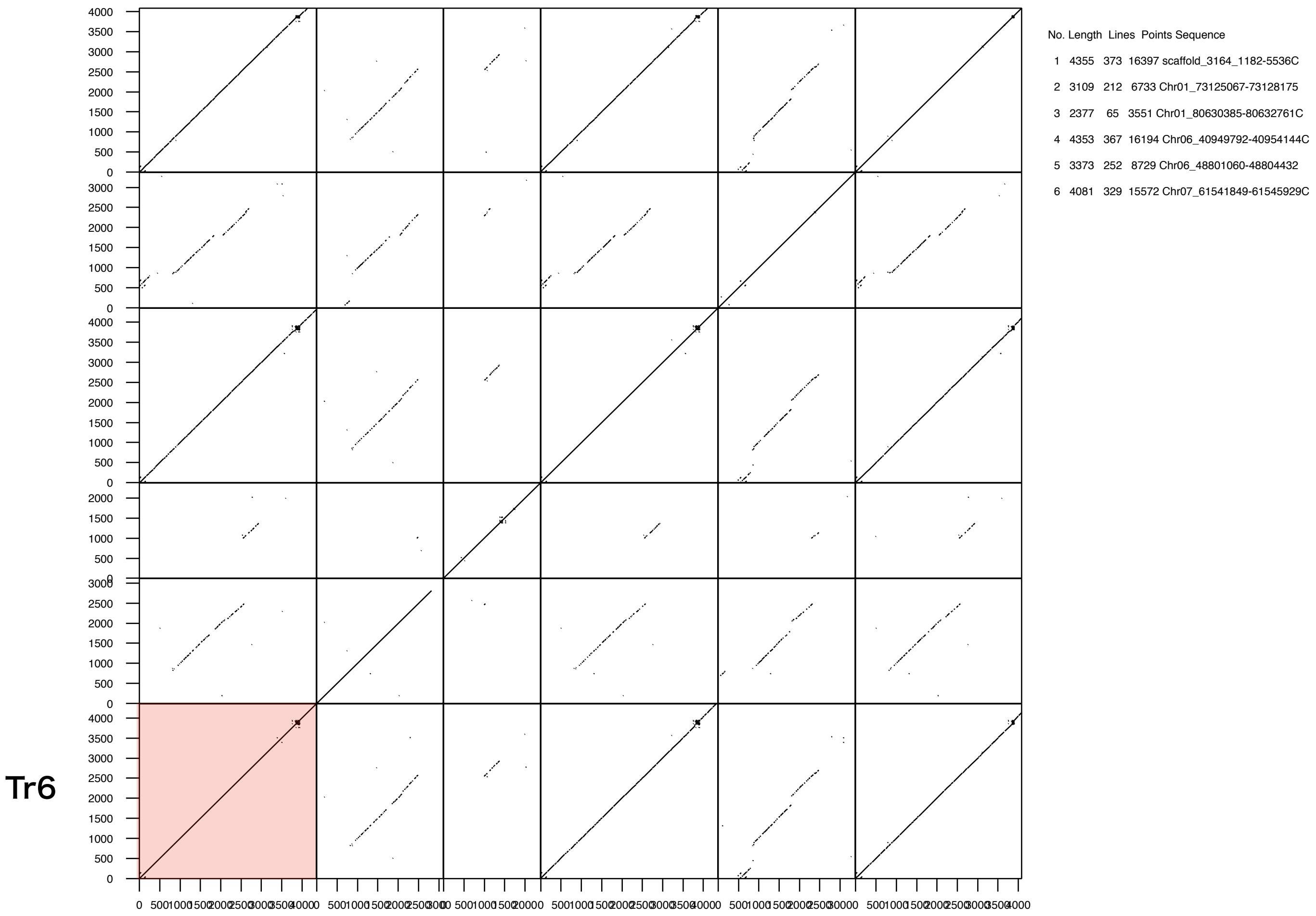
Tr6
No. Length Lines Points Sequence
1 4355 373 16397 scaffold_3164_1182-5536C
2 3109 212 6733 Chr01_73125067-73128175
3 2377 65 3551 Chr01_80630385-80632761C
4 4353 367 16194 Chr06_40949792-40954144C
5 3373 252 8729 Chr06_48801060-48804432
6 4081 329 15572 Chr07_61541849-61545929C

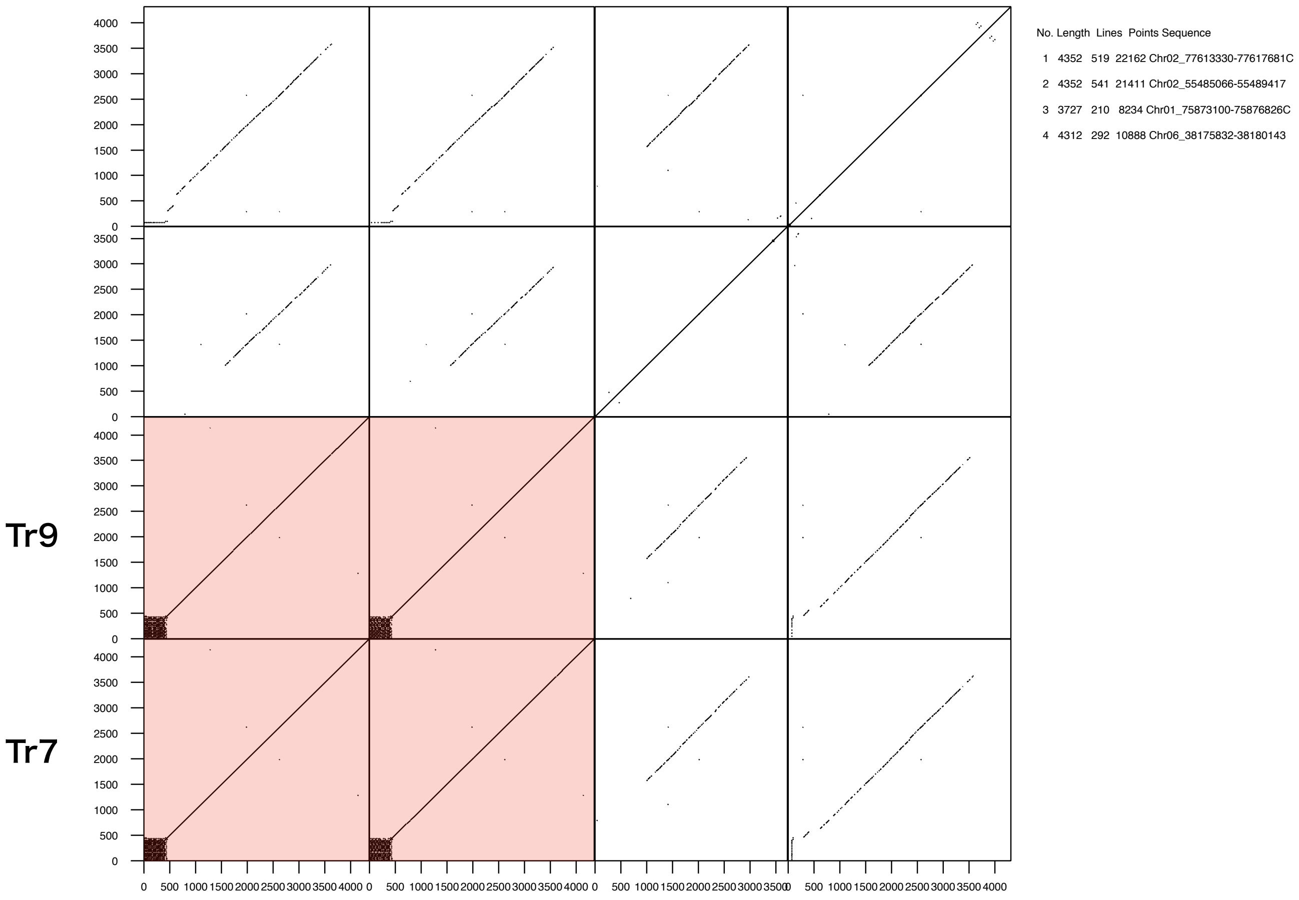
Tr9
Tr7
No. Length Lines Points Sequence
1 4352 519 22162 Chr02_77613330-77617681C
2 4352 541 21411 Chr02_55485066-55489417
3 3727 210 8234 Chr01_75873100-75876826C
4 4312 292 10888 Chr06_38175832-38180143

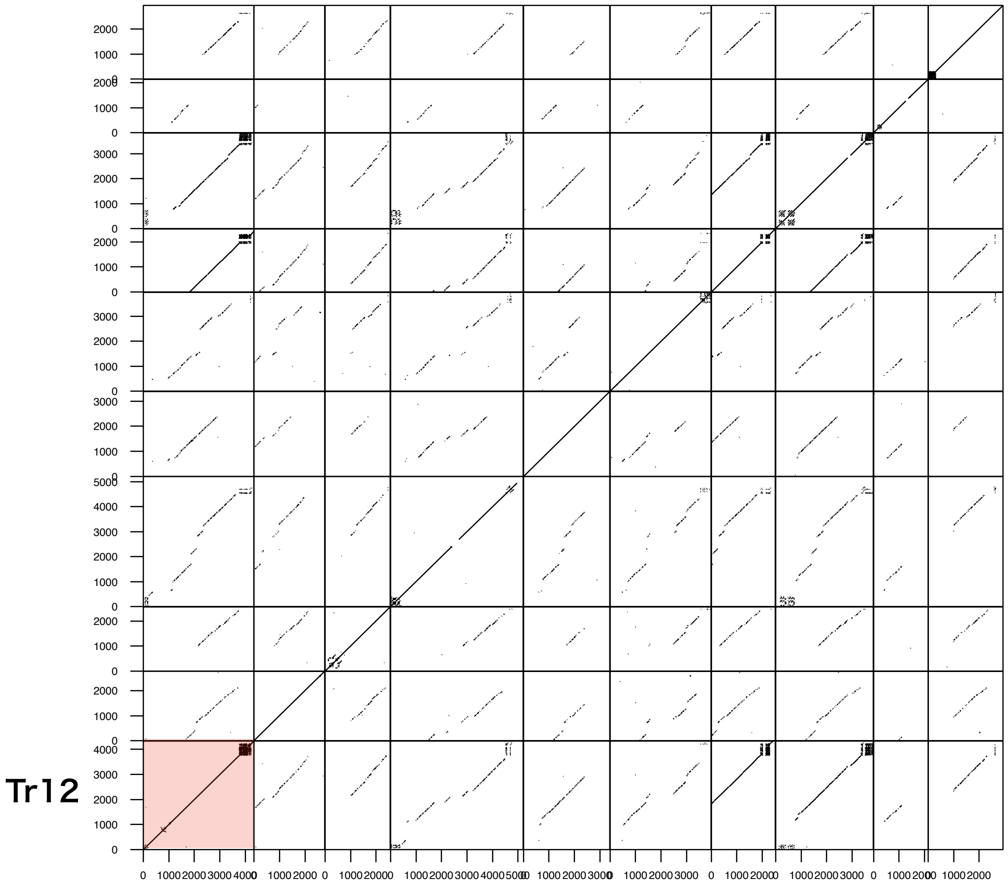

No.	Length	Lines	Points	Sequence
1	4337	1044	28777	Chr06_112307032-112311368C
2	2777	372	9467	Chr03_121999296-122002072
3	2561	347	9105	Chr04_103771015-103773575
4	5207	720	18391	Chr05_74049007-74054213C
5	3389	312	9486	Chr05_109020839-109024227C
6	3963	487	13149	Chr09_25287806-25291768
7	2518	619	17706	scaffold_2530_1-2518
8	3829	981	26014	scaffold_29_22539-26367C
9	2139	103	3983	scaffold_57_1-2139
10	2935	331	10402	scaffold_57_5848-8782

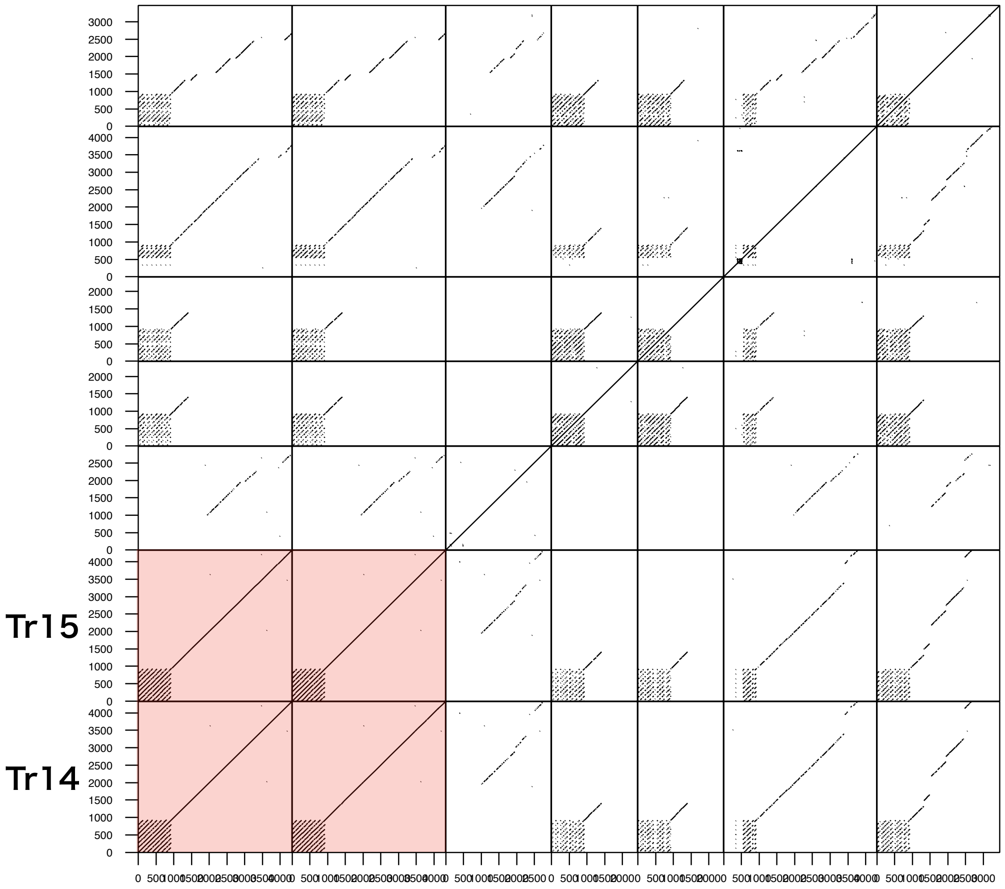

No.	Length	Lines	Points	Sequence
1	4328	1096	36242	scaffold_51_230432-234759C
2	4325	1096	36580	Chr01_83497100-83501424
3	2974	187	6687	Chr01_96615127-96618100C
4	2425	998	25070	Chr04_54730435-54732859C
5	2412	1013	24605	Chr04_55473190-55475601
6	4310	990	24516	Chr09_25212862-25217171
7	3461	1163	29944	Chr09_41012139-41015599C

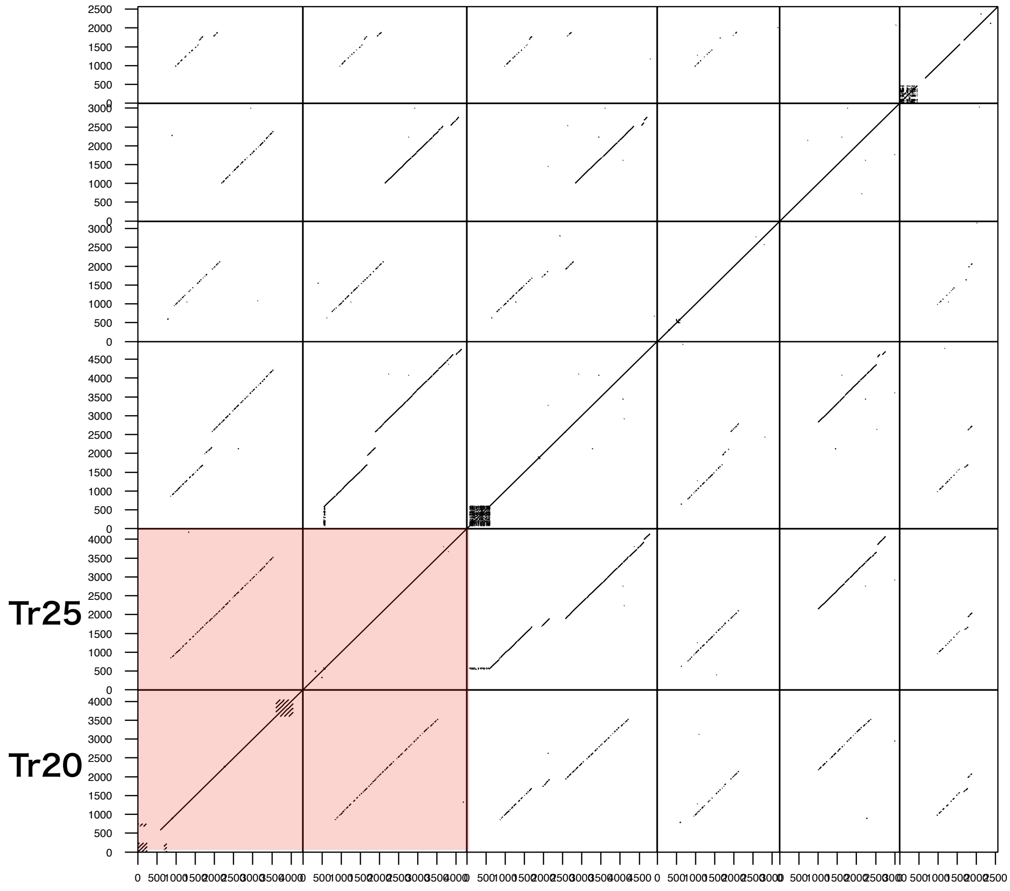

No.	Length	Lines	Points	Sequence
1	4298	294	11355	scaffold_16_1255554-1259851
2	4274	260	12769	Chr01_107511548-107515821
3	4962	581	19783	Chr05_65761707-65766668
4	3190	153	5908	Chr06_41655045-41658234C
5	3120	118	7385	scaffold_470_13449-16568
6	2562	225	6529	scaffold_53_169162-171723

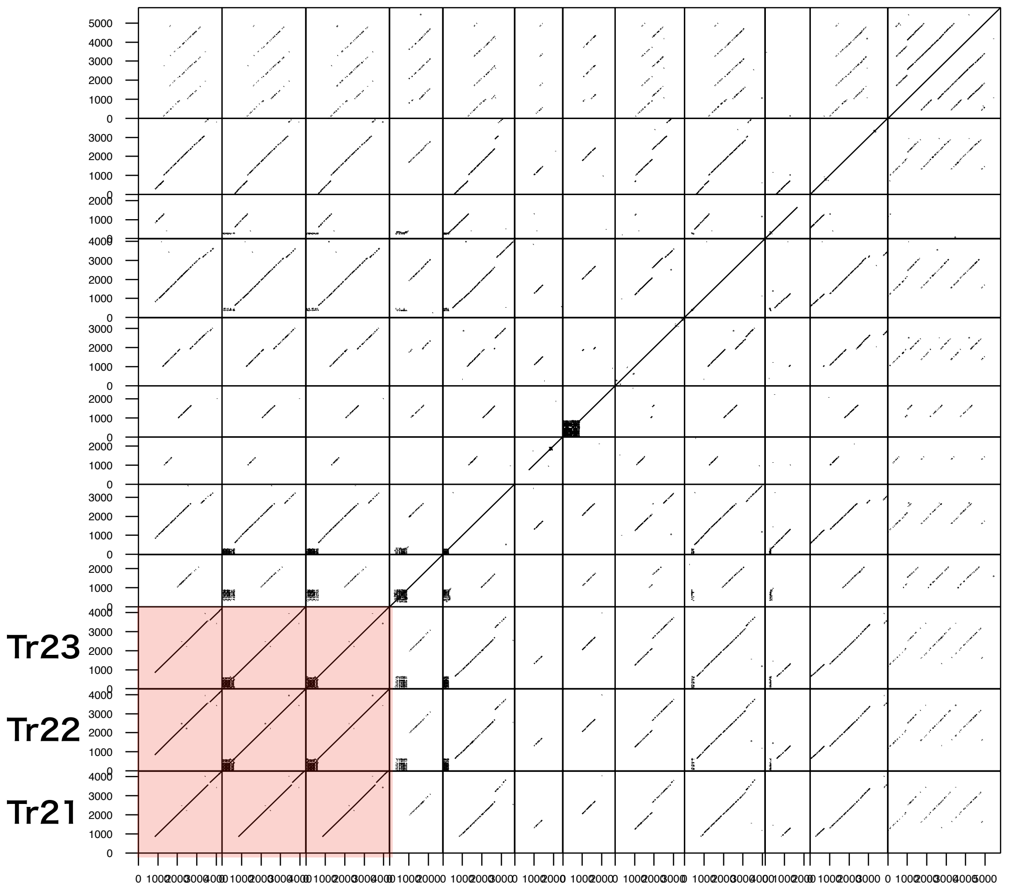

No.	Length	Lines	Points	Sequence
1	4295	496	20604	Chr04_107656217-107660511
2	4295	1332	38623	Chr01_168454606-168458900C
3	4295	1348	39019	Chr03_106167762-106172056C
4	2738	957	19021	Chr01_86117569-86120306C
5	3678	1079	28671	Chr02_109157450-109161127
6	2465	146	5690	Chr04_62227445-62229909
7	2686	843	25998	Chr04_62231301-62233986
8	3564	449	15429	Chr05_54048027-54051590
9	4128	793	24265	Chr05_54184581-54188708C
10	2308	263	7977	Chr06_24802786-24805093C
11	3987	656	21140	Chr09_25905917-25909903C
12	5796	1037	27278	Chr09_28311164-28316959

Tr27

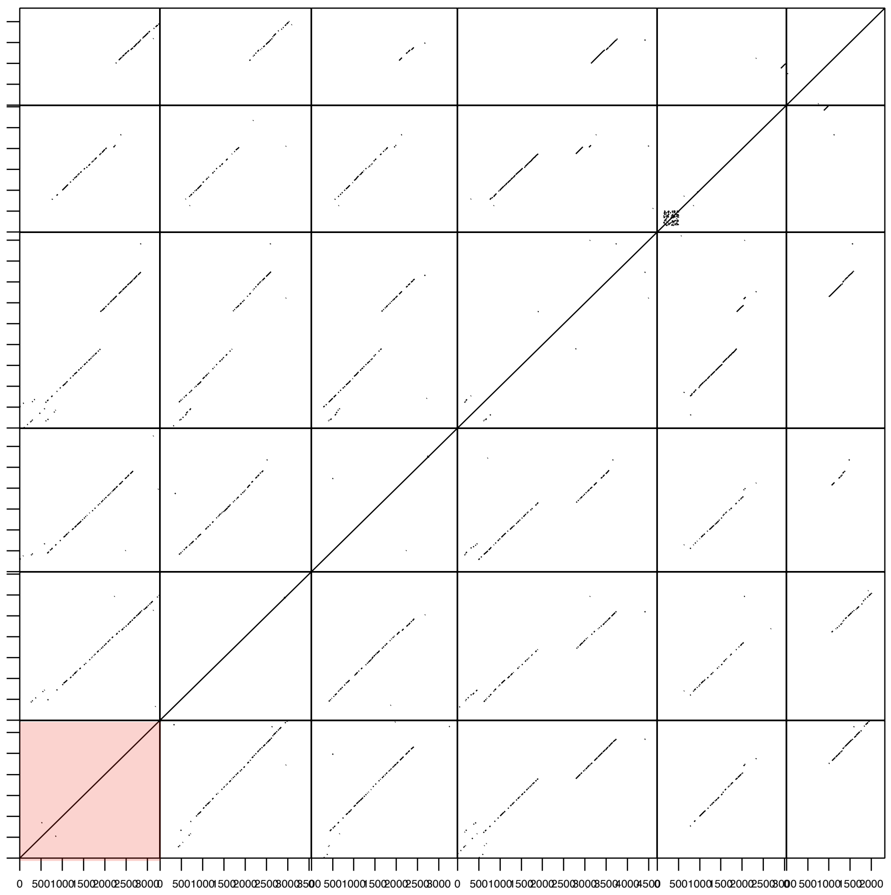

No.	Length	Lines	Points	Sequence
1	3292	258	8771	scaffold_4830_1-3292C
2	3546	257	8250	Chr01_45228745-45232290
3	3427	229	7980	Chr01_86084092-86087518
4	4687	275	10974	Chr04_69163503-69168189
5	3031	225	7781	Chr06_126862332-126865362
6	2314	84	4461	Chr06_126866557-126868870
