# Supplementary figures and images for: Multiple massive domestication and recent amplification of Kolobok superfamily transposons in the clawed frog Xenopus
Source: Zoological Lett. 2018 Jun 16;4:17. doi: 10.1186/s40851-018-0100-4 (PMC6004289; doi:10.1186/s40851-018-0100-4)

D1

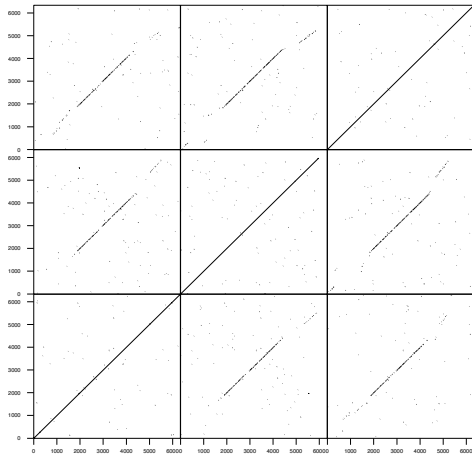

D2

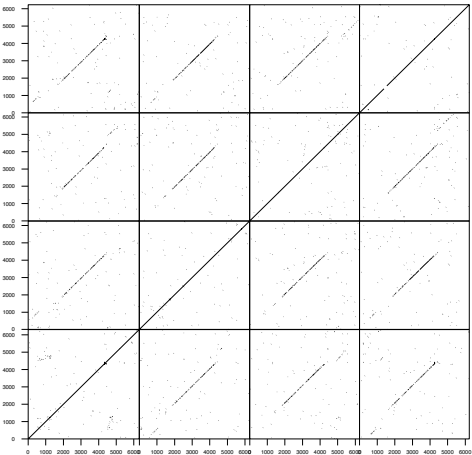

D3

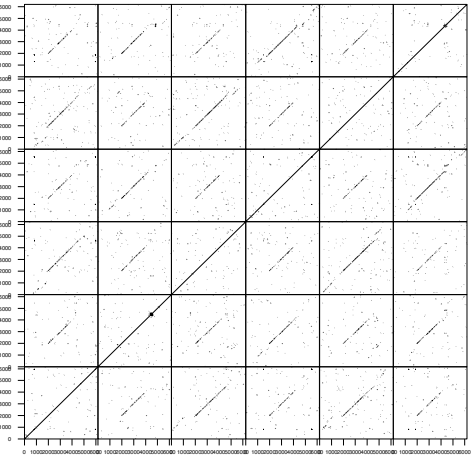

D4

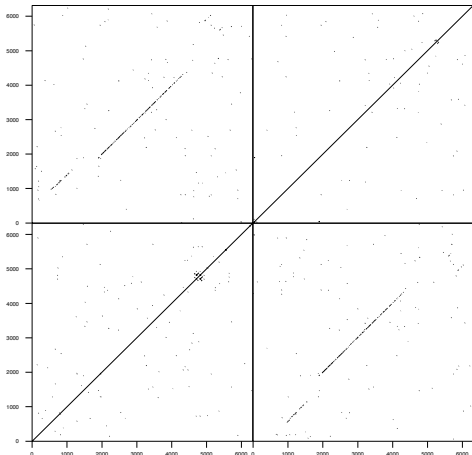

D5

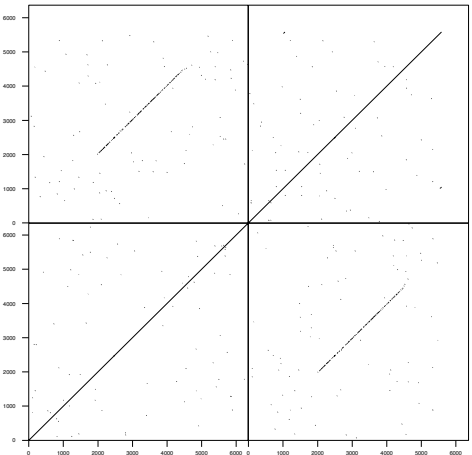

D6

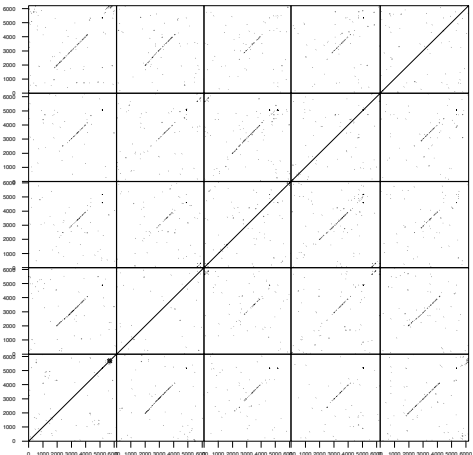

D7

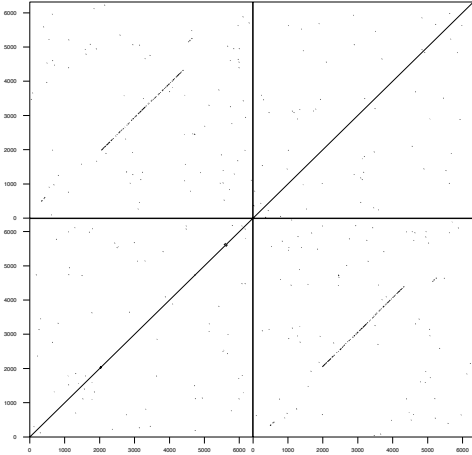

D8

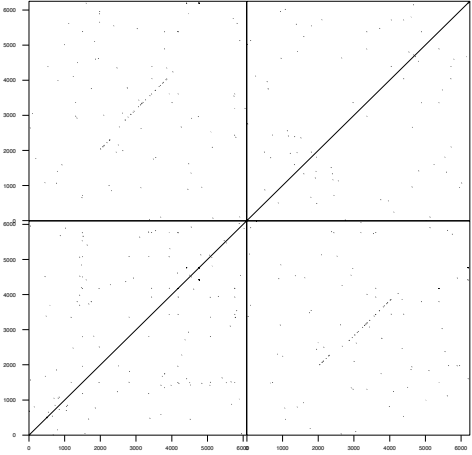

D9

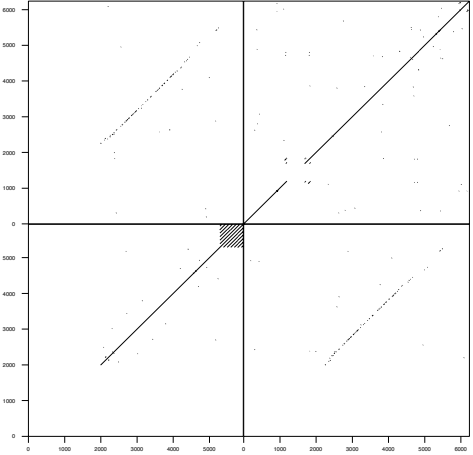

Supplement: Supplementary file 3 — Figure S3. XKol-Tpase CDSs and flanking upstream and downstream 2000 bp sequences of subfamily D1–D9 were compared by dot plot analyses (word size = 10). (PDF 319 kb) [file 40851_2018_100_MOESM3_ESM.pdf]
