# Supplementary material for: Multiple massive domestication and recent amplification of Kolobok superfamily transposons in the clawed frog Xenopus
Source: Zoological Lett. 2018 Jun 16;4:17. doi: 10.1186/s40851-018-0100-4 (PMC6004289; doi:10.1186/s40851-018-0100-4)
Supplement: Supplementary file 4 — Figure S4. Multiple alignment of prospective transposase CDSs from the X. tropicalis and X. laevis genome databases and from CDSs cloned from X. tropicalis Nigerian and Asashima lineages. (PDF 167 kb) [file 40851_2018_100_MOESM4_ESM.pdf]

```
Tr20_scaffold_16|4  ATGCCTAAGTGTATTGTTAATGGATGTCCACACAGGAGTGGCCAAAAACTGCTTTACCCGGATGTTGTTCTACATCCCTTTCCAAAAAATATTGAAAGGATTCGAAACTGGCTCTTGCAAACAGGCCAGCACTCGGAAGACCTCGAACTA
Tr25_Chr01|4        ...GA.------G.CCAC.CA...GTGG.CA.A.AC.AC------------------............T................................A..........GA...................A........T....A.
Kol_01_Nig          ......................................................................................................................................................
Kol_01_Asa          .....................................................................T...............................................................T.........T......

Tr20_scaffold_16|4  CTTACCCAAAGAATATTTCAAGGATGGAAAACTGCAAACTTCAGAATGTGTTCGAAACACTTTACTGAGGGTTCCTACATGCAAAGCGGATCACGAAGAATTTTAAAACCAAATGCAGTACCAACAATTTTTGATACTGTCCGTGTTCCG
Tr25_Chr01|4        ....G............AG..................................T.....T..........A...........T..............T......................................T...AAA......A
Kol_01_Nig          ......................................................................................................................................................
Kol_01_Asa          .....................................................................................................................................................A

Tr20_scaffold_16|4  ATTACAACTTTATTCACAGTCGCCCCCTCTCTGCCAATAGTCGCTTCTGCTAAACGAAGACGAGTTGACGATGAACAGCCATCGACTGCTTCGACCATCGTAAGAGTCATTTCGCGTCTTGTTACTGTTGGAACACAGACCGATGCTAAA
Tr25_Chr01|4        G........G.GA........C........G..........A..................A......................A...T.....TA.CT.........A.......A.....C..................T.........
Kol_01_Nig          ......................................................................................................................................................
Kol_01_Asa          ...G...............A....................AA..........................................................A.................................................

Tr20_scaffold_16|4  GTGAATATGGTCAATACTTCGACCATGACGTGCACTAGATGTAGAAATTGCTCTACACAAACAAAAAAAATTCTTACTAAGGAAACGAGTTCTCAAAGTGGAGAAGAATTCATCCAGGCAGATGTTCATCACATTCTCCAAGATCACCTA
Tr25_Chr01|4        ..........C.........A........A.......C....CA...C...................G...AA......T.....T.G...............................T...........A....AA............
Kol_01_Nig          ......................................................................................................................................................
Kol_01_Asa          ...................................................................................................................................T....A.............

Tr20_scaffold_16|4  TATCCTGTGTGTTTTTCAACTCGATCCAAACCAACCAGCAAAATAAGTGATCTGCAATCGACAATGCATTCAACATCAGCACCAATGGAGTCCTTCATTTCACCTATTGGAGGGGAACCTTCTACCAGCATATTAGAACCATTTGTTCAG
Tr25_Chr01|4        ..........T...........C....................................A.....T.............................A............A....A...A.C...GG....T........T...........
Kol_01_Nig          ......................................................................................................................................................
Kol_01_Asa          ...........................................................A.......TA........................................A.G.................T........T...A.......

Tr20_scaffold_16|4  TCTTCTTTTGGTTCTATAGCCGATGGAGATTCTACATTTGATATTCAAGAACTATCATCAGGTGGATATAGTACTGACAGTGATTCATTTGAAACCGAGGAGGAACGGACAGTTGTACCTGAAGTCATAAAATCCAAAAAGAAATTTATT
Tr25_Chr01|4        ......CCATCGGT.T...AACCAAA...............................A.C......A............A.A..---------.........A...A..................A.............GA.........
Kol_01_Nig          ......................................................................................................................................................
Kol_01_Asa          ..................A.......................C...............................................C...............A...C.......................A...............

Tr20_scaffold_16|4  ATTTATGAAGATAAACTGGATGATCTCTTGCATGTAGTGCGCTGCCAACACAATATTGACCCACCGTGCCAGTCTCCAATAGTTGAGGTAGAAAAAAAATTGGATGGCAGTATGTTGAGTGTTCGGCTAACGTGTTTAGATGGTCAT---
Tr25_Chr01|4        ..........................A---------.................A...........T......G.................A.......T.......................G....C...A...............AAT
Kol_01_Nig          ......................................................................................................................................................
Kol_01_Asa          ...............A.............................................................................................................A........................

Tr20_scaffold_16|4  TCAATGCTATGGAACTCACAGCCCACCTTTGGTGAAACGTCAATAGGAAATCTGGAAATTACTGGCTCTATTATCCTTACTGGAGCAACTTATCAAAAAACGTATGATATTTTCAAGTTGTTGAGTATCCCATTTATATCACATACAATG
Tr25_Chr01|4        ...C.............................A....T..T...........A.............T.G.G............................T.........G..T..........A.......................CC
Kol_01_Nig          ......................................................................................................................................................
Kol_01_Asa          ......................................T.....................................................................................A.........................

Tr20_scaffold_16|4  TTTTATAACTATCAGAGAAACTATGTCTTCCCTGCAATTGATCATCAATGGAAAAAGGAAAAGGACGTCATGGCAAGGAAA---------AGGACGCCAATGGCATTGGCAGGTGATGGCCAATTTGATAGCCCTGGTCATTCGGCTAAA
Tr25_Chr01|4        ........A........................................................T.A.T..---..A...AATTTGTTG.AA.AA...............................................T......
Kol_01_Nig          ......................................................................................................................................................
Kol_01_Asa          ......................................................................................................................................................

Tr20_scaffold_16|4  TTTGTACATATACCATGATGGACGTAACATCGAAGAAAATCATCGACTTCAAGATTGAACAGCTTGGGTCTGGAAAAACTGCCGGACAAACGGAGTGTGAGGCTTTTGACAAATGTCTACACAATCTAGTC------GAAAAAGTAAAA
Tr25_Chr01|4        .......................T....T...A...........T.........................T..........T.T...G...T.........A....G..T............T.......TATAAAG.............
Kol_01_Nig          ......................................................................................................................................................
Kol_01_Asa          ........................................................................................................................T.............................

Tr20_scaffold_16|4  GTTAAAGTTGTTGCGACGGATCGACGCGGCTCCATACGGAAATTAATGGCAACGAAATATAAAAAAATTGATCACCAGTTCGACGTGTGGCGCTTGTGCAAGAGTTTACACAGGAAGCTTCTTGCTGCTAGCAAAAAAAGAAAATGTAAA
Tr25_Chr01|4        ......A.......A..A.......A.T.........................A.......................A.....T.......A....................A.......................C.............
Kol_01_Nig          ......................................................................................................................................................
Kol_01_Asa          ................................................................................T............................................C........................

Tr20_scaffold_16|4  GATATATCCCGCTGGATCAGCGCCATAACCAATCATCTCTGGTGGAGCGCACAAACCTGCGATCAAAACGTAGATCTTCTACTTGATAAATGGAAGTCGGTTTTGTACCATGTGGCAAACGAACATTCCTTCGGGGCATTAAAACACTAT
Tr25_Chr01|4        .....CG..........T..T........A........A........T............A.....................................A.....................A.G........A.A...C......A.....
Kol_01_Nig          ......................................................................................................................................................
Kol_01_Asa          ......................................................................................................................................................

Tr20_scaffold_16|4  CAAAAATGTCAGCACCGGCGCTTAACACCCGAGGAACAGAAACAAACTTGTTGGATTGGTGCCAATCACCCGGCACATTCTGTCCTTGCTGAAATCATTAACGATACTAAATTTCTGAACGATTTATCACACATCGGAAAATTCTGTCAC
Tr25_Chr01|4        A.C..........................T...C..A....G...G............A............A........A..............A.....T...A..GG.........A..........TG....A.............
Kol_01_Nig          ......................................................................................................................................................
Kol_01_Asa          ....................................................................................................................................G............A....

Tr20_scaffold_16|4  ACTGGTGATTTGGAGAACTTTCACAGTAAGGTCTCAAGGTACAAACCAAAAAGATTGTCATTTCCAATGGACTCTGTGATAGCTCGTACCACCCTAGCGGCATTATCACACAACCGCAACGTCAAAAGAAAACAGGGTTCTGTCCAGTGT
Tr25_Chr01|4        .......................T........A.TGC...........................G..A.......A...........................................T.....C..........C.......A.....
Kol_01_Nig          ......................................................................................................................................................
Kol_01_Asa          ...........................................................................................................G.................C.......G..C........C....

Tr20_scaffold_16|4  CCGGCAAAGTCTACATTATCGTTTGGACAGAACCGTGACCAAATAGTTTTCCCAAAACTGAAAAAGGACTGGGCTGCGAAACCAGTTTTTGAAGGAGGAGTTGATGATCATTTGTTTGAAATACTCCAAGACTGCTTAAAGATTTTGACT
Tr25_Chr01|4        ..T.............CC.A............A..CTT.A.......C..........A..............T..........A....C.......TT..............A....................T...............
Kol_01_Nig          ......................................................................................................................................................
Kol_01_Asa          ..T...........G..............A.....C...........C.........................TG......................TT...................................T...............

Tr20_scaffold_16|4  GGGGAGTTACAACATGATTGGTCTTCAACTGCCCGTGAAATGCCCAACAATTATAGCTACTGT---TGA
Tr25_Chr01|4        .....A......................................G..A...ATAGCTACTGT.GAA...
Kol_01_Nig          .....................................................................
Kol_01_Asa          ......C....................T......A......................A...........
```

```
Tr18_Chr02|0        ATGCCGTATTGCATCGTGAAAGGGTGTCCTCACCGAACTGGGCAAAAACTGAAATATCCTGATGTGGTTCTACATCCATTCCCACGTGATATTCATCTAATTAAAAAATGGTTAATGCAAACCGGCCAACATAACCAAGGTATTGATACC
Tr37_Chr01|0        ------------------------------------------------------------------------------------------------------------AT....------------------------------------
Kol_02_Nig          ......................................................................................................................................................
Kol_02_Asa          ..........................................................................................................................T...........................

Tr18_Chr02|0        TTATCCGAAAGGATTTTTCAAGGTACCAAAAACAATGCTTTCAGGATGTGTTCTACACATTTCACACCTGACTCTTACATGATGAGGGGTTCTAGAAGACTTCTATCACCAAAAGCGGTTCCCACCGTTTTTGCAAGTATACCAACTCCA
Tr37_Chr01|0        ---------------------------------------------------------------------------...------.AA..C------------------------------------------------------------
Kol_02_Nig          ......................................................................................................................................................
Kol_02_Asa          ......................................................................................................................................................

Tr18_Chr02|0        GCTATTATTAATGTTCCATTGGAAACAACTCTGCCTTCCCAAAAAAGGAGAAGAGTCGATGACGAGGAAGCATCCACCTCCACACGACTGGTCAGAATTGTGTCACATCTTATCACTGTAGCAACACAAACTGAATCTTATACCAAGACT
Tr37_Chr01|0        ------------------------------------------------------------------------------------------------------------------------------------------------------
Kol_02_Nig          ......................................................................................................................................................
Kol_02_Asa          ......................................................................................................................................................

Tr18_Chr02|0        TGCTCAACAATGACATCAAGAAGGGTGTTTTCTCGCCAAGCAACAATGCAAACAACTGGAAATGGAGGAAAAGTAGTTTCTACACAAACGGAAGATATATATCAAGCTGATGTTATGCAA------GTTAGAAAGGATCATACAGTTTGT
Tr37_Chr01|0        ---......T.........................T.......GT.C......G....C............C...................................A.T.TG.T.TTCTACACCTT.A.A..CTACA..GC.....---
Kol_02_Nig          ......................................................................................................................................................
Kol_02_Asa          ..................................A...................................................................................................................

Tr18_Chr02|0        TTTTATTCTGATTTATTATCTGCATCTCCCATTTCACCTGCCTTACAATCTCTGGAGAACCCAGAAACACCATTGAAAGGTCGGGGTTCACTCATTAAACACCCATCCTCAATATTTTCATCACCTACAGTAGCAGAACCTAGAGATACT
Tr37_Chr01|0        ------A.....A.......................A.AA............A..........................A..A..............C..................................A.................
Kol_02_Nig          ......................................................................................................................................................
Kol_02_Asa          ...............................................................................A..........................G.........................A.................

Tr18_Chr02|0        ACTTTTGAAATATCGGAACCATCATCTTCTGAAAATTCAAAAGAAGAACAAGAAACTACGGAAAGTGGGCAATCCAAGTATTTGAGAGCGAGAAAATTTATAATCTTTGAGGACAATTTAGATGCCCTACTACATCTTGTCAGATGCCAG
Tr37_Chr01|0        ..............T....................A..G.....................A.........C............C...............C..........A..........................G............
Kol_02_Nig          ......................................................................................................................................................
Kol_02_Asa          .........................................T...............................................A....................A.......................................

Tr18_Chr02|0        CATGCAGTTAAACCACCATGTCAGGCACCTATAGTAGAAATTAAAAAAAGTACTGACGGAAGCTTACTCAAGGTTCAGTTGTGTTGTATAAATGGACATACTTCTTTGAAATGGTTTTCACAGCCCGTGGTGGATGAAACATCTATTGGA
Tr37_Chr01|0        ........A....................................................................A............G..................................T........CC..............
Kol_02_Nig          ......................................................................................................................................................
Kol_02_Asa          ......................................................................................................................................................

Tr18_Chr02|0        AATGTAGCTCTGGCTAATGCGATCATTCTCAGTGGTTCAACATATAGAAAAGTGAGAGATATGTTCACCATTCTCAATGTACCCTTTTATTCCCACACCAGCTTTTACTGCTATCAACGAAAATTTGTTTATCCAGCAATAGATATACAG
Tr37_Chr01|0        .................C..T...........G..................C..........C................................T...CG.......CA........................................
Kol_02_Nig          ......................................................................................................................................................
Kol_02_Asa          ....................T...............................................................................C.................................................

Tr18_Chr02|0        TGGAAAAAAGAACAGGCTGCTATAAAGGAATCCTTATCAGGACAATCCGTCACCTTAGCAGGCGACGGACAATATGATAGTCCTAGACATGCAGCCAAATACTGTACTTATTCAATGATGGACCTCATGTCCAAAAAAAATTGTCCATTTT
Tr37_Chr01|0        .........................T.A....G..............A...G....G........T..................G...T.T..........T.....A.................T........................
Kol_02_Nig          ......................................................................................................................................................
Kol_02_Asa          ...............................................T...G..................................................................................................

Tr18_Chr02|0        GAAATTGACCAGGTCGGTCCTGGCAAAAATGCAAACAAACTGGAACCATTAAACTTTGAGAAAGCATTGAACTGGTTGATCCATGAAAACTTCGATGTTAAAATAGTTGCTACAGACAGGCACAGCGTGATCAAAAATATCATGTCAAGT
Tr37_Chr01|0        ........................................................................................AG.T.........G................................................
Kol_02_Nig          ......................................................................................................................................................
Kol_02_Asa          ......................................................................................................................................................

Tr18_Chr02|0        AAATTTGAAAATATAGACCACCAATTTGATGTGTGGCATCTATGCAAGAGCATACATAAGAAATTATTGGCAGCAAGCAAAAAAAGGAATTGCAGTGACATTGCATCCTGGATCACTCCCATTACAAATCATCTGTGGTGGTCGTCTCAA
Tr37_Chr01|0        .C....A..C...............................G.................A.................................C..................G............C......T..........A..A...
Kol_02_Nig          ......................................................................................................................................................
Kol_02_Asa          ......................................................................................................................................................

Tr18_Chr02|0        ACCTGCAAGCAAAATGTAAATGTTCTGATCGACAAATGGAAATCATTACTATTCCATATTGCCAACAAGCACACTTTTAAATCCCTACAATACTATCAAAAATGCCAGCATCAACGGCTGACTGCTGCACAAAAAAAACATACTAGATGG
Tr37_Chr01|0        ..............C..C....................................T.......A.......................G.G.....A.................G............A.....G......T...TC..G...
Kol_02_Nig          ......................................................................................................................................................
Kol_02_Asa          ..............................................................................................................................A.......................

Tr18_Chr02|0        ATTGGTTCAAATCATCCTGCTTACAACAGTCTCAAACTTATTGTTAATGACACT------TTACGTGATATTCGTAAAATTGAGAAATTCTGTCATACCAGTGACTTAGAAAATTTCCACAGTAAAGTACTGAAATACAAGCCCAAAAGG
Tr37_Chr01|0        ....................AC..................A.A.........A.TCACTG....T.....G.....................C..C........T..............T..C....G.........T........G..A
Kol_02_Nig          ......................................................................................................................................................
Kol_02_Asa          .....................................................C.....................................................C..........................................

Tr18_Chr02|0        TTAAGTTTCAAAATGGATGGGATGATTGCAAGAACAGCACTAGGGGCTTTGTCACACAACAGAAATGTCCACAGACAACAGGCTGTTGTCACATCTGCCAAAAAATCAACATTGCCTGTAGGAAAAAAAAAGATTTAAAATTGTTTTTCCA
Tr37_Chr01|0        ...T.C.......................C.............C.........................A...........T.......A.A..................C...........C......C...................T
Kol_02_Nig          ......................................................................................................................................................
Kol_02_Asa          ......................................................................................................................................................

Tr18_Chr02|0        AAGCAAAAAGAACAATGGGTAGCCAAACCAATGTATGAGGAAGTATTGGATGAACACCTTTTTGAGATAATGTCAGACTCAATAAAAAATTTTGACTGGTGAGATCAGCCATGAATGGCTTTCTATGTCACAAAGGATGCCCAAAAATATT
Tr37_Chr01|0        .........A......................A.......C.C.T.......................C........T...........C................C......................A........G...........
Kol_02_Nig          ......................................................................................................................................................
Kol_02_Asa          ......................................................................................................................................................

Tr18_Chr02|0        GCCACTTATGAAAGACCAGAAAAAACTGAAGTCATTGACCAACATTCGTCATGCTTTGCAAGACCCTGA
Tr37_Chr01|0        ..T....G...........C....T.......A.....G.G.T...T....C.A....A..A.T.....
Kol_02_Nig          .....................................................................
Kol_02_Asa          .....................................................................
```

```
Tr24_scaffold_561|0  ATGCCGAGTTGCATAGTAAAAGGCTGCATTCACAAGAGCGGCCAGAAGGCGTTACACCCGGATGTGGTCATGCACCCCTTCCCGCACAATCGGGAGCAGATCAAGAACTGGGTGCTGCAGACCGGTCAGTACACTGAGGATCTCGAGACC
Kol_03_Nig           ......................................................................................................................................................
Kol_03_Asa           .............................A.................................................................A......................................................

Tr24_scaffold_561|0  ATGACTGACAGAATCGTCAAAGGCCTAAAACATTCCAGTTTCCGAATGTGCTCGAAACACTTTACAGAAAACTGTTACATGATGAAAAACACCAAAAAGGTCCTGAAACCGAACGCCGTGCCGACTATATTTCCCACGATCCCGAGGCCG
Kol_03_Nig           ....................G....................................T............................................................................................
Kol_03_Asa           ....................G.....................................................................................................................T...........

Tr24_scaffold_561|0  ACGGTAATCACCGCCCAACCTTTACCCGTACTGTTACCCCCTGCCAAACGGCAACGGGTAGACAACAACCACGAAGAA---CAACAACCGTCGACTTCGACGACCATCGTACGGATAGTGTCCCGACTTGCGACCGTTTCGACGCAAACA
Kol_03_Nig           ......................................................................................................................................................
Kol_03_Asa           .......................G......................................................CAA.................A.........................................T.........

Tr24_scaffold_561|0  AAAGCGAGAATGTTCGCTAATTACAACGTGGAAACGATGGATATTTATAAGAACGTCGGGATACAGAATACGGTGGTTATGAAGGACATGTCCACGCAAACGCACGACGTGGCACCTACAGACGTTCCCAGCACCACCAAGGACCGCCCA
Kol_03_Nig           ......................................................................................................................................................
Kol_03_Asa           .............................A.............................................................................T..........................................

Tr24_scaffold_561|0  TATCCGGTGTTCTACCCCGTTCTAGTCAAACCCGAATCCATGCCAGGCATGAATAAAATAACTTCGATTTTACAATCCCCTTCTGTTGCCATTCAGTTTGTTCCGCCAACCAGATCCGCCCTTGTGGGAAATGGTGAAAGATCCTCGGTC
Kol_03_Nig           ...........................................................................................................................A..........................
Kol_03_Asa           .......................................................................................T.T.....A...........................A..........................

Tr24_scaffold_561|0  GAAGATCGAAGAGACGAGGCGACTTTTAAAATTCAAGAACGGGCTGGAAGTCGAGATGAGGGAACGACGACGGGGAACGGCGATTATATCAAGCAGAGAAAGTTCATCGTATACGAAGACCAACTGGATCTACTACTGCGTCTTGTTCGT
Kol_03_Nig           ......................................................................................................................................................
Kol_03_Asa           .....................................................................G................................................................................

Tr24_scaffold_561|0  TGCCAGCATAGGTCGGATCCTCCCTGCCAAGCCCCCATTACACGTATTAAAAAGCGCACCGACGGCAGTCTCTTAAACATACAACTCTGCTGTCTCAACGGACACGACTCTCTCCTGTGGAATTCTCAACCCACGTCGGGAGAGTTCTCG
Kol_03_Nig           ......................................................................................................................................................
Kol_03_Asa           ........................................................T.............................................................................................

Tr24_scaffold_561|0  ATCGGAAACGTGGCTCTCGCCAATTCCATGGTTCTGGTGGGATCGACCTATCAAAGGATGAAAGAGTTTTTTCAGCTTATGAACATTCCGTTGTTTTCTCGTGCCGCGTTTTACAAATACCAAAAAGAGTTCATTTTCCCGGCCATAGAC
Kol_03_Nig           ...........T..............G..............................................................A............................................C...T...........
Kol_03_Asa           ...........T..............G.......................C..........G........................................................................................

Tr24_scaffold_561|0  ATTCAGTGGAAAAAAGAACGCGCGGAGATCAAACAATCATTGGCCGGGAAAGCCGCGGCGTTAGCTGGCGACGGGCAGTTCGACTGCCCCAGTCACAGCGCTACGTATTGCACGTATTCCATGATGGAGCTGATGTCCAAAAAGATTATC
Kol_03_Nig           .......................................................T............................C.................................................................
Kol_03_Asa           ....................................................................................C.................................................................

Tr24_scaffold_561|0  GATTTCAAAATTCGACGGGTCAGATTTGGAAAAAAGTCAGACGAAACGGAAACCTCCTCTTTATATTCGTGTTTGAAGAGTCTAAAGGACGAAAAGATAAACGTCGGAATGGTGGCGACCGACAGACACGACGGGATAAAGAAACTGATG
Kol_03_Nig           .................T...G..CC............C..T.G..T.....T.---..G...C.......CC....A...........................A....C.......................................
Kol_03_Asa           .....................G..CC............C....G..T.....T.C....G...C......................................................................................

Tr24_scaffold_561|0  CGTACCGAGTTCGAGGGCGTCGACCACCAGTTCGACGTCCGGCACATTTGCAGAAGCCTAAGGAAAAAATTACTCGCTGCCTCTAGAAAGAAAAACTGCGGCGAGGTCGCATTTTGGATTAGGCCTATAACGAACCACATGTGGTGGGCG
Kol_03_Nig           .....GA...................................................................T..C..........................C.....G.......................................
Kol_03_Asa           .....GA.............................................A........................C.....C....G...............C..T..G....................................T..

Tr24_scaffold_561|0  GCGCAAACGTGTCGACAAAACGCGGATCTTCTCGTCGAAAAGTGGAAATCGGTTCTGTTTCATATATCGAACGAACACGCGTTCCCTTCGTTTGTACATTATAAAAAATGCCAGCACCGGCGGATAATGGCAGCCCAAAGGAGGAGTTAT
Kol_03_Nig           .............................................................................T.....................................................G..................
Kol_03_Asa           ............AA.G..............................................C........................................................A...T..........................

Tr24_scaffold_561|0  AGATGGATCGGCCCCGCCCACCCTGCTCACTCCGCCTTACGGAAAATCATAATGGATCCGTTATTGTTACGCGACCTACGCAAAGTAGGGAAGTTCTGCCATACGGGCGAACTAGAAAACTTCCGTGGCAAACTCATAAAGTACAGACCT
Kol_03_Nig           ........A.......................................................................................................................C........................
Kol_03_Asa           ........A.....................................................................T........................T...G.................C........T...............

Tr24_scaffold_561|0  AAGTGCCTGTCCTTCAATAGGGACTCGGTGATTGCCAGGACCTGTCTCGGCATTTTGGCGCATAATAGGAACGTTCACCGACAGGAAGTTGTAAAAAAACCGTTGAGTCTCGACAAGCCGATTTACGCCGCCGTCGTTGACGATCATCTT
Kol_03_Nig           ............................C......................G....A..........................................AT.GC........C......A.......A...T.....C............
Kol_03_Asa           ............................C......................G.A.............................................A...C........C..............A.........C............

Tr24_scaffold_561|0  TTTGATATTCTATCGGACTCGATTGACATTCTGACAGGGAAACTGACTCACCGGTGGCCGTCGGAGACCAAATTCGTGCCCAAAAATATAGCGACATCGGATAGGCCAATCAAAAGCGAGGTCATAGCCAATCATGTTTATCAGTTCTCA
Kol_03_Nig           ...........G.............................................................C........................C........................................T.........G
Kol_03_Asa           ...........G.............................................................C.......C............T...C........................................G..........

Tr24_scaffold_561|0  CATTCCTAG
Kol_03_Nig           .........
Kol_03_Asa           TG.......
```

```
Tr28_Chr04|0      ATGCCAAATTGCATAGTGAAGGGTTGTCGCCACAAAAGTGGACAAAAGATCCAATACCCAGATGTCGTCCTTCACCCTTTTCCAAATAATATAAATCTGATAAAGAACTGGCTGTTACAGACTGGTCAGGACTTGGGGGATATTGATGTC
Kol_04_Nig        ..................................................................................................................................................................
Kol_04_Asa        ..................................................................................................................................................................
Kol_05_Nig        .....C.G...........A.....................G.......TG..T........T..T.....TT.................C..A..............A.......A.............T............C.
Kol_05_Asa        .....C.G...........A.....................G.......TG..T........T..T.....TT.................C..A..............A.......A.............T............C.
Lv14_chr4L|0      .....C.G...........GA..............T.....C.....G...G..TG..T...G..............T..............C..A..............A.......A.............TT..T..........C.
Lv20_chr4S|0      ..........................................................................................T.A........G......................................T.............G
Lv21_chr4S|0      ......G.....C.....A.......................G..G.T.TG..T........T.........T.................C..A...............................TT..C............CT
Lv22_chr4L|0      ...................A.........................T......................................C.....A......................................T..A...........

Tr28_Chr04|0      CTTGCAGATAAAATTCTAAAAGGAAAGAAAACAGCAAATTACCGAATGTGCTCGTGTCACTTTACAAGGATAGCTATATGGCTAGGGGTTCGAAAACCACCTTAAAACCAAATGCCGTCCCAACAATTTTTCCAATAATACTGCCACCT
Kol_04_Nig        ..................................................................................................................................................................
Kol_04_Asa        ..................................................................................................................................................................
Kol_05_Nig        T.......G........G........T.....C..C.GC.T...T........AC.............A................A...C..C....T..................T..T............GAT.C.---T.A.....G
Kol_05_Asa        T.......G........G........T.....C..C.GC.T...T........AC.............A................A...C..C....T..................T..T............GAT.C.---T.A.....G
Lv14_chr4L|0      T.......A...G............T......T.C.GC.T...........AA........G....A...T...........A...C..C....T..................T..T......C..C...GAT.C.---T.......A
Lv20_chr4S|0      .................G.................C..C.T.............A.....................A...........A.....A..................T.....G...........TG.T---A.AT.G..A
Lv21_chr4S|0      T.......A...G............T......T.CGCC.T...........AC.....T.........A................A...C..A....T..................T..T........C...AAT.CT---T.A.....A
Lv22_chr4L|0      .................G...............G.C.....C.T.............A..C......................A.....G.................TA.......C.......TG..........A..

Tr28_Chr04|0      GCTGTT---GTTTGCAGTCAGATATCATTACCGCCAGCTAAGAGACTGAGAGTAGAAGATGAAGTACCATCTACCTCTGCCGCTGTTGTACGTATAGTTTCAAAACTCGTCACCGTGCATACGCAAACTGATGATAGGGTTTTCCGAAGA
Kol_04_Nig        ..................................................................................................................................................................
Kol_04_Asa        .T................................................................................................................................................................
Kol_05_Nig        ....C.TCT...CCT....T.........G..AA...........A.................C................A..A..................T.....T.....A..A..A..A...........C..AA..C..AA..AT
Kol_05_Asa        ....C.TCT...CCT....T.........G..AA...........A.................C................A..A......................T..A..A..A..A...........C..AA..C..AA..AT
Lv14_chr4L|0      ....C.GCT...CCT....T...G.....G..AA...........G........................G..T....T.A..A..........A.........T.....A..A..A..A.....G.........AA..C..AA..AT
Lv20_chr4S|0      A..CC.......CAT.........C........AA....A.....................C...................TA.............G...........A.............A.....C..............T.A....
Lv21_chr4S|0      ....C.GCT...CCT....T...G.....G..AA.........................C.A........A.........A..A...........CA.........T.....A..A..A..C.....C.........A..C..AA..AT
Lv22_chr4L|0      A..---......CCT..CTC....---..G..AA....TG.........A.......C....CC.....C.......TA..........C......T...........A................A...........C.A........A....

Tr28_Chr04|0      GACGCTTCAGTCAACACATACAACTGGCCAATGCAAGTAGATGTTGCTACACAGACTGATCCACAAGTAGAGACAAAGGTTTTAAAAACTGAAACTAAAAATGAATCTCAAGAAGTAAAGGGTTGGGCTGCTGAGATGGACCATTTGTAC
Kol_04_Nig        ..................................................................................................................................................................
Kol_04_Asa        ..................................................................................................................................................................
Kol_05_Nig        ...C.C.GG.....T.G.........C...G..ACT...CAGC........G......C...G.T.C..C.......CG.C.A.A.G.............GC.GCAG.........G.......AT....CA.......T.AA....T.......T
Kol_05_Asa        ...C.C.GG.....T.G.............G..ACT...AGC.........G......C...G.T.C..C.......G.C.A.A.G.............GC.GCAG.........G.......AT....CA.......T.AA....T.......T
Lv14_chr4L|0      ...TA..GG.....G.G.C...........G.AACT...AG......G..........C..A.T.C..C.......G.T.A.A..G...A.......GT------.........G......CA.T.....CA.......T.AA.....A.C......T
Lv20_chr4S|0      ....A.........T..G............TT.C...A..................C...C.T.G.......G.A.......G.C........C.G..G..G...............C.....G........A......A.........
Lv21_chr4S|0      ...T.C.GG.....G.G.C........A..GAAACT...AG.........G.....C...A.T.C..C.......G.A.A.A.GG...A.......GGC.G.AC....G....G......AT.....CA.......T.AA.....C......T
Lv22_chr4L|0      .....G.........T..G............A..T...A.........T..............C...G.....TG.A.......G..........AC.G..G..G..............A.........T.G..........A........A..T

Tr28_Chr04|0      ------CATAAAAAATCAACTAAAAAAACCATTAACAAATCTTTCATATCGAATGTGGTTAGA---CCAACAGTACAGGAGCAAGCAGGT---AGATTT--------------------------------------CTTGGTGTTTGT
Kol_04_Nig        ..................................................................................................................................................................
Kol_04_Asa        .............................................................T....................................................................................................
Kol_05_Nig        CCAGTAT..G.TGC.C.G.TA....C..G...G..A.C....AG.T.A.CAA---ACCACC.A.GAG......AA.......G.......CGATGCTC.......................GACCCAAGGGGTGCATCAT.....C.....
Kol_05_Asa        CCAGTAT..G.TGC.C.G.TA....C..G...G..A.C....AG.T.A.CAA---ACCACC.A.GAG......AA.......................GATGCTC.......................GACCCAAGGGGTGCGTCAT.G...C.....
Lv14_chr4L|0      CCAGTAT..G.TGC.C.G.TG....GT.G...G..A.C....AGC..A.CAA.CCACCA.A.AG.....TGG.CAA..............GAT.CTC..GAATCTATTGAAAATCTAGAAGACCCGTTGGATGCATCAT.....C.....
Lv20_chr4S|0      .........A.....C...C.T........GTA..C..A.........C..A...A...AAA...........G...............G....T..GAT.CTC..GAATCCTTTGAAAATCTAAAAGACCCACTGAATCCATCAT.......A..C.
Lv21_chr4S|0      CCTGTAT..G.TGC.C.G.TA....---.G...A..A.C....ACC..A.CAA---ACCA.A.A.AAT.TG..TA......A.......T....GGTGCTC..GAATTCTTTGAAAATCGAGAAGACCCACTGGATCCATCAT.....C.....
Lv22_chr4L|0      .........A.C...C...C..........GTA..C..A.........C.A.A.A...AAA.A..A...........................T.CAAT.CTC..GAATCCTTTAAATATCTAGAAGACCCTTTGGATCCATCAT...A..A..C.

Tr28_Chr04|0      CCGAGTAGGCTATTTCAGGAGCCC---------AGTGCAATTGTTGTGCAAAGAGACTTTGTAAAACAGAAAAAATTTATAGTGTTTGAAGACTCCTTGGATATATTACTTTCTCTTGTTCGTTGCCAGCACTCACAATTGCCACCTTGT
Kol_04_Nig        ..................................................................................................................................................................
Kol_04_Asa        ...G...........................................A..................................................................................................................
Kol_05_Nig        ...AG......C..........T.AGGTAGTTTA.A.A...ACACA.AA....G..C....G....................A.C.T.............A...A.C.GTC.G...A.....G...........A..T.....G.C..........
Kol_05_Asa        ...AG......C..........T.TGGTAGTTTA.A.A...A.ACA.AA....G..C....G....................A.C.................A...A.C.GTC.G...A.....G...........A..T.....G.C..........
Lv14_chr4L|0      ...G.......C..........T.TGGTGCTTTA.A.AA.G...CA.AA....G..C....G....G...............C...................A...GTC.....G.....G.....C...........C..........A...
Lv20_chr4S|0      ...TG..GCATC..A...C.................A...ACAG...A.....A.......C.....................C.....................GG.C.......A.......C.........A.........GC..........
Lv21_chr4S|0      TT.G......TC.........CT..AGTGCTTTA.A.A..TCCACA.AA....G....TG...........G..........T...................A...GTC.T...A.....G.....C..........G.........A...
Lv22_chr4L|0      ..TGAA.C.TC.......T.................A.A....A..C...A......A...........................T..T........................G.C.T...A........................GC..........

Tr28_Chr04|0      CAAGCACCAGTTAGTCACATTGAAAAAAAAGTAGATGGAAGCTTGCTAACTGTGCATTTAACTTGCCTCAGTGGTCACAATTCACTAGTATGGAGTGCACAACCAGTGTTTGGAAATATATCCATTGGCAATGTTTTAATGGCCTCTACG
Kol_04_Nig        ..................................................................................................................................................................
Kol_04_Asa        ..................................................................................................................................................................
Kol_05_Nig        G.................................................................................T.A.AG........A.............A.T.C..................G.......C..................T...G.A
Kol_05_Asa        G.................................................................................T.A.AG........A.............A.T.C..................G.......C..................T...G.A
Lv14_chr4L|0      ..........................................G.................................A.A....C.....G.........C....A.A.C.....G.............G......GC..................G....GA
Lv20_chr4S|0      ..................................C........T.............................G...T.A.A...C.....A........C.....A.A.C.......G.............G.............C..............C..A
Lv21_chr4S|0      .........A........T.....G.......CC........G.................................A.A.A.....C...A.........A.A.C...........................G.G...............................GA
Lv22_chr4L|0      ....................AA.............C.......A..................A...T.A.A...C..T..A...---TC.....A.A.C.......C.........G...........................................CG.A

Tr28_Chr04|0      ATTCTACTAAGCAGTGTTTCATTAAAGAAAGTTCTAGAAATGTTTCATATTTTTGGAATACCAGCAAGCTCACCTAAGACATACCATGAATATCAAAAGGCGTATCTTTTTCCAGCAGTGGACTCTCAGTGGAGGCTGGAAGAAGGTAAA
Kol_04_Nig        ..................................................................................................................................................................
Kol_04_Asa        ...........................................................................T.....................................A..........................................
Kol_05_Nig        ...T.T.A...TG..TC.G.....G.C.....C.....C.....CC..AA..G.................T....GT.C.........T..C...............T...G...........................T.......G..C.G.
Kol_05_Asa        ...T.T.A...TG..TC.G.....G.C.....C.....C.....CC..AA..G.................T....GT.C.........T..C...............T...G...........................T.......G..C.G.
Lv14_chr4L|0      ...T.....C..TG..TC.......TG.T.....C.....C.G.......A.AGG...........A...T...................A............A.T.........C..............A..........A.....T.....GA.C...
Lv20_chr4S|0      .....T...........T.....C.TG......................G.........................T...........AG.......................A.........C..............A..............T........C..G
Lv21_chr4S|0      ...T...G..TG..TC......TG.C.....C.....C.......ACAGG..........A...T...................................T........C..............A..........A.....T.....GA.C...
Lv22_chr4L|0      .......................TG.....................G.........................T...........................GG.......A........................A..............T.TT....A.C..G

Tr28_Chr04|0      ATAAAAGCGGAATTGGCAGAAAATCCAGTTGCGCTAGCAACAGGTAGGCAGGTCATCAATCTTGGGCATTTCAATAAGTACTGTATGTATTCCATGATGGATGTAATGTCCAAAAAAATTGTCTCTTTCAAACCTGAACACTTTGGTCCA
Kol_04_Nig        ..................................................................................................................................................................
Kol_04_Asa        ..................................................................................................................................................................
Kol_05_Nig        ......AA.AC...T....G...AT.......A..G...G...............................C..G...........................G..T..TG...................AT.A..............
Kol_05_Asa        ......AA.AC...T....G...AT.......A..G...G...............................C..G...........................G..T..TG...................AT.A..............
Lv14_chr4L|0      ......AA..........G...A.........A.......G....C..........G............C.......A......CG.......................G.....T...................ATGA...C..........
Lv20_chr4S|0      ......AA................AT.T....TA..................TG..............C.G..............C....G....C.......................................T..AT.A...C..........
Lv21_chr4S|0      ......AA...........G...A.........A.......G....A.........................C............G....G...........G.............................ATGA...C.......T..
Lv22_chr4L|0      ......AA............TAG.G...................TG..G..................C.....................A.CG..............G.............................AT.A...C..........

Tr28_Chr04|0      GAAAGTATATTAGGAGATATTGGCAAAGAAGCATTGCAAATTTGTCTTGATCAACTAATTTATGGCAAGGTTAACGTGAAAATAATTGCCACCGATCAGCATGTCGGAATCAGAAAACTTATGGAGATAAAATATCCACATATTGAACAC
Kol_04_Nig        ..................................................................................................................................................................
Kol_04_Asa        ...........................C.................................................................................C..................................................
Kol_05_Nig        ...GAC.C....T.G..A.C..AA....C..A....C...GAC..C..................A...AG..AC.GG.TA.C.G..............A.............T........G..............................C.......
Kol_05_Asa        ...GAC.C....T.G..A.C..AA....C..A....C...GAC..C..................A...AG..AC.GG.TA.C.G..............A.............T........G..............................G...C.......
Lv14_chr4L|0      ...G.CC.C....AG..A.C..AG....CTGA.C..C....AC..CG.C.............AG..AT..AC.GG.TA.C..G.........T..A.......CACT.....T.........A................................
Lv20_chr4S|0      .....C......TG..A.C..AG.G.C.CA......GCC...............A....AGG..T.GG..A.C..............GG.........C.........A...........................G.......
Lv21_chr4S|0      ...G.CC.C....AG..A.CC.AG....C..A.T...C..GGAC............C...A....AT..AC.GG.TA.C...........A.......CCCT......T........A.............C.............G.......
Lv22_chr4L|0      .......C.C....TG..A.CG.AG.G.C.CA...T...GCC.................G...AG..T.GG..A.C.............A.........C..........G..........C..................G...C.......

Tr28_Chr04|0      CAGCTGGATGTTTGGCAGCTTTGCAAGATCGTTGCTCAAAGACTAGAGGAGACGAGCAAGAAAAGGAAATGTGGGAGCATAGCCAGATGGATTCCTGCCATCACAAACCATCTCTGGTGGTCCGCCCAAACCTGCAAACAGAATGTTAAT
Kol_04_Nig        ..................................................................................................................................................................
Kol_04_Asa        ..................................................................................................................................................................
Kol_05_Nig        .........A...............ATGTC....G..G..A..........G.T..........A...T..CA.T.T.......GAT.........................................TT..............T..A..C..CG..
Kol_05_Asa        .........A...............ATGTC.......G..A..........G.T..........A...T..CA.T.T.......G.T.........................................TT..............T..A..C..G..
Lv14_chr4L|0      ...T.....................A.GTC.........A..........G.A...........A.......CA.T.C..........T...C..TG..........A...................T.............T.........A...G..
Lv20_chr4S|0      .........................ATG.C..T.C........A..........G.A....T..............CA.T.A..........T.......GG..........A...................T.......T....C...A..C..G..
Lv21_chr4S|0      .........................A.ATC.........A..........G.A...........A...........A.T............T...C..TG............G.A...................T.............T.........A...G..
Lv22_chr4L|0      ...........G.............ATG.C.G..CA...A.T..A...T.G..A..T.CA...C....T....AC...........T......GA.T............A..................T................C...A..CAC.G..

Tr28_Chr04|0      CTTTTGATTGATAAATGGAAGTCGGTTCTTCTTCCATGTTGCAAATGTGCACCATTTTCCGTCCCTTAAACTTTACCAAAAGTGCCAGCAGTGAAAGGATTTCAAAAATGGAAGAAAAGAACACAGAGTGGATCCATTCAGATCACCCTGCC
Kol_04_Nig        ..................................................................................................................................................................
Kol_04_Asa        ..........G.......................................................................................................................................................
Kol_05_Nig        G....C..G..G.G...........C.......T...A.........C..A..TAG.........G..A....T......A.....A..T........C..........G.G.C...GC..C..G.A....A.C......T.C..TTA.C..T..A..T
Kol_05_Asa        G....C..G..G.G..........AC.......T...A............A..TAG.........G..A....T......A.....A..T........C..........G.G.C...GC..C..G.A....A.C......T.C..TTA.C..T..G..T
Lv14_chr4L|0      .....C.....G...............................................AG.......A....GT.C...T.....A.....A.........CA....C.....GG.T.C...GA..C...............A.C.......T.C..TA..A.....T
Lv20_chr4S|0      ...........G..........T..A..A.................................AG.....GA.......G....TAC...A..C.A........C.G...C.......G.TGCT..GC...T.......A.T......T......GGA.........T
Lv21_chr4S|0      ...........G...............................................AG.......A....GT.G...T.....A.....A.....A..CA....C.....G.T.......GA..C...............A.C.......T.C..T.A.C..T.....T
Lv22_chr4L|0      ......................A...........T...............................AG...CGAT..A..G.....C...A..C............G....C.....T.TGA...TC..TT..........T......T.......GCA......T.....T

Tr28_Chr04|0      CATGCAGCCCTGGCTGAAATAATCAACGATCCCCTTCTGCTGGATGATATTTCAAAGACAGAAACCTTCTGTCACACCAGAGATTTGGAGGCTTTTTACAGGACAATTGAGAAATGCCAGTCGGAACATTTACCTTTGAGTATTGATGAA
Kol_04_Nig        ..................................................................................................................................................................
Kol_04_Asa        ..................................................................................................................................................................
Kol_05_Nig        ..C.A.........TC...........GT.......A.....C.........C..G.C...G.......AA.....C.........T.........A...........C.A...............A.........A..G........T.A.CCGT...C.......T
Kol_05_Asa        ..C.A.........TC...........GT.......A.....C.........C..G.C...G.......AA.....C.........T.........A...........C.A...............A.........A..G........T.A.CCGT...C.......T
Lv14_chr4L|0      ...............C...................TT....CT.........G.......G..G.......AA...........................................T.C.A...............T.......A..........TT.CC.CGT..C..........
Lv20_chr4S|0      ...............C.........C...........T.............G....A..G.....AA........AG..............................C..C.AC..........T............A.........T............C.......G
Lv21_chr4S|0      ..C.......T......CA.............A.....TG....CT...........G.....G..C..AA...........................................T.C.A...............T.......A.........TT.A..CGT.GC..........
Lv22_chr4L|0      ..CT.............T..........G........T...G.......C.......AA......T..AA...........................................C...C...............TG......C..T..CG..T..............G

Tr28_Chr04|0      CTATATGCGCGTACTATACTGGCGGCTCTTTCATACAATCGCAATGTGCCCAGA---GAGCAGACCTCTGTAATATGGCCAGAATTATCCAATTTGAACACTGGGGATACCTTTAACAAAGTA------------GAAGAGAGCGAATGG
Kol_04_Nig        ..................................................................................................................................................................
Kol_04_Asa        ..................................................................................................................................................................
Kol_05_Nig        T..C...T...C...GCT.....T...T.G..T..T.....GG..C..GA....GGTC...CC---..A..G---G.....TTGAA...ACCA....G..---.CA..A.A.CCCC.....A.TGTTTTCCCCGAG...AGA..A..TCT.
Kol_05_Asa        T..C...T...C...GCT.....T...T.G..T..T.....GG..C..GA....GGTC...CC---..A..G---G.....TTGAA...ACCA....G..---.CA..A.A.CCCC.....A.TGTTTTCCCCGAG...AGA..A..TCT.
Lv14_chr4L|0      .......T...C...GCT.....T...T.A..........G...GAA.........A.CACAA..AA.G---......TTGAA.G.ACCA......GT...A..A.A.....CT....T.TTTTTCCCCTACG...AGA..A..TCT.
Lv20_chr4S|0      .......T...C...GGC.....T...T.A..........T.G...A..A..G...............CA.......C.A.......C.G...C.......G.AC.....GT..C.......T..AT..A.A.A..A---------...........---A.A..T..G...
Lv21_chr4S|0      .......T...C...GCT.....T...T.A..........A......G.A.T.........A.CACA...GA.G---......TTGAAC..ACCA......TGT....A..A.A.CA.C.....T..GTTTTCCCTATG....AGA..A..CCT.
Lv22_chr4L|0      .......T...C...GG.......T...T.AA..........T.G.......A..G.......TC..C.......CA.C....CA.......CAC....TTT.......T.T...A..A.A.A.......---------...........---A..A..TC.G...

Tr28_Chr04|0      GTG------CCCAAATCTGAGGAT---CGTGAAAGCTACCTCTTAGATATAATGTCGGACTCTGTGAAAATCTTGTTTGGCGGTCTGCAATATAATTGGATCTCGGTGCCGACCTTTCTCCCTCAAACAGTGGTACGCTGCAAAGCGGAG
Kol_04_Nig        ..................................................................................................................................................................
Kol_04_Asa        ..................................................................................................................................................................
Kol_05_Nig        ..TCTCGATA...T.CAG..A..CGCTGCG......TC..T.T.........GTC.....AC.AAT...........C....T.AA...G.C.------------------------------------------------.....G.AGT
Kol_05_Asa        ..TCTCGATA...T.CAG..A..CGCTGCG......TC..TCT.............AC.AATG..........C..A.T.AA....G..C.------------------------------------------------.....G.AGC
Lv14_chr4L|0      ..TCTCGATG...T..A...A..CAGTGTA......TG..T.T.C.............AC.AATAA.......G.....AA.T.AA..AG..C..------------------------------------------------....G.A..
Lv20_chr4S|0      ...........A...........T..........C...........A.......TA...........................A....AA.....G..A.....A....AT..A.CAT.A.A.....T...A....T---....TC........A...
Lv21_chr4S|0      A.TCTCGAT....TG.G....AA.CGGTGTA.....TG..T.T.CG..........AC.AATAA......GG......AA.T.AA..AG..C..-----------------------------------------------.....G.AG.
Lv22_chr4L|0      .........T......A....T.......T.......T.C......C.A...........TA....A..........................CA..G.G...A.....A.....G....A.CAT....A........T...A......---....TC.........A...
```

```
Tr28_Chr04|0        GTCTAA
Kol_04_Nig          ......
Kol_04_Asa          ......
Kol_05_Nig          ---...
Kol_05_Asa          ---...
Lv14_chr4L|0        ---...
Lv20_chr4S|0        ......
Lv21_chr4S|0        ---...
Lv22_chr4L|0        .A....
```

```
Tr29_Chr04|0   ATGCCCAGTTGCATAGTGAAAGGTTGTCGCCACAAAAGTGGACAGAAGATCCTGTATCCAGATGTTGTTCTTCATTCTTTTCCAAATAATATACATATGATAAAGAACTGGATGTTACAAACTGGTCAGGACTTTGGGGATATTGATGCC
Kol_05_Nig     ......................................................................................................................................................
Kol_05_Asa     ......................................................................................................................................................

Tr29_Chr04|0   TTTGCAGAGAAAATTCTGAAAGGAAATAAAACCGCCAGCTTCCGTATGTGCTCACGTCACTTTACAAGAGATAGCTATATGGCTAAGGGCTCCAAAATCACCTTAAAACCAAATGCTGTTCCAACAATTTTTGATACATTACCACCGGCT
Kol_05_Nig     ......................................................................................................................................................
Kol_05_Asa     ......................................................................................................................................................

Tr29_Chr04|0   GCTTCTGTTCCTAGTCTGATATCATTGCCAACAGCTAAGAGAATGAGAGTAGAAGATGAAGCACCATCTACCTCTGCCACTATTGTACGTATAGTTTCTAAACTTGTCACAGTACAAACACAAACTGATGACAGAATTCTCAAAAATGAC
Kol_05_Nig     ......................................................................................................................................................
Kol_05_Asa     ..................................................................................................A........A..........................................

Tr29_Chr04|0   CCCTGGGTCAATAGATACAACTGCCCAGTGACTGTCAGCGTTGCTACGCAGACCGATGCTCCAGCAGAGACCGACGATATGAAAACTGAAACTAGCAGCAGATCTCAAGAGGTAAAGATTTGGCATGCTGATAAAGACTATTTGTATCCA
Kol_05_Nig     ......................................................................................................................................................
Kol_05_Asa     .......................G...........A...................................A..............................................................................

Tr29_Chr04|0   GTATATGATGCACCGATAAAAACAAGCATGAAAACATCAGTTAAACAAACCACCAAAGAGCCAACAAAACAGGAGGAAGCAGGCGATGCTCTTGACCCAAGGGGTGCATCATTTGGTCTTTGTCCAGGTAGGCCATTTCAGGAGTCAGGT
Kol_05_Nig     ......................................................................................................................................................
Kol_05_Asa     ...........................................................................C.......T.......................G.....G................................T...

Tr29_Chr04|0   AGTTTAAATACAAACACAGAACAAGGACACTTGGTAAAACAGAAAAAATATCTTGTGTTTGAAGACTCATTGAACAGTCTGCTTACTCTGGTTCGTTGCCAACATTCACAGTCGCCACCTTGTGAAGCACCAGTTAGTCACATTGAAAAA
Kol_05_Nig     ......................................................................................................................................................
Kol_05_Asa     ..............T......................................A................................................................................................

Tr29_Chr04|0   AAAGTAGATGGAAGCTTGCTAACTGTGCATTTAACTTGCTTAAAGGGTCACAAATCACTAGTATGGAATGCCCAACCAGTGTTTGGAAATGTATCCATCGGCAATGTTTTAATGGCCTCTGCAATTTTTCAAAGTGGTTCTGCATTAGAC
Kol_05_Nig     .....................................................................T..............................................T.................................
Kol_05_Asa     .....................................................................T..............................................T.................................

Tr29_Chr04|0   AAAGCTCTACAAACCTTAAATGTTTTTGGAATACCAGCAATCTCGTCCAAGACATATCACGAATATCAAAAGGCTTATGTTTTTCCAGCAGTGGACTCTCAGTGGAGGCTTGAAGAGGGCAGAATAAAAAAGACATTTGCAGGAAAATCA
Kol_05_Nig     ......................................................................................................................................................
Kol_05_Asa     ......................................................................................................................................................

Tr29_Chr04|0   GTTGCACTGGCAGCAGGTAGGCAGGTCATCAATCTTGGGCATTCCAGTAAGTACTGTATGTATTCCATGATGGATGTGATTTCTGAAAAAATTGTCTCTTTCAAAATTAAACACTTTGGTCCAGAAGACACATTATGGGAAACTGAAAAA
Kol_05_Nig     ......................................................................................................................................................
Kol_05_Asa     ......................................................................................................................................................

Tr29_Chr04|0   CAAACATTCCAAGACTGCCTTGATCAACTAATTAATGAGAAACTGGATATCAGAATAATTGCCACCAATCAGCATGTTGGAATCAGGAAACTTATGGAGATAAAATATCCACATATCGAACACCAGCTGGATATTTGGCAGCTTTGCAAA
Kol_05_Nig     ......................................................................................................................................................
Kol_05_Asa     ................................................................................................................G.....................................

Tr29_Chr04|0   TGTCTTGGTCGAAAACTAGAGGAGGCTAGCAAGAAAAAGAATTGCAGTATCATAGCCGATTGGATTCCTGCCATCACAAACCATCTCTGGTGGTCTTCCCAAACCTGCAATCAAAACGTCGATGTTTTCATGGAGAGATGGAAGTCGGCT
Kol_05_Nig     ......................................................................................................................................................
Kol_05_Asa     .......C..................A...............................G............................................................T...........................A..

Tr29_Chr04|0   CTCTTTCATATTGCAAACGTACATAGTTTTCCGTCGCTAAAATTTTACAAAAAATGTCAGCATGCAAGGATTTCAGAGACGGAGCAACAGGAAACAAACTGGATTCCTTTTAACCATCCAGCTCACGAAGCCCTGGTCGAAATAATCAGT
Kol_05_Nig     ......................................................................................................................................................
Kol_05_Asa     .................T.....................................................................................................G..............................

Tr29_Chr04|0   GATCCCATTCTCCTGGATGACATTGCCAAGGCAGAAAAATTCTGCCACACCAGATATTTGGAGGCATTTTACAGCAAAATTGAGAAATACCAGTCAGGACATTTATCATCCGTTACTGATGATTTACATGTGCGCACTGCTCTGGCTGCT
Kol_05_Nig     ......................................................................................................................................................
Kol_05_Asa     ......................................................................................................................................................

Tr29_Chr04|0   TTGTCTTATAATCGGGATCTGGACAGAGGTCAGCCCTCAGTGGGGCCATTGAAATCACCATTGGACGCAGAAAACCCCCACAAAATTGTTTTCCCCGAGGAAAGAAGAGATCTGGTTCTCGATACCATACAGGAAGACGCTGCGGAAAGT
Kol_05_Nig     ......................................................................................................................................................
Kol_05_Asa     ......................................................................................................................................................

Tr29_Chr04|0   CACTTTTTAGATATGTCGTCACAAATTGTGAAAATCTTGCTTGGTGAACTGGAACATAAAGGGAGTTAA
Kol_05_Nig     .....................................................................
Kol_05_Asa     ....C.........AAT.........G...............A......................C...
```

```
Tr19_Chr04|0    ATGCCAAATTGCATTGTAAAGGGATGTCCTCACAGAACAGGCCAAAAATTAAAACACCCGGATGTTACCCTTCATGCCTTTCCACATAATTTACATCAAATAAAGAAATGGCTAATGCAAACAGGACAATAT---GGTCATGATCTTGAT
Kol_06_Nig      ........................................................................................................................
Kol_06_Asa      ........................................................................................................................
Lv11_chr4S|1    ....G....T.....G.................A.....C...T......................G..A..C......................C.......C....C..A..A.C.....
Lv16_chr4L|1    ...........................T...A...........T..........A.....C.A..........................TC.........T..C......A....C....G
Lv17_chr4S|2    ..A...GA..T..................A.T...A...........T.........T......C.......A..........G.................T..C..CAT..A.C..C.....

Tr19_Chr04|0    TTAGTGGCAGACAAAATTTTAAGGGGAACTAAAAATTCAAATTTTAGAATGTGCTCTACCCACTTCTCAGAACACTTTTATACATATAAAGGATCAAAGAGGATACTAAAGCCAAATGCAGTCCCAACAATTTTCCTTACTTCTCCAGTA
Kol_06_Nig      ........................................................................................................................
Kol_06_Asa      ........................................................................................................................
Lv11_chr4S|1    A.C..A...C.A.....AA.GTTA---.GC...CC.A..................T.......TG..GG.A...CT....T........G......C...G.................CA.A.........
Lv16_chr4L|1    .C.C............AC....A...G.C...................A..........G.G.A.......T.....C.........G..G...........T.........TA.....C....
Lv17_chr4S|2    ..............G..C..C.A...GTC....G....TG..........................G...........T.........G.G.C...CC...........T..T.......TTC....AT.....G

Tr19_Chr04|0    CCTTCAATAATAAGAGCTCAGAGTTGTGGTTCTTCAGATCCTGCTGCAAAGAGAGTGAGAGTAGATGAT------CCATCAACA---TTC------ACAGTTGTACGTGTAGTTTCAAGACTTATCACTATCGGGACACAAACTAATGAC
Kol_06_Nig      ........................................................................................................................
Kol_06_Asa      ........................................................................................................................
Lv11_chr4S|1    .G.C........AC.T...A..------A......T......A........A..........C...AATGAG.........TCA....................A.........G.....G.T...........G.C---
Lv16_chr4L|1    .T.C...............A..------A......T......A........A...........CCTGAA.........TCA...................G...............G.T............G..C..
Lv17_chr4S|2    .T.C.........AT....A..------G......T......C........A....A....G...CATCAA...------.....TAAATCC..T...A....A..G...........G.....G.............G..C.T

Tr19_Chr04|0    AGAAAAAGTCGAACTGATGCGGGTACATTGACAACGAAAATGTCTGTCAATCAGGACATTGCAACACAAACCGAACCTCCGTGTAAAATGGAAATCGGCATACAAACCGGTGATGATTCAATAGAGGCAGAACCTTGGAAGGTCGAGAAA
Kol_06_Nig      ........................................................................................................................
Kol_06_Asa      ........................................................................................................................
Lv11_chr4S|1    ....T..T.A....GA....TA...........---..TT..G..A.G..AA....TT..A.........T...TT...AGT.....A......T.CT.......A..C.............A.......G........T..A.G.
Lv16_chr4L|1    ....G..A...........TTA.....A.......A....T.....AAAG..A....TG..................A..AGT...........T.CT.......G..............T..A.....G.T..........C.....
Lv17_chr4S|2    ....G..A..AG........TA.................T.....AAGG.A.CA..TG...............T.G..AAA..............CT..T.....G..................A.....GATC........T.....

Tr19_Chr04|0    GATCATTCATATCCAGTTTGTTTTGTTGATCCCTATAAAAGTGTGGATCCTCTTGAAAATATATCAAGTGTTTCAGAAAGTAAAAAGTCACTTTCTTCAAAAGAATATTTACCTACTGTTCATACACTAGAAGGGGAATCTTCACCAAGC
Kol_06_Nig      ........................................................................................................................
Kol_06_Asa      ........................................................................................................................
Lv11_chr4S|1    ......AT...C.........................T..A.A...................CGT..........A..........A...G.........G....T.....C...G....TCT.C......G..A.......
Lv16_chr4L|1    ......AT...C...........A.C..............A.A.....C......G.---------A.A..T.........G....C.A....G......G..G....T.....C........TC.A..........C....A..A.
Lv17_chr4S|2    ......A.G..C...........C...............T.A.A..A..C..........---------AAA..G................C...A.C..G......G..G....T.....C......T.T.....A.........C.......A.

Tr19_Chr04|0    CTGACAAATTCACCAGAGGGCATCTCAACAGATCAAAACATTCTTAATCTAACTACTGAAAGTCTAAAGCAGTCTGCCATACACAAACATTGTGGCTATGTAAGACACAGAAAATTTATCATCTTCGAGGAGAACCTTGATAGTCTACTG
Kol_06_Nig      ..........................................................................A.............................................
Kol_06_Asa      ........................................................................................................................
Lv11_chr4S|1    .C.---..AAAG.....CAC.TA.....A.........T.C..C....C...............GGT......A......T......C....A......CC....A.........C.........T.....................T..
Lv16_chr4L|1    .CA..........T.......T.................TT..........A................G.......A..G.....C......C...AT......C.....A..................T........T.............
Lv17_chr4S|2    ..T....C..........A.CAG.................T............................GG.......A...GC.TT.......C.A.A......C.....A..................T.....................G...

Tr19_Chr04|0    TATCTTATTAAATGCCAGCATCAGCAAACACAGCCGTGTCAAGCACCTATCATAGATATTCAAAAATCCATTGATGGAAGCATGGTTCAAGTAAAGCTTGTATGCCTGGATGGTCATGACTCGCTTATATGGAACTCACAGCCGTTATCT
Kol_06_Nig      ...................................................T....................................................................
Kol_06_Asa      ........................................................................................................................
Lv11_chr4S|1    ...T.G..........A............C...A...........A..TC..........G.........A..........................T.....A.........CA........G...........G.....AC....C
Lv16_chr4L|1    ...T.G.................CA.......ACA..A...........A..TT..A.......G....T....A..............A.........G....T.....T.........CA.G......G...........T.....A.....
Lv17_chr4S|2    C..T.G..........A............C...A..............TC..........G.........A..........................T.....A.........CA........G...........G.....AC.....

Tr19_Chr04|0    GGTGATATTCCTGTTGGAAATGTTTTATTAGCCAACTGTGTTCTTCTCTGTGGTTCTTCATTTCAGAAAGTTAAAACAATGTTGAATTTATTAGGTATCCCGTCTTTTTCACTTTCTACCTATTATAAATACCAAATGTCATATCTTTTT
Kol_06_Nig      ........................................................................................................................
Kol_06_Asa      ........................................................................................................................
Lv11_chr4S|1    .C...CG..T...CC........C.............GC...................CT.........A......G.G.AG.....TC..G....GC..........TC......GA.A.........C..........G.....C..C......
Lv16_chr4L|1    .....C...T.............C......G.......A....G...G.T........TG.........A.........G.AG......G.........A........A.T........AGA....A....GC.....T.........C..C......
Lv17_chr4S|2    ..C..CG..T...CC........C.............GC......................T.........A.........G.AG.....TC....G...C..........TC......GA.A.........C..........G.......C......

Tr19_Chr04|0    CCAGCAATTGATCTACGCTGGCGCCAGGAGCAAGAGTATAATAAAAAGGAATTGTCTGGAAAATCAGTTGCTGTAGCTGGAGACTGCCAGTTTGATACTCCTGGCCAGGCAGCTAAATACTGCACGTACACCATGATGGATGTAGTGTCC
Kol_06_Nig      ........................................................................................................................
Kol_06_Asa      ........................................................................................................................
Lv11_chr4S|1    ...............G.AG.........A..A......AG....C............A...........T......A.T..............T.................T........T.C..C.....T..T..A......................A....T
Lv16_chr4L|1    ...T............AG.........T..A......AG..C.C.........................T......T..............T.....C...........T...CT..A.C.....T..T..A......................A.....
Lv17_chr4S|2    .......G.........G.........A..A......AG....C............A...........T......A.T..............T.G...............T...T...T.C..C.....T..T..A......................A....T

Tr19_Chr04|0    AAAAAAATTCTTTCCTTTACTATTGACCAACTTGGAAGAGGAAAAACCTCTGCTGAGGTGGAAAAGGCTACATTCGAGACATGCTTAGACAATCTGTTAGATGAACATGTAGATGTCAAGATCATTGCTACAGGCCGTCATGACGATATC
Kol_06_Nig      ........................................................................................................................
Kol_06_Asa      ........................................................................................................................
Lv11_chr4S|1    ........C........C...............CA..........---AA.........A.....AA..G....T.....G.........T...A.A..........A..A...........................A.........T.GC..T
Lv16_chr4L|1    ..............................A.................TA.G.T.A.....A.........T............G..T..A..A........CA..A............................CA..........T.G....
Lv17_chr4S|2    ........C........C.........................TG......G..........A.AC..G..T.............G..T..CA.A.........A..A...........................AT.........T.GC..T

Tr19_Chr04|0    GGAAAACTCATGGAAACAAAATACTGTAGAATTGACCACCAGCTTGATGTGTGGAGTCTATGTAAAAGTCTGGCAAAAAAACTCATTGTGGCAAGTAAGAAAAGGCACTGTCGTGACATCTACAAGTGGATCTCTGCAATAACAAATCAT
Kol_06_Nig      .............................................................................................T..........................
Kol_06_Asa      ................................................C............................................T..........................
Lv11_chr4S|1    ............C..........C.G.TG........................AA............G.A...........AG...CA.......G..............A.........G...................G.......
Lv16_chr4L|1    .A.......G...C..........C...C...............A...............A...G.....G...G.AT..........GG...CA...................G.............G................C.........
Lv17_chr4S|2    ............C..........C.G.TG........................AA............G.A...........AG...CA.......G..............A.........G...................G.......

Tr19_Chr04|0    CTCTGGTGGTGTGCGCAGACCTGCAACCAAGATGTTGACGTTTTGCTCGATAAATGGAAATCTGTTATGTTCCATTTATCAAATAAGCACAGTTTCCATTCCTTGGAAAATTACAAAAAATGCCAACACAAGAGAATTCCTGCTGTGAAA
Kol_06_Nig      .....................T.......................------------------------------..............................................
Kol_06_Asa      .....................T..................................................................................................
Lv11_chr4S|1    ..G..................T..T........................C...........G..G.......A...C....T..................C.C.T..T..G........GC.CA..........
Lv16_chr4L|1    ..G..................T..T.................T..........G...............A..G.......A...C..........A.................C.T..T............C..AA..........
Lv17_chr4S|2    ..G..................T..T.................T.............C...........G..G.......A...C....T..................C.C.T..T..G........GC.CA..........

Tr19_Chr04|0    GCGCGCAGGTTTGCATGGATTACATCTGAACATCCTGCATATGCTCCCCTGTCTAAAATTATTAATGACAAAGCCTTGCTCAGAGACATTTCAAAAATAGAAAAATTTTGCCACACAAGAGACCTAGAGAGCTTTCGCAGTAACGTCCTC
Kol_06_Nig      ...............................C........................................................................................
Kol_06_Asa      ...............................C........................................................................................
Lv11_chr4S|1    .A.....A..G...T........G..CC...........C..A.G..........G.........C....G.......A..C................................C.........C.........A...C..A.C...A
Lv16_chr4L|1    .A.....A..G...T...........C...........C..A.G..........GG...........C..........C................................G.........................A...C..A.....A
Lv17_chr4S|2    .A.....A..G...T........G..CC...........C..A.G..........G.........C....G.......A..C................................G.........C.........A...C..A.C...A

Tr19_Chr04|0    AAATACAAATCTAAACGATTGTCGCTTAATATGGACTCAATGTATGCAGATACAATTCTGGCTGCTCTTTCACAAAACAGAAATGTGAGTAGAGAAGAAGCAAAGCTAAACTGCCCACAAAACTCGCCTTTGCCTTTTGGAGAAAAACAC
Kol_06_Nig      ....................T...................................................................................................
Kol_06_Asa      ........................................................................................................................
Lv11_chr4S|1    ..............A...CA..AT.C..C..............CA.....................G.....T...........T........G........AA....T.CTG............------.............G.....
Lv16_chr4L|1    ..............A...C.G.AT.C.GC..........G....CA............T..............T...........T........G..T.T...TA....T...G.......A.........................G.
Lv17_chr4S|2    ......C....C...A...CA..AT.C..C..............CA.....................G.....T...........T.C.....G.........AAAG..T.CTG............------.............G.....

Tr19_Chr04|0    TATAAAGTCAGTTTCTGCAAGCAGAATAACAGGGTTGAA------------GATGATGTGGTTGATGATCATTTGCTTGATATAATGTCAAACTCACTGAAAATTCTGAATGGAGAGCTGGTTAATCAGTGGTCCTCAAGGTCAAGAATA
Kol_06_Nig      ........................................................................................................................
Kol_06_Asa      ........................................................................................................................
Lv11_chr4S|1    ........AGC.....C.........AC......A...TAAATAT.........A.....T..C..C...T.....T.................................C...........C...T......................
Lv16_chr4L|1    .G......AGC.....C....TC.G.A........A...TAAATTTGTTGGT..A.....T.....GA..T...........C..............T..A...........................A..............
Lv17_chr4S|2    ........AGC.....C.........AC......C...TAAATATGTTTGT.CA.....T.....C...T.........................CT...........G...........................T..............

Tr19_Chr04|0    CTCCGCTAA
Kol_06_Nig      .........
Kol_06_Asa      .........
Lv11_chr4S|1    ........G
Lv16_chr4L|1    ........G
Lv17_chr4S|2    .........
```

```
Tr5_scaffold_769|10  ATGCCTAATTGCATAGTACGCGGGTGTCCACACAAAACTGCACAAAAAGACAAATATCCAGATGTCAGTTTACATTATTTTCCAAACGACCTCAATGCAATAACGAATTGGCTGAGACAAACTGCGCAGTATGGTGAGAATTTGGAATCC
Tr8_Chr08|9          ............................................................A..........G.....C........T.....................................G.............T..........A
Tr11_scaffold_609|9  ............................................................A................C........T.....................................G.............T..........A
Tr36_scaffold_161|8  .........................................G..................A..........G...CTC........T......................................A............TG..........
Kol_07_Nig           ............................................................A..........G.....C........T.....................................G.............T..........A
Kol_07_Asa           ............................................................A................C........T.....................................G.............T..........A

Tr5_scaffold_769|10  GTAGCTAACGAAATTCATCAATCGGCAAAAACAGGAAGACACAGAATTTGTTCTGTCCATTTCACAGAAGATTCGTTTGTCGCCAAACGTTCAAAAATAATGCTAAAAGCGAATGCTGTTCCCACAATTTTTGATATTCAGGCCACTCCA
Tr8_Chr08|9          .......................A...................................C.................CT........G.C.G.....G..........C................G...............C........
Tr11_scaffold_609|9  .......................A...................................C.................CT........G.C.G.....G..........C................G...............C........
Tr36_scaffold_161|8  ..C.............................G..........................C.......G.........C....T....G.G.G.....G..........C.......A.............A..........C........
Kol_07_Nig           .......................A...................................C.................CT........G.C.G.....G..........C................G...............C........
Kol_07_Asa           ...................T...A...................................C.................CT........G.C.G.....G..........C................G...............C........

Tr5_scaffold_769|10  GTTTCGGTAACCGCAATGGAATCTAAATCCTTCCAAAGTCACAAGAGAAGAAGAGTAGAGGATCATATTCCTTCCACCTCTCACACGATTGTGCGTATTGTCAAACATTTTATGACTGTTGCAACCCAAACTGAAGAAAAGATCTACGTT
Tr8_Chr08|9          ....TA.....A..T.........CT.C....T..............................G...................TT............................C................................T...
Tr11_scaffold_609|9  ....TA.....A..T.........CT.C...................................G...................TT............................C................................T...
Tr36_scaffold_161|8  ....TT.....A..T..........CTC....A..............................G...................T...........C.................C................................T...
Kol_07_Nig           ....TA.....A..T.........CT.C....T..............................G...................TT............................C................................T...
Kol_07_Asa           ....TA.....A..T.........CT.C...................................G...................TT............................C................................T...

Tr5_scaffold_769|10  GATGCAAGTACCACATCCATGGATATGCAGCTTGGTATTCATAAAGCCAGTGGTTCTGATAATCCA------CAGCTCGCTATAGGAGTACAGACAGGAGACGATTCAGTGCCAGCCGAACCTTGGCGAATCCAAAAAGATCATGGCTAT
Tr8_Chr08|9          ...................C......T.......................................CATTCA.T.....A.....................T.........G...T.........AA.......................
Tr11_scaffold_609|9  ...................C......T.......................................CATTCA.T.....A.....................T.........G...T.........AA.......................
Tr36_scaffold_161|8  ...........A.......A..............................................CATTCA.......A.....................T.........G........G....A.....A....G.............
Kol_07_Nig           ...................C......T.......................................CATTCA.T.....A.....................T.........G...T.........AA.......................
Kol_07_Asa           ...................C......T.......................................CATTCA.T.....A.....................T.........G...T.........AA.......................

Tr5_scaffold_769|10  CCCGTCGCGTTTTCCACGTCCATAAAATGTATGTTGGATATAGATAAACCTAATCCAACTGTACCGGGTAAGACCATACAAACAGTGCAAGAATCTAATTTGGAGGGAGAATATGAATTGTTTGAACTTACTGACAGTCAGCTAAGCAGT
Tr8_Chr08|9          .....T..A........AC.G..C..........................C.......A.T....C.....AGA...G............................A...GG.G..........C....T..........C.........
Tr11_scaffold_609|9  .....T..A........AC.G..C....................A.....C.......A.T....C.....AGA..AG............A...................GG.G....A..........T..........C.........
Tr36_scaffold_161|8  ..T..T..A........AC.G..G....C...C.........................A.T....C.....AGA...G...G..A.G.......A................G.G...............C....................
Kol_07_Nig           .....T..A........AC.G..C..........................C.......A.T....C.....AGA...G............................A...GG.G..........C....T..........C.........
Kol_07_Asa           .....T..A........AC.G..C....................A.....C.......A.T....C.....AGA..AG............A...................GG.G....A..........T..........C.........

Tr5_scaffold_769|10  ATCCAGGCAGTGGAGCCCAGTATA------------------AAAGAAGAAAAGGACACGTCTATTTATGAACCAGAGGAAAGCGTTGGTTTTACAGAAGCTGATTCAACGTTTACTGAAAAAAAAGATGAAATTCTTCAGCGCAGTTTT
Tr8_Chr08|9          .........A................................G.G..............A.......................T..AC............A...........................G..............A......
Tr11_scaffold_609|9  ......A..A................................G................A..........................AC............A.....T.....................G..............A......
Tr36_scaffold_161|8  .........A..............GAAGAAGAAGAAGAAGAAG................A.......................T..AC............A.....C..A......G...........G...........AA.A......
Kol_07_Nig           .........A................................G................A.......................T..AC............A...........................G..............A......
Kol_07_Asa           ......A..A................................G................A.......................T..AC............A.....T.......G.............G..............A......

Tr5_scaffold_769|10  ATAGTATTTGAAGAACTACTGGACCAACTATTTTATCTAGTAAAATGTCAACATAGTGCTAACTCGCCATGCCACGCACCAATAGTTGGAATTGAAAAAAAATTAAATGGAACTATGGTAGAAGTCAAGTTAACATGTCTTTCCGGACAT
Tr8_Chr08|9          .............................C.........A...G............C........A........T............CA...AC..........G....................A.................T......
Tr11_scaffold_609|9  ........C....................C.........A...G............C........A........T............CA...AC..........G....................A.................T......
Tr36_scaffold_161|8  .............................G.........A..................G......A......T.T.............A...A...........G......................................T......
Kol_07_Nig           .............................C.........A...G............C........A........T............CA...AC..........G....................A.................T......
Kol_07_Asa           ........C....................C.........A...G............C..C.....A........T............CA...AC..........G....................A.................T......

Tr5_scaffold_769|10  TGCTCTTTGATATGGAACTCTCAGCCAATAGCAGGACAGGTATCTATTGGAAATGTATCAGTAGCCTGTGCCATTTTACTGAGTGGATCACCCTTTACAAAAGTGAAGGAAATGTTTCAGCTATTGTCTATCCCATTCTTTTCTCATGCT
Tr8_Chr08|9          ...G...................A...C...........A...................G..............................T................A...............A.......A........C......A.C
Tr11_scaffold_609|9  .......................A...C...........A...................G..............................T................A...............A.......A........C......A.C
Tr36_scaffold_161|8  .......................A..GC...........A.......C......T....T.....A...........G............T................A...............A.......A...............A.C
Kol_07_Nig           ...G...................A...C...........A...................G..............................T................A...............A.......A........C......A.C
Kol_07_Asa           .......................A...C...........A...................G..............................T................A...............A.......A........C......A.C

Tr5_scaffold_769|10  GCATATTGCACCTATCAGAAACAATATATATTCCCCGCAATTGATTTGGCATGGATCAAGGAACAAGAATTATTGAAACAGGATCTTGCGGATAAAGCCGTTGTTTTGGCTGGCGACGGCCAATTGGATAGTCCCGGCCATAGTGCAAAA
Tr8_Chr08|9          A......AT..A..........G....C.G......C.T.......................................G....GA....A........T..............T...........T........A..T........T...
Tr11_scaffold_609|9  A......AT..A..........G....C.G........T.......................................G....GA....A........T..............T...........T........A..T........T...
Tr36_scaffold_161|8  A......A...T..........G....C.G......T.T.................................G...........A....A...C....T..............T...........T........A..T........T...
Kol_07_Nig           A......AT..A..........G....C.G......C.T.......................................G....GA....A........T..............T.A.........T........A..T........T...
Kol_07_Asa           A......AT..A..........G....C.G........T.......................................G....GA....A........T..............T...........T........A..T........T...

Tr5_scaffold_769|10  TACTGTACCTATACCATGATGGACATTACGACCAAAAAAATTGTGGATTTCACTATAGAGCAAGTTTGTCCTGGAAAAACTTCAGGGCAAACGGAAACAATTGCTTTTGCAAAATGTCTATTACGCTTGGAAAAAAAAGGAGTAGACATT
Tr8_Chr08|9          ..........................A.T............CT..C.................A...T...A...C...G........G..T...GGG......A....A...........C.A.......G.........A.....G..
Tr11_scaffold_609|9  ..........................A.T............CT..C.................A...T...A...C...G...........T...GGG......A....AA..........C.A.......G.........A.....G..
Tr36_scaffold_161|8  ..........................A.T............A..........A..........A..AT...G.....GG.......AT...T...GGG..GA.......A...........C.AA......................G..
Kol_07_Nig           ..........................A.T............CT..C.................A...T...A...C...G.....A..G..T...GGG......A....A...........C.A.......G.........A.....G..
Kol_07_Asa           ..........................A.T............CT..C.................A...T...A...C...G...........T...GGG......A....AA..........C.A.......G.........A.....G..

Tr5_scaffold_769|10  AAAGTGTTGGCAACTGATAGACACAGTAGTATTAGAAAATTCATGCAAACTAAATCTGACACCATCAACCACCAATTGGATGTATGGCACATTTGTAAAGGTCTGGTAAAGAAGCTTAGGGCCGCAAGTAAACGAAGAAAATGCAAAGAT
Tr8_Chr08|9          .G...A.................T..C..........GCA.G...A..GTG..C.A.CCG.................T.....................A.................CA..T..........AA...G........G...
Tr11_scaffold_609|9  .G...A.................T..CT.........GCA.G...A..GTG.CC.A.CCG.................T.....................A.................CA..T..........AA...G...A....G...
Tr36_scaffold_161|8  .....A.A...............T........A.A.....A...........G.A....G..A..............T.....................A.................CA..T..........AA...T....TG..G...
Kol_07_Nig           .G...A.................T..C..........GCA.G...A..GTG..C.A.CCG.................T.....................A.................CA..T..........AA...G........G...
Kol_07_Asa           .G...A.................T..CT.........GCA.G...A..GTG.CC.A.CCG.................T.....................A.................CA..T..........AA...G...A....G...

Tr5_scaffold_769|10  ATAGCCCACTGGATAGGGCCAATCACCAAGCACTTGTGGTGGTGTTCCCAAACATGCGACCGGAACGTAGAAAATCTTCTAGATAAATGGCGATCGCTGCTCTATCATATCGCCAACAAACACACGTTTCCAAATCTCAAAACATATAAA
Tr8_Chr08|9          .....A.......................C...............................AT..T..T..C......T................TG............................A....G.....G.G...........
Tr11_scaffold_609|9  .....A.......................C...............................AT..T..T..C......T................TG............................A....G.....G.G...........
Tr36_scaffold_161|8  .....A.C.....................C...........................T...A...T..T........CT................AG............................A....G.....A.....T.......
Kol_07_Nig           .....A.......................C...............................AT..T..T..C......T................TG............................A....G.....G.G...........
Kol_07_Asa           .....A.......................C...............................AT..T..T..C......T................TG............................A....G.....G.G...........

Tr5_scaffold_769|10  AAATGCCAACACAGAAAAATGACAGCTGGGGAAATAAGAGAAAAAAAAATGGATAACTCCATCTCATCCGGCTTATTGCACTTTAGTGGCAATTCTAACCGACAAACTACTGATTAGAGACATTTGCCAAACTGAGAAATTCTGCCATACG
Tr8_Chr08|9          ......G......AG.....T.G..G..C.......G.A..T.................................A.............C.......A.AG.....A.....G.A........AC.....T...................
Tr11_scaffold_609|9  ......G......A......T.G.....C.......G.A..T.................................A.............C.......A.AG.....A.....G.A........AC.....T...................
Tr36_scaffold_161|8  .............A......T.....A.A.......G.A.........------------------------------------------------------------------------------------------------------
Kol_07_Nig           ......G......AG.....T.G..G..C.......G.A..T.................................A.............C.......A.AG.....A.....G.A........AC.....T...................
Kol_07_Asa           ......G......A......T.G.....C.......G.A..T.................................A.............C.......A.AG.....A.....G.A........AC.....T...................

Tr5_scaffold_769|10  GGGGATCTGGAGAACTTCCGCAGTAAAGTGCTGAAATACTGGCCGGAGAGAATTGATTTTACATTAGATTCCATGTACGCGAGGACAATGCTGGCGGCGCTGTCACATAACAAAAACGTGAATCGGCCGCAGGCTAGCGCTCGTTGTAGC
Tr8_Chr08|9          ................A.A...C.....CT......C...TA.............C...A.C...............T..TC..........A..A...........C............G....T..A.........CT.T..A.....
Tr11_scaffold_609|9  ................A.A...C.....CT......C...TA.............C...A.C...............T..TC..........A..A...........C............G....T..A.........CT.T..A.....
Tr36_scaffold_161|8  ------A....T...------------------------------------------------------.....C---------------------------------------------------------------------------
Kol_07_Nig           ................A.A...C.....CT......C...TA.............C...A.C...............T..TC..........A..A...........C............G....T..A.........CT.T..A.....
Kol_07_Asa           ................A.A...C.....CT......C...TA.............C...A.C...............T..TC..........A..A...........C............G....T..A.........CT.T..A.....

Tr5_scaffold_769|10  AAAATATCGGAACTCCCTGTTGGAGAGAAAACATTTCAAATAGTCTTTCCTAAACATAACAAAGAATGGGTAGCCAAACCTATTATTGAAGAAGTGGGTGATTGGCGTCTGTTTGACATTATTTCTGATGCAGGCAAAATATTGAAAGGG
Tr8_Chr08|9          ....A...A...T.T..A........A....G....A.......A.....A........A..............A.....G...T........A.TTC..GTT.A.....A................................G.....T
Tr11_scaffold_609|9  ....A...A...T.T...........A....G....A.......A.....A........A..............A.........T........A.TTC..GTT.A.....A................................G.....T
Tr36_scaffold_161|8  ------------------------------------------------------------------------------------------------------------------------------------------------------
Kol_07_Nig           ....A...A...T.T..A........A....G....A.......A.....A........A..............A.....G...T........A.TTC..GTT.A.....A................................G.....T
Kol_07_Asa           ....A...A...T.T..A........A....G....A.......A.....A........A..............A.....G...T........A.TTC..GTT.A.....A................................G.....T

Tr5_scaffold_769|10  GAGATTGTTCCCCGTTGGGAATCTCATAGCAAGGGATTAGCGGGAAATATTGCATCCAAAAACAGCCCTGAGAAACCTGTTGTTGTAGCACAA---TACTCACTCAATAATGTGACAGTTGTTTGGGGTAATACATAG
Tr8_Chr08|9          ...G......A............A...C...........C.TAA....A...A...A..G.A...G...A...T.A................GCAC....AGG--------------------.TT..A.CGTGC..A
Tr11_scaffold_609|9  ...C......A............A..GC...........C.TAA............A..G.A...G...A...T...............G..GCAC....AGG--------------------.TT----------GA
Tr36_scaffold_161|8  --------------------------TCATCC..C...-------------------------------------------------------..----------------------------------------...
Kol_07_Nig           ...G......A............A...C...........C.TAA....A...A...A..G.A...G...A...T.A................GCAC....AGG--------------------.TT..A.CGTGC..A
Kol_07_Asa           ...G......A............A...C...........C.TAA....A...A...A..G.A...G...A...T.A................GCAC....AAG--------------------.TT..A.CGTGC..A
```

```
Tr32_Chr03|0        ATGCCTAATTGTATAGTTCATGGATGTCGCCATCGAACCGGACAGAAAGAAAAATACCCTAACGTAACTCTACATAATTTCCCCAATGACATACATAAAATTAAAAATTGGCTTAGGCAGACTTCGCAGTATGGTGAAGAACTGGATTCA
Kol_08_Nig          ......................................................................................................................................................
Kol_08_Asa          ......................................................................................................................................................

Tr32_Chr03|0        GTAGCAAATGCAATCCAAGAAGTTTCTAAAACTGGTAGATATCGCATGTGTTCGGTTCACTTCACCGAAGATTTATTCATGACTCAAGGCTCTAAAAGGGTACTAAAACCTAATGCTGTACCCACAATTTTTGTTACTCAACCAATTCCA
Kol_08_Nig          ......................................................................................................................................................
Kol_08_Asa          ......................................................................................................................................................

Tr32_Chr03|0        GTGTCTGTAACAGCCATGGAATCTGCACCTTACCAAAGCCGCAAAAGAAGACTAGTAGAAACGGAGGTGGTTCCTTTAACCTCGCACACCATAGTGCGAGTAGTTCGAAATCTTATTACCATAGGAACGCAAACCGATGCTTCTCCTGTG
Kol_08_Nig          ......................................................................................................................................................
Kol_08_Asa          ......................................................................................................................................................

Tr32_Chr03|0        TACCCCACACCAATAAAACCAACTCATGCAAATACTCGAGCTCAACACAGACAAACTTCATTACAAGAAACCCCTAAAGTATCAAATCTGGAAGAAGAACAGAATTTGTCTCTAACACTTGATGAATCCCTAAGTAGTATAGAGGAAATT
Kol_08_Nig          ......................................................................................................................................................
Kol_08_Asa          ......................................................................................................................................................

Tr32_Chr03|0        GACCCCAGTAGAGAAGGTGAAATAGATACATTATTATATGAGCCTGAAGAAAGTTTCACAGAGAAAGATACTACAGAAAAACCTGACAAAATTCATCAGCGACGGTTTATAGTGTTTGAGGAACTTCTGGACCAACTTTTGTATTTGGTA
Kol_08_Nig          ......................................................................................................................................................
Kol_08_Asa          ..G...................................................................................................................................................

Tr32_Chr03|0        AAATGTCAACACAGTACACCATGCCACGCACCTATTGTGGAAATCAAGAAAAAAAAATTACGGAACAATGGTAGAAATAAATCTAACCTGTCTTGCTGGACATTGTTCTTTGGTTTGGAATTCTCAACCAACGGCAGGACAGATTTCGGTT
Kol_08_Nig          ......................................................................................................................................................
Kol_08_Asa          ......................................................................................................................................................

Tr32_Chr03|0        GGAAATTTATCATTAGCATGTGCCATTTTATTAAGCGGATCTTCCTTTACCAAAGTTGAGGAAATGTTTAATATGATGGCAATACCAATTTTTTCTCACAAAACATTTTATAGATATCAGAAACAATACATTTTTCCTGCCATTGATCTA
Kol_08_Nig          ......................................................................................................................................................
Kol_08_Asa          ......................................................................................................................................................

Tr32_Chr03|0        GCATGGAAAACCGAACAGGAATCATTAAAAAAGGACATACTTGGTCAAGCTGCTGCTTTGGCTGGTGATGGACAGTTTGACAGCCCTGGTCATTCTGCTAAATACTGTACATACTCAATGATGGACATTAGGATGAAGAAAATTGTGGAT
Kol_08_Nig          ......................................................................................................................................................
Kol_08_Asa          .................................................................................................................T....................................

Tr32_Chr03|0        TTCACAATTGACCAAGTTGGCCCTGGAAAAAATGTTGCGAAAATCAAAAACATTGCACTTGAAAAATGTCTTGAAAATTTGGAACAAAAAGGAGTTGACATTAAAGTTTTGGCCACAGATAGACATAGCAGCATAAGAAATATTATGAAA
Kol_08_Nig          ......................................................................................................................................................
Kol_08_Asa          ......................................................................................................................................................

Tr32_Chr03|0        ACAAAAAATTACATCATCAACCATCAATTTGATGTGTGGCATATTTGTAAAAGCTTGGTTATAAAACTAAGAGCTGCAAGCAAAAAAAGAAAATGTAAAGACATTGCACAGTGGATTGGACCCATTACTAACCACTTGTGGTGGTGTGCT
Kol_08_Nig          ......................................................................................................................................................
Kol_08_Asa          ......................................................................................................................................................

Tr32_Chr03|0        CAAACATGTGACCAGAACGTAGAGAATCTTTTAGATAAATGGCGTTCATTACTTTACCATATTGCTAATAAGCACACATTTAAGAACCTTAAAACTTATAAAAGCTGCCAACATAAGCAATTACCAGCTGAGGAAAGCAGAGACAGAAAA
Kol_08_Nig          ......................................................................................................................................................
Kol_08_Asa          ...........................................................................................................................A........................G.

Tr32_Chr03|0        TGGATTACACCCTCACATCCTGCTTACAGCACTCTGGTTGGCATTTTAAACAGCCCTCTACTAATTAAAGACATCTCGAAGATTGAAAAATTCTGCCACACAAGAGATCTGGAAAACTTTCACAGTAAAATTCTGAAGTACAGGCCAAAA
Kol_08_Nig          ......................................................................................................................................................
Kol_08_Asa          ......................................................................................................................................................

Tr32_Chr03|0        AAAATCTCATTTAACATTGATTCAATGTATGCCAGAACCATGCTGGCTGTCCTGTCTCACAATAAAACTGTTAATCGCCCCCAGGTAACGATTTGCAGCAAAAAACACAAACTTTTTGTTGGTGAAAAAAAGAATTACAGTGGTATTTCC
Kol_08_Nig          ....................................................................................................................................................A.
Kol_08_Asa          .....................................................................................C.....................................................T..........

Tr32_Chr03|0        CAAGGAAAAGAAGGAAAACCAACCCACCTTTTAAACATCATTTCTGATGCTGAAAAAATTGTTAAAGGGGAGCTAGTTCACCGTTGGGAGTCAAGAACGGGAGGTTACCTGCCAATATTGCGTCAAACAAAGACCTGAAAAATTGA
Kol_08_Nig          ..................................................................................................................................................
Kol_08_Asa          ..................................................................................................................................................
```

```
Tr14_scaffold_51|5  ATGCCAAATTGTATTGTTAGAGGCTGTCCACACAAGACAGGACAAAAAAAAGCACATCCAGATGTGACTCTGCATAGTTTTCCCAACAATATAAACCTAATAAAAAACTGGCTTAGTCAGACTGGCCAATATGGACAACAAGTTGACTCT
Tr15_Chr01|5        ......................................................................................................................................................
Kol_09_Nig          ......................................................................................................................................................
Kol_09_Asa          ......................................................................................................................................................

Tr14_scaffold_51|5  TTTGCAGAAAAAATTTTTAACGGACTGAAAACTGCAAGTTACAGAATGTGTTCACAGCATTTCAGTCAAGACTGCTTTACTGTGAAAGGAGATAGACGAATCCTTACACCAAACGCTATCCCCACCCTGTTTATAAGTCGTCAAGTGACT
Tr15_Chr01|5        ....................G............................................................A....................................................................
Kol_09_Nig          ....................G............................................................A....................................................................
Kol_09_Asa          ....................G............................................................A....................................................................

Tr14_scaffold_51|5  GCTGTTGTTACAGCCCAAGAAACTGGTACAAGTATTCCCTCAGCAAAAAGAAGAAGAGTAGAGGAAGATGATACTATGACAACTACTTCTTCTACAATAGTTCGTATTGTTTCTCGCCTAGTAACAGTACAGACACAAACAGAACTCAAA
Tr15_Chr01|5        .........................................G....................................C.......................................................................
Kol_09_Nig          .........................................G....................................C.......................................................................
Kol_09_Asa          .........................................G....................................C.......................................................................

Tr14_scaffold_51|5  GATTTTACAGATAATTCCTCAACAACCGACATAAAATATTTTACAAAAGATGTTGCTTGTGGAGCTGAAAATGCAATATTCAACAAAGAAATAGCTATTCAGACGGGCGACGACTCAGTAGAGGCCGAAGAATGGAGGATTATGAAAGAT
Tr15_Chr01|5        ..................A...................................T...............................................................................................
Kol_09_Nig          ..................A...................................T...............................................................................................
Kol_09_Asa          ..................A...................................T...............................................................................................

Tr14_scaffold_51|5  CATCTTTATCCAGTTGCATTTTCTACACCAAGAAAGGCAACAGTTCTTCCAAATGTATCTATTCCTGATACTGAAATATACACATCTCAAGAAGAATCAAACATTGATTTAGAATCCATATCTGATGAAGAAGAAAACCTCAATGAAAAT
Tr15_Chr01|5        ......................................................................................................................................................
Kol_09_Nig          ......................................................................................................................................................
Kol_09_Asa          ......................................................................................................................................................

Tr14_scaffold_51|5  CTGGATTCAACTTATGAACCTTCGATGGATACTTTTACATCGGAACAGCATTTTGGTGATGATTCTGTATGTAGTCAACAAGTACCAGAAATTCAGCAAAGAAAATTTCTTGTTTTTGAAGATCAGTTGAATCAATTATTATATTTGTGT
Tr15_Chr01|5        ......................................................................................................................................................
Kol_09_Nig          ......................................................................................................................................................
Kol_09_Asa          ......................................................................................................................................................

Tr14_scaffold_51|5  AGATGCCAACATTCATTTACTTCACCGTGTCAAGCACCTATTATTGGGATTAACAAGAAATTGGATGGGACTATACTTGAAGTCCAGCTAACATGTCTAGAGGGCCATGAATCGTTGTTATGGAAATCACAGCCCTTGGCGGGCCAAATG
Tr15_Chr01|5        ...............................................................A..................................................---.................................
Kol_09_Nig          ...............................................................A..................................................---.................................
Kol_09_Asa          ...............................................................A......................................................................................

Tr14_scaffold_51|5  CCACTGGGCAATGTTGCAATTGCAAATGCTATTCTTTTGAGTGGATCATCTTTTACCAAAATCAAAGAATTTCTGGAAATACTTGGTATGCCTTTTTTTTCAAACACCACCTATTACAAATATCAAAAAAGATATGTCTTTCCTGCTATA
Tr15_Chr01|5        ......................................................................................................................................................
Kol_09_Nig          ......................................................................................................................................................
Kol_09_Asa          ......................................................................................................................................................

Tr14_scaffold_51|5  GAGTTGGCCTGGAAACAAGAAAAGGAAAAATTGATAAGTGAGATGTCTGACCAAGCTGTAGTCCTAGCTGGAGATGGACAATTCGATAGTCCTGGCTACAGTGCTAAATTCTGCACGTATACCATGATGGACATTTTAACTAAGAAAATT
Tr15_Chr01|5        ......................................................................................................................................................
Kol_09_Nig          ......................................................................................................................................................
Kol_09_Asa          ......................................................................................................................................................

Tr14_scaffold_51|5  GACACATTTACAATTGACCAGGTAGTCCCAGGAAAAACATCCGGGCAAATAGAAGCTGCTGCTTTTGAAATGTGCCTGAACGAAATTCAAGAACAGGGGATTGACGTCAGAATAATTGCCACAGATCGTCATCCTGCCATACGTAACTTG
Tr15_Chr01|5        .T....................................................................................................................................................
Kol_09_Nig          .T....................................................................................................................................................
Kol_09_Asa          .T....................................................................................................................................................

Tr14_scaffold_51|5  ATGAAAACTAAATACAACCTTATCGATCATCAGTTTGATGTGTGGCATCTCTGCAAAAGTTTAGCCAAAAAACTGTCTGCTGCCAGTAAAAAAAAAAAATGGAAAAGACATAGGACCATGGATTGGAGCAATTACAAACCATTTGTGGTGG
Tr15_Chr01|5        ........................A.............................................................................................................................
Kol_09_Nig          ........................A.............................................................................................................................
Kol_09_Asa          ........................A.............................................................................................................................

Tr14_scaffold_51|5  TGCTCGCAAACCTGTGACCACGATGTCAACCTCTTAATTGACAAGTGGAAATCGCTGTTATTTCATATCGCAAACAAGCATACTTTCAGAAGTCTAAAAAATTACACCAAATGTGATCACAAAAGATTGCCTGCTTCACAAAGCGAGGAT
Tr15_Chr01|5        ..........................................................................................................................................G...........
Kol_09_Nig          ..........................................................................................................................................G...........
Kol_09_Asa          ..........................................................................................................................................G...........

Tr14_scaffold_51|5  AAAAATTGGATCACCACTAGTCATCCTGCTCATACTGTCTTGACGCAAATAATTACAGATCCAAAACTCATCAAAGATATTTCAAAAGTAGAAAAATTTTGCCATACTGGGGAGCTGGAAAATTTTCATAGTAAAGTGTTAAAATTTAGG
Tr15_Chr01|5        ......................................................................................................................................................
Kol_09_Nig          ......................................................................................................................................................
Kol_09_Asa          ......................................................................................................................................................

Tr14_scaffold_51|5  CCCAAAAGAATATATTTCGGCATGGATTCAATGCATGCACGCACAATGCTGGCTATTTTATCTCACAACAAGAATATTGGCAGACCGCAAGCAACAATTCAAGATGCCACAAAAAGTAGTCTTGAACTTGGGCAGAAGAGATTCAAAATA
Tr15_Chr01|5        ......................................................................................................................................................
Kol_09_Nig          ......................................................................................................................................................
Kol_09_Asa          ......................................................................................................................................................

Tr14_scaffold_51|5  GTTTACCCCAAACAAAAGAAAGATTGGGTGGCTAAACCCATTTACGAAAAAGTGACTGACAGTCATTTATTTGATATCATGTTGGACTCAGCCAGAATTGTCAATGGCGAACTGGTCCATCGATGGGAGTCACGAAGTTCAAAGTACCCT
Tr15_Chr01|5        ......................................................................................................................................................
Kol_09_Nig          ......................................................................................................................................................
Kol_09_Asa          ......................................................................................................................................................

Tr14_scaffold_51|5  GCAAACATTGCATTGACTGAAAGGCCTGAAAAAACTGAAGTGATTGCTAAGCACTTGTCTCGATTTGAGAGAGAATAG
Tr15_Chr01|5        ...................................C..........................................
Kol_09_Nig          ...................................C..........................................
Kol_09_Asa          ...................................C..........................................
```

```
Tr1_Chr05|1     ATGCCTAGCTGTATAGTGAGAGGTTGTCCACACAAAACTGGGCAGAAAGCTAAGTATCCGGATGTGACGCTTCACACCTTTCCACACAAGTTAGATTTAATAAAGAACTGGCTTCGACAGACGGGCCAATATGCAGAAATCAATCACGTC
Kol_10_Nig      ......................................................................................................................................................
Kol_10_Asa      ......................................................................................................................................................

Tr1_Chr05|1     GCGGAAAGGATCCTACAAGACATTAAAAAAACAAGCTATCGCATGTGTTCCAACCACTTTACTGAAGATTGTTTCATGAATTTGGGAAGTAAAAGAGGCCTAAAACCAAACTCCATACCAACCCAGTTTGTACATCGTAAAGTCACTGCT
Kol_10_Nig      ......................................................................................................................................................
Kol_10_Asa      ..................................................A........C..........................................................................................

Tr1_Chr05|1     GTTCTAACAGCACAAGAACCTGTAACAAATATACCGGCGGCAAAAAGAAGAAGAGTCGAGGAAGATCCTATGCCAACTACTTCGACAATAGTTCGTATCATATCTCGCCTAGTAACGGTACAAACACAAACTGAACGCAAATATTTCAAA
Kol_10_Nig      ......................................................................................................................................................
Kol_10_Asa      ......................................................................................................................................................

Tr1_Chr05|1     AACAGCTCTACGATAACCGATATAAGTTATTCTGCTAGGGGAGATCAAACAATCCACAAT------GGAGATGAGTCAACAGAGGCTGGAGAATGGAGGATCCCGAAAGATCATTTGTATCCAGTTGCATCTGCTACGCGCGGTAAGCCT
Kol_10_Nig      ......................................................................................................................................................
Kol_10_Asa      ...........................................................CGAAATT........C................................................................A.A........

Tr1_Chr05|1     AGATTTCTTCAAAAAACCACAGAAATGCCTTCCTTATTCATTCGTGATACTGGAACCCATCATGATGAGCCAAACTTTGATATAGAATCCATATCAGACGAAGAAAATATTCACGATGAAACTTTGGATTCAACGTCGGAACACGACCAG
Kol_10_Nig      ......................................................................................................................................................
Kol_10_Asa      .....C.........................................................................................C......................................................

Tr1_Chr05|1     AAACTGCACTCAAGTCATTTTGGTCACGATTCTATGTTGTCTCGACAAGTAGAAGATATTAATCAAAGAAAATTTCTTGTTTTTGAAGAGCAGTTGGACAAATTATTCTATTTATGCAGATGCCAACATTCATCTTCTTCTCTGTGTCAA
Kol_10_Nig      ................................................................................................A................G....................................
Kol_10_Asa      ....A..................................A........................................................A................G....................................

Tr1_Chr05|1     GCACCTATCATTAAGATAAACAAGAAATTAGATGGGACTCTCCTTGAAGTCCGGCTACAATGTCTAGAAGGGCACGAATCATTAGTCTGGACATCGCAGCCCTTGGCAGGCCAAGTTTCACTGGGTAACGTAGCGATGGCAAACGCTGTA
Kol_10_Nig      ......................................................................................................................................................
Kol_10_Asa      ......................................................................................................................................................

Tr1_Chr05|1     CTGTTGAGTGGATCATCTTTTACAAAAATCAAAGAGTTTATGGAAATACTCGGCATGCCATTTTATTCACCAACCATCTATCGCAGATATCAAAGAGACTATGTGTTTCCTTCTATAGATTTGGCCTGGAAGCGAGAAAAGGTAAACTTG
Kol_10_Nig      ......................................................................................................................................................
Kol_10_Asa      ......................................................................................................................................................

Tr1_Chr05|1     CTTAGTGAGATGTCCGACAGAGCTGTAGTTCTAGCTGGCGATGGACAATTCAACAGTCCGGGTTACAGCTCTCAATTCTGCACTTATACAATGATGGACGTTTCAACTAAGAAAGTCATTGCTTTCTCCATTGACCAGGTGGTCCCAGGA
Kol_10_Nig      .....C..................T.............................................................................................................................
Kol_10_Asa      .....C.............................................................................A...............A..................................................

Tr1_Chr05|1     AAAACCTCCGAGCAAATGGAAGCTGTTGCTTTTGAAAGGTGCCTCGATGACGTTAAAGACCAGGGGACCGATGTGAAACTAATAATCACAGATCGTCAGCCCGCCGTACGTCAGTTGATGTTGTCTAAATACAAATCTATCGATCACCAG
Kol_10_Nig      ...............................................................................................C......................................................
Kol_10_Asa      ..........G...........................................................................................................................................

Tr1_Chr05|1     TACGACGTGTGGCGCGTCTGCAAAAACGTTGCCAAAAGGCTGTCTGCTGCCAGCAAAACGAGAAACGGGAAAGCCATCGCCCCGTGGGTCGAAGCCATAACAAAGCATCTGTGGTGGTGCTCACAAACCTGTTACCGGAACGTCGACGTT
Kol_10_Nig      ......................................................................................................................................................
Kol_10_Asa      ..........................................................................................................................G...........................

Tr1_Chr05|1     TTAATCGACAAATGGAAATCCGTTTTATATCATATCTCTAACAGGCATACTTTCCCAGCTCTTAAACATTACAATAAATGTCATCACAAAACATGGACTGCGGCACAAAGTGAGTGTAAAAGATGGATCCCCCCTGGTCATCCTGCTCAC
Kol_10_Nig      ......................................................................................................................................................
Kol_10_Asa      ......................................................................................................................................................

Tr1_Chr05|1     ACCATCTTGACGAAAATAATTACCGACCCCAAACTAATCAAAGATATTTCGCAGATTGGGAAATTCTGCCACACAGAGGAACTTGAGAATTTCCATAGCAAAGCATTCAAATTTAGGCCTAAAGGGATGTGTTTGGGGGTGGATTCCATG
Kol_10_Nig      ......................................................................................................................................................
Kol_10_Asa      G.......................................................A.............................................................................................

Tr1_Chr05|1     CACGCGCGCACCATGCTGGCCGTTTTATCTCACAACAGGAACACGGGCAGATGGAAGGCAACCAATCACGATCCCAGAAAAAGTCGTCTGGAGGTTAGGCAGGAGGGAATCAGAACTACATACCCAAAACGGAAGGAGGATTCTGTGGCT
Kol_10_Nig      ...................................................C................T...........................G.....................................................
Kol_10_Asa      ...................................................C................T...........................G............................................C........

Tr1_Chr05|1     AAACCCATCTATGAACAAGTGACCGACCCTCATATATTTGATATCATGTCGGACTCGGCCAGAATTGCAAAAGGCCAACTGCTTCATCGATGGGAGTCACGGTGTTAA
Kol_10_Nig      ............................................................................................................
Kol_10_Asa      ..................................................A.........................................................
```

```
Tr7_Chr02|2     ATGCCTAGCTGTATAGTGAAAGGATGTTCTACTTGGACTGGTCAAAGGGAAAAAAACCCATCCATTATTTTGCATTCATTTCCCAAAAATATTGAGCAAATAAAAAAATGGTTAGCTCAGACGGGCCAATTTTGTGATGACATTGATGCC
Tr9_Chr02|2     ......................................................................................................................................................
Kol_11_Nig      ......................................................................................................................................................
Kol_11_Asa      ......................................................................................................................................................

Tr7_Chr02|2     AAGGCTGAAGAAATTTTAAAAGGCAAATCTAATAACAGATTCCGTATGTGTTCACGACATTTCTCAAGGGACTCTTACATTGCAAAAGGTGTCAAAATTCAGCTGAAACCAAATGCGGTACCTACCATATTTAATATAGTTACACCTGAC
Tr9_Chr02|2     ......................................................................................................................................................
Kol_11_Nig      ......................................................................................................................................................
Kol_11_Asa      ......................................................................................................................................................

Tr7_Chr02|2     ACCTCGATTGCAATATTACATGATATACCGTCAGCTAAACGCAGAAGAGTGGAGGACGAGCCATCAACCTCTTCTTGCACCGTACGAATAATTTCACGTTCAGTCACAGTCGCTACGCAAACTGATCAAAAAATTTTTACTTGTGATTCC
Tr9_Chr02|2     ......................................................................................................................................................
Kol_11_Nig      ......................................................................................................................................................
Kol_11_Asa      ...................................................................G................................................................T........A........

Tr7_Chr02|2     TCAGTTAACACTGACAATTGGCCATTAATATATAATGTTCAGACGCAGACCGTTCCTTTTGCTATAACCAAGGACATTGAAGTGCAAACAGGAAGTCACTATGTAAATGCAGATGCATGGAAAATTAGGAACGATCATCGTTATTCAACT
Tr9_Chr02|2     ......................................................................................................................................................
Kol_11_Nig      ......................................................................................................................................................
Kol_11_Asa      ......................................................................................................................................................

Tr7_Chr02|2     TGTTTTTCAACACCTGTTAAGGAGAATCAAGAAAGCAAGAATGTTCCAATTAGTCATTCAACACCCATTGCTTTCACTGGAAATACTCAATTATCTCCAATTAATAAACCAGATTCCGAGGCTGAAGAAGATGAAGAAAGTGTGTCTTTT
Tr9_Chr02|2     ..................................................................................................................................T...................
Kol_11_Nig      ..................................................................................................................................T...................
Kol_11_Asa      ........................................................................A................G........................................T...................

Tr7_Chr02|2     ATGGACGACCCTAAAGATCTCACCTATAACCTTTCCAAGTCTTCACAAAGCAAATCTTTAATTTATTGTTCATCAGAGAGTGATTGTGATGACAGCATTCACAGACAAGTGGATACAGTTCGCGAAAGAAAATTTATTGTGTTTGAACAC
Tr9_Chr02|2     ......................................................................................................................................................
Kol_11_Nig      ......................................................................................................................................................
Kol_11_Asa      ......................................................................................................................................................

Tr7_Chr02|2     TGTCTAGATATTCTTTTTCATTTAGTGCGATGTCAGTATCAGATAGAAAATTGTTGTGATGCCCCAGTTGTCGAAATAAAAAAAAAAATTGATGGAAGTCAGCTCAGTGTCAAATTGACATGCCTGAATGGCCACCAAGCTCTCTTTTGG
Tr9_Chr02|2     ............................................................................................................................C.........................
Kol_11_Nig      ......................................................................................................................................................
Kol_11_Asa      ......................................................................................................................................................

Tr7_Chr02|2     AGAAGCCAGCCAGTAATTAAAAGTACCTCTGCAGGCAATGTTCTAATGGCAGCATCAGTTTTACTAAGTGGATCATCATTCAGGAAAGTACATGAGATGTACAGTATATTAGGAGTATCGACCATTTCGCACACAACTTTTTATAACTAT
Tr9_Chr02|2     ........A.............................................................................................................................................
Kol_11_Nig      ......................................................................................................................................................
Kol_11_Asa      ......................................................................................................................................................

Tr7_Chr02|2     CAGCGAGCCTATTTATTTCCTTCGATTGACTATCACTGGCAGCAAGAAACTAACAACATAAGGAAAGAAATTGGCAACAATGAAGTCGTTATTGCTGGGGATGGGCAATTTGATAGCCCTGGACACTCAGCAAAATATTGTATTTACTCA
Tr9_Chr02|2     ......................................................................................................................................................
Kol_11_Nig      ......................................................................................................................................................
Kol_11_Asa      ..............T........A..............................................................................................................................

Tr7_Chr02|2     TTAATGGATGTGCTTTCAAAGAAAATCATCTCATATACAATTGAACAACTTGGTCCAGGTAAAAACTCATATAACCTTGAAAAGGAGTCATTTCAAAGTTGTCTGGATGGTCTTCTTGCCGACAATATTAATGTGAAAATTGTTGCCACA
Tr9_Chr02|2     ..............................................................................................................................G.......................
Kol_11_Nig      ......................................................................................................................................................
Kol_11_Asa      ..............................................................................................................................G.......................

Tr7_Chr02|2     GACAGACACTCAGTAATCAGAAATGTCATGTCAACTAAATATAAAAATATAGATCACCAATTCGATGTATGGCATCTTTGCAAGTCACTAAACAAGAAACTGATGGCTGCTAGCAAAAAAAAAAATTGCGGAGATATTTCAAAATGGATC
Tr9_Chr02|2     ..........................................G...........................................................................................................
Kol_11_Nig      ......................................................................................................................................................
Kol_11_Asa      ......................................................................................................................................................

Tr7_Chr02|2     TCTTCTATAACAAACCATTTATGGTGGTGTTCTCAAACGTGTGACCAAAATGTTGAAGTCTTACTATCCAAATGGAAATCACTCATGTTCCATATTTCGAATGTTCACACTTTCCCAAAACTTGAGCATTATAAAAAATGCATTCACCCA
Tr9_Chr02|2     ......................................................................................................................................................
Kol_11_Nig      ......................................................................................................................................................
Kol_11_Asa      ......................................................................................................................................................

Tr7_Chr02|2     AAAATATCACGCAAAGAAGTAAGAAAAGCACCATGGATTAAACCAGGGGATCCAGCACATGCCACACTTGCAAGTATTGTAAATAGTAAATACCTTCTGAAGGACATGCATCATATTGAGAAATTCTGCCACACCGGTGACTTAGAGAAC
Tr9_Chr02|2     ......................................................................................................................................................
Kol_11_Nig      ......................................................................................................................................................
Kol_11_Asa      ......................................................................................................................................................

Tr7_Chr02|2     TTTCACAGTAAGGTACTGAAATATCGGTTGAAGCGGATTGCTTTTAAGATTGATGCTATGCATGCCAGAACTGTGCTTGCTATTCTTTCGCACAACCGAAATGTCAATCGTCCACAAGCAACTGTCAAATGCGCAAAAAAGTCAACCTTA
Tr9_Chr02|2     ......................................................................................................................................................
Kol_11_Nig      ......................................................................................................................................................
Kol_11_Asa      ......................................................................................................................................................

Tr7_Chr02|2     CCTGTTGGATCAAAGAGGTACAAATTTGTATTCCCTAAGCATAAAAAAAAATTGGGTGACAAGGGCCATCTATGAGGAAGTAGTGGATGACCACCTACTCGACATTGCTGGAGATAGCTTGAAAATATTGTCTGGGGAGCTGATTCACAAC
Tr9_Chr02|2     ......................................................................................................................................................
Kol_11_Nig      ......................................................................................................................................................
Kol_11_Asa      ..............................................................................................................................G.......................

Tr7_Chr02|2     TGGGATAGCAAATCCTCATCTGTCCCTGCAAATATTGCAACACTGGAGCGACCGGACAAAAATGAGCTGATCAGTAAACATGTGTCAAGATTTGCTCGATAA
Tr9_Chr02|2     ......................................................................................................
Kol_11_Nig      ......................................................................................................
Kol_11_Asa      ......................................................................................................
```

```
Tr13_Chr02|0      ATGCCAAAATGTATAGTGACCAACTGTCCACACAGGACTGGACAGAAAGAAATTTATCCATCTGTCATCCTTCATCCATTTCCAGCAAATATCGAAAAAATAAAAAAGTGGTTATTACAAACTGGTCAGAACTATGGTGACTATGAAGTA
Kol_12_Nig        ......................................................................................................................................................
Kol_12_Asa        ......................................................................................................................................................
Lv7_chr2L|0       ...................A...A...........................C.A......A....A...........................T...........C...................AG..G....................
Lv8_chr2S|0       .......................A..........A................C.G...............................G.......T...........C..........G........AG..G....................

Tr13_Chr02|0      TTTGCAAAAAAGGTCCTAGAAGCTAAAAAAACAGATGCATATCGGATATGTTCCAGACACTTTGCTGAAGATAAATATGTGCAAAAAGGTCCACGTAAACTTCTTAGAAAAGATGCCATTCCCACTCTTTTCAGCAACCTTCACCCTTTA
Kol_12_Nig        ......................................................................................................................................................
Kol_12_Asa        ......................................................................................................................................................
Lv7_chr2L|0       ......G.....A.TT..AT.........................G.......AC.T...............C.G........G.........T............A.........TG........A....T..................
Lv8_chr2S|0       ......G.......A......................................AC....T............C........A.G.G.......T.............T........AG........A.......................

Tr13_Chr02|0      ATTCAGCTTCACAGTATCACAGGACCCCCTCCACGAAAAAGAAAAAGGGGAGATGACAAAGAGTCGGGCCCATCAACTTCCAGTATAGTCCGTATCATCTCTCGAGCGAGAACCATTGGCACTCAGACAGATCCTGAATATTTGAAGAGA
Kol_12_Nig        ......................................................................................................................................................
Kol_12_Asa        ......................................................................................................................................................
Lv7_chr2L|0       ..C...........C...................A.........G......A.......GA.CGG..T...........................AG..........A......................T......G..T.C.......
Lv8_chr2S|0       ..............C..................A.G.......GG..............G....G..T..A........................AG..........A......................C......G..T.........

Tr13_Chr02|0      ACTACTGGAATGACCACCCTTCCCCTGTCATTCATGTGCTCCACATATACGCAAAGTGAGGTACCATTTGAGGTGATGCACATGCAATGCCAGACCGGTGAAGATTCAGATCGTGCTGAGTTTTGGAGAATTGAAAAAGATCACCTGTAT
Kol_12_Nig        ......................................................................................................................................................
Kol_12_Asa        ..........A.........................................................................................................A.................................
Lv7_chr2L|0       .............A....A.....T.C.........CA......................C....T...........................A..AAA.............A........................G.....A......
Lv8_chr2S|0       ...T...A.....A....G.....T.A.....G.........T.G...............C....T..........C................A.A.A......G...........A.....C..............G............

Tr13_Chr02|0      CCTGCCAGCTTCTCAACACCCTCAAAACCAAGCATCAACAGCCAGTGGAACACTGATGAATATTCAGAGCCGGCAAAGTTGTGGAGGGTTGAAAAAGATCACCTGTATTCTATCAGCTTGTCAACACCCACAAACCCATGCATAATAACT
Kol_12_Nig        ......................................................................................................................................................
Kol_12_Asa        ..............G.......................................................................................................................................
Lv7_chr2L|0       ...A...A..................G..---..------........C.G..AG................T............C.A........G.......................................A.......C......
Lv8_chr2S|0       ...A..........T...A....C.....---..---------.A..C.G..C.G................T..............A..A.G.G..........A........TG..........A..TG.....A.......CT.....

Tr13_Chr02|0      ATTCAAACCCCCAAATCCACAAGA---------CCCGACACACTTCCAGTTCAAGAAAATAAGATGACTGAAGTGGCACTAGCGACATCATTCTCCACTACTGAAGAGCCGAAAGACCCAAATTTAGACTCTCTTGAGTAC---------
Kol_12_Nig        ......................................................................................................................................................
Kol_12_Asa        ...........................................................C..........................G...............................................................
Lv7_chr2L|0       ........A.AA.....T.....CGGTCATATA..T.......C....A.........AG...........G...A.....A..T..........G......A..TT..A..C.....GC.....C.CA........G...ATGTGTGCT
Lv8_chr2S|0       ........A.AA.....A.....CGGTCATATG..T.......C.T..A.........AG..G..G...............AT..T....T......T.C.....T..A...T......C.....G.C.............ATGTGTGCT

Tr13_Chr02|0      ---ACGCCGCAGGATTCTAGAGGATCTTCT---AAATCTAAC---TACAATCAGGCTGACAAAATCAGTTATATAGCTGACAGAAAATTTGTCGTTTTTGAAAGCTCTTTGGATAAATTACTCAGGCTTATTCCTTGTCAGCACAATGAG
Kol_12_Nig        ......................................................................................................................................................
Kol_12_Asa        ....................................................................................................................................................GT
Lv7_chr2L|0       GAC.T.T.A......G...T......CG..CTG.....A..TGACC....C..AC.CA......C.GA......CA..............A..A...C......G.............................................
Lv8_chr2S|0       AAC...T.A......G...T......CA.GCTG.....G..TGAC.G...C..AC.CA.....CC..A......C...............A..A...C......G........................C....................

Tr13_Chr02|0      GGTGAAAAGTGTGAAGCACCAATCAAAGAAATACAAAAACATATAGATGGTTCTATGGTCAATATACAACTATTGTGCCGTAACAATCACAAGTCCCTTTACTGGAATTCTCAGCCAATGACTTCAGACATGGCAGTGGGCAATATATTA
Kol_12_Nig        ......................................................................................................................................................
Kol_12_Asa        A.........................................................................................................................C...........................
Lv7_chr2L|0       ..C.....................GC..................................T.....A.GT.........A..T................A.................G....C.......T.........G........
Lv8_chr2S|0       A.C..............T.....TGC....................................A................A...................A.T.............................T...C..............

Tr13_Chr02|0      ATGTCTTCTTCTATTGTTCTAAGTGGATTATCATTTCAGAAAGTCAAAGAAATGTACGACCTTTTTGGGGTACAGGCAATTTCCCATACAACATTTTGCCAGCACCAGAATAAATATATTTTCCCAGCAATTGACTATAACTGGAAGAAA
Kol_12_Nig        ......................................................................................................................................................
Kol_12_Asa        ......................................................................................................................................................
Lv7_chr2L|0       ........A......A.G..........CG.....C.....................A.......................................CA.G.........C...G..................C............A...
Lv8_chr2S|0       ........G......A............C......C........A.......................T.........................................C.........................GG........A...

Tr13_Chr02|0      GAGCAGCAAGAAATTTTCAAGGAAATTGGAGAGGATAGTGTTGTCCTTGCAGGTGACAGACAGTTTGACAACCCTAGACAATCTGCAAATTACTGCATTTATTCCTTCATGAACATCCCTACACGCAAAATTGTAAATTTTAAAATTGTA
Kol_12_Nig        ......................................................................................................................................................
Kol_12_Asa        ..........................C........C......................................C.............T.............................................................
Lv7_chr2L|0       ..A.....T........T..T........GC....GCA.........C.....A..T.............G..C..GA.C.........................A...G......C......AA.........................
Lv8_chr2S|0       ........G.....G..T.................GCA...............A....G...........G..C..GA.T........................G.A...C.....C......AA..G..............G....C..

Tr13_Chr02|0      CAGTTGGAAGCTGGTCAGAAACCTACAGTAATGGAAAAACAGGCTTTCCAAGAGTGCCTCGATGAGCTGCTTTCCAAAAAAGTTAACGTGCAGATGGTGGCAACGGATAGACATGTTCCCATCCGTGAATTGATGGCCACAAAGTACAAA
Kol_12_Nig        ......................................................................................................................................................
Kol_12_Asa        ......................................G.................................................................T.............................................
Lv7_chr2L|0       .C.....................AGTGAC..........G.......T..GA.....T.T..C...G.................GC...............T..A..C..................C.......................
Lv8_chr2S|0       .................A.....AGTG.A.G........GCT.....T..G..C.....T.........................CT...A..C.......T..A..C..................C.......................

Tr13_Chr02|0      CACATTGAACATCAGTTTGATGCATGGCAACTGTGTAAATGGGTTGCCAAAAAAATCACTGCAGCAAGCAAAAAGCCCAAGTGCCAAGGGCTTACATGGTGGATAACCCCTATTCAAAATCATCTTTGGTGGTGTGTGCAGACAAGTAGA
Kol_12_Nig        ......................................................................................................................................................
Kol_12_Asa        .....................................................G................................................................................................
Lv7_chr2L|0       A...AA.G...............T....TT.....C..A.T...........................T.G.C.T.............AT.....T.....................G...............................G
Lv8_chr2S|0       A...AA.G.........C.....C.....C.....C....T..................A........T.G.C.T..........A..AT.....T.....................................................G

Tr13_Chr02|0      CAAAATCCTGATGTACTGATTGATAAGTGGCTATCCGTTTTGTTTCATATTGCTGATGTACACAATTTCCCAAGGTTAAAACATTACAAAAGGTGTATTCATACAACATTAACTCCACAAAAGAGAAAAGAAATTAAATGGATAAAGCCA
Kol_12_Nig        ......................................................................................................................................................
Kol_12_Asa        ..............................T....T..............................................................G.................G...............G.A...............
Lv7_chr2L|0       ........A........CG............A............C.............................A.................A.....G....G...G........................G.C.............T.
Lv8_chr2S|0       ........A........C.............A............C...................G...............C.................G....G...T..C.................G...G.C..G............

Tr13_Chr02|0      AGCCATCCAGCACACAGAGCCCTGGAGAAAATTGTAAGCGACCCAGCACTTCTTAAAGATTTGCAACACATCACCCAGATCAGTCACACCCCACAATTCGAAGTGTATCGTAGCAAGTCCTTGAAATATAGATCTAAAAACTACTCCTAC
Kol_12_Nig        ......................................................................................................................................................
Kol_12_Asa        ......................................................................................................................................................
Lv7_chr2L|0       ..............T........T..............T.................G.......G..........A..........T..............G..A..........T...............G.....G.GT........T
Lv8_chr2S|0       .A.....................T..............................A.G.......G..........A.....G...T..............G..C................................G.GT........T

Tr13_Chr02|0      AAAATAGAATCGGCATATGCAAGAACTATATTGGCAATCCTTTCAAACAACCACAGTGTGGGCAAAGAACGGACAGCATTAAGGAGTCCATCAAAATCTGACCTGCCAGTAGTGGAAAAATGCTGCAAGACAGTTTTTTCCAAGCAGGAA
Kol_12_Nig        ......................................................................................................................................................
Kol_12_Asa        ........C....................................................................................................AT.......................................
Lv7_chr2L|0       .....G..C.....T...........C...................................T.G...G.A.---C..A.G---.C................T......CCAGT.G....A...A.C.A.C.....AC.T.T........
Lv8_chr2S|0       .....G..C..C..T.....T.....G...........T..G....................T.G...GA..---C..A.G------..CTG..........T......CAGA.......A...A.C.A.C..G..AC...T........

Tr13_Chr02|0      AAAGACTGG---GTGGCAACACCCATTTACAAACAGGGTGTAGATAGCCATCTGTTCAAACTAATGAATGACTGCTTGAGCATAATGAGCGGAGACGTTCTTTGTGAATGA
Kol_12_Nig        ...............................................................................................................
Kol_12_Asa        ....................................................................C..........................................
Lv7_chr2L|0       .TG.G.---AGG....T...G..........G...A........G.A.........T..G...T.........................T......A....C.TA---...
Lv8_chr2S|0       ..GA.....CGA------.....G.......G...A....G..G.A.......A.....G...CA........................T......A....C.CA---...
```

```
Tr21_Chr04|9   ATGCCTTGCTGCATAGTTAAACAGTGCCATAGCTGGACCGGTAGAAAAAAAGTAAAGGAAAACGTGATTCTTCATGTTTTTCCGAAAAACTTTTCTAGGATTAAAGAATGGCTTGCAAAAACGCAACAATATTCAGCTGAGGACATTGAA
Tr22_Chr01|9   ......................................................................................................................................................
Tr23_Chr03|9   ......................................................................................................................................................
Kol_13_Nig     ......................................................................................................................................................
Kol_13_Asa     ......................................................................................................................................................

Tr21_Chr04|9   GTTTTGTCCCAGAAAATAATGCAAACAAAAAAAAATGATAACTATAGGATCTGTTCTAAACACTTTACAGATGACAGTTATCTCATTAAAGGTGATAAAAAGGTCCTTCTTCCTTCTGCAGTTCCAACATTGTTTGGACATGACTCATTC
Tr22_Chr01|9   ......................................................................................................................................................
Tr23_Chr03|9   ......................................................................................................................................................
Kol_13_Nig     ......................................................................................................................................................
Kol_13_Asa     ......................................................................................................................................................

Tr21_Chr04|9   CAGAGGCATAGTTATAGTTCTTCAACCTGTTCATTGGAAGAGTCATCTGGAACAGAAACTAATCAGCCTGTAGCCGGGCCTTCCAAGCGTAAACGTAGTAATAAGTCAAAAAAAATCCTAATTGAGGCATCCACTCAAACTGATAATGTT
Tr22_Chr01|9   ......................................................................................................................................................
Tr23_Chr03|9   ......................................................................................................................................................
Kol_13_Nig     ......................................................................................................................................................
Kol_13_Asa     ......................................................................................................................................................

Tr21_Chr04|9   TGTGCAGACCAGGGAACACAAGTCCCTGAATTTCAATGGACACAGGCAGAACCTGAAGCTGTAGGAAAGGATCACAATTATACTTTTTTACAGTCAACTCCAATAAAAAGTCACATAGACAAGCATGCATCAATACCAACCACGGTTACG
Tr22_Chr01|9   ......................................................................................................................................................
Tr23_Chr03|9   ......................................................................................................................................................
Kol_13_Nig     ......................................................................................................................................................
Kol_13_Asa     ......................................................................................................................................................

Tr21_Chr04|9   CCTATTAAACCCACAGATCCCATGTCCAATTTACAGTTAGAACATGACAGTTCACTAAATACTGAAAATCTGGATACAGATGACCTATTTGTCAGTGACATTTCCAATCTTTATGACCCGAACGATTCAGATTTTATTGTAGGGAAAGAC
Tr22_Chr01|9   ............................................................T.........................................................................................
Tr23_Chr03|9   ......................................................................................................................................................
Kol_13_Nig     ............................................................T.........................................................................................
Kol_13_Asa     ............................................................T.........................................................................................

Tr21_Chr04|9   ACCCTGGAACACATTTCTGTAGATGAACTTGATTCTACTTTAGAATCCAGGGTACCCGATGTAAAAAAATCCTCCATTGAAGACCGTAAATTTATTGTGTTTGAAACGTGCTTGGATAGTTTATTTTATAAAGTCCAATGTCAGGCTTCT
Tr22_Chr01|9   ......................................................................................................................................................
Tr23_Chr03|9   ......................................................................................................................................................
Kol_13_Nig     ......................................................................................................................................................
Kol_13_Asa     ......................................................................................................................................................

Tr21_Chr04|9   GAATGTAACTGCCTTGTGACCCGCTTTAGAAAAATTACAGAAGGGTCCTATTTGACTGTTACCGGATATTGTGCGGTTGGCCATCGTTTCAAATTGTTTGAAAGTCAGCCAAAAGTAAATAATTACAGCACCGGAAACATATTATTAGCT
Tr22_Chr01|9   ......................................................................................................................................................
Tr23_Chr03|9   ......................................................................................................................................................
Kol_13_Nig     ......................................................................................................................................................
Kol_13_Asa     ......................................................................................................................................................

Tr21_Chr04|9   GCTGCAGTTCTATTCACCGGGTGCAACATTAACAGGATTCATGAATTTTTAGATATTTTAGGGGTGCAACAGATTTCCCAAAGAACATTTTTTCGTTATCAACGAAAATTTTTATTCAAAAGTGTTCATCATTTCTGGGAAAAAGAGCGT
Tr22_Chr01|9   ......................................................................................................................................................
Tr23_Chr03|9   ......................................................................................................................................................
Kol_13_Nig     ......................................................................................................................................................
Kol_13_Asa     ......................................................................................................................................................

Tr21_Chr04|9   AAGCTAATAAGAGAGAACATTGGAAAGCACCCTGTTGCACTTGCTGGTGATGGCCAGTGTGATAGCCCCGGTTTCACAGCAAAATATTGCACCTATAGCTTCAGTGATCTTGCCACTAATAAAATCGTGGACTTTGAAATAGTGCAGCGA
Tr22_Chr01|9   ......................................................................................................................................................
Tr23_Chr03|9   ......................................................................................................................................................
Kol_13_Nig     ......................................................................................................................................................
Kol_13_Asa     ......................................................................................................................................................

Tr21_Chr04|9   AGTGAAACATCTTCCTCCAATGCCATGGAAAACGTAGCATTTCAAAGATGCCTTGACAGAATAATCCAAGAAGGTATTGATGTGTGTGTAGTTGCAACAGACAAGCACCCCAGTATAAAAAAAACATTGGAAAGTGACAAATACACCATA
Tr22_Chr01|9   ......................................................................................................................................................
Tr23_Chr03|9   ......................................................................................................................................................
Kol_13_Nig     ......................................................................................................................................................
Kol_13_Asa     ......................................................................................................................................................

Tr21_Chr04|9   AATCATCAACTAGACATTTGGCATTATGCTAAGAACATCAGAAAAAAAAATTATAGCAGCTAGCAAACGTAAGCAATGCAAGGATTTAGGCCTTTGGGTTGAGTCAATAATTCGACATTTCTGGTGGTGTGTTCGAAACTGTCAGGGAGAT
Tr22_Chr01|9   .......................................C..............................................................................................................
Tr23_Chr03|9   .......................................C..............................................................................................................
Kol_13_Nig     ......................................................................................................................................................
Kol_13_Asa     ......................................................................................................................................................

Tr21_Chr04|9   GAAGAACTGCTGAGGGAAATGTGGGTCTCAGTTCTTCACCACACAGTCAACCAGCACAGATGGAGAGGAAGTAGAAAATACTTCAAATGCTCCCATGGCAGATTATCAAAAGAGCAATCAGAATCCAAAGAGTGGCTTCTAAAAGACAGT
Tr22_Chr01|9   ......................................................................................................................................................
Tr23_Chr03|9   ......................................................................................................................................................
Kol_13_Nig     ......................................................................................................................................................
Kol_13_Asa     ......................................................................................................................................................

Tr21_Chr04|9   GCAGCCTATCTACAACTAACAGATATAGTTACCAATCCGATACTTATAGGTGACTTACAAAGATTGGTGAACAACTGTCACACAGGAATGTTGGAATGCTACCATTCCAAGATGTTAAAATTTAGGACAAAACGCATCCATTTTACAATG
Tr22_Chr01|9   ......................................................................................................................................................
Tr23_Chr03|9   ......................................................................................................................................................
Kol_13_Nig     ......................................................................................................................................................
Kol_13_Asa     ......................................................................................................................................................

Tr21_Chr04|9   GATTCTATGGTAGCTAGAACGTCTCTCGCAGTCCTCTCTCATAATTTCAACACTAACAGGCAGCAGGCTGTTGTAAAACGCCAAACCACAGGCGGAGCACAAGTGGGGGAAAAGCGTACAAGCCTAGTCTTCCCACGATCCAGAAAACGG
Tr22_Chr01|9   ......................................................................................................................................................
Tr23_Chr03|9   ......................................................................................................................................................
Kol_13_Nig     ......................................................................................................................................................
Kol_13_Asa     ......................................................................................................................................................

Tr21_Chr04|9   TGGGTTGTTCGTACAATTTATGAAAAAACATCAAATGAACACCTTTTCCATCTTATGGAAGACCTGTTAAATATTGTGGAAGGGAATTTACTTACCACATATGAGTCTGTGACAGACACTTTGCCCAGAAATCTAGCAACTGTTGAGAAA
Tr22_Chr01|9   ......................................................................................................................................................
Tr23_Chr03|9   ......................................................................................................................................................
Kol_13_Nig     ......................................................................................................................................................
Kol_13_Asa     ......................................................................................................................................................

Tr21_Chr04|9   CCAGATAAAGCATCTGCAGTGGCAGAACATCAATCAAGGTTCTGA
Tr22_Chr01|9   .............................................
Tr23_Chr03|9   .............................................
Kol_13_Nig     .............................................
Kol_13_Asa     .............................................
```

```
Tr27_scaffold_4830|5 ATGCCAAAGTGTTTGGTCAAAAATTGTCCACACAAGACAGGAAATAAAGCATTATACCCAGAAGTGGTATTACACGTTTTTCCTAGAAACTTGGACAGAATAAAAATATGGCTAAATTTCACGGGTCAGGATTTTCCCGATTTCGACGGA
Kol_14_Nig           ......................................................................................................................................................
Kol_14_Asa           ......................................................................................................................................................

Tr27_scaffold_4830|5 TTTGTTCAACAAATTTTCAGTTCAAAGAAAATAGATAATTACAGAATATGTTCCGTACATTTCCCTGAAACGTCATATATCTTGAAAGGAACCAGAAGATTACTTCGACAGGATGCAATGCCAACTCTCCAATTTGACAATACTTCATTT
Kol_14_Nig           ......................................................................................................................................................
Kol_14_Asa           ......................................................................................................................................................

Tr27_scaffold_4830|5 TCAGAATCGTGGATGACTAAACGCCCACTACAAAGTTTTTCAACGGAAGTTCTGCCTTCGACCTCAACTTCATTACAAGAACCATGCAATTGTTCATGTCACCGGAGTGATATATCAACAGCTACGCAAGAAACTCAAACTGATCCAACT
Kol_14_Nig           ......................................................................................................................................................
Kol_14_Asa           ......................................................................................................................................................

Tr27_scaffold_4830|5 ATGTTCGTTAACATCCCAGAAACACAGATTCCAGCCTTTCAAAGATCAAGTTTGGCTCAGGAAATAGTAGTGAATCTGGATCATTCGTATGCTCGTCATCTTCCAGTACGTGAATATGCTCAGTCAACTCCACATAAAAGGAAATCAAGA
Kol_14_Nig           ......................................................................................................................................................
Kol_14_Asa           ......................................................................................................................................................

Tr27_scaffold_4830|5 AAAACTTTGCAGTTGGAAATAACACCAATTAGTCCTGGAATAAATCCACCAATACTTGAGGAAAGTGAGGAAGAACTCATTCCAATGGATACATCTGATGATGAAAGAAATGACAGTTCTCATGGTATAGTTCCTTTAGGTTCAGATAGT
Kol_14_Nig           ......................................................................................................................................................
Kol_14_Asa           ..................................................G.............................A..........A..........................................................

Tr27_scaffold_4830|5 CATCAACCCGTGAGTGAAGACCTACAGACTACTTTTGAAGGATCCACTATACAAACTCCTGCAAAAGATCAAAAGGATGAAACATATTATCCTGACACTTCCCCATCAAATGCCTCATTTTTTATACAAGGTGAGGAGACAAACATAAAC
Kol_14_Nig           ......................................................................................................................................................
Kol_14_Asa           ........A..................................................C.G.......A...............................A..................G.C...........................

Tr27_scaffold_4830|5 AAATCTATGTCTACCGAAGAACAGATGGTAGAAGAGCAGAAATTTATTGTATTTGAAAGTTGCCTGGACAAACTTATTTATATGATAACTTGTCAGTCTTCGCTGGGTTGTATGGCTAAAATAGAACACATAATTAAAAGGTTTAAAGGT
Kol_14_Nig           ......................................................................................................................................................
Kol_14_Asa           .............TT........A...............................................................T.............A..........C..................C........A...G.....

Tr27_scaffold_4830|5 TCGCTTGTAAAAATTCGAGGTGTTTGTTTTGAAGGACACAACTTCCCTCTCTGGGAAAGCCAGCCTATGATAAATAATTTTGCAGCTGGAAATCTTTTGTTAGCATCATCCATCATTCTTAGTGGATCCAGTTACCAAAAAGTGCATGAT
Kol_14_Nig           ......................................................................................................................................................
Kol_14_Asa           ...............................................................................................G.....T...G....A.................A.....................

Tr27_scaffold_4830|5 TTGTGCAATATTTTTGGATTAAAACATTTTTCTGAGAGCACATTTTATCGTTATCAGAAAAGATTTATTTTCCCTGTTATTGACTTGCATTGGATTACTGAAAGAAGCAAGGTCAAACATGATTTAACGGGACAGGCACTATGTGTGGGA
Kol_14_Nig           ......................................................................................................................................................
Kol_14_Asa           ..............................................................G......................................G.................C......T.T........TT...........

Tr27_scaffold_4830|5 GGAGATGGACAATGTGATAGTCCAGGGCATAGTGCCAAGTACTGCATCTATACTATGATTGACCTGACTTCAAAGAAAGTGGTAGATTTTGAGGTAGTCCAGGTTACCCAGTCCTCATCATCTGTTGCTATGGAAAAATTAGCATTTGAG
Kol_14_Nig           ......................................................................................................................................................
Kol_14_Asa           ...................................................................A...C.......................T.....A...........T.....C..............................

Tr27_scaffold_4830|5 CTGTGCCTTAACAGAATTCTCAGTGAAAACCTGGAGGTGCATATCATTGCCACAGATCGTCATCCAAGCATACGCAAATTGATGGAAGATAAATTCAAGACGATAAATCATCAGTATGATGTTTGGCACTATGCTAAATCCTTACGGAAG
Kol_14_Nig           ......................................................................................................................................................
Kol_14_Asa           ..........................................................................A.....A.....................................................................

Tr27_scaffold_4830|5 AAAATAGCAGCCGCTAGTAAGAAGAGGGCCTGTGCGGATTTGAAGTTGTGGCATAACCCCATAATTAACCACTTCTGGTGGTGTTGTAGACGATGCAAAGGAAATGAACAAGAGCTGAGAGAAAAATGGCAATCCCTGCTATATCACGTT
Kol_14_Nig           ......................................................................................................................................................
Kol_14_Asa           ....................................................................................................................A.................................

Tr27_scaffold_4830|5 CAAAACAAACATAAGTGGAAAAAGGGTAAAAAATATCAAAAATGTGCCCATTTACCTCTGTCAAATGATGAGATCCAAACAACTCAGTGGCTTGACAAGTATTCTCCAGCTTTTGCAAATTTGCAGGAGATTGTAAAAAATGATCAGATT
Kol_14_Nig           ......................................................................................................................................................
Kol_14_Asa           ...............................................................................A....................................T...............A.................

Tr27_scaffold_4830|5 GAGAAAGATCTTCCACATCTCACTTATTTTTGTCACACTGGCATCATTGAAACATACAACAGTCTATCTCTTAAATTCCGATTGAAAAGAATTCACTACGGAATTGATTCCATGGAGGCAAGAAGCAAACTCGCTGCACTCACCCACAAT
Kol_14_Nig           ......................................................................................................................................................
Kol_14_Asa           ..............................................................................A..A.................................................A..................

Tr27_scaffold_4830|5 TTTAATGTTGGAAGACAACAGGCTGTTGTCAAAGTTCAAAAACACACAGGAGAAGCAGTTGGAAACATTCAG-----------------------AACCCAAAATGG-------------------------------------CAGTTC
Kol_14_Nig           ....................................................................CAGAACCAAAATGGAGTTCAGGAAAAGC.GTAA.CG....ACTGTTCGCAATGTTTACGAAACAATGTCCATTGAA..T..T
Kol_14_Asa           .................................................C..................C.GAACCAAAATGGAGTTCAGGAAAAGC.GTAA..G....ACTGTTCGCAATGTTTACGAAACAATGTCCATTGAA..T..T

Tr27_scaffold_4830|5 AGC--------------------------------------------------------------------------------------------------------GAAAAGCAG----------------------------------
Kol_14_Nig           TCAGTGATTTCTGCTGACATTATTCGAAAGGCTAAGGGTACTCTGTCTACCTCATGGATACCTAGAGCTCCAACTGTACCGGCTAACATAGCATCTGTTCCTAGACCA.....AG..GAGGCAGTTCGTGAACATTTCTCTCGTTTCACT
Kol_14_Asa           TT.GTGATTTCTGCTGACATCATTCGAAAGGCAAAGGGTACTCTGTCTACCTCATGGATATCTAGAGCTCCAACTGTACCGGCTAACATAGCATCTGTTCCTAGACCA.....AG..GAGGCAGTTCGTGAACATTTCTCTCGTTTCACT

Tr27_scaffold_4830|5 ------------------TAA
Kol_14_Nig           AAATATAATCGAAATGCC...
Kol_14_Asa           AAATATACTCGAAATGCC...
```

```
Tr10_Chr08|0   ATGACAAAATGCATAGTGAAGGGTTGTCGTAACACAAGTCGGAAAAACAAACGTGACCTTGGGGTGACTTTGCATGGGTTTCCTTGTTCCATCGAGAGGATAAAGTTGTGGCTTCAGCAAATTGGACAAGATTTTGGTAACCTTGATTCC
Kol_15_Nig     ......................................................................................................................................................
Kol_15_Asa     ......................................................................................................................................................
Lv4_chr8L|0    .................A...........G...T............................A..C.......................T..TA.............A.........................G................

Tr10_Chr08|0   TATGCACAAAAAATTCTGGATACACGAAAAAGGAATATTTTTCGAATCTGTTCTGCTCATTTTGAACCTGAAAATTATATCTGTCAAGGTATGAAACTGGTTCTTAGAGCTGATGCAGTTCCAACTATTTTCCCTGCAAGCAACTGCAGA
Kol_15_Nig     ......................................................................................................................................................
Kol_15_Asa     ......................................................................................................................................................
Lv4_chr8L|0    .....G..G........T......A....G...........................................G......................T.............A......A................G..G...........G

Tr10_Chr08|0   ACAGACAGAAAAGAAGACATTGTTGTTCCACCTCCCAAGAAATTAAAACGTAAAGCTGACACTGTACAAACATCACTTGACAATATGAATCTGTCTGGAACAAATCAGACTAAT---GAAGAAGAAGAAGCTATAACCTATGCCATCCGC
Kol_15_Nig     ......................................................................................................................................................
Kol_15_Asa     ......................................................................................................................................................
Lv4_chr8L|0    G...T...T....GTA........T.C....T.T..........T........G.....T..............T.A.A.A........C.A...............A......GAA....................G...........T

Tr10_Chr08|0   AAATACATGTTGGAGAATACATTGAAGCCGATGCCCAAAGAAATGGTGGATGCCTCTACTTCCACAGATCCTAAATACTTCAATACAGATCAGGGTGTACAGTGGTCTGAAGATGAATTTAATTGTACAAAACAACCACTAATGATAAAG
Kol_15_Nig     ......................................................................................................................................................
Kol_15_Asa     ......................................................................................................................................................
Lv4_chr8L|0    G.T..T......A.........G.....T..CAT.T...A................................G.........C....A....A...........T................G....G.T.G........AG.A.....T.

Tr10_Chr08|0   CATGACCCTCCTACCACTAATATTTTCTGTACTCCATTAGCACCTAAAGAAGAACGAGGATGGGATCCAGCCTACAAGGAGGGTATTTCTAACACTGGTGGTACTATCTTATTGCCGTTGCATAAAGTTCACACCCAAGTAGATGGTTGT
Kol_15_Nig     .......................................................C.............................................C................................................
Kol_15_Asa     ......................................................................................................................................................
Lv4_chr8L|0    ..AAG.T..TT..........G.C---....T....A..TT..............C.A.............A...........ACC.A......T..A...C...G....TC....AC..A....G......CAT..........A....

Tr10_Chr08|0   TTGAATTGTGCAATGTCACAACACACATCTTCCACGGAAAATATAACATTTACTGCACCCAAAGTAATAAAGCTTGAGTCTGATTCAGAAAGTTCACCTATAACTAGAACTAAGAGCTTTATTAAAAGAGATGTTTCAGAGGCTGCTATG
Kol_15_Nig     .........................................................................................................................................G........A...
Kol_15_Asa     ......................................................................................................................................................
Lv4_chr8L|0    .............A.......TG.............AC............A........A..............................................A...A........C.....G....................G...

Tr10_Chr08|0   GCACATGAAAGGAAGTTTATTGTTTTTGAATCTTGCTTAGATGATCTATTCCTAAAACTGAGTTGTGGTTCTGGAAATGGCTGCAGAGCATGCATAACTGGACTTGAGAAATATGTTGATGGCTCATTTTTGACGGTCATTGGGCACTGC
Kol_15_Nig     ......................................................................................................................................................
Kol_15_Asa     ......................................................................................................................................................
Lv4_chr8L|0    ...........A...................................T...........A..C....................TC.C...CAT.......A......................A.T......T.A...T...........

Tr10_Chr08|0   CACAACGGACACCGTTTCCACCTGTGGCACAGCCAACCTGTAAATGGGCAGGTTGCTGTTGGAAATCTTCTAATGGCAGCTGCTTTGCTCTTTAGTGGGTCTAGCTTTCCTAAGGTAAAGGAGATGAATAAATTGTTAGGCCTTCAACAA
Kol_15_Nig     ......................................................................................................................................................
Kol_15_Asa     ......................................................................................................................................................
Lv4_chr8L|0    ..T..T........A........A.....T.................A..A...............................................C....A...............A.............................G

Tr10_Chr08|0   ATAAGTGCAGAGACATATTATGACTATCAGCAGAAATATTTATTTCCAACGGTTGATGTACACTGGCACCAAGAAAGACAACTGCTTAGGGATGCTTACATTAGCAGCCCATTGTCTCTTGCTGGGGACTGCCAGAAAAACATTCCAGGT
Kol_15_Nig     ......................................................................................................................................................
Kol_15_Asa     ...........A..........................................................................................................................................
Lv4_chr8L|0    ..............C..C........C.....A.GC.....G........A.................G.............GT....A.........T...........T.....C.......................T.........

Tr10_Chr08|0   CACTCTACTGAATACTGCAGCTACACTCTTCTTGATGTTGCCACTAAGAGAATTTTGGATTTTCAAATTGAACAGATGTCTGAGAGAACTCCTCCAATTGCTGGCGAGAAGCTGGCTTTCAAGACCTGTCTTAACAGAATATTAGATGAA
Kol_15_Nig     ......................................................................................................................................................
Kol_15_Asa     ......................................................................................................................................................
Lv4_chr8L|0    .....C..A..G.......A..........A....C...........A......C.....................CA...............T.CG......CA......T.................T.A..............A...

Tr10_Chr08|0   CAGTTTGATGTCAAAGCTGTTGCTACTGACTGTGACCCTGGCATAAAAAAAATTATGAGAAAAAAATATGGCTACCTTAAGCATGAATATGATGTTTGGCTTTATGCTCGTATTCTGAAACAACGGCTGAAATATTTGAGCAAGAGAAAA
Kol_15_Nig     ......................................................................................................................................................
Kol_15_Asa     ......................................................................................................................................................
Lv4_chr8L|0    .......................................A.G.....................................................A.....G......................AC.........C.A..........G.

Tr10_Chr08|0   GCATGTCCTGAGCTTGAAAAGTGGATTCCTACCATTACAAGCCATTTATGGTGGGCTGCCAACACCAGCCATGGAAATACTGATTTGCTCCTTGAGAGATGGCAGTCTTTACTCCCACACTTAGTAAATCAGCATAAATGGAAGGGCATG
Kol_15_Nig     ......................................................................................................................................................
Kol_15_Asa     ......................................................................................................................................................
Lv4_chr8L|0    ...........A..................G.........................C.....A.................A........G.G......G................T....A.T........A....C..........TC.

Tr10_Chr08|0   AAATTTAGCTCAGGTTGCACCCATCGGCCATTAGCAGCTAGGGAACAGAAGTCCTGCCCGTGGCTGAAAAAAGGAACTTTGGCATTTTACTCATTAAGACAAGTAATAATACATCCTCATGTGTCTAATGATTTGCTTCATTTGTCTCAG
Kol_15_Nig     ......................................................................................................................................................
Kol_15_Asa     ......................................................................................................................................................
Lv4_chr8L|0    .T...CT......C................C.......C........A.C.........T...................CA...C.G...........G.....G.....GA......GC...A...........G.......A..A...

Tr10_Chr08|0   ATTTCACATGCTGAGGAAGTTGAAATGTATCACAGATTTGTTTTAAAGTATCGACCAAAGTGTATGCATTTAAAAATGGATGCCATAGAAGCAATGACGAAGTTGGCAGTGTTGGCTTATAATGCAAATATACACAGGCAGAAACTAAGG
Kol_15_Nig     ......................................................................................................................................................
Kol_15_Asa     ......................................................................................................................................................
Lv4_chr8L|0    TG................A....G....................T..A.....C.......A.........G..........................A...C......CT........C................AC......T.....

Tr10_Chr08|0   TGTTTTTCTGTAAAAAAAGGAAAAGGTGTGTTTGGCACAGTGACTCATGTCCGCAAATGCCGACTTGCCAAACCTCTGTACCATGAAGCCTCCAGTGAACATGTTATACCTATGATGACAGATGTAATTAAAATATCGTCAGGCAAGCTA
Kol_15_Nig     ......................................................................................................................................................
Kol_15_Asa     ......................................................................................................................................................
Lv4_chr8L|0    ........A.C..T............A..C..............A...............A.............CG.......C..C........C.......................T.................TA....T......

Tr10_Chr08|0   TGTCACAGCTGGTTTCCTCGGGTTACCACTGCCCTTAGGCCTTAG
Kol_15_Nig     .............................................
Kol_15_Asa     .............................................
Lv4_chr8L|0    CAG............T....................TCA.....A
```

```
Tr26_Chr09|0    ATGCCAAAGTGTATAGTCCGAAGTTGCCCTCACAAGAGTGGAAAAAAGATTCAGTACCCTGATGTGATTCTGCACGTATTCCCCAAGAACTTTGAACGAATTAAGAGATGGCTTCAACAGACCGGTCAGGAATTCAAAGATCTGGATAGA
Kol_16_Nig      ......................................................................................................................................................
Kol_16_Asa      .....................................................................................................A................................................

Tr26_Chr09|0    TACGCGCAGTGCATTCTGGAAGAGAAGAAAAATGACAATTATCGCATGTGCTCTGACCACTTCGCCCCGGAATGTTACACATTTAGAGGAAGCACTAAGGCTCTGAGGGAAGATGCGGTGCCCACTATATTTCCCGATGCGAATGGGAGA
Kol_16_Nig      ......................................................................................................................................................
Kol_16_Asa      ......................................................................................................................................................

Tr26_Chr09|0    CCAATGATAAAAGAAAGCAACTTCAATCGATCGTATGCTAAAAAAATACGACTGCAGGCCCAATTAGCAGAGACGCAAGCATCAAATTCTCAGAACACTGAGGTACCAGCTGCATTCATGAGCCATGCAGATTCCTTAGCCAGTTATTTA
Kol_16_Nig      ......................................................................................................................................................
Kol_16_Asa      ......................................................................................................................................................

Tr26_Chr09|0    GCTGGCAAAAGGAGAGATGCAAGCACTCAAACAGAGGGACATGGCGTTGCCACAGACCAAGCCGCTGAGTTTCCAGAAATTGTGATGCCAACAAATGCTGAGCCTGACCTCTGTAACAGTAACATGATTAAAGAGTTAATGGGTGAGAAG
Kol_16_Nig      ......................................................................................................................................................
Kol_16_Asa      ......................................................................................................................................................

Tr26_Chr09|0    ATCCCTTTGCATGATAAGCAAACATCTTGTCTTGGCAGGAACTGTTCTGTTGGGGGGTCCACTGTAACCTCTGTGCAGTCACAAAAGAGGAAAGAATCTTCTCTCCCGTTGGAAACTACTCATGAAGTCAGTGTGACCATTGAAGACATT
Kol_16_Nig      ......................................................................................................................................................
Kol_16_Asa      ..........................A...........................................................................................................................

Tr26_Chr09|0    TCAGAAGAGGATATAGTGCAAGAAAGGAAATTTTTGGTCTTCGAGTCATGCCTGGATACCTTGTTCTATAAACTCACGTGCGGTGCCGGTGTAGGCTGCACGTCTCCAGTCGGGAGCTTCAGAAAACATGTCGACGGCTCATTTCTCTCA
Kol_16_Nig      ......................................................................................................................................................
Kol_16_Asa      ................................................................................T...................................................................T.

Tr26_Chr09|0    GTAACTGGGTGTTGTGTGAATGGGCATCAATTCCAGTTGTGGCAAAGCCAGCCATACTTGGAACAGGTTGCTGCTGGGAATCTTCTGTTATCGGCTGCCATTCTCTTTAGTGGCTCTAATTTTAAGCAAGTCCATGAAATGAATAACCTC
Kol_16_Nig      ......................................................................................................................................................
Kol_16_Asa      ..T...................................................................................................................................................

Tr26_Chr09|0    TTGGGGTTGCAGCAGATATCCAGAACTGATTATTTGAAACATCAAAAGGAATTTCTCTTTGGGACTATTGACCATCACTGGCTTCTGGAGCAGCAGAGAGTCAAGGAAGAAATTGGCAAAAAAGCTTTGTGTATTGTTGCGGATGAGATG
Kol_16_Nig      ......................................................................................................................................................
Kol_16_Asa      ...............................................A......................................................................................................

Tr26_Chr09|0    TACAGCAGCTCCAAGCACACAGCGAAACATTGTTCCTATGCAATGATAGATCAGGCTACACAAAAGGTTGTTGATTTCAAAGCTTCTCCCCTTGCCCGGATTAGGTCTTTAACTGTGAGAAGGGCTTTTCAGAATTGCCTTGACCGGATA
Kol_16_Nig      .....................................................................................G................................................................
Kol_16_Asa      .....................................................................................G................................................................

Tr26_Chr09|0    CTGGATGACAAATTTGATGTGCATGCTGTTGCTACAGATTGCCACCCTGGAATAGAGAGATTAATGCGAGAGAAGTATGTGGCCATTAACCATCAGTATAAAGTCTGCAGTTATTCTAAAGAACTAAAGAAGCGCCTGATCCTGGCCAGT
Kol_16_Nig      ...............................................................................................A......................................................
Kol_16_Asa      ......................................................................................................................................................

Tr26_Chr09|0    AAGAAAAAGATGAGTGAAAAAATTGAGTTATGGATAAATCCCATCATCAACCATTTTTGGTGGAGCATTAAAACTTGCGGTGGGGATGGAAACATGCTTCGTGAAAGGTGGCAGTCTCTTCTATATCACCTCACAAACCAGCATAACTGG
Kol_16_Nig      ......................................................................................................................................................
Kol_16_Asa      .................................G....................................................................................................................

Tr26_Chr09|0    GTGGATTTAGACCTGTATAGTTCTTGTTCCCATAAGCCACTAACGTGGAAGGAGAGGAAGGCCACCCCCTGGCTGAAAAAAAAGAGCCTAGCCTATAAGCATCTTGTGGAAGTTGTAAAAGACCCACAGATGGAAAAAGACTTTCCTCAT
Kol_16_Nig      ......................................................................................................................................................
Kol_16_Asa      ..........................................................................A.........................................T.................................

Tr26_Chr09|0    CTGTCTTGCTACTGCCACACTGGTTACGTTGTTAGCTTCCATAGCTACATTCGTAAGTACAGAGCCAGTTTAGCGCTTGATATGGATTCGATGGAAGCGAGGACCAAACTGGCCGTACTTGCCCACAACCATAATATTGGTAAACAGGGC
Kol_16_Nig      ......................................................................................................................................................
Kol_16_Asa      .......................A.....C........................................................................................................................

Tr26_Chr09|0    AAAATACGAGTGCCCGTGAAGCATAAAAAATGGTTTGATATGCAAAAAGAAATGAAGATTGCCTCCAGAGCAAATAGCAGATGGCTTGTGAAAACTGTTCACGGAGAAATGTCAACCGCACATTTGAAAGGTTTGTTAACGGACCTTCTT
Kol_16_Nig      ...................................................................................................................A..................................
Kol_16_Asa      .............................G.....................................................................................A..................................

Tr26_Chr09|0    AGAATGTGCAATGGCAAACTCGTCTCCCCATGGAAAGCGAGATCTGTGCATCTTCCCCACAGTGCAAGGGCTGCACGTCGACCTGTCGCTGGAGCAAGAAGAAAATAA
Kol_16_Nig      .................................................................................................A..........
Kol_16_Asa      ..............A.............................................................................................
```

```
Tr12_Chr06|9   ATGCCCAGCTGCATTGTGAGAGGCTGCTTTCACTGCAGTGGGAAAAAGTCCTCT------------------------------------------------------------------------------------------------
Kol_17_Nig     ......................................................................................................................................................
Kol_17_Asa     ................C...T....................A............GGATTTCAAATCCACCTGCACTGAGGCCACCCCCCATTTGCGCCCCCCCCCCCTCTCCCCGGCGCGCATGCGCGAACAAAACTCCCCGCCGCGCTG

Tr12_Chr06|9   ---------------------------ACT---------------GGA---------------------------------------ATAATTATG---------CAC------------------------------ACCTTC------
Kol_17_Nig     ......................................................................................................................................................
Kol_17_Asa     CACCAATGCAAGTGCGAATGCGCGGCC.T.TAAACTCCCATACGGA.CATTGGGGAGAAGTCCCCATTGCTCCGTATGGCCACTAAATGTTAA.ATATCGTTGCGGCAGGGCGGCATGCCGCCTCTAAAATTGTGCCGC...AGGCCTGG

Tr12_Chr06|9   ---CCA------------------------------------TCATGTCTCTTGAAGATAAAAAGGTGGCTACGTCAGATAGTACAGCATACAAACCAAGATTTTGGTGACATT---------GATTGTTTTGCAGAAAAGATTTTTAAT
Kol_17_Nig     ......................................................................................................................................................
Kol_17_Asa     GCCTTTGTGGCCTCGCCACAAATCTGGGCCTGCCTCTACAGGAATAA.TATGCATCATGTCTC.T.AA.A.TAAAAG.TGGC...C...G.T.GTA..GC.AACGAACC.AGA.TTTGGTGACAT.GC...GCACA..GCAT..---T.A

Tr12_Chr06|9   ATGAAGAATTCAAATTCTTATCGTGTATGCAGTGCACACTTTACAAACGAAAGTTACAAATTGCAAAAAGGCAAGAGAAGATCACTCAAA---GCTGATGCCACACCA---ACTAGTTTCAAAATTCCGATAACAAATGTTTACAGA---
Kol_17_Nig     ......................................................................................................................................................
Kol_17_Asa     ..A------CA..GAAT.C.AAT.CCTATTGTG------.A.G..GTGCTC.C..TAC..ACAA..GCTACA..TT.C.A.AAGGCA.G.GAA.A.C.CTT..AG.TGATGC.ACACCAACC.T..T.A------..AT.CC.ATT.CCA

Tr12_Chr06|9   ---AACAATGCATCATCTTCAAATAGT------GAACAACATCCC---TCAGTGGCACCCACACAAGCTGCAAAAGTGGAT---AAAGCTGTGCAATGGCCAGAATATGAGTTCAACACTGAAGGGGAACAGTGGAAGGTTGAACATGAT
Kol_17_Nig     ......................................................................................................................................................
Kol_17_Asa     AACGTGC.CT..AT.ATACATC..C..CAAATA.TGA.CAGCAT.CTTCAGTG.CAC..ACACA.GCTACA..GGT..ATGCTAGC.CA.T.A..G------------AT.CA.....T.C...A.TTCA.CACTG.A.GG.....GTGG

Tr12_Chr06|9   CATGTGTATGAAACACAACCAAGAATAATACAGTCCACGCCAATGAAAAGGTTTCAACACCCACAGCCATCAGTGGATGTTGGCTCCCTGAATGAC---------------------TTTGAACGAGAT---------------------
Kol_17_Nig     ......................................................................................................................................................
Kol_17_Asa     ATG..TG.AC.TGAT..TTTGTATGA..C...AC.A.GAA..C.A..GTCAAC..C.ATGAA.AG.TTTCA.CACCTACAGCCA..AG..G...TTGGCTCCCTGGATGATGCTGAGA.GCTGAAC..GATATTTTACAAACAGACACAA

Tr12_Chr06|9   ---------ATTTTAACAGAC---------------TCAATGTCACCAAACCTCCACAGC---TCTGTACATGATGACCTTAGTTGTACAGTAACATCAAATTACTCCAGAGCTTCTGAC------------ACTGAG---TAT------
Kol_17_Nig     ......................................................................................................................................................
Kol_17_Asa     TGTCACTGA.CC.CC....CTTTGTGCATGATGACC...G.TGT..AGG.A.AT..A.TTACTC.A.AG.T.CTGACA.AG...ATATTCCAG.A.ATG.GGGGTATA.TGA.....TGTAGAAGAAGACAAG.A...CTT.G.AAAAGA

Tr12_Chr06|9   ---ATTCCA------------GAAAATGATGGG---------------ACCAATGAA---------------------------------------------TCTTCG------------------------GTGACT------------
Kol_17_Nig     ......................................................................................................................................................
Kol_17_Asa     ATGGAAAT.TATTGTTTTTGA.TC.TGCC.T.ATTCATTAAAGGAGAAGAA..A.GGTTAAAAATGCCCCCCACCCATACATAGATAGGCCTCATGTCCCTG.AGCT.ATCGCTACATTTTTTTACGTTTTTTCA.G.ACTTTCAGAGCA

Tr12_Chr06|9   ---------------------GACACATCGACT---GTTGTAGAA------------------------------------------------GAAGATGAG---------CAGAGCTTTGTAAAAGAACATAAATTT------------
Kol_17_Nig     ..................................................................................................................................G...................
Kol_17_Asa     CTCACTGTTCGCTTAACTAAATG.G...GTGTGAATC..C..CCTCCCCTAGACGCGAGTGCTCCTGTTTGTGAGATTAAAAAGCGCACTCACAGTATATGTAGCTGGTCTTG.TCTGGGTGC.GGAGCG.GGC...ATGGGAATCTTCT

Tr12_Chr06|9   ------ATTGTT------------------------------------------TTTGAGTCATGC------CTTGAT---------------------------TCATTAATAAGAAAACTAACCTGCAGTTTTGAGTGCAACAACAAA
Kol_17_Nig     ......................................................................................................................................................
Kol_17_Asa     TTACAC.GCTCAACGTTTTTCTTCCTGTTTGAATTCTGATCATCTGAACAGGTGAAATA.GGG.AGACTTAAGG.CACTATTGAGAGAACTGAAGGTATGCCTGCAGC.TGAC.TT..CTCTTTA.TAGCC...CCT.CTCCTTTA.T.

Tr12_Chr06|9   AATCTTTGCAATGCCCCGTTCATTGAAATTAAAAAACATTGCCAAGGCACATTTGTGTCAATTTCAGTTGCTTGCCTGAATGGACATAGCTACACATTGTGGAAAAGCCAGCCGTGCATTGGAAGAATGGCGGTTGGAAATGTTTTGAGT
Kol_17_Nig     ......................................................................................................................................................
Kol_17_Asa     .GAAAAATA.CCTG.AGA..TGAGTGT.AC..C...A..CTTTGCAATG.TCCGT.CA..GAAATTAAAAAACATTGTC.A..CACATTTGTGT.GA.T.CAGTTGTTTGC.T.AATGGACAT..CTAC..AT.GT.T..AAGCCA.CCG

Tr12_Chr06|9   TGTGCTGCATTATTATACAGTGGGTGTAATTTTTATAAGGTGCGGGACATGTTTGGTTTATTGGGACTTCAATTCATATCGCATAACACTTTTTACCGTTACCAGCGTGAGGTTCTTTTTCCAGTTATTGATGTACACTGGCAGCAAGAA
Kol_17_Nig     .................................................................................................A............................T.......................
Kol_17_Asa     ..CAT..G.AG.A.TGTAGC...AAA.GT...GAG.TCA.CTGCATTAT.ACACA..GGC.GTAATT..T.TAAGG.GAGTG.A.TGTTCGG..TATTGGGA.TTGAGTTCA.ATCGCA.AACAC.T..T.CCATT..CA..GTG...TT

Tr12_Chr06|9   CAACAAAGACTACATGACGCATTCAATGGCATAGAATTGTGCCTTGCGGGTGATGGGCAATGTGACAGCCCTGGTTACAGTGCCAAATATTGTGTTTACAGTTTTATGGAAGTGAACACTAAG---------------------AGA---
Kol_17_Nig     ......................................................................................................................................................
Kol_17_Asa     .TTTTTCC.G.TAT...T.A.CA.TGGCAGCA....CAACAAAAACTACA...CACATTCAA.TG..TAAAATTG.G.CT...AGGTG..G.GCAA---T..GAC.GACCG.GTT...G.GCCAAATATTGCAAATAGAGTTTT.TGGAA

Tr12_Chr06|9   ---------ATTGTG---------------GAC---------TTCGAAGTTGTTCAGGTTACAGAGGCT------------AAGACTTCACCAGCTATGGAGAAACTAGGATTTACAAAGTGC---CTGGAAAGAATT---TTGGCTGGA
Kol_17_Nig     ......................................................................................................................................................
Kol_17_Asa     GTGGACACT.AG.G.TATTTAAAAAGTGGA.TAAAACATCACAGT.GT.....T.T.GC.GCC.AT.AGATGCTTTGCTTTGATT...TAACTA.TGAA.TCT.ACT.CTGA.TGGTT.CC.TGGGCAAC.TC.CAGGTAA..CT.CAC

Tr12_Chr06|9   ------GCATATAAA------GTAAGAACAATAGCA---ACAGAC------CGTCATCCAGGA---------------ATTATA------------------AAA---------TTAATGCGTGATGAGTATAAAGACGAAGGCATAGCA
Kol_17_Nig     .........................................................................................................................................AA...........
Kol_17_Asa     TCCACTTTT..A.C.GCATGATA..T.TACC.CTA.GAA.ATTGTGGACTTA.AGG.TGTTC.AGTTACAGAGGCTAAGACT.CAGCAGCTATGGAAAAACT.GCATTTACAAAG.GCCTG.AA..A.T.T.GGCTGGATCATATA.AGT

Tr12_Chr06|9   CATGAGTTTGATCTGTGGCACTATGCCAAGAGTTTAAAGAAACGCTTGTTTGCAGCAAGCAAGAAGAAAAACTGTGGTGATATAGCTGAATGGATACCAGCTATAACAAACCATCTGTGGTGGTCTTCAAGCAAGTGTGAAGGTGACTTG
Kol_17_Nig     ......................................................................................................................................................
Kol_17_Asa     A.ATGCAA.AGCAACAAA.CG.CAT.ATG..TG.GTG.AGTGTA.AAAAAGAA..GC.TAGCACGTG.GTTT------------------AAT---.TGTGGCACTATGC.---AA.---------AGTTTA...AAACGCTTG------

Tr12_Chr06|9   CGTATGTTGCGGGAA------AGA------------------TGGCAA------TCTGTTCTGATG------CACATTTCTGATCAGCATGAATGGGATCATGGGATAATGTACCATGCGTGCACACACAAAAAGTACACAGATGAGGAG
Kol_17_Nig     ......................................................................................................................................................
Kol_17_Asa     ------..TTCA.C.AGCAAT.AGAGAAATTGGGCTGCTATAGCTG..CTATAACAA.CCA.CTGTGGTGGT.TTCAAGAA---------------AG.GTGAA.G.G.CT.GTGTATGT...TGGA.AG.TGGCA.TCT.TTGTGATGC

Tr12_Chr06|9   CGTAGCCAACGACCATGGATAGAAAAAGACTCACAAGCCTACTTGTCCTTGGCAGAAGTAGTCTTGTCAAAAAATATGACAAAAGACCTTGAGCACATG---GCTCATTTTCGCCATACCGGAGCATTGGAAGTCTATCATAGTTTT---
Kol_17_Nig     ......................................................................................................................................................
Kol_17_Asa     ACATAT..GAT.AGCAT..AT.GG.T.ATG.AC..T..G.G.ACACA.AAAAAGT.CAC..ATGA.GAGCGT.GCCGACG.CC.TGGA.A..AA.AGACTCACAAGTA.ACTTGTCCCTG---...GAA.T....CTGTCA.AAAAAATG

Tr12_Chr06|9   ---TCCCTA---AAGTAC---------CGGCCT---AAAAGGATCCATTTCAAAATGGATGGAATGGAGGCCCGTACAAAACTTGCAGCTCTAGCCCATAATGCAAATGTACACAGAGAGCGATCTCGTGTCCGTTGTGCTGGTGAGGGA
Kol_17_Nig     ......................................................................................................................................................
Kol_17_Asa     ACAGAAGACCTTG..C..ATTTTCGCCATA.TAGAGC.TTA..AGTG.A...C.G.T------.GTACC.G..TA.A.GG.---T.CAT.TC.AAATGG.TG.TTTTGAGG---CCC.TA------------------------------

Tr12_Chr06|9   AGGGACCCCGTTGGCTCACTTCGTCAT---------AAACTTGTGTTCCCAAAATTCAAAAAAAAA------TGGGTTACTAAAGTACTGTATGAGCCTACAACCAATGAACACATAATGCCAATGTTAGTGGATGTGCTCAAATTACTT
Kol_17_Nig     ......................................................................................................................................................
Kol_17_Asa     ---C.AAA.T.G------.AG.TCT.GCCCATAATGC..A.GTACACAGAG.GCAATCTTGTGTCTGATGGTCT...G.GGG...AGACACTGTT.G..CACTTTGTCAT.A..T.G------------TGTTCCCAAAAT.C.AA.AAA

Tr12_Chr06|9   CAGGGCAAATTG---TCTCATAGTTGGGATTCAAGAAACAGCACTATGCCAGACAACATTGCCACA---GTACAGAGACCAGATAAAAGTTTGGCA---GTTCAGCAACACATGTCAAGATTTCAAGATCGGGACATG------------
Kol_17_Nig     ..........................................................................................................................................TAAACCCAGTAC
Kol_17_Asa     A.AA.GGGT.ACTAAAGCAC..TA..A.CCAAC.ACC.AT....ATGTAATTC...TG..AGTGG.TGT.CT..A.TTA.TTCAGGGCAAA.T.TCTCATAGTT.------GGA.TCCACAAA---C.G.AC------............

Tr12_Chr06|9   ------------------------------------------------------------------------------------TAA---------------------------------------------------------------
Kol_17_Nig     CATCTAACAAGCTTTTTAGGA...AAAATCTTCCATGGTGGCAATGCAAGTTGGTGCTCCTGTGCTTTGAAAGGGCGAATTCCAGC.CANN...........................................................
Kol_17_Asa     TATGCCAGACAACATTGCCACAAAACAGAGACCAGCTAAAGGTTTGACAGTTCTGCAGCATATGTCAAGATTTCAAGATTG...GG.CGTGTAAACAAGCTTTTTGGTAAAATCATCATCCACGATGGCAATGCAAGTTGGTGCTCCTGT

Tr12_Chr06|9   ----------------------------
Kol_17_Nig     ............................
Kol_17_Asa     GCTTTGAAAGGGCGAATTCXXXXGATAT
```

```
Tr34_scaffold_731|0  ------------------------------------------------------------------------------------------------------------------------------------------------------
Kol_18_Nig           ATGCCGAAGTGCATCGTTTCCCGCTGCCCCAGCTCGTGTTCCAACAAACAGACCAAATCTACGGGGATCGTGATGCACTCGTTCCCCTGCAACCTGGACAGAATCAAGAACTGGCTGCTGAACATTGACCAGAACTTTGGGAACATAGAC
Kol_18_Asa           ATGCCGAAGTGCATCGTTTCCCGCTGCCCAAGCTCGTGTTCCAACAAACAGACCAAATCTACGGGGATCGTGATGCACTCGTTCCCCTGTAACCTGGACAGAATCAAGAACTGGCTGCTGAACATTGACCAGAACTTTGGGAACATAGAC

Tr34_scaffold_731|0  ---------------------------------------------------------------------------------------------------------ATGAGCCTCCGACCCGACGCCATTCCCACTGTCTTCAAAGAGCGG
Kol_18_Nig           ACGTTCGCCAGTCGGATCCTGGAGGAAAAGAAGAAGCATTCGGACCTGTATCGCCTGTGCTCGGAACACTTCACCCCAGAAAGTTACATCAACACCGGCAAAAGG.............................................
Kol_18_Asa           ACGTTCGCCAGTCGGATCCTGGAGGAAAAGAAGAAGCATTCGGACCTGTATCGCCTGTGCTCGGAACACTTCACCCCAGAAAGTTACATCAACACCGGCAAAAGG.............................................

Tr34_scaffold_731|0  CCGCAGAACAGAGGGATAAAGTGCGGGCCGCCTGTCAGACGCCACAGTAGAATAACGAACCCGGAGGGGTCCAAATACGTGGATGTCGGCACCAACACCGAGCCCGTAGTAGTGACGGATATTGGCTTAATGGGCGCCAACCAGGACGGG
Kol_18_Nig           ......................................................................................................................................................
Kol_18_Asa           .....A........................................A...........................................................................C...........................

Tr34_scaffold_731|0  TCACCTAAACGCGTGGATGTCGGCACCAACACCGAGACGGTTGTGATGAAGGATATCAGCACCCGCACCGATATCTATCATTCCGTGAAGCACCGCGGCACGCACACCGACCCGTTCTGGGGCAAGAAAAATGTCAGAACCAAAACCGAT
Kol_18_Nig           ......................................................................................................................................................
Kol_18_Asa           ..G...................................................................................................................................................

Tr34_scaffold_731|0  CCACTTTTTGGCAAGAAAAATGCCAGTACCAGCACAGAACCCGCCGCTAACGGCCTTCAACCCCAAGGTGAAATTGGTTTTTCAAAACCGGCAAAGCGCAAGCGTCTGGGCGCGGACGCGGCGTCTCGTTATTCGCTGCTGTCAGACGAG
Kol_18_Nig           ......................................................................................................................................................
Kol_18_Asa           ..........................................A....C......................................................................................................

Tr34_scaffold_731|0  GCGGAGCCTGGGGTAAAGCAAACGCCTTCAAAGCGCAGATACAGAAGCACTAAAAACCCAAATTATATCCCATTGAGCCGCCCAGTGCTGGAAAGCGACACGTCCGACAGCAGCAGCGCCACCTCCGAGGACGATTCCGCGGGGAACGCG
Kol_18_Nig           ......................................................................................................................................................
Kol_18_Asa           ...................................................................................................................................A..................

Tr34_scaffold_731|0  GACGAAGCGAGCGCGGATCAGGCGGACATCTTGGTCCACGAGAGCAAGTACATTGTGTTCGAGTCGTGCCTGGACGAGCTGCTGCTGAAAATGAACTGTAGCTGCGGCCGACCAATCGCAGAGCTCATCAAAAGCGTTCAGGGGACCTTC
Kol_18_Nig           ......................................................................................................................................................
Kol_18_Asa           ............A.........................T...............................................................................................................

Tr34_scaffold_731|0  CTGTCCGTGTCGGGGCGCTGCGAGGCCGGCCATGTCCGCCATATGTGGGACAGCCAGCCCAAAAACGGAGGGACCCCCGCCGGAAACATCTTGTGTTCCGCCGCCATTTTATTCAGCGGCAGCCGCTTCCACCAAGTCGACGAACTGTTC
Kol_18_Nig           ......................................................................................................................................................
Kol_18_Asa           ...................................................................................................................................G..............A...

Tr34_scaffold_731|0  CGATTCATGGGGCTGCAGTTCATCGCTCAGTACGCTTACTACCAATACGAGAAGCGATTCCTCTTCCCCGTTCTGGAACGTTCCTGGCAAAGTGAGCGCCGGGCGCTGAGGCAAAGCCTGGCGAGGAAATCCGTCTGCCTGAGCGGCGAT
Kol_18_Nig           ......................................................................................................................................................
Kol_18_Asa           ..C................................C................................T.....A.................C.........................................................

Tr34_scaffold_731|0  GGGCGGGCGAGCAGCAGGCCGGGCAGCAAGTACTGCGCCTACGCCTTCCTGGAGGCCACCGGCAAAAAAATAGTCGACTTCAGCATCGTGAAGAAAACGGAAAAGCAGTCGATATCCGCCGCGAAGAGACACGCCTTCGAGACCTGTCTG
Kol_18_Nig           ......................................................................................................................................................
Kol_18_Asa           ...................................................................................................................................T..................

Tr34_scaffold_731|0  AACAGCGTGTTGGCGGAGAAGCTGAACGTGGAGGTGGTCGCCACGGAGCGGCACGAGGGGATACGGAGGTTAACGTGCGAGGAGTTCTCCCCTATTAGCCACGAGTATGACGCCTGGCATTACGGCAGAGCCATACGGAGGAAACTCCTC
Kol_18_Nig           ......................................................................................................................................................
Kol_18_Asa           ......................................................................................................................................................

Tr34_scaffold_731|0  GCAGCCAGTAGGAAGAGAAGCTGCGGCGACATCGCTGCCTGGACGCCGGCCGTTATTAACCACCTGTGGTACGCGGCCAAGTGCAGCGCCGGGGACGGGGATTTACTCCGAGAGAAATGGCGGTCCATCCTGCGCCACGTCAGGAACGAG
Kol_18_Nig           ......................................................................................................................................................
Kol_18_Asa           ......................................................................................................................................................

Tr34_scaffold_731|0  CATCAGTGGACTAACGGACTCTCCGCCCATTCCTGCGGCCACCGGAGACTTAACCTCTTAGAGTCCAAGCAGCGCCGCTGGCTAAAGGTCGACTCCCGCGCGTATCAGCAACTGAAGAAAATCGTCACGGACCCCTTGGTTCTGGGGGAC
Kol_18_Nig           ......................................................................................................................................................
Kol_18_Asa           ......................................................................................................................................................

Tr34_scaffold_731|0  CTGACCCACCTCAGTTCTCTCAGCCACACGGATCAGATCGAGGTTTTCCACAGTTTCATGTTAAAGTATCAGCCCAGAAGGGCCCACGTGAGCCTGGAGGCCATGGAGGCAAGTACCAAACTAGCGGCGCTGGCCCACAATGCCAACGTC
Kol_18_Nig           ......................................................................................................................................................
Kol_18_Asa           ......................................................................................................................................................

Tr34_scaffold_731|0  CACCGGCACCAGTCCGTGGCTAGAATTCCGGGTCGGAATATAGGCTTCCATTCCAAAACCAGGAAACCTTGGACTACCAAGAGGATATACGATCCGGAATCCTCGGCACATTTGTTCCCCGTGTTAGTGAGCGCTCTGAAACTGGCCAAT
Kol_18_Nig           ......................................................................................................................................................
Kol_18_Asa           ......................................................................................................................................................

Tr34_scaffold_731|0  GGGGAGCCGAGCCACAGCTGGGGGGCACGTGAAACGCGGAAAAACCGATAA
Kol_18_Nig           ...................................................
Kol_18_Asa           ...................T............TG........G........
```

```
Tr2_Chr02|3     ATGCCAAAGTACATTATACATCATTGCAAACATGGATCCGCCAGAAATAAGAAACCATCTAGCATTATTATGCATGTTTTCCCTACGGATATAAATAGAATAAAATCTTGGCTATTAGCAACAGGCCAGAACTATGGGGATCTAGATGCC
Tr35_Chr05|3    ------------------------------------------------------------------------------------------------------------------------------------------------------
Kol_19_Nig      ..........G.....GT....................................................................................................................................
Kol_19_Asa      ..........G.....GT.............................................................................................................TT---.GCA..A.AAGTAC.AAG

Tr2_Chr02|3     TTTGCAGAAAAAGTACTAAGTGGGAAAAAAAAATGATCTATACAGACTGTGTTCAGCTCATTTTACAGATGAATCATATATTTGT------GAAGGACAAAAGAAAAAGTTAAAAAAAAATGCAATACCAAGTGTATTCCTTTCTTCAGCT
Tr35_Chr05|3    -------------------------------------------------------------------------------------.....------------------------------------------------------------
Kol_19_Nig      .......G...............................................................T.......................T......................................................
Kol_19_Asa      .GG.A.A.....TG.TCT.TACA..CTGTGTTGAGCTC..TTTACAGA..A------------------------.TC..A.A.TTGTGAAGGTC.A..G.A...GTTAA......TGCAAT.CCAAG------.GTA..C..------

Tr2_Chr02|3     TCAGTAGATCAATCACCTGACTTATCAGCAGGCATGGAAACTTTGCAAGCTTCCGGCCATGTTGTTTCC------AAAACATCATTCACAGAAGCAACAATGGTTGAATCA--------------GCTTTCCAAACCAAAAATGCCAAA
Tr35_Chr05|3    ---------------------------------------------------------------------......------------------------...................................................
Kol_19_Nig      A...C..........T...................................................................................C..................................................
Kol_19_Asa      ---------------------..C.TCAGCTA..GCAG.T.AA.CATCTGACTTAT.AGCAGGCA.GGAAACTTTGC..GC.TCCGGC..TGT------TGTT.CC..AACATCATTCACAGAAGCAACCATGGTTGA.TC.GCTTTCC.

Tr2_Chr02|3     AGTAAAAAGAAGAAAAATCCAAAAAGATCTTTAGCCACCATAGAAAACAACACTTTGTGTATGCCAGAATGTAAAGAAATTGGGATAAATACTGAT---TATTTCATT------ACGAAAAAACACACTGGTTGCAATACATCAAAAACC
Tr35_Chr05|3    .........................................................................................................................................C............
Kol_19_Nig      .A...............C.................................G.....................................................................................C............
Kol_19_Asa      ---..CC.A..ATGCC.AAA.T...A.GAAGA.AAAC...A.A.G.T.TTT.GCCACCA..GAAA.C..C.CTTT------------GTGTA..CCAGAATG.AA.GAAATTGGGAT...T.CTGA---TTA.TTC..TACGA.....A

Tr2_Chr02|3     TTTGGAAAAAGAAATGCATCAACCTTAGCTTCAACAAAAATGAAAGATAAAAGTACAATGTGTAAT---------CTGTCTGACTTT---GGA--------------------TTTCGTGAAACTCCTATAAAGGAATGTAAATGTCTG
Tr35_Chr05|3    ......................................................................................................................................................
Kol_19_Nig      ..............C....................................................................T.................................T................................
Kol_19_Asa      CACT.GTTGCA.C.CATCAA..A.C.TTGGAA..AG...CGC.TCA.CCTT..CTTC.ACAAA...GAAAGATAAAA..ACA.TG.GTAATCTGTCTGATTTTGGATTTTGTGAAACTCCT.T.AA------GGAATG.AA.TG.C.GCA

Tr2_Chr02|3     CAATCTTCATCT------------------TCAGACAAAAAAAAA---------------ACATGTAACACTGAATTAATTCCTGAC------AATCATGTTAGTGATGTAGAT---ACAGATGAAGAAGAAATTTTATCAGATAGTGAT
Tr35_Chr05|3    .............................................................................................................................T........................
Kol_19_Nig      ....T........................................................................................................................T........................
Kol_19_Asa      ATT.TCATC.TCAGACAAAAAAAAAAACCATGT.ACACTG..TT..TTCCTGACAATCATGTT.GTG.TGTAG---A..CAGATGATGAAGAAATT.T..CAG.TA.TGA..TG.CAGTG.ATGTT.ATTATG.A.C.T..CA.CCACTTG

Tr2_Chr02|3     ATGTCA------GTGAATGATAATGATGAATCTTTCCAACCACTTGAAATC------AGTACAGATGACACAACAGAAGAAGAAAATGAAATTGAAGAACTAGATGTT---ACAATGGATACTACAGAAGATGTTGGCATTTCATTTTTA
Tr35_Chr05|3    ......................................................................................................................................................
Kol_19_Nig      ...................T....T.............................................................................................................................
Kol_19_Asa      .AA...GTACAGA...CAC.AC.GA.GA.GAAAA.GA..TTGAAGA.CTAGATGTTACAATGGATACTACAG.AG---ATGTTGGCATTTCA.---TTTTAGA.AAAGATCCTTTG.ATG..AG..CCTTCATCGTATT.GAATCA.---

Tr2_Chr02|3     GAAAAAGATCCTTTGGATGACAGCACCTTCATCGTA------TTTGAATCTTGCCTCGATAAATTGTTATGGAATGCCCACTGTGTTCAC---TAT------------CCATCT---TGTATTTCATCT------ATAAAGCGACTGAAG
Tr35_Chr05|3    ..................................................A...................................................................................................
Kol_19_Nig      ..................................................A..T..................................G...............................G.............................
Kol_19_Asa      ---------GTC.C.ATAA.TT.TTATGGA..GCCCACTGTG..C------GCTA..C..CTTG....TCATCTATAAAG.GACTGAAGAAGTC..TGTCTGGGTCATTTC...CTG.CAGAGCT..A.GTCAATC.GGTCATCA.TTCC

Tr2_Chr02|3     AAG---TCATTGTCTGGGTCATTTCTCTCTGTCAGA---GCTATATGTCAATCTGGT------CATCATTTCCATCTATGGGACAGCCAGCCCCACAAAGGTCGTTTACACTATGGTAATATTCTCATGGCTTCAGCAATTTTA---CTT
Tr35_Chr05|3    .......................................................................................................A..............................................
Kol_19_Nig      ...........................................................................................................................A.G........................
Kol_19_Asa      .TCTATGGGACAG.CA.CC.C---ACAAAG...GTTTACA....GGTAAT..TAT.ATGGCTT..G..A..T---TAC------------------------TTAG.GGGTC.AAT.TC.C.AAGGT.TA.AAT.TGT.CCAACTCCTAA

Tr2_Chr02|3     AGTGGGTCC---AATTTCTCTAAGGTTTACAATATGTAC---------CAACTCCTAAATCTG---------CACCAGATATCTCAGTCTTGTTATTACAGATATCAAAAT---ATTTTCATGTTTCCTGTAATAAACTATCATTGGGAA
Tr35_Chr05|3    ...........................................................................................C..........................................................
Kol_19_Nig      ........A..................................................................................C..........................................................
Kol_19_Asa      .TCT.CA..AGAT..C..AG.CTTC..ATT.CAGATAT.AAAATATTTTC.TGTT.CCTGTAATAAACTATCATTGG..A.AGGA.CAGA------A....C---TT.TTGAGTC....A...------------------GTAA..CC.

Tr2_Chr02|3     AAGGAACAGAAACAGCTTATTGAGTCTTTTAATGGTAAGGCCATTGCTCTCTCTGGTGACGGACAATGCGATAGCCCAGGATTTTCGGCAAAA---TACTGTATTTACACATTAATGGAACAGACAACAAAAAAAAATTATTAATTTCTGT
Tr35_Chr05|3    .....................................................................................................G................................................
Kol_19_Nig      ...........................................................T.................G........................................................................
Kol_19_Asa      TT.CTCTCTCTGGT.A.GGACA.TG.GA.AGCC---C...ATT..CGG.AA---------------------------------------.TACTGTA.T..CAC.TTA..GG.AC---AGACA.CA.......TGAT.A.T..CTGTG

Tr2_Chr02|3     GTTGAACAAATT------TCTCCACCTCAGACTTCATCACACATGGAACCAATTGCATTTGAGAAATCCCTTGTGCAACTTCAAAAGCAAAATGTAATTGTTAGGATGATCTGT---------ACCGACCGCCACTCCTCAATCCGCAGA
Tr35_Chr05|3    ......................................................................................................................................................
Kol_19_Nig      ......................................................................................................................................................
Kol_19_Asa      T.GA.CA..T..CTCCACCTCAG..T.---CA.CACA..---------TGG.ACCA...GC.T---.TGAGAAAT.CCT.GTGC..CTTC..A------AGC.AA...TAA.TGTTAGGATGAT.T.TA.CGAC.G..A.TCCT.AATCC

Tr2_Chr02|3     ATCATCAAAGATAATTATGCAAATATTAAACATCAATTTGATGTGTGGCATTTTGCAAAATCCTTGAGAGTGAAACTGTCTGCGGCATGTAAAAAGAAAAAATTCTCTGAAATCAAAGAGTGGATTTCACCAACTATA---AATCATCTG
Tr35_Chr05|3    ..............A........................................................................................A..............................................
Kol_19_Nig      ..A.....T.....A........................................................................................A......................---TA.C.A.CT....TAA...AT
Kol_19_Asa      GCAGAAT..TCA.TG------.TA.A..TG..AAT...AA.C---ATCA..----------.TGA..T.T.GC---------------AT.---------------.TGC...ATCCTTGA---------GAGTGA.ACTGTCTG.GG.AT

Tr2_Chr02|3     TGGTGGTGTGCAAGAACCTGCAATGGCAACCCAGATTTATTAAGAGAGAAATGGTTGTCC--------------------TTGTTATATCATGTAACAAATGTACATCAG---TGG---------CAAACTGGA---------CATCTC
Tr35_Chr05|3    ......................................................................................................................................................
Kol_19_Nig      CT.....GTGTGC..GAAC.TGCAATGG.AACCCAGAT...TT.AGAG.GAAA.G..TG....................CC..G.TAT..CATGT..CAAATGTA..T...CA.........TGGCAAACT.........GGA.AT
Kol_19_Asa      GTA------AT...------..AAAT.CT.TGA.A.C.AAG..T.GATTTCACCAACTATAAATCATCTGTGGTGGTGTGCAAGA.CC.GCAA.GG...CCCAGAT.T.TTAAGA.AGAAATGGTTGTC..T.TTATATCATGT.A.AA

Tr2_Chr02|3     ---TACCATGAGTGTGCACACAGTGCCCTTGCACCCCATTTGGATCATAAGAGAAAATGGATGCAAGATGAATCTACAGTTTTTGAGCAATTCAAGGACATTATTACATCACGCAAAATTCTGGATGACCTTGCCCAT------TTATCT
Tr35_Chr05|3    ................................................................................................................A.....................................
Kol_19_Nig      ...CT.T.CC.TGAGTGTGCACACAGTGCCCTTG.A.CCCATTTGG..C.T.AG.G.AAATG.ATGC.A.TTGAAT.TACAG..TTTG.GCAATTCA.GGAC....TTA..TCACGC.AAA.TCTG..TGACCTTGCC......CAT.TA
Kol_19_Asa      ATG...ATCAGTG.CAA..TGGACAT.TC.------ACC------ATG.GTGTGC.CACAG...CCTTG------CACCCCA..TG.ATCA.A---AGAGAA.A.GG..GCAAA.TG.A...AC.GTTTT.GAG.A..TCAAGGAC..TA

Tr2_Chr02|3     CATTTTTGCCAT---ACTGGAGAGTTGGAAGTTTAC---CAC---AGCAATTTGCTAAAATATAGGTCTAAGCGTCATCATTATTTTATAGATGGGATGATTGCTAGAAGCCAATTAGGAGCACTTGACCACAATTTTAATGTCCAAAGA
Tr35_Chr05|3    ...................................................C..................................................................................................
Kol_19_Nig      TC.CA..TTTGC...CA.ACT.GAGA.TTG.AAGTT...T.....CA..GCAAT..GCT.A.ATATAGGTCTAAG......C...ACT.TATA.ATGG...GAT.GCT..AAGCCA.TT..G.GCACTTG..C.CAA.TT.AATGTCCA.
Kol_19_Asa      TTACA.CA.GCAAAATTCT.GAT.ACCTTGCCCATTTAT.T.ATTTTTGCCA.A..GG.GAG.T..AAGTTTACCACAGCA---A.CTGCT.AAAT..AGG.CTA..CGT.ATCA.TACT---T.AT.GATGGGA.GATTGC.A---GA.

Tr2_Chr02|3     GAACAGGCCAGAGTTTGTACAACTGGTCCTGAATGTGACTCAGTTGGATCTTTGAGG---CAC---AAACTGGAGTTTACAAAGTCAAAAAAAGACTGGGTTGTAAAGCCAATCTACACTCGTCCTTCAAACTCATTC---CTCTTTGAA
Tr35_Chr05|3    ................................................................................G.....................................................................
Kol_19_Nig      AG.G.ACAGGCCAGAGT.TGT..AAC.GG.CTTGAATGTGACTCA.TTGGA.CTTT....AGG...C.CAAACT.GAGTTT.C.AAGTC....A.AGACTGG..TGTAAAGCCAAT.TACAC..G.C.TTCAAAC.CA...T..C.CTTT
Kol_19_Asa      .CCA.TTAGGAG---------CACTTGA.CAC.AT.TTAATGTCC---AAAGA..ACAGG.CAGAGTTTG.AC.AC.GGTCCT.AATGTG.CTC.G.T..A.C.TTGAGGC.CAA..TGG------AGTTTACG.AGTCAAAAAAAGACT

Tr2_Chr02|3     TTACTCAAGGATATCATTAGGCTTGCTTGTGGAGACATAGATATACAATGGCAGCCAGTTCGTGCT------GATCTGCCTTCTAATATTGCTTCGAAACCTAGACCGGACAAGTATGAAGCTGTTGAAAAACATCTTTCACGTTTTAAA
Tr35_Chr05|3    ........................................................................................................G.............................................
Kol_19_Nig      GA.T.ACTCA.GGAT..C.TTAGGCT.GC.T.T.GAGACATA.ATAT.CAATG..AGCCAGT.CG.......C.GA..TGC..TC..A.AT.G.TTCGAAACCTAGACCGG.CA.GT.T.AA.C..TTG..A.A.A.CTTTCACG.TTT
Kol_19_Asa      GGGT.GT.A---.G.CAATCT------------ACAC.T.TC------------------.T.CAAACTCATTCCTCTTTGAA.T.CTCA------------------------AGGAT.T.AT.A---GGCTTG------.T.G.G---

Tr2_Chr02|3     ACACAGAGTTAA--------------------------------------------------------------------------------------------------------------------------
Tr35_Chr05|3    ......................................................................................................................................
Kol_19_Nig      .A.ACACAGAGTTAA.......................................................................................................................
Kol_19_Asa      ---------G.GACATAAATATACAATGGCAGCCAGTTCGTGCTGATCTGCCTTCTAATATTGCTTCGAAACCTAGACCGGACAAGTATGAAGCTGTTGAAAAACATCTTTCACGTTTTAAAACACAGAGTTAA
```

```
Tr16_Chr04|0        ATGCCAAAATGCATAGTGAAAGGATGTACCAATTATGGGCAAAGAAAAACTATGTTTCACAATGTTATTTTACATGTATTTCCAAAAAATCCAAACATCATAAAAACATGGCTTCAACAGTTCCCAGAGGGTTTTGGTGACATTGACACT
Kol_20_Nig          ......................................................................................................................................................
Kol_20_Asa          ......................................................................................................................................................
Lv10_chr4S|1        ................A.....................A..........................G..........................TG............................T........---......T.........

Tr16_Chr04|0        GTGTGTCAAAAAATTATTGCGGGGAAAAAGACAGATGCCTACAGAATCTGCTCAGAACATTTTAGCACAGACTCATACTGTGTCCAGGGAAATAGATGGGTTTTAAGGAAAGATGCAATACCTACTATTTTTCCAGGAAAAGATTCTACT
Kol_20_Nig          ......................................................................................................................................................
Kol_20_Asa          ......................................................................................................................................................
Lv10_chr4S|1        ..A..C..............T...........G....................T....................G.....C..T.................G.........................................G...G.C

Tr16_Chr04|0        TCATCAGCTGATACAGCAAAACAAATACCAGGTCCAGTAGCTAGCATTCAAGTATCTGCCCCTTGTCCCAAAGATGTGCTTTTGTCACAGTATCCCTCTACCTCATCTTTCCAAAGTCACCCAGCTTTGCTAGTAAGTGCCCCGGTACCG
Kol_20_Nig          .......................G..............................................................................................................................
Kol_20_Asa          .......................G..............................................................................................................................
Lv10_chr4S|1        ......AG..............CG..C.T.A..A..A..C...........CA.....G.A.G...G...................T....T...T...G.....AA...T..........................C.....A......

Tr16_Chr04|0        AGCTCGAGTAACCTTACTTGTTTGCCAATAATTTCATCATTTATAAATTCACCTGAAATATCCTTTCCTGCCACTACTCCTCAAGCTGTCAAGACTGTTGATGTTGGAGTAAATACAGATTCTTTCATGAATAATGAAAATAATTGTGGC
Kol_20_Nig          ......................................................................................................................................................
Kol_20_Asa          ......................................................................................................................................................
Lv10_chr4S|1        .....A...............................................C................T..........A...............................C.......T...........................T

Tr16_Chr04|0        ACTCATCAAAATTTTGGAATGTTTCCAGCCCTGACTGTAACCAAAGCAAAAAACAGTGAGGTTAAAAATGCTAGTTATTACCCCGACACTTCAAATAAAACACCATTATCAGATCCAGTTCCGAGCATTCATCAGTCTGTTACTCCACAT
Kol_20_Nig          ......................................................................................................................................................
Kol_20_Asa          ...................................................................................................................A..................................
Lv10_chr4S|1        G...........G...........T....T...........TG........C..CA...........................T.G..AG............G....T..GAC......GT..A..C..........T.C.A........

Tr16_Chr04|0        CACACAAGTCATGAGTGGTATTCAGTATCTCAAAGAATCATATCCTCTCCTGCTGATATTAGTTATGACAAACCAGGTTGGGAGGAAGATGAAACAGCTCAACGATCTGTAAAATCAAAAGAAGAGTTGACCATTTTAAATGAAAATTGT
Kol_20_Nig          ......................................................................................................................................................
Kol_20_Asa          ......................................................................................................................................................
Lv10_chr4S|1        ..ACA.------------...A..........GA..GTG.G.........A...................T....A.............A...A.C..TGT.....GT..C.C................C........G...G.G.....

Tr16_Chr04|0        GACTATAAAGTT---ACCTTTGATACTGAGGAACCTGTGGAAAAGCAATCAACGTCTTTAAATCCATTTGAGGAGAGCACTTTGTTAGTTTTTGAATCCTGCCTAGACAAATTATTAATGTCCTCAAGATGCTTAGGGGGTAAGAACTGT
Kol_20_Nig          ......................................................................................................................................................
Kol_20_Asa          ......................................................................................................................................................
Lv10_chr4S|1        ......G..A.GGAT..A..G..........G......A...GCT.......TC....................T.....A............................T.....................T........A.....T..C

Tr16_Chr04|0        TCATCTGGTATAAAGCGAATCAAGAAGTATTATATAGGGTCATTTTTATCTGTAAGAGCTGTATGTCACAATGGTCATCATTTTCATTTATGGAATAGCCAACCCAGCAAAGGTCATCTAGCATATGGCAATATCCTACTCTCTGCTGCT
Kol_20_Nig          ......................................................................................................................................................
Kol_20_Asa          ......................................................................................................................................................
Lv10_chr4S|1        ..C....C...C...A.................TC......G...........G................G.......T........C.G..........................G...............G.......G.........

Tr16_Chr04|0        GCTTTATTCAGCAAATCAAGTTTTGCCAATATATTTTCAGTGAATAAACTTCTCCGTATGAAGCAGATCACAGAATCTGCTCATTATCGCTATCAGCAGAATTTTCTGTTTCCCGTGATTAATCACCGTTGGAAAAAAGAACAACAAAAC
Kol_20_Nig          ......................................................................................................................................................
Kol_20_Asa          ......................................................T...............................................................................................
Lv10_chr4S|1        ..A..T.....TG.G..G......A....A........G..A..C..........A............................A....T..........................A...C..........................CG.

Tr16_Chr04|0        TTACAACTTGAATTTTCCAGAAAATCCCTGTGCCTGACTGGAGACAGTGCATCTAATAGCCCTGGGCCTTTA---CCAAAATATTGTTTATATACTCTAACAGAATCCACTACTAATAAAATTCTGAGTTTTAAGGTGGAACATTCTTCT
Kol_20_Nig          ......................................................................................................................................................
Kol_20_Asa          ......................................................................................................................................................
Lv10_chr4S|1        ..........................................C.....A...G....AAG....---C.TTCA..........................AG...G......................A................GT....

Tr16_Chr04|0        CCAAGTACACCATCAAAAGCCCTTAAGAAAACAGCATTTAAAAAGACATTAGATGAAATCCTAGATAAGGAATTTTCTGTAGAAATGATATGTACAGATAGTCATAAGGCAATTGGTGAGATTATTCGGGAGGAGCATAGTGAAATAACG
Kol_20_Nig          ......................................................................................................................................................
Kol_20_Asa          ......................................................................................................................................................
Lv10_chr4S|1        ................T...............T.................C.....G..TG...G..GCA.G..C........T.................C..C...C.....A.......G...A............G.......G.A

Tr16_Chr04|0        CATCGCTTTGACGCATGCCACTTTGCCAAATCTTTGCGCACAAAACTTGTAAAAGCAAGTCGAAGGAGAAACTGTGCAGAATTGTGTAGTTGGATTGACCCAACTGTGAATCATTTTTGGTGGTCCTTAAAAACATCTAATGGTAACGTA
Kol_20_Nig          ......................................................................................................................................................
Kol_20_Asa          ......................................................................................................................................................
Lv10_chr4S|1        ....AGG....T..T...........A...........T..C.....C................A.....G....A.....C......AC...........................................G.GCGG...A.......

Tr16_Chr04|0        GAGTTGCTGTTAGAGAAATGGGCATCGTTGGTAAACCATGTTATTAATGTTCATGAATGGCCAGGCACAACCATACTGTATCATAATTGTGCTCATAGCCAGAGAGAAATGCCATGTGAAAGAAAATGGCTGCATAGAGAATCCACTGCC
Kol_20_Nig          ......................................................................................................................................................
Kol_20_Asa          ......................................................................................................................................................
Lv10_chr4S|1        ..A...........A...........................GCC.......................G....A.G.......C.G...CA.....AT.T..C......G..CAC...................G...............

Tr16_Chr04|0        TTCAATACTTTCAGAAGCATTGTTTTATCCAAAAGGGTAATCTGCGATCTTTCAAATCTCTCTCATTTTTACCAAGCAAGACAAAAT------AAATGTTATCACAATGATATGCTGAAGTGTCATCCAAGACTATATCATGATTCCTCT
Kol_20_Nig          .....................................................................................................C................................................
Kol_20_Asa          .....................................................................................................C................................................
Lv10_chr4S|1        .......G......GA....................A...GAAA..........CG.T.T.G...A....T....A...........AGCTTT..C..C------.GA..G..TT.C.G.CAC...........................

Tr16_Chr04|0        GAGGCCATGCTAGCAAGAACAGAGCTTTCTGCTATGCACCATAATTTCATAACTGAAAGAGCACAAACACAATTAAAAACTGAGCTCCGGCACAGGCCTGGATTCTTCCGGGAAAATAAAGAAAGGCTAATAAAGTCTGTCGCCAAGACT
Kol_20_Nig          ......................................................................................................................................................
Kol_20_Asa          ......................................................................................................................................................
Lv10_chr4S|1        ..T......G......A.....C....G.........GT...........C.T.....A..A...G...........G........GA......TAT.......T.CAGT.............T.C.......T.......T........

Tr16_Chr04|0        ACATCTAATGATTTCTTTCTAGATATTTTAAAAGATGTGGCCAGATATGTTAATGGTGAACTTGACATCAAGGTTCAGTCCTAG
Kol_20_Nig          ....................................................................................
Kol_20_Asa          .....C..............................................................................
Lv10_chr4S|1        ...........A..T..CT.....C...............A..A..T.A.C.G......G.........C.....A........
```

```
Tr17_Chr09|0      ATGCCAACTTGCATAGTGAAGGGATGTTCCAACAGCTGCTCA---AAAAAGCAGTTTCCTCACGTTGTGCTTCACGCTTTCCCCGGAAATCTCACCCAAATAAGAAATTGGCTGGAGCACATTCCCCAGAATCTTCAAAACTTCAGAGAC
Tr38_Chr09|0      ------------------------------------------------------...---------------------------------------------------------------------------------------------------
Kol_21b_Nig       ..........................................................................................................................................................
Kol_21b_Asa       ..........................................................................................................................................................
Kol_21a_Nig       .....G..C................CTGC.......AAGCGG..G.......C..G.....C..C.......T...T..............AT.....AG..C......C.A........----------..G..T...G.....
Kol_21a_Asa       .....G..C................CTGC.......AAGCGG..G.......C..G.....C..C.......T...T..............AT.....AG..C......C.A........----------..G..T...G.....
Lv2_chr6S|1       .....G..C..........T........GC.......AAGCGG..G.....A.....G.....C..C.....T.T..........G........TC....AG..C.....CC.........A---------..G..T...G.....

Tr17_Chr09|0      ATCGACGAAATGGCGCTAAGAGTATTAAAGGGGAAAAAAAGCGACACGTTCCGCGTGTGTTCGGAACATTTCACCGTGGATAGTTACACCGAACAAGGCAAAAAAAGACGAATCCTCAAACGAGAGGCGGTACCAACGCAGTTTCCCGAA
Tr38_Chr09|0      ----------------------------------------------------------------------------------------------------------------------------------------------------------
Kol_21b_Nig       ..........................................................................................................................................................
Kol_21b_Asa       ........G.................................................................................................................................................
Kol_21a_Nig       ...A.....G.....A..T.A.A.T...G.A..A.............C.....TC..................CC...........GT.C..G....CC..---..GG.T..T...AA........G..G....TC..C......
Kol_21a_Asa       ...A.....G.....A..T.A.A.T...G.A..A.............C.....TC..................CC...........GT.C..G....CC..---..GG.T..T...AA........G..G....TC..C......
Lv2_chr6S|1       ...A.....G.....A..T.A.A.T...G.AA...............A......C.....C..............CC...........GTTCA.G....CC..---...G.TT.G...A.G........G.....A.TC..C..T...

Tr17_Chr09|0      ACCTTCTCGCCTCCTTTGAAGAAACCGAAGACACAGAGAGTCTCTAAAAAACAT---AAAGAT-----------------------------------------------------------------TTGGACTCCACTCTGCTCCAA
Tr38_Chr09|0      --------------------------------------------------------...------------------------------------------------------------------------------------------
Kol_21b_Nig       ..........................................................................................................................................................
Kol_21b_Asa       ...............A...........G..............................................................................................................................
Kol_21a_Nig       .....T..AT..G.G..CGTC..T.T..GTG.G..AC..T.TAAA..G...A.GGGG......GCGGAGTCAGAACCTGACTTGCAGTCCATATCATCACTTCTGTCCATCGCCACTTTCCCATATTCC..AACT..GT.......T..G
Kol_21a_Asa       .....T..AT..G.G..CGTC..T.TC.GTG.G..AC..T.TAAA..G...A.GGGG......GCGGAGTCAGAACCTGACTTGCAGTCCATATCATCACTTCTGTCCATCGCCACTTTCCCATATTCC..AACT..GT.......T..G
Lv2_chr6S|1       .T..C.......GAG...GCA.TTTT...TG.G..A...T.TAAA..G...A.GGGC......GCAGAGACGGAACAGGACTTGCAGTCTATATCTTCAGTACTGCCCATTGCTCATACTCCATGTTCCC.AA.....AT..........G

Tr17_Chr09|0      GTGGCACCTAAT------------------------CACTCACAAGTCGTAATTCAA-------------------------------------------------------------------------------------------
Tr38_Chr09|0      ------------.......................---------------------..........................................................................................
Kol_21b_Nig       ..........................................................................................................................................................
Kol_21b_Asa       ..........................................................................................................................................................
Kol_21a_Nig       ---..C..GC.CAAACCAGAAGATGCTTCCGGAACTT.T...TC..CTT.GC.A.CGTCTCCGCACCCAGAAACTTCTCTTCTGGAGCCGACCGAGACATCTGCGGAAACCTCCTCCCAGTCAAATCCGCCGACGGAGGAAAGATCATCT
Kol_21a_Asa       ---..C..GC.CAAACCAGAAGATGCTTCCGGAACTT.T...TC..CTT.GC.A.CGTCTCCGCACCCAGAAACTTCTCTTCTGGAGCCGACCGAGACATCTGCGGAAACCTCCTCCCAGTCAAATCCGCCGACGGAGGAAAGATCATCT
Lv2_chr6S|1       AC.C....C.CA...CCAGCAGATGTCTTTGTGGCT..T...TC..CTTCGT.AAC.TCACCTCGCTCGGAAACTGCTCCTCCAGATCCAACTTGGATAGCTGCAGGAAATCCCTCGCAATCAAATCCATCGATAGAGAAAAA...TCT

Tr17_Chr09|0      ------CAACTTCCC---TGTGTT---TGTTCAAGAATATCAGTTGCAAAAAAGCCAATATTGGTCGACGCCAGCACTTACACCACCTGTTACTTTGAGAAGAAATCAGCTTCTACTAACACGGGTACTAAATTTGCCAAGAGAACTGTG
Tr38_Chr09|0      .....---------..-------..---------------------------------------------.TGA....TGAT.A..TT.A.AG...A..GAGT.TG..G..C..T...TTC..........GT....AG.G...C
Kol_21b_Nig       ..........................................................................................................................................................
Kol_21b_Asa       ..........................................................................................................................................................
Kol_21a_Nig       GGACTA....AA..TTCC....C.CAT..CAAC.A.---..TA.CC.G....GTG.......T......A..GA..TGA....TGAT.A..TT.A.AG...A..GAGT.TG..G..C..T...TTC..........GT....AG.G...C
Kol_21a_Asa       GGACTA....AA..TTCC....C.CAT..CAAC.A.---..TA.CC.G....GCG.......T......A..GA..TGA....TGAT.A..TT.A.AG...A..GAGT.TG..G..C..T...TTC..........GT....AG.G...C
Lv2_chr6S|1       GGACTA....CA..TTCT....C.CAT..CAA..A..---C.T...C.......GCG.......T..T.....A..GA..TGA.T..GGAT.A..T.A.AG...A..GAGT.TG..G.....T....TT..G........GT....AG.G...C

Tr17_Chr09|0      GCCTCACAGACA---GTATTTAACCAGAAAAATAAGAGGTTGCAGTGCAATTTCATGCCGGGACAGTATCCAGTAACGAGC---CAGACTGTGGCTACTCCCTTGGAAAGGCCACCGCCATGGGCTGCTCCTCAAGTGCACTGGGCAACA
Tr38_Chr09|0      ..G..G....AGATGA.T...---..............G.T.........T..AC....TA....T.....C.A...CC.A..AATC...T..T..T.A....TAC.AA.CTCA------------.....GCA.....G.CC...------T.C
Kol_21b_Nig       ..........................................................................................................................................................
Kol_21b_Asa       ..........................................................................................................................................................
Kol_21a_Nig       ..G..G....AGATGA.T...---..............G.T.........T..AC....TA....T.....C.A...CC.A..AATC...T..T..T.A....TAC.AA.CTCA------------.....GCA.....G.CC...------T.C
Kol_21a_Asa       ..G..G....AGATGA.T...---..............G.T.........T..AC....TA....T.....C.A...CC.A..AATC...T..T..T.A....TAC.AA.CTCA------------.....GCA.....G.CC...------T.C
Lv2_chr6S|1       ...A.G..A.AGATGA.T...---..............G.T.........T..AC..C.CA....T..C..C.....CC.A.AAATGTCAT.AT.AT.A.G..TGC.AC.CTCA------------......CA....CGA.....------T.T

Tr17_Chr09|0      CCAAGCCTCAAAAACAGCTATAACGGGCGGCCCTGGATATGGGTTCAACCTGTCTGAGAATGAGAGAGGAAGCAGAGTCCGATTCCGTTGCTACAAAGGGTGTAGATATGCGCGTG---GGTGCTACAGAACACACGGACCCGACCAAT
Tr38_Chr09|0      ---------.C............T........T.........A...............................................G..T...........................................TA....T....T..
Kol_21b_Nig       ..........................................................................................................................................................
Kol_21b_Asa       .......................................................................................................T..................................................
Kol_21a_Nig       ---------.C............T........T.........A...............................................G..T...........................................TA....T....T..
Kol_21a_Asa       ---------.C............T........T.........A...............................................G..T...........................................TA....T....T..
Lv2_chr6S|1       ......T.G....G..........A.A.....TA.....G.......C...................T.........A.....A.....A.A..CA..AT.....A.A.C.G....A..A.GGC..G..........T..T......T...AT..

Tr17_Chr09|0      GAGCCTTTAGTTCAGAACACATACAATGCCAAA---GTTGCCGATGATGAATGCGAAGTGGAATCTACAGTCGATTCAGAGAATCTGACGGATTTGGGATACAAAGATGAGGTTGATGAGAACAAGTTTCTTGTATTCGAGTCTTGTCTG
Tr38_Chr09|0      ..A.......................................................................................................................................................
Kol_21b_Nig       ..........................................................................................................................................................
Kol_21b_Asa       ....................G..G..T....C...A.....A................................................................................................................
Kol_21a_Nig       ..A.......................................................................................................................................................
Kol_21a_Asa       ..A.......................................................................................................................................................
Lv2_chr6S|1       ..............A.G..T.G....GT....TGTT.....T..........AT.........G...................TG.A..CT.....T...A..............C.............A...........T...........

Tr17_Chr09|0      GATAAACTTCTGTGGGGCTCACGTTGCAGGGGAGGGAGGAACTGTCCGGCAGCCATTAAGAGCATTCGGAAGAAAATAACCGGATCTTTCCTAAGTGTAAATGCTGTTTGTACCAAAGGACATTCATTCCATCTTTGGGACAGTCAACCC
Tr38_Chr09|0      .......................................................................................................................G..................................
Kol_21b_Nig       ..........................................................................................................................................................
Kol_21b_Asa       ..............A...........................................................................................................................................
Kol_21a_Nig       .......................................................................................................................G..................................
Kol_21a_Asa       ..........................................................................................................................................................
Lv2_chr6S|1       .....G.....CAT......T..G............A.........A..G..........AT...A..........T..A...........C.....G..........C..T..C...........T.............................

Tr17_Chr09|0      AAAAAGGGCCACATGCCGATCGGCGATGTGTTAATGTCGGCCGCCATCGTACTAAGCGGCTCCAGTTACAGCAAAGTGAGTTACATGAATAAAATTCTGGGGCTCAGGCAGATCGGGAAGGAAACCCATTATCGCCACCAAAGACTCTTC
Tr38_Chr09|0      ........T.................................................................................................................................................
Kol_21b_Nig       ..........................................................................................................................................................
Kol_21b_Asa       ..........................................................................................................................................................
Kol_21a_Nig       ........T.................................................................................................................................................
Kol_21a_Asa       ........T.................................................................................................................................................
Lv2_chr6S|1       ........T...........T..T.....T..G.....A.....TG.T..CT...........A..........................................A...G....A..ATC...A.........T..............

Tr17_Chr09|0      CTGTTCCCGACCATCAATCACCACTGGGAATCGGAGCAGCACCGAGTGATGAAAGAACTGGGGCAGAAACCAGTGTGCCTCGTCACTGATAGGAATTGCCACAGTGCAGAAAAGCGCTGTGTTTGCACATTTATGGAGGCAACCTCAAAG
Tr38_Chr09|0      ..............................T........................GT.......................G.........................................................................
Kol_21b_Nig       ....................................................G.....................................................................................................
Kol_21b_Asa       ..............T...........................................................................................................................................
Kol_21a_Nig       ..............................T........................GT.......................G.........................................................................
Kol_21a_Asa       ..............T........................................GT.......................G.........................................................................
Lv2_chr6S|1       .....T........T.............A...........G..AA......A.....GT.....A.........GT.A.C.G.T..TG.......A.......T..T...C.AG......A..........A...................C.......

Tr17_Chr09|0      AAAATTGTGAGCTTGAAGGTGAATCAAGCGTCGCCTGAAACATCTTCCCTCGCACTCGAGAGACAAACATTCCAAAGCGCCTTGGATAATGTGCTGCAGTGCAACGTAAATGTTCAGCTGGTCTGTACGGAGCCGCGGCCCTGGGTAAAG
Tr38_Chr09|0      ....................G.....................................................................................................................................
Kol_21b_Nig       ..........................................................................................................................................................
Kol_21b_Asa       ..........................................................................................................................................................
Kol_21a_Nig       ....................G.....................................................................................................................................
Kol_21a_Asa       ............................A.....................................................C.......................................................................
Lv2_chr6S|1       ..G......G..T..T.......G..G..GT.A..A..............T..T.T.A.A......GA.C......T.....T......CG.A.........CC.....T............C........A.......A..A.GT.A........A....

Tr17_Chr09|0      AACCTGATGGAAGAGAATTATGGGCACATCCTACATGAGTTTGATGCCTGGCGCTACGGGAAATCCGTTGGTAACAGAGTGGCTGCCGCCAGCAAAAAGAGAAACTGTTCCGAGCTTTCCCAGTGGGGCGCCCCAGTGAGAAACCATTTA
Tr38_Chr09|0      ..............................................................................................................G........A.................................
Kol_21b_Nig       ..........................................................................................................................................................
Kol_21b_Asa       ...........................................................................................................A..............................................
Kol_21a_Nig       ..............................................................................................................G........A.................................
Kol_21a_Asa       ..............................................................................................................G........A.................................
Lv2_chr6S|1       ......................CCA....................T.........T.....G...A.......A.....................................T........AG.......A........C..........

Tr17_Chr09|0      TGGTGGGCGGTTAGATCATGTGAAGGCAACACGGAACTAATGATGGAGAAATGGAACTCACTAATATATCACATAATTGGGGTACATAAATGGAGGACGGGCGCGCTCTTTCACACTTGTGAACATGCGCCTCAGGAGGATGAAGTGACA
Tr38_Chr09|0      ..................................................G....................G...........................T.......A....GGC...................................
Kol_21b_Nig       ..........................................................................................................................................................
Kol_21b_Asa       ..........................................................C..........G..........................................A......................................
Kol_21a_Nig       ..................................................G....................G...........................T.......A....GGC...................................
Kol_21a_Asa       ..................................................G....................G...........................T.......A....GGC...................................
Lv2_chr6S|1       ....................CA.........C.....T........A..G........G...........TG.G..............C....A...A..T..C..T.A...TGGA..............AG........C..C.CTG..

Tr17_Chr09|0      AGATCATTTCTAGTGAAAGGTTCTAGAGCGCATCGAAAGCTGAGAGAGATTGTACTGAGTCGGGACATCAACAAAAATCTCCCAAAGCTGCGGAATATTTGTCACATGGGGGAGCTGGATGTGTTCCGCAGCAACGCCCTCAAGTACCGG
Tr38_Chr09|0      .....G.......G...............T...A........................................G................................................................................
Kol_21b_Nig       ..........................................................................................................................................................
Kol_21b_Asa       .............G............................................................G...............................................................................
Kol_21a_Nig       .....G.......G...............T...A........................................G................................................................................
Kol_21a_Asa       .....G.......G...............T...A........................................G.........................................................A................
Lv2_chr6S|1       ....TG..C...AG.....A....A.....TT..A....A...............A.A..........G.....T.....T..GT..............CT..........CT..TA....T.......T.C

Tr17_Chr09|0      CCAAAGCAAAGCCACTTCTCCGCCCATTCCTTAATTGCACGGACTCAGCTGGCCGCACTAGACCACAATAGGAATGTCCATCACCAGCTGGCCATCTTGGCTAATGGAAGAGATGAGGAAGTGCTAAGGGAGGACTATCCCAGGGTGGAA
Tr38_Chr09|0      ....................TA.........................................................................................................T........................
Kol_21b_Nig       ..........................................................................................................................................................
Kol_21b_Asa       ....................TA....................................................................................................................................
Kol_21a_Nig       ....................TA.........................................................................................................T........................
Kol_21a_Asa       ....................TA....................................................................................................................................
Lv2_chr6S|1       ..........A.........AGGGG.....C.........C.....A..A...T..........................T.......................A...A..C..A....C..................................

Tr17_Chr09|0      TTCCGTGAGTTGAAGAAGGACTGGCGAGTGAAACCTGTGTATGACTTGGCACGGTGTGATTTTATATTCACTATCATGAAAGACGTGGTCAGATACGTGTATGGGGAAAGGTGTCCTGGGTAG
Tr38_Chr09|0      ........................A.............C..........G......C.................................................A.............
Kol_21b_Nig       ..........................................................................................................................
Kol_21b_Asa       ....A............T........................................................................................................
Kol_21a_Nig       ........................A.............C..........G......C..........................................T............A.............
Kol_21a_Asa       ........................A.............C..........G......C.................................................................
Lv2_chr6S|1       ..TA.CC....A............A..........A..T......A.A.A...T....................AC...............AG.................A....TT.......
```

```
Tr4_Chr09|0      ATGCCAAAGTGTATTGTAAATGGATGTTCCCATGGCAGCAGAGGTAAAACCCCATCTCCAAATGTGGTACTGCATGTGTTTCCTGGAAATATTGATATTATAAAAAGATGGCTTGTAGCTATTGGGCAAAACTTTGGTGACTTGGACACC
Kol_23_Nig       ......................................................................................................................................................
Kol_23_Asa       ......................................................................................................................................................
Lv9_chr9_10L|0   ...................................T..............A..G...............G....C..T....................A..............CA.G..A.....A.....T..................

Tr4_Chr09|0      TATGCTCAAAGAATTATAGAGGGCAAGAAAACTGATTCCTTCCGAATTTGTTCATGTCACTTTACTGAAGATTCTTACACTTTTCAAGGAAGAAGAAAGGCTTTAAAGAAGTCAGCAACTCCCACATTGTTTCTTTGGAGTGGATCTGCT
Kol_23_Nig       ......................................................................................................................................................
Kol_23_Asa       ......................................................................................................................................................
Lv9_chr9_10L|0   ................................G...............................................C.....G..G...C.G......C.G..A...............................A..........

Tr4_Chr09|0      TCAGATGGATGTAAAGCAGTCCCTGCAAAGAGAGCCCGTAATGATAAAGGTGACCAATGTGAATCTCTGCCTTTTCAGTCTCACCAGAACTCAGCATCTAATTCTTTCTCACAGCAAACAATTACTGGC------CAGACAAAGAAGAAA
Kol_23_Nig       ......................................................................................................................................................
Kol_23_Asa       ........................................C...................................................C.........................................................
Lv9_chr9_10L|0   ..GA...A.........G...............C.....CC.A......A......T.C..........T..GC........G...A..T..CA.G..........C....A..G.....TC.G.....CAATCT...........A...

Tr4_Chr09|0      ACTATAAAAATCAGGCGTAGTAATGATTCTACCTGTACAAAACAAAATTCCTGTAAGAGGAATGCTTCAACTCAAACAATATTTGAAAATGGGGATGCAGTTACATCCTACTATTGCTCGCAGTGTGCTTCACCACTCAATAAAACTAGT
Kol_23_Nig       ......................................................................................................................................................
Kol_23_Asa       ................................................................................................................................C.....................
Lv9_chr9_10L|0   ....A....C.TG..TAC.........C.....A.AG.............T..............C...............A........---...A...ACC................A..T.....A.........------..C...

Tr4_Chr09|0      GTTACATCAGTTCAGACATTGAATTTGCTGCCCTCAGAACCGGCATACAGCAAAATTCATTTAGTTGATTCTGAAGGGCAATCTGAATGTAAATCTAAACCTACTATTTTTCATGATATTAAAATGGAAATTGAAGACGCTGAGAACACT
Kol_23_Nig       ......................................................................................................................................................
Kol_23_Asa       .....................G................................................................................................................................
Lv9_chr9_10L|0   AAA..CAGT..........C.G.............C.........CC..A.....CGA....T...........C.TA..G......CA.C.G......G...G..A......G...G...........G.......GC....A.....C

Tr4_Chr09|0      TTATCAGATGACGGCACAGATACATCAAGTAGCATTGGTGATGATTCCGACATA------------TCTAAACATAGTGAAGATAGCCCCATATTCATAGAATATGAAAGTGAGGAAGATGATGAAATAGATCAGTCAATAGAACCTTTT
Kol_23_Nig       ......................................................................................................................................................
Kol_23_Asa       ......................................................TCTTCTCTGCCT....................................................................................
Lv9_chr9_10L|0   ............AA..........A........T..A.............TGC.TCTTCTCTACCT.....T.T.....T....TA.T.......TG.....C....T.C....................C......C........A...

Tr4_Chr09|0      GATGTTTTAAATACTGCTAAGGATCCGGTGGATGATCACACTTTCCTAGTTTATGAATCTTGCCTTGATAAATTGCTATTGTCTTCAAGATGTGGTAGAGATCCAAACTGCTATTCACCAATAAAAAAATTGAAAAAGTATGTATATGGA
Kol_23_Nig       ......................................................................................................................................................
Kol_23_Asa       ......................................................................................................................................................
Lv9_chr9_10L|0   .............................C.....C..T.....T.......T...............C........GC................................C..........T.....G........A.....T.T...C

Tr4_Chr09|0      TCATTCTTAACTGTCAAAGCGGTATGTCAGAGTGGACACCATTTTCATCTTTGGGACAGTCAGCCTCGTAAAGGTTCCATATATTATGGAAACCTGCTGATGTCTGCATCAATCCTTCTTAGTGGTTCTGACTTTGCAAAAGTTTATGCC
Kol_23_Nig       ......................................................................................................................................................
Kol_23_Asa       .............................................................................................T........................................................
Lv9_chr9_10L|0   ..C.................A.................TA..........G..........................................T..T..G....G...................................G.........

Tr4_Chr09|0      ATGAATAAGCTCTTGAAGTTAAAACAAATGTCGCCATCTGCTTTCAAGCGATATCAATCAAATTATTTCATTCCAGTTATAGACCACCACTGGAAGACTGAGCAGGACAAACTAATAAAGCATATTTCAGGGAAATCTGTATTTCTTGTA
Kol_23_Nig       ......................................................................................................................................................
Kol_23_Asa       ......................................................................................................................................................
Lv9_chr9_10L|0   .....................................................C.G............AT....C......A...............T......A..A...G.G........G...G........C.............G

Tr4_Chr09|0      GGAGATCATGATATAAACATCCCTGGTAGCTTTTCTAAGTACTGCACATATTCACTCATGGAAGGCGCCTCCATGAAGATTATCAATTATAGGGTGGACCAAGTTTCTTCTACCACTTCCTCTGCTGATGCTGAAAAACAGTCTTTTCAG
Kol_23_Nig       ......................................................................................................................................................
Kol_23_Asa       .....................................................................................G................................................................
Lv9_chr9_10L|0   .A....A..C..GCC...........AT.....CA.C.......T.....C........A.....A.......A..............T.........A......------------..T........CA............G....A..

Tr4_Chr09|0      AAATCACTGGATGAACTCTTGGAAAAAGATGTTAAGGTGAAAAGCATATGTACAAATCGTCGCAAGGCAATAAGAAAACTTATTTATAAAGACTATTCCGACATACTACATAAATATAATATTGACCATATTAGCAGGTCTTTAAGGAAC
Kol_23_Nig       ......................................................................................................................................................
Kol_23_Asa       ..............................................................A.......................................................................................
Lv9_chr9_10L|0   ......T.....A.G...........G..AA..C..............G..........G---..AT..............G..C.G..G..T...C...G.....A....C.T......G.CAG...........A..G...G..A...

Tr4_Chr09|0      AAACTGTCATCTGCTAGTAAACAGAAAAACTGTAGTCAGATATCACAGTGGATTAAACCCGCAGTCAGTCATCTATTGTGGGCCTCTCAGACATGTGATGGTAGTGCTGATTTGTTGAAAAAGAAGTGGCAATCATTACTGAATCATGTT
Kol_23_Nig       ......................................................................................................................................................
Kol_23_Asa       ......................................C.................................................G..........................................G..................
Lv9_chr9_10L|0   .........G............GA........C........C.............C......T....A......G........T...A................C.............................T...A.......G...

Tr4_Chr09|0      ACAAAGGTGCACAAATGGGAGACTTCTCAGCAATTCCATGGATGCACACAAGAAAATCTGAGTGCGAGGTGCAAAAGAAAGTGGATGAAATGTGGTTCAACAGCATTCAACAGATTCAAAGAAATTGTTATGTCTGCTCAACTCATCAGA
Kol_23_Nig       ......................................................................................................................................................
Kol_23_Asa       ......................................................................................................................................................
Lv9_chr9_10L|0   G...C...............A...G.......TG...........G.C..T..G...........T.........G................................T..........G...C................G.........

Tr4_Chr09|0      GACCTCAATCATCTTTCAGAGTTCTGCCATACAGATGAACTAAAGCTATACCACAGTAATCTCCTTAAATATCGGCCAAGAACACTTCACTCCATGGATGACGTGGTTGTCCGCACTCAACTTGCAGCTCTTGATCATAATTACAATGTT
Kol_23_Nig       ......................................................................................................................................................
Kol_23_Asa       ......................................................................................................................................................
Lv9_chr9_10L|0   .....T....C.......A..A.......C............G...T....T....AC.........C...CT...................G.G...G...A...........T.....................T.........G...

Tr4_Chr09|0      CACAGAGGAAAAGCACAATGGAATGATACCGATGTGCAGGACCCTTCTGCTGGTTTACAGCCTAAAGTAAGAAAATGTGTCTTTGATGCAGCTTCAAAAGCTTTCCTTTTGGTCATCCTGAAGGATATTGTGAGGTATGTGGAAGGAGAA
Kol_23_Nig       ......................................................................................................................................................
Kol_23_Asa       ..............................C.......................................................................................................................
Lv9_chr9_10L|0   ........G...T...CGGA....TT....C.......A..........G..C................G..........T....................................................CA.T..CT.........

Tr4_Chr09|0      GTCAATATCGAAAGGCAAACTTTAAGTGACCGACTTCCTGACAACCCTTCACTGGAACACAGGCCAATGAAGAATGAATCATGGTGA
Kol_23_Nig       .......................................................................................
Kol_23_Asa       .......................................................................................
Lv9_chr9_10L|0   ........T..G..................A..T..T..............T.......G........A...G....GCA.......
```

```
Tr3_Chr01|0          ATGCTTTTGCAACTTGGTCCGCAGCAACCACCCTTACAGCTTGGGACTCAGCAACTACCTCTGCAACTAGGAGCACAATACCAGCCTTTGCAGTTAGGGGTTCAACAGCCAGCTTTGCATGTGGGAACCCCCCAACCAACTTTCCAAGTA
Kol_24_Nig           ......................................................................................................................................................
Kol_24_Asa           ......................................................................................................................................................

Tr3_Chr01|0          GGATGTCAGCTTCCATTAGGAGCTCAACATCAATCTTTTCAACTTACGGCTCAGCAATCTGCTCTCCAGTTATTGGCTCAACAGTCAGCTTTACAATTAATGGCTCAGCAATCAGCTTTACAATTACTGACCCAGCAATCAGTTTTACAA
Kol_24_Nig           ......................................................................................................................................................
Kol_24_Asa           ......................................................................................................................................................

Tr3_Chr01|0          CTAATGGCCCAGCAACCAGCTTTACAAGTCACAGCACAGCAACCAACTGTACAAGTTACAGCCCAGCAGCCAATTTTAGAGCAACAAGGTATTCCCCAATTAAATATGAATACTGGGGCCCCTGGGCCAACATCAATTATACCCATGAGG
Kol_24_Nig           ......................................................................................................................................................
Kol_24_Asa           ......................................................................................................................................................

Tr3_Chr01|0          ATATTAAGCACTGACCATGGTCTTATTCTGGACATAGACCCAACAGGAACCTTTATTAAGTCAAAAGGAGAAGTAAAAAATGTATTAACAAAGAAAGAAATGGTTGATGCCTCAACATCTACATTTGAAGCTGTTAAAATGGAAGACAAA
Kol_24_Nig           ......................................................................................................................................................
Kol_24_Asa           ......................................................................................................................................................

Tr3_Chr01|0          AGTGTACAATGGCCTGAGTTTGAATTCAATTTTGATGGCGAGCTGTGGAAAATAGAGCAGGATCACTTTTACCCTTCATGTTTTACCACTGTCAAAAATGACAGACCTTCCACCAGACACCAAAAATACCCTTTTGATGTTGGCAGTCCT
Kol_24_Nig           ......................................................................................................................................................
Kol_24_Asa           ......................................................................................................................................................

Tr3_Chr01|0          ATTCTTACAGAACAAGACATACTCTTGGATTCTGAAATTACAAGCAGGAAGACATTTTCTTTCATTGGCTCAACATTAAAAGAAATAAAATCTGAAACACATGACTCAGATAGTGAACAAAGACACACTCTGAGAACAAGCAAAAAACCC
Kol_24_Nig           ......................................................................................................................................................
Kol_24_Asa           ......................................................................................................................................................

Tr3_Chr01|0          TTTTGTGAAAGAGAAATAGAGACAGAGGTTGCAAATGAACGAAAATTTATTGTTTTTGAGTCTTGTCTTGACTTTCTTTTCTATAAAATGTGTTGTCGTTTTGGAAATGGCTGTAAAGCCCATATAACTAAAATAGAAAAATTTGTTGAT
Kol_24_Nig           ......................................................................................................................................................
Kol_24_Asa           G.....................................................................................................................................................

Tr3_Chr01|0          GGTTCCTTTTTATCAGTCACTGCCCGCTGTCAAAATGGTCATCGCTTCCACCTCTGGCACAGTCAGCCACTGATGGGATGTATGGCTGTAGGAAATATTCTCACAGCAGCTGCTGTGCTTTTTAGTGGCTCCAACTTTCATAAAGTATAT
Kol_24_Nig           ......................................................................................................................................................
Kol_24_Asa           ......................................................................................................................................................

Tr3_Chr01|0          GAAATGAACAGTGTATTGGGCTTGCAGCAAATAAGCAAAACTGTTTATGACAGTTATCAAAATACATTTCTCTTTCCCACAATTGACCTTCATTGGCAACAGGAGTGTCTCCGCCTCAATAGGGCTTTTGCCAACACTCCACTGACTTTG
Kol_24_Nig           ......................................................................................................................................................
Kol_24_Asa           ......................................................................................................................................................

Tr3_Chr01|0          ACTGGAGATGGCCAGTTTGAACAAGTAGGACACAATATTAAAAACTTTAGTTACACATTCATGGAATCTGCAACAAAAAGAATTGTAGATTTTCAAATTGAACCAGTATCTGATTTAACTTCTACATCTACAGTAGAAAATCATGTATTC
Kol_24_Nig           ......................................................................................................................................................
Kol_24_Asa           ......................................................................................................................................................

Tr3_Chr01|0          AGCATTTGTTTAAACAGATTACTGAATAATGATTTTAAAGTAAAATCTGTTGCCACTGGACACAGTTCTGCCATAAAAAAAATTATGCATAAAAAATACAGACATTTGAAACATGAATATGATGTATGGCATTATGCCAAAGGTGTGAAA
Kol_24_Nig           ......................................................................................................................................................
Kol_24_Asa           ......................................................................................................................................................

Tr3_Chr01|0          AAACACTTAACCAGGGCTAGGAAAGGGAAAACATTGGTCAAGCTCATCAATTGGCTGCCAGCTATTTCTACTCATCTATGGTGGTCATGCATCACCAGTCAAAGAAACACTGTTATGTTTCAGGAAAGATGGCAATCCTTGCTTCATCAT
Kol_24_Nig           .........................................................................................T............................................................
Kol_24_Asa           .....................................................................T................................................................................

Tr3_Chr01|0          GTAACAAATCAGCATCAATGGAAGAATGCAGAAACATTTCATGGTTGTTCTCATGGGAAGTTAACTTCCATGCAACACAGACAGTGTTCATGGTTGAAAAAAGGCACTCCAGCATTCCATACTCTAAGAGACGTTGTAATGAACCCTCAA
Kol_24_Nig           ......................................................................................................................................................
Kol_24_Asa           ......................................................................................................................................................

Tr3_Chr01|0          ATAACAAAAGATTTTCACTGCATGTCTCGGTTTTGCTGCACAGAGGAAATGGATGTTTACAAGAGCTTTGTTACCAAATATCAAACAAATAGGTTTCATTTAAATATTAATGCAATGGAAGCAAGAACAAAGCTGGCTGCTCTTGCTTAC
Kol_24_Nig           ......................................................................................................................................................
Kol_24_Asa           ......................................................................................................................................................

Tr3_Chr01|0          AATGCAAATGTACACAGGTACCCAATACACACAAAATACATGGGAAGAGGGAGTATTGGTAGGGCAAGACAGAAACACATGTTCCCAAAATGCAGAAAGCGACGTGTAGCCAGATCTGTTTATACAGACACATTTACAGAACATACTCTG
Kol_24_Nig           ......................................................................................................................................................
Kol_24_Asa           ......................................................................................................................................................

Tr3_Chr01|0          TCTATGATGGCTGATGTGCTAAAGATCTACACTGGCAGACTAAACCATAGCTGGATTCCCAGGTGTGTCAATGAACCCACAAACATCCCTTCAGAACCTGTACCTGTGCAATTATGGACCATTGCGCCAAGCGTCTCCCATTTTCCAGTT
Kol_24_Nig           ......................................................................................................................................................
Kol_24_Asa           .........................................................................T............................................................................

Tr3_Chr01|0          TTGCTATAA
Kol_24_Nig           .........
Kol_24_Asa           .......G.
```

```
Tr30_Chr09|0      ATGCCATCTTGCCTAGTCCATGGTTGTCGTCACAGCACAGCAAGAAAAGGCCTCTATCCTGGCATCGTCTTGCACGGATTCCCAAAAAACCTTTCCAGAATAAAGCAATGGCTCTTAAACACAGGACAGAATTTTGGCAACCTTGATGCC
Tr31_Chr09|0      ............A......G........A..........T.............AG.......T..T...........................AA...................A...TG...CA........A............G...
Kol_25_Nig        ......................................................................................................................................................
Kol_25_Asa        ......................................................................................................................................................
Kol_26_Nig        ............A......G........A..........T.............AG.......T..T...........................AA...................A...TG...CA........A............G...
Kol_26_Asa        ............A......G........A..........T.............AG.......T..T...........................AA...................A...TG...CA.....................G...
Lv19_chr9_10L|0   ............A......G........A...................T..T.C........A....C..................C.................A........G.G....TC......A...G.....G..A......AT

Tr30_Chr09|0      TTTGCACAGAAAGTCCTGGATGGAAAGAAACACAATTCCTATCGTATTTGCTCTTCCCATTTTTCCTCCGATTGTTACGTCCAACTCGGCTGTACAAAGGGACTGAAAGCAGATGCAGTGCCCACAATATTTGCTTGGAACACCCCA---
Tr31_Chr09|0      ................A.A.............................T...G...........C.T.........A..T.C......C.C.....A.................T..............................GAG
Kol_25_Nig        ......................................................................................................................................................
Kol_25_Asa        ......................................................................................................................................................
Kol_26_Nig        ................A.A.............................T...G...........C.T.........A..T.C......C.C.....A.................T..............................GAG
Kol_26_Asa        ................A.A.................A...........T...G...........C.T.........A..T.C......C.C.....A................................................GAG
Lv19_chr9_10L|0   ................................G...........A.........G.............T.......T.......T......A.T...............................G.....................GAG

Tr30_Chr09|0      ------GGCGAGAGCGAGGACAGTGCTTATCACATTCACCGCTCA---ACATACCCCGAGCGACCATGTGGCCGCTGTATGAAAAAGAAAATGGTCGACGCAAGCACGCTCACAGATCCAGCAATGTTTTCCAAAGAAGCAGGTGTGCAG
Tr31_Chr09|0      .........AGAGAGA.CAGT..------------A.T..----..GTT..T..TC....G.T....---.AG.......G...........T..T..C.....A........---------------------------C..G.A
Kol_25_Nig        ......................................................................................................................................................
Kol_25_Asa        ......................................................................................................................................................
Kol_26_Nig        .........AGAGAGA.CAGT..------------A.T..----..GTT..T..TC....G.T....---.AG.......G...........T..T..C.....A........---------------------------C..G.A
Kol_26_Asa        .........AGAGAGA.CAGT..------------A.T..----..GTTC.T..TC....C.T....---.AG.......G...........T..T..C.....A........---------------------------C..G.A
Lv19_chr9_10L|0   AGCAGA..GCGC....CTATTC.C------------A.T....ACTTT.TG.....G..A.....T.....T..G..........TT........T..T........ATC...G.................A.........T.....A...

Tr30_Chr09|0      TGGCCGGAGTTTGAGTTTAATACAGCTGGTGAGAGATGGAAAATAAAGCACGATCACTATTACTATTTAAGCTCTTCAAGAAAAAGCACCCAAAAACCTACAGTGGGTCGTGATCGTCAAAAAGAC---------ACAAGTAATAAGATC
Tr31_Chr09|0      .C...A..C---------TC.G...T......A.A.......G............TC.....C.....G..........G...........G...........A..A.....T.G.......TTCAGTAGAC..G.......TA.AG
Kol_25_Nig        ......................................................................................................................................................
Kol_25_Asa        ......................................................................................................................................................
Kol_26_Nig        .C...A..C---------TC.G...T......A.A.......G............TC.....C.....G..........G...........G...........A..A.....T.G.......TTCAGTAGAC..G.......TA.AG
Kol_26_Asa        .....A..C---------TC.G...T......A.A.......G............TC.....C.....G..........G...........G...........A..A.....T.G.......TTCAGTAGAC..G.......TA..G
Lv19_chr9_10L|0   .....A..A..C.........................G...................C...C...C............G...........G...........C.....C...T.........TCTGATGGGC..G.......T.AA

Tr30_Chr09|0      AACAACAAAGATGATGAGGAGGATGATGATGAAGACGATGATAATGATAGCAATGATAGCTTCCAAACCCACCAGTCCTCAGTACACGTAGATCCATTTTATTCTGGTATACCAGAGATAACCAGAGCAGACGGTTTCATGGATTTCACG
Tr31_Chr09|0      ......CC....---------........C---.....A..CG.AATC---------TCT..------------------A...GT........................T..........TG.......TA.A..T.........G.A
Kol_25_Nig        ......................................................................................................................................................
Kol_25_Asa        ...................................................................................................................................G..................
Kol_26_Nig        ......CC....---------........C---.....A..CG.AATC---------TCT..------------------A...GT........................T..........TG.......TA.A..T.........G.A
Kol_26_Asa        ......CC.......------........C---.....A..CG.AATC---------TCT..------------------A...GT...................................TG.......TA.G..T........TG.A
Lv19_chr9_10L|0   ....G.CG......G...C....G.TG...---..TA......GCAG..A.........A.AC...G..T......C....C...A.TT...........C.......G........A.......A.......A....T.......C...T

Tr30_Chr09|0      CTATCCTCCTACAAAGTAAGACACAGCATACAAAATACGTTC---------GGAAGTACAGAGCACACCTTACCCATGGACAATGACGTTGCAGCGTTAGATGTTGTCCATCAAAGGAAGTTTATAGTGTTTGAGTCTTGCCTTGATGTC
Tr31_Chr09|0      .C....C..C...........G.TG....G.....C.T.............A................G..T...G.............G....TT..................................A..............C......
Kol_25_Nig        ......................................................................................................................................................
Kol_25_Asa        ......................................................................................................................................................
Kol_26_Nig        .C....C..C...........G.TG....G.....C.T.............A................G..T...G.............G....TT..................................A..............C......
Kol_26_Asa        .C....C..C...........G.TG....G.....C.T.............A................G..T..................G....TT.....................................................
Lv19_chr9_10L|0   .C.......C...................C.....T.....C.A..C.AGCAGTAATA.........................G.......TAC.....TTC.....A...CG......C..........C..A...................

Tr30_Chr09|0      TTACTTCGTAAGCTTTCTTGTGGATTTGGTGTGAACTGCAGTGCTCCTATTGTTGGACTTGAGAAGCACGTGGAGGGTTCTTACTTGTCTGTAGTTGGACGGTGCTCCAAAGGCCACCGCTTTCATCTATGGGACAGCCAACCTGTAGTA
Tr31_Chr09|0      ............................G..................C........G.........T....................................T....................G........................
Kol_25_Nig        ......................................................................................................................................................
Kol_25_Asa        ......................................................................................................................................................
Kol_26_Nig        ............................G..................C........G.........T....................................T....................G........................
Kol_26_Asa        .......A....................G..................C........G.........T.........................................................G........................
Lv19_chr9_10L|0   ..G....AC........C.....T.........G.....C..T.C..C..CA................A...A....C....................CA............................G..........G.......C.

Tr30_Chr09|0      GGAGACGTTGCACTGGGTAACCTGTTGACATCGGCTGCTCTGCTTTTCAGTGGTTCCAGATTTTATAAGGTTGAAGAAATGAGTCGGCTATTGGGACTGCAGCTAATTTCCCGCGACGTCTACCATAAATACCAGCGTACATTGCTGTTT
Tr31_Chr09|0      ......A.............T.........................................G...............................................A..AC....................................
Kol_25_Nig        ......................................................................................................................................................
Kol_25_Asa        ....................T.................................................................................................................................
Kol_26_Nig        ......A.............T.........................................G...............................................A..AC....................................
Kol_26_Asa        ....................T.........................................G...............................................A..AC....................................
Lv19_chr9_10L|0   .....T...........C..T..T...G....A..................C.....T.......CA..............T...A...GA....C.................T.....ACT....G............G....T..A...

Tr30_Chr09|0      CCTACAATTGATCATCATTGGCTGTATGAGCGCAAACTGCTCAAAGAGGCTGTAGGATCTAAAAAACTGTGCCTTTCTGGGGATGGGCATAACTCTACGGTCTCTATGTACACGTTCGTCGAACCCGAGAGCAAACTTATTTTAGATTTT
Tr31_Chr09|0      ......G.C.C.A.GT........C......A.....A............C..............T.......C....T.........G.GT......T.T....C..........ACT..............G.AA............
Kol_25_Nig        ......................................................................................................................................................
Kol_25_Asa        ......................................................................................................................................................
Kol_26_Nig        ......G.C.C.A.GT........C......A.....A............A..............T.......C....T.........G.GT......T.T....C..........ACT..............G.AA............
Kol_26_Asa        ......G.C.C.A.GT........C......A.....A............C..............T.......C....T.........G.GT......T.T....C..........ACT..............G.AA............
Lv19_chr9_10L|0   ........C....GC........TA.......T.G..G.........TA.C.......A..C.......................T..C.....A.A.......C..........ACT......C....T.....A...C........

Tr30_Chr09|0      CAAGTTGTTCAGAAGGCAGAGAGACGTAAAAGGCCAGACTTGGCGATGGAAAAGCTAGCCTTTGAAATCTGCTTAGATCGACTTCTCAAAGAGCATTATGTTGTTGAGGCCATTGCAACAGACCAACATCCAGCTATTGAAGAACTAATG
Tr31_Chr09|0      .....A............A...C.A...CTTCC.A...AA.T------...................C......G...................G..T..........A.....T.................T.GC.....G........
Kol_25_Nig        ......................................................................................................................................................
Kol_25_Asa        ......................................................................................................................................................
Kol_26_Nig        .....A............A...C.A...CTTCC.A...AA.T------...................C......G...................G..T..........A.....T.................T.GC.....G........
Kol_26_Asa        .....A............A...C.A...CTTCC.A...AA.T------...................C......G...................G..T..........A.....T.................T.GC.....G........
Lv19_chr9_10L|0   ..G..G.........T.TA.A.CCA..C...AA.AG...C.A...A.A....C.........A.......A..T..............A.....TA....C.......................G...T.T..............C...

Tr30_Chr09|0      TGTGAAAAGTACAGTTCTGTCATTCACAAGTATGATATTTGGGTTTGTGCCCAAAAGGTTAAGAGACAGTTGGTGGCCGCCAGCAAAAAGAGAAAATGTTCTCAGATAAGAAAGTGGATCCCTTCAATCATGCTACACTTTGAATGGTCT
Tr31_Chr09|0      ...........TG.CAG...TTG.............G.....C...A.......G....A..A..G..A........T..........GA...........A.....C..C............G.......C.AG.....G......GT.
Kol_25_Nig        ......................................................................................................................................................
Kol_25_Asa        ......................................................................................................................................................
Kol_26_Nig        ...........TG.CAG...TTG.............G.....C...A.......G....A..A..G..A........T..........GA...........A.....C..C............G.......C.AG.....G......GT.
Kol_26_Asa        ...........TG.CAG...TTG.............T.....C...A.......G....A..A..G..A........T..........GA...........A.....C.CC............G.......C.AG.....G......G..
Lv19_chr9_10L|0   ...C.G..........C..A.................G..G.CT...A..............A..A.....A.....T.T...........A..G..G..C..A.....T..C.......A...G.G.....T...................

Tr30_Chr09|0      TCCAGAACTAGTCACGGCGATGTAGCCTTGTTTCGTGAGAAGTGGCAGTCACTGCTAATGCACATTACTAATCACCATAAGTGGGATGGTTCCAAAGTGTGCCATGTATGTTGCCATAAAGCTCTGGTTCGCCATTCACGGCCCCTTCCC
Tr31_Chr09|0      ..G..........T..A.....G........................A.........................G........C...CA......T...........C....C............T..AAC...TG..T...AT.A......
Kol_25_Nig        ......................................................................................................................................................
Kol_25_Asa        ..............................C...............................................................................................G.......................
Kol_26_Nig        ..G..........T..A.....G........................A.........................G........C...CA......T...........C....C............T..AAC...TG..T...AT.A......
Kol_26_Asa        ..G..........T..A.....G........................A.........................G........C...CA......T...........C....C............T..AAC...TG..T...AT.A......
Lv19_chr9_10L|0   ....A.........A..GA.C..TT.............A.......A..G.........................G.......GC..C...GT...A.......C...CGA...............CC....TA....G......A......

Tr30_Chr09|0      TGGATCAAAATGTGGTGTCCGGCCTTCCATGCTTTACGTGAAGTCATTCTTTCCTCCAGGTTGGCGAAGGACTTGGCTCTCCTTTCTCAGTTTAGCCACGGTGAAGCTGTTTTGTTATACCATCGTCTTCTTCAGAAGTATATGCCTAGT
Tr31_Chr09|0      ..........AA...AC...A........C...............GG............ATC..A.........A....C.GTT...............TT..CA...ACA............G...C..C....A.A..A....G...A...
Kol_25_Nig        ......................................................................................................................................................
Kol_25_Asa        ......................................................................................................................................................
Kol_26_Nig        ..........AA...AC...A........C...............GG............ATC..A.........A....C.GTT...............TT..CA...ACA............G...C..C....A.A..A....G...A...
Kol_26_Asa        ..........AA...AC...A........C...............GG............ATC..A.........A....C.GTT...............TT..CA...ACA............G...C.......A.A..A....G...A...
Lv19_chr9_10L|0   .....T..GT...................................G..........T..A.C..A............A.C.GT.....................CAG..AGA.....................C.......A.....G....G..

Tr30_Chr09|0      TCAGTTCATCTTAGGATGGATGCTGTGGATGCAAGGACCAAACTAGCAGCTCTTACGTATAATGCAAATGTGCACATGAGACGTACAAAGGCTGAGGTCTCCGTAGAAGTATCTGGTGGTGCT--------------------GATTAC
Tr31_Chr09|0      GAT....G.T.C.........T...C.........................A........C......T..A......CAG.G.AA.G..G.C..A....T...CC.AG..C.A........T..GACCTACAGATAGACAAAGTG...---
Kol_25_Nig        ......................................................................................................................................................
Kol_25_Asa        .......................................................................G..............................................................................
Kol_26_Nig        GAT....G.T.C.........T...C.........................A........C......T..A......CAG.G.AA.G..G.C..A....T...CC.AG..C.A........T..GACCTACAGATAGACAAAGTG...---
Kol_26_Asa        GAT....G.T.C.........T...C.........................A........C......T..A......CAG.G.AA.G..G.C..A....T...CC.AG..C.A........T..GACCTACAGATAGACAAAGTG...---
Lv19_chr9_10L|0   GT...C.G.............T......................G......G.A..C..........A......G.G.A......AT..C........C.....AC...............GCCCAGGAGAGACTCAAGATG......

Tr30_Chr09|0      AGTTGGCCGACCTTAAAATCACTGTCCAAACCAGACTCGAATGCTCATTTGTTGCGCATGATGGTGGATGCTATAAAACTGTGCTCCATTAACCCAAACCCATGT---TAG
Tr31_Chr09|0      .AC...T.TG....GTC....ACA..T......A.........G..................................T.........C....T....G.A.A.AGTC...
Kol_25_Nig        ...............................................................................................................
Kol_25_Asa        ...............................................................................................................
Kol_26_Nig        .AC...T.TG....GTC....ACA..T......A.........G..................................T.........C....T....G.A.A.AGTC...
Kol_26_Asa        .AC...T.TG....GTC....ACA..T......A.........G..................................T.........C....T....G.A.A.AGTC...
Lv19_chr9_10L|0   .......G......G...............................................................T.......TG..........G..TTGCAGTC...
```

```
Tr33_Chr09|1        ATGCCGAATTGCATTTCACCCGGTTGCAATAACAAAACTAACAAAACAACACACGCAAAAGGAGTGATAATGCATTGCTTCCCACCGAACATTACAATGATTAAGTTGTGGCTTTTGCAGATCGGCCGAGATTTTGGAGATGTTGATGCC
Kol_27_Nig          ........................................................................................................................
Kol_27_Asa          ....................................................................................................T...................
Lv13_chr9_10L|0     ..........C..GTGT.T.C......................T......A.A.A.............G.......A..CT......A...............A.A.C...T..A......

Tr33_Chr09|1        TTCGCCCAAAAAGTACTGGATCACAAGAAAAATGGCGTGTTTCGTTTGTGTTCAGAGCACTTTGCAGCCGATAGTTATGTAGACCACTGTGCAACAAAGCGGCTTAAAACAGATGCACTGCCCACAATTTTTCCGTACCGGAAAACAGCT
Kol_27_Nig          ........................................................................................................................
Kol_27_Asa          .......G.........................................................................A......................................
Lv13_chr9_10L|0     ..T..A.....A.........G...G.....T.CT.A.................T..CT.C.T..A.......G....T.A..A.............G........GT....T.........TA............

Tr33_Chr09|1        CCCGTATGGGAT------------------ACTGGATTGTCATCT------------------------TTC---------TTTAACATAAACGCCTTATCCGCAAAGTGTTCACTGAGCGCCTCGCCATGTTCTACAAATGAAGGTAGT
Kol_27_Nig          ........................................................................................................................
Kol_27_Asa          ......................................................................................A.................................
Lv13_chr9_10L|0     ..T.CT.AT.C.GAAATATGTGCCCCGTTA.A....GCCCA.AGCTCACCTTCTGCAAATTATACCAGA..TCCATCTGCC.....AG....T..T...G........CCC..T......---A.T....A..A.GT.GGA....A.G..

Tr33_Chr09|1        AGTCGTAATCCCAACAATATATCCACGGGGAAAGCGATTTGCAAATGCGTACGTAAGAAAATGGTGGATGCAAGCACGCTCACGGATCCAGCAATGTTCTCCAAAGATGACTCTTTGCCATGGCAAGAGATACATGGAAGTGGCAAAGGA
Kol_27_Nig          ........................................................................................................................
Kol_27_Asa          ......................................T.............T.........A................................G........................
Lv13_chr9_10L|0     CA..C.T.....G.........T....C...A...............A...........T..C.............A....G.......T.........A.......G......A....A.C.A..........T.

Tr33_Chr09|1        CTGAAA---AGAAAAGGCCACCATGCGAAGTTCTCTAGAATAAGCTGCCGAAATCCTTTAGAGAGTGGTGCCTCTCTAGGTATAAGTAATAGGATGATCTTTTCTAATGACCAACCACAAAGTCAG------GTTTCTGGAATC------
Kol_27_Nig          ........................................................................................................................
Kol_27_Asa          ........................................................................................................................
Lv13_chr9_10L|0     T.....ATA.A.G.T.AT..G..CA.TG.T....TC...---------AAG...TTCCAC..T.C.TCA..GAACGCT.AC---TC.T.......T..T---...G......C......G......GTACGG............CAAGAG

Tr33_Chr09|1        ---------------------AAAGCAAAAACTGGTAGCATTCCAAACATTTCCAGCAGTTCTGTAATAACAGAACCACCCTTCTCCATGCACTATGATGTTGCCATTAGAGATCTTGTGAACGAAAGAAAGTTTATCGTATTCGAGTCT
Kol_27_Nig          ........................................................................................................................
Kol_27_Asa          ........................................................................................................................
Lv13_chr9_10L|0     ACACTTGAAGTTTCCTCCTAC............AC.AT........T..........A......G......G.......................CA....A..C.............T................T..T......

Tr33_Chr09|1        TGCCTCGATGTCTTGCTTCTCAAGCTTTCCTGTGGGTTTGATGTGAACTGCAGTGCTCATATTGTTGCACTTGAGAAGCACATAGAGGGTTCCTGGCTGTCTGTAATTGGACGCTGTTCCAAAGGCCATTGCTTTCATCTGTGGGACAGT
Kol_27_Nig          ........................................................................................................................
Kol_27_Asa          ........................................................................................................................
Lv13_chr9_10L|0     .....T....................C....CA..........G......C.............A...............A..........T....C...T...........A.........CCA.........T........C

Tr33_Chr09|1        CAACCTGTAGCAGGAGACATCGCACTGGGAAATCTATTGACTTCGGCTGCTGTGCTTTTTAGTGGCTCCAATTTTTATAAGGTTGAAGATATGAGTCAGCTTATGGGCCTACAGCTTATTTCCTATGACATGCACTATAATTACCAACGT
Kol_27_Nig          ........................................................................................................................
Kol_27_Asa          ......A.............T.................T....A.........................................................C..................
Lv13_chr9_10L|0     ..G.......T.........T.............T...T.C..C................C.....TGGC........A........A........A...C.A.......................T.CAT.......A.....G..C

Tr33_Chr09|1        ATGTTCCTGTTCCCTACAATTGATAAGTACTGGCAGTGTGAACGCAGAAGGCTCAAAGAAGCAATGGGATGGAAAAAAATGTGCCTTTCTGGAAATAAGCAAGGTTCCAAATACTCTACATACACCTTCACTGACGTCGAGACAAAACAT
Kol_27_Nig          ........................................................................................................................
Kol_27_Asa          ...........T.........................................................C..................................................
Lv13_chr9_10L|0     ..........T.............C.......A.A...G....A......T................C.....G................G.......G.TC.....G.......G.....A...G....T..T.........G.

Tr33_Chr09|1        ATTCTAGATTTTCAAGTAGTGCAGAAGTCAAAGACTTCTACGTCCCTAGAGATGGAAAAGCTCATCTTTGAAACGTGCTTAGATCGACTTCTAGAAGAGCATTATGATGTTGAGGCCATTGTAACAGACCAACATCCCGCTATTGAAGAA
Kol_27_Nig          ........................................................................................................................
Kol_27_Asa          ...................................................G....................................C..A............................
Lv13_chr9_10L|0     ................T..C...C..........AA....A.....T.CA...........AGC.........C......A...........G......A...T..T..............C...............T.....AA.....

Tr33_Chr09|1        CTGATGTGTGAAAAGTACAGTCCTGTCGTTCATAAGTATGAC------GTTTGCTTTTATGCCCAAAAGGTAAAAAGACAGTTGGTGGCCGCCAGCAAAAGGAGAAAATGTTCACAGATTAGCAAGTGGATCCCTGCAATCATCCAACAC
Kol_27_Nig          ........................................................................................................................
Kol_27_Asa          ........C.................T..............T....................................................A.T........................C....................
Lv13_chr9_10L|0     A.A...........A....A............C........TATTTGG..G..T------........................A.A..T.................T........T..............T.................

Tr33_Chr09|1        TTGGAATGGGCTTCCAGAACTAGTCATGGCGATGCAGAATTGCTGCGTGAGAAGTGGCAGTCACTACTCCTGCATGTTACAAACCACGACGAGCTGGATGGTTTCAGGGTCTGCCACGTGTGTTGCAATAAAGCTCTGAATCCCCATTCA
Kol_27_Nig          ........................................................................................................................
Kol_27_Asa          ............................................................C..............A............................................
Lv13_chr9_10L|0     .........T...GG.....C........G.........C.T...T..............A..............CA............A.TA...GT....C..C...A..G.....T.CA......C..............TG.TT....

Tr33_Chr09|1        CGGTCACTTCCCTGGATCAGAAAATGGAGTCCAGCCTTCCATGCTTTACGTGGCGTGATTCTTTCCTCCAAGCTGACAAAGGACTTAGCCTGTCTTTCTCAGTTTAGCAGCGGCAAAGAAGTTACGTTATACCATTGTTTCCTTCAAAAA
Kol_27_Nig          ........................................................................................................................
Kol_27_Asa          ...........................................A............................................................................
Lv13_chr9_10L|0     ...................AG..........TG..G..............A.........T..........A..TG....A.....G.............CA.....T.AT..A.G....A...A..........C....T........

Tr33_Chr09|1        TATAGGCCCAATAAGATCAGTTTCAGGATGGATGCTGTGGATGCAAGGACCAAACTGGCAGCTCTTGCATACAATGCAAATGTACACATGGAACATAAGTTAAGCTTTGTATCCTCGAAGGTGAAGGATAGTGTT---------------
Kol_27_Nig          ........................................................................................................................
Kol_27_Asa          .............................................................T..........................................................
Lv13_chr9_10L|0     .....A....G.G.AG..TA..................................A.........A.........A......G.......G...C.CAAAGCCTAAA........A..A..A.....A......TGCCTACAGAGACAC

Tr33_Chr09|1        ------------------------GACCCATCGCTACTGAAACCACCGCACAGGCTCAACTCAAATACTCATTTGCTGCAAATGATGTTGGATGCTATAAAATTGTGTTCCCTTAACCAG-----------------------------
Kol_27_Nig          ........................................................................................................................
Kol_27_Asa          ........................................C...............................................................................
Lv13_chr9_10L|0     AAAGTAGTTTACTCTGGAACCAACAG......TGA.......T....A.C..AAT.A.........G.........G.A.G..........................C............ATCCAGAACTGCAATTCAAGGTTCACCCTG

Tr33_Chr09|1        ------------CCGTAG
Kol_27_Nig          ..................
Kol_27_Asa          ..............C...
Lv13_chr9_10L|0     CTCCAAGGTGCAAAA...
```

```
Tr6_scaffold_3164|5 ATGCCTAACTGCATTGTTAAAGGATGCCCTCATCGAAGTGGCCAAAAACTGCAGTATCCAGATGTTGTTTTACATCCGTTTCCACGTAATATTCATCAGATAAAAAATTGGTTAATGCAAACTGGACAACATGCAGGTGTCATTGATACC
Kol_28a_Nig         ------------------------------------------------------------------------------------------------------------------------------------------------------
Kol_28a_Asa         ------------------------------------------------------------------------------------------------------------------------------------------------------

Tr6_scaffold_3164|5 TTATCTGATAGAATTTTACAAGGTACCAAAAATTCAAGTTTCAGAATGTGCTCTCAACATTTCACACCTGATTGTTATATGATGCAGGGTTCAAGAAGATTTCTGAAACCAAATGCAGTTCCAACCATTTTTGCAAGCATACCTGTTCCA
Kol_28a_Nig         ------------------------------------------------------------------------------------------------------------------------------------------------------
Kol_28a_Asa         ------------------------------------------------------------------------------------------------------------------------------------------------------

Tr6_scaffold_3164|5 GTTGTAATTAATGTATTTGAATCTGATACAACACTGCCATCCACAAAAAGGAAAAGAGTGGATAATGAGCAACCTTCCACCTCAAGTCAACTGGTCAGAATAGTCTCACGTTTAGTAACTGTAGCAACACAAACAGAGATTATTACAAAG
Kol_28a_Nig         ------------------------------------------------------------------------------------------------------------------------------------------------------
Kol_28a_Asa         ------------------------------------------------------------------------------------------------------------------------------------------------------

Tr6_scaffold_3164|5 ACTTGTGCAACAATGACATCCAAATCTAAGTTCTCTCGCCAATCGGCTACACAAACCACTGGAACTGTAGGGAAAGAAATAAGTACACAAACTGAAGAAGTCTTTTATGCAGATGTTAACCAACTAAGAAAAGACCATACTTATCCAGTA
Kol_28a_Nig         ------------------------------------------------------------------------------------------------------------------------------------------------------
Kol_28a_Asa         ------------------------------------------------------------------------------------------------------------------------------------------------------

Tr6_scaffold_3164|5 TGTTTTTCTACACCGGTAAAATCAATGCAACCCACCTGCGATATTTTTTCTGCTTCTCCCATTGCACCAACATTGCAACCTCTTGAGAACCTTGAAACACCATCTAAAGATCTAGGTTCACAAGTTGAACAAGAATCCATAATTTGTTCT
Kol_28a_Nig         ------------------------............A.................................................................................................................
Kol_28a_Asa         ------------------------............A.................................................................................................................

Tr6_scaffold_3164|5 ACTCCAACGGCTACAAACCCCAGAGATACGACTTTTGAGACATCAGAGCAATCTTCATCTGACAATGCATCAATCAAAAGTCAAGAAGAGACTGCTGTGAGTGGTCGATCAATGTATGTGCAGAAAAGAAATTTTATAATATATGAAGAG
Kol_28a_Nig         .............................A..........T...............................................................A.............................................
Kol_28a_Asa         .............................A..........T...............................................................A.............................................

Tr6_scaffold_3164|5 AATTTAGATGACTTGTTCAAACTAGTCAAATGCCAGCACAGTATTGAATCACCCTGTCAAGCACCTATAATACAAATAAAAAAAACTACAGAGGGAAGCATGTTAAAAGTCCAACTGATTTGTTTGAATGGACATGATTCTTTCAAATGG
Kol_28a_Nig         ......................................................................................................................................................
Kol_28a_Asa         ......................................................................................................................................................

Tr6_scaffold_3164|5 ATGTCACAACCAATGGCAGGGGCCACGTCTATTGGCAATGTAGCACTGGCAAATTCAATTGTTCTCAGCGGTTCAACTTACACAAAAGTTAAAGATATGTTCAACATTCTGAATGTGCCTTTCTATTCTCATACCAGTTTTTACCGCTAC
Kol_28a_Nig         ......................A..................................................................G............................................................
Kol_28a_Asa         ......................A..................................................................G............................................................

Tr6_scaffold_3164|5 CAACGAGAATATATTTTTCCAGCTATAGATAAACAGTGGCAACAAGAAACAGCTGCCGTTAAGACATCCTTATCGGGACAATCTGTTGCCTTAGCAGGAGATGGACAATTCGATAGCCCGGGACATTCGGCAAAGTATTGTACGTACACT
Kol_28a_Nig         ...........................................................A....TC..........................................................................C..A......
Kol_28a_Asa         ...........................................................A....TC..........................................................................C..A......

Tr6_scaffold_3164|5 ACGATGGACCTTCTAAGCAAAAAAATTGTACATTTTGAAATTCAACAATTGGGTACTGTGAAAAATTCTTACGCTTTAGAACAATCATCATTTACAAAATGTTTGCAAACCTTGCTTGATGAGAAGGTAAAAGTCAAAATACTTGCAACA
Kol_28a_Nig         ........................................................C.............................GG.....................................A...............G........
Kol_28a_Asa         ........................................................C.............................GG.....................................A...............G........

Tr6_scaffold_3164|5 GATAGACACAGTGGTATTCGAGCATTGATGAAAAGCAAATTTAAAAAAATAGACCACCAGTTTGATGTGTGGCATCTTTGCAAAAGTATTCAGAAAAAATTATTAGCGGCAAGTAAGAAAAGGAAATGTCGTGATATTGCACACTGGATC
Kol_28a_Nig         ...............................................C......................................................................................................
Kol_28a_Asa         ...............................................C......................................................................................................

Tr6_scaffold_3164|5 AATGCAATTACCAATCACCTTTGGTGGTCATCTCAAACCTGCAAACAAAATGTCAATGTTTTGCTAGCGAAATGGAAGTCGGTACTTTTTCATGTTGCTAACAAGCACACATTCAAATCTTTAAAATACTACAAAAAATGTCAACATCGC
Kol_28a_Nig         ................................................................................A..................T..........................C.......................
Kol_28a_Asa         ................................................................................A.............................................C.......................

Tr6_scaffold_3164|5 CGACTTACTGCTGCACAGAAAAAAGACGCACGATGGATTGCTTGTAATCATCCGGCACACTCCACTCTTAAAGAAATCATCAATGACCGATTTTTGTTGCGTGATATTGCTCAAACAGAGAAATTTTGTCACACTGGTGATTTGGAAAAT
Kol_28a_Nig         ..........A.............................G...C.........................................................................................................
Kol_28a_Asa         ..........A.............................G...C.........................................................................................................

Tr6_scaffold_3164|5 TTTCACAGCAAAGTTCTCAAATACAAGCCCAAAAGGATTTCTTTTAAAATGGATGCAATGATTGCAAGAACAAGTCTGGGAGCTTTGTCACATAACAGAAACGTCCATAGACATCAGGCTGTTGTTAAAAGAGCAACAAAAACATCTTTG
Kol_28a_Nig         .........................................C............................................................................................................
Kol_28a_Asa         .........................................C............................................................................................................

Tr6_scaffold_3164|5 GCTTTTGGCGAAAAAAGATTTAAAATAGTGTTTCCAAAAATGAAAAAAGATTGGATTGCAAAGCCAGTATACGAGGACGTGATTGATGAACATCTTTTGGACATCACGTCAGACTCTATAAAAATATTAACTGGTGAGATTGGACATGAC
Kol_28a_Nig         ..........................C......................................................GA.................................A..............G..................
Kol_28a_Asa         ..........................C......................................................GA.................................A..............G..................

Tr6_scaffold_3164|5 TGGGTTTCCATCTCAAAGCAGATGCCACAAAATATTGCCACCACTGAACGTCCAGAAAAAAGTGAAGTAATTGAAAAACATATGTCTCGTTTTTCAAGATGTTAA
Kol_28a_Nig         .........................................................................................................
Kol_28a_Asa         .........................................................................................................
```
